# Enantioselective and collective total synthesis of pentacyclic 19-*nor*-clerodanes†

**DOI:** 10.1039/d3sc04335e

**Published:** 2023-10-23

**Authors:** Zhi-Mao Zhang, Junliang Zhang, Quan Cai

**Affiliations:** a Department of Chemistry and Research Center for Molecular Recognition and Synthesis, Fudan University 220 Handan Rd. Shanghai 200433 China quan_cai@fudan.edu.cn

## Abstract

We report herein the collective asymmetric total synthesis of seven pentacyclic 19-*nor*-clerodane diterpenoids, namely (+)-teucvin (+)-cracroson A, (+)-cracroson E, (+)-montanin A, (+)-teucvisin C, (+)-teucrin A, and (+)-2-hydroxyteuscorolide. An ytterbium-catalyzed asymmetric inverse-electron-demand Diels–Alder reaction of 4-methyl-2-pyrone with a chiral C5-substituted cyclohexa-1,3-dienol silyl ether is the key feature of the synthesis, which provides the common *cis*-decalin intermediate with five continuous stereocenters in excellent yield and stereoselectivity. From this diversifiable intermediate, the total synthesis of (+)-teucvin and (+)-2-hydroxyteuscorolide was realized in thirteen and eighteen steps, respectively. From (+)-teucvin, five other pentacyclic 19-*nor*-clerodanes were divergently and concisely generated through late-stage oxidation state adjustments.

## Abstract 2

The concise and collective total synthesis of seven pentacyclic 19-*nor*-clerodane diterpenoids were achieved by a catalytic asymmetric inverse-electron-demand Diels–Alder (IEDDA) reaction and late-stage oxidative transformations.

## Graphical abstract

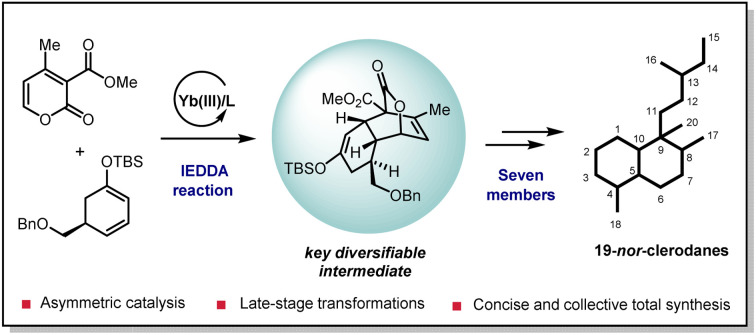

## Introduction

**Fig. 1:**
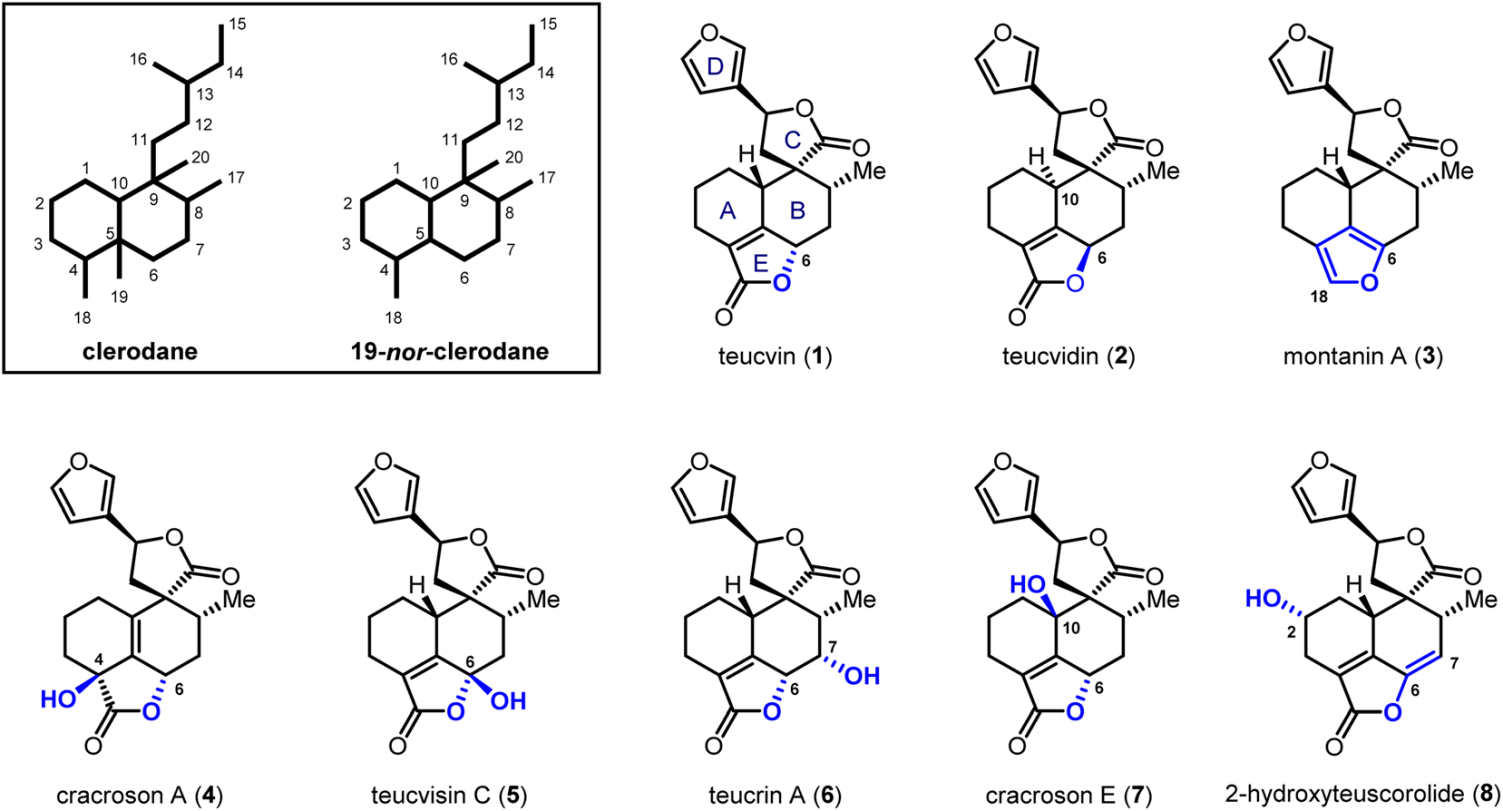
Representative pentacyclic 19-*nor*-clerodane diterpenoids.

Clerodane diterpenoids and their 19-*nor* variants are a diverse group of natural products; over 1300 family members have been isolated.^1^ Fascinating biological and pharmacological activities are shown by this class of compounds, including insect antifeedant,^2^ selective *κ* opioid receptor agonist,^3^ anti-cancer,^4^ anti-inflammatory^5^ and antibiotic activities.^6^ Consequently, the total synthesis of clerodanoids has attracted extensive attention from the synthetic community for decades.^7^ Among these molecules, pentacyclic 19-*nor*-clerodanes are attractive synthetic targets owing to their intriguing structures, which feature a compact and densely-functionalized decalin core, a spiro γ-lactone unit, and a fused α,β-unsaturated-γ-lactone moiety (Fig. 1). This unique type of polycyclic structure provides a great platform for the development of new synthetic strategies. For instance, the Williams group explored a creative 6π-electrocyclization for the construction of ABC tricyclic ring scaffold in a concise fashion.^8^ Collaborating with Ley *et al.*, the Williams group also developed a late-stage Diels–Alder approach for the synthesis of the main decalin scaffold with the furanospiro-γ-lactone moiety and most of the required functionalities.^9^ Despite these encouraging achievements, total syntheses of pentacyclic 19-*nor*-clerodanes are still quite challenging due to their structural complexities; only two research groups have succeeded in this area to date.^10,11^ In 2003, the Liu group reported the first total synthesis of teucvin (1) in a racemic form *via* the normal-electron-demand Diels–Alder reaction,^10*a*^ and then realized the racemic total synthesis of teuscorolide and montanin A (3) by the same strategy.^10*b*^ In 2012, the Lee group furnished the first enantioselective total synthesis of (−)-teucvidin (2) through an elegant Michael/Conia-ene cascade cyclization based on the chiral pool strategy.^11^ However, to the best of our knowledge, the total synthesis of more complex pentacyclic 19-*nor*-clerodanes with higher oxidation states (*e.g.*4–8), especially in an enantioselective manner, has yet to be realized.

Compared with the traditional “single-target” strategy, the collective total synthesis of a range of structurally diverse natural products from a common intermediate has emerged as a powerful technique in organic synthesis.^12^ This strategy would not only trace biosynthetic relationships within targeted molecules, but also provide great opportunities for their comprehensive biological evaluation. Structurally, teucvin (1) is one of the most representative family members of pentacyclic 19-*nor*-clerodanes.^13^ Decorating hydroxyl groups at different positions (C2–C10) on the decalin moiety of teucvin (1) generates various types of family members (4–8). Based on this structural analysis, we plan to develop a unified approach for the collective total synthesis of pentacyclic 19-*nor*-clerodane diterpenoids. First, an efficient method for the synthesis of a decalin intermediate with multiple contiguous stereogenic centers and most of the required functionalities should be developed. From this common intermediate, the total synthesis of teucvin (1) and other family members could be accomplished concisely. Going forward, further transformations to adjust the oxidation states of teucvin (1) and introduce oxygenated functionalities regioselectively and stereoselectively are required to realize the divergent synthesis of clerodane congeners with higher oxidation states.

**Scheme 1:**
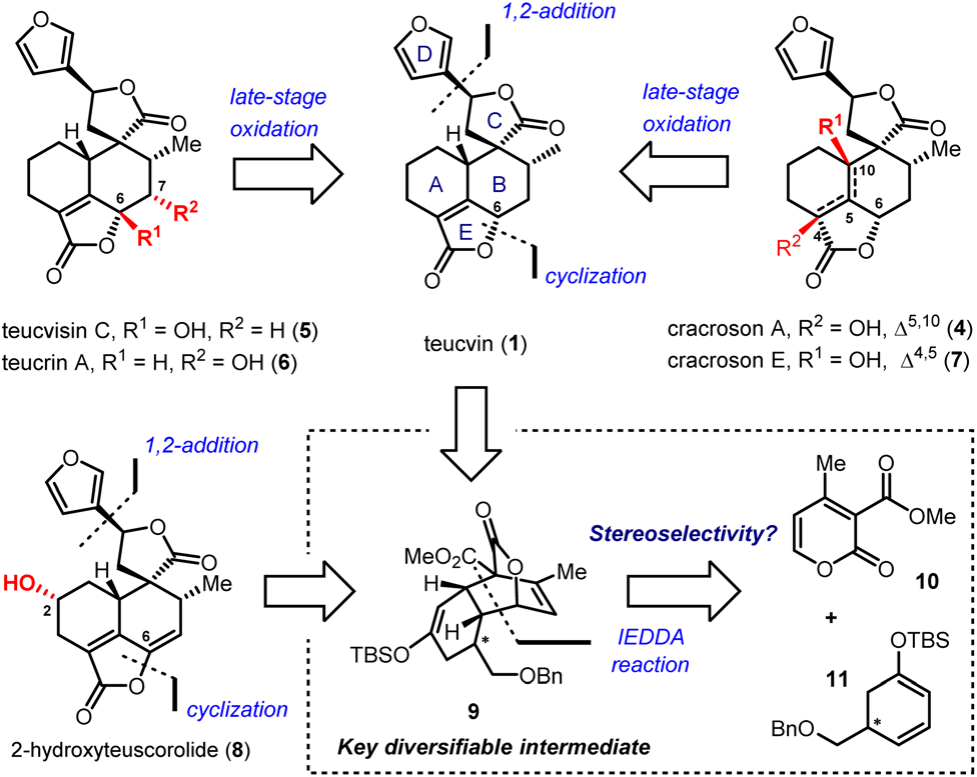
Retrosynthetic analysis based on the 2-pyrone-based IEDDA reaction and late-stage oxidative transformations.

2-Pyrone-based Diels–Alder reactions are a prominent method for the construction of six-membered carbocycles.^14^ Recently, catalytic asymmetric inverse-electron-demand Diels–Alder (IEDDA) reactions of 2-pyrones have attracted increasing attention owing to their great potential in the synthesis of bioactive natural products, which was demonstrated by Markó,^15^ Posner,^16^ Gademann,^17^ de la Torre^18^ and our group.^19^ In 2020, our group has developed an efficient method for the construction of densely functionalized *cis*-decalin scaffolds by ytterbium-catalyzed asymmetric IEDDA reactions of 2-pyrones and then applied it to the total synthesis of terpenoids 4-amorphen-11-ol and *cis*-crotonin.^19*b*^ Based on this reaction, we designed a general and unified approach for the collective synthesis of pentacyclic 19-*nor*-clerodane diterpenoids. As shown in Scheme 1, the key diversifiable intermediate 9 bearing five continuous stereogenic centers was generated through the catalytic asymmetric IEDDA reaction between 4-methyl-2-pyrone 10 and C5-substituted cyclohexa-1,3-dienol silyl ether 11 with a suitable absolute configuration. From tricyclic lactone 9, both teucvin (1)^13^ and 2-hydroxyteuscorolide (8)^20^ could be generated by downstream transformations involving the stereoselective 1,2-addition to attach the 3-furyl group, and the cyclization step to construct the α,β-unsaturated-γ-lactone E ring. From teucvin (1), we proposed that by sophisticated late-stage manipulations of the oxidation state on the decalin scaffold, five other 19-*nor*-clerodanoids, including montanin A (3),^21^ cracroson A (4),^22^ teucvisin C (5),^23^ teucrin A (6),^24^ and cracroson E (7),^25^ could be synthesized divergently.

## Results and discussion

### Preparation of the common synthetic intermediate *anti*-9

**Scheme 2:**
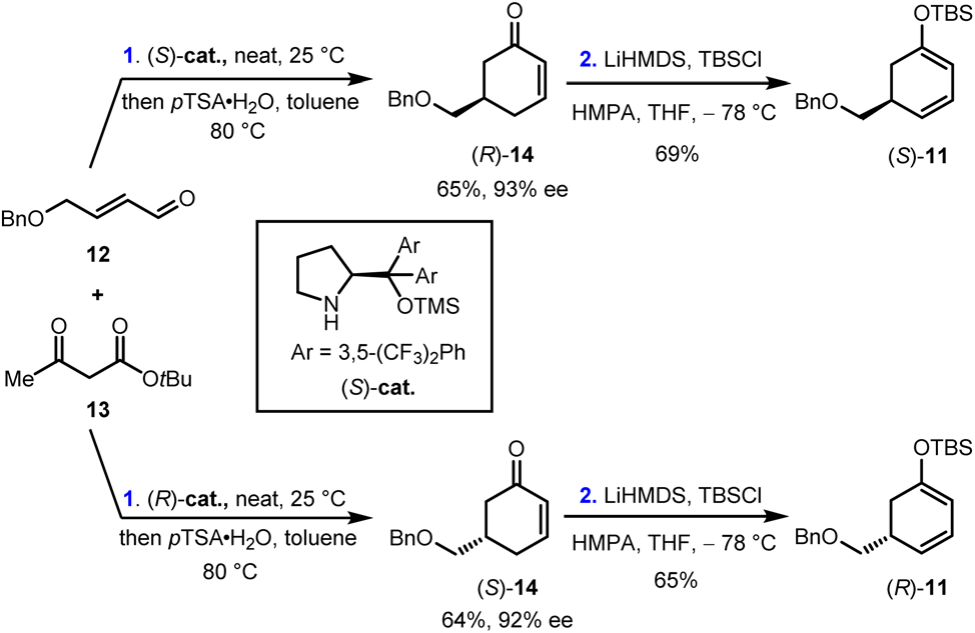
Synthesis of enantiopure cyclohexa-1,3-dienol silyl ether (*S*)-11 and (*R*)-11 by an organocatalyzed asymmetric Robinson annulation. *p*TSA, *p*-toluenesulfonic acid; LiHMDS, lithium bis(trimethylsilyl)amide; TBSCl, *tert*-butyl dimethylsilyl chloride; HMPA, hexamethylphosphoramide.

Our synthesis began with the preparation of enantiopure cyclohexa-1,3-dienol silyl ether (*S*)-11 and (*R*)-11. As shown in Scheme 2, the Robinson annulation of α,β-unsaturated aldehyde 12 with *tert*-butyl acetoacetate 13 catalyzed by (*S*)-cat. Followed by the decarboxylation under acidic conditions gave cyclohexenone (*R*)-14 in 65% yield and 93% ee.^26^ (*R*)-14 was then treated with LiHMDS and TBSCl to provide (*S*)-11 in 69% yield. Enantiomeric (*R*)-11 was prepared in 42% yield and 92% ee over two steps using (*R*)-cat. as the catalyst. Furthermore, (*rac*)-11 was also synthesized by the same reaction sequence (see ESI for details†).

With these C5-substituted cyclohexa-1,3-dienol silyl ethers in hand, the pivotal IEDDA reaction was investigated. Initially, the IEDDA reaction of 4-methyl-2-pyrone 10 with enantiopure (*S*)-11 (93% ee) was attempted in the presence of catalytic amounts of Yb(OTf)_3_ without a chiral ligand (Table 1, entry 1). This reaction proceeded smoothly but showcased only moderate substrate-control for the stereoselectivity, giving *anti*-9 and *ent-syn*-9 as a 3.1 : 1 mixture in 78% yield and 94% ee. Then the effect of kinetic resolution in the IEDDA reaction was investigated (Table 1, entry 2). Upon treating 2-pyrone 10 (1.0 equiv.) and (*rac*)-11 (2.0 equiv.) with Yb(OTf)_3_ and (*S*)-L1, a mixture of *anti*-9 (78% ee) and *syn*-9 (90% ee) was generated in 73% yield and 1.2 : 1 dr. Although the diastereoselectivity was not good, the high ee of *syn*-9 bearing a sterically hindered substituent on the concave side of the *cis*-decalin scaffold indicated that very good stereocontrol might be induced by the chiral catalyst. To confirm this hypothesis, we investigated the reaction using sterically mismatched (*R*)-11 (92% ee) as the dienophile (Table 1, entry 3). Intriguingly, the thermodynamically disfavored product *syn*-9 was afforded as the main product with >99% ee and 3.8 : 1 dr. Encouraged by these findings, to improve the performance of the IEDDA reaction, sterically matched (*S*)-11 (93% ee) and Yb(OTf)_3_/(*S*)-L1 were used (Table 1, entry 4). To our great delight, through synergistic stereo-control of the substrate and the chiral catalyst, *anti*-9 with the right stereochemistry was afforded as the main product with a dramatically increased 98% ee, 10 : 1 dr, and 87% total yield in a gram scale (4.34 g).

**Table 1:** Investigations of the pivotal IEDDA reaction (the second step of asymmetric catalysis)^a^

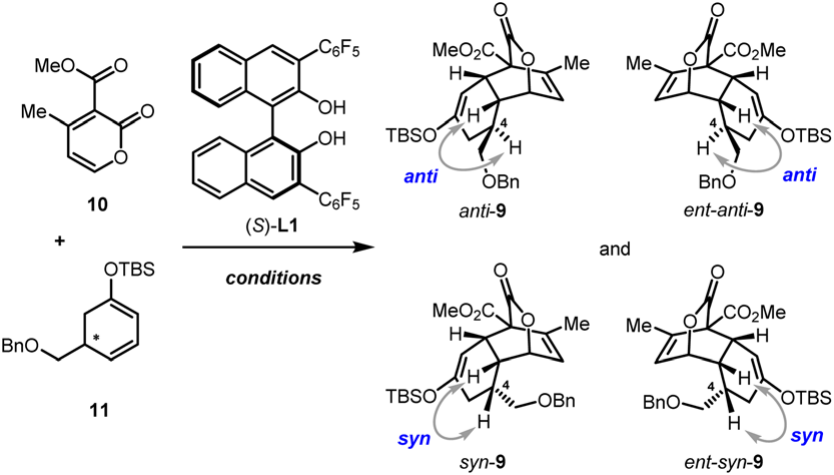			
Entry	Conditions	Results	Products
1^b^	(*S*)-11 (1.5 equiv.)	78%, 3.1 : 1 dr	*anti*-9, 94% ee
	Yb(OTf)_3_		*ent-syn*-9, 94% ee
2	(*rac*)-11 (2.0 equiv.)	73%, 1.2 : 1 dr	*anti*-9, 78% ee
	Yb(OTf)_3_, (*S*)-L1, DIPEA		*syn*-9, 90% ee
3	(*R*)-11 (1.5 equiv.)	75%, 3.8 : 1 dr	*syn*-9, >99% ee
	Yb(OTf)_3_, (*S*)-L1, DIPEA		*ent-anti*-9, 72% ee
4	(*S*)-11 (1.5 equiv.)	87%, 10 : 1 dr (4.34 g)	*anti*-9, 98% ee
	Yb(OTf)_3_, (*S*)-L1, DIPEA		*ent-syn*-9, 19% ee

a Reaction conditions: 10 (0.10 mmol), 11 (0.15 mmol), Yb(OTf)_3_ (10 mol%), (*S*)-L1 (12 mol%), DIPEA (24 mol%), 4 Å M.S. (25 mg), DCM (0.25 mL) at 25 °C.

b (*S*)-L1 was not added. DCM, dichloromethane; DIPEA, *N*,*N*-diisopropylethylamine; M.S., molecular sieves.

### Total synthesis of (+)-teucvin

**Scheme 3:**
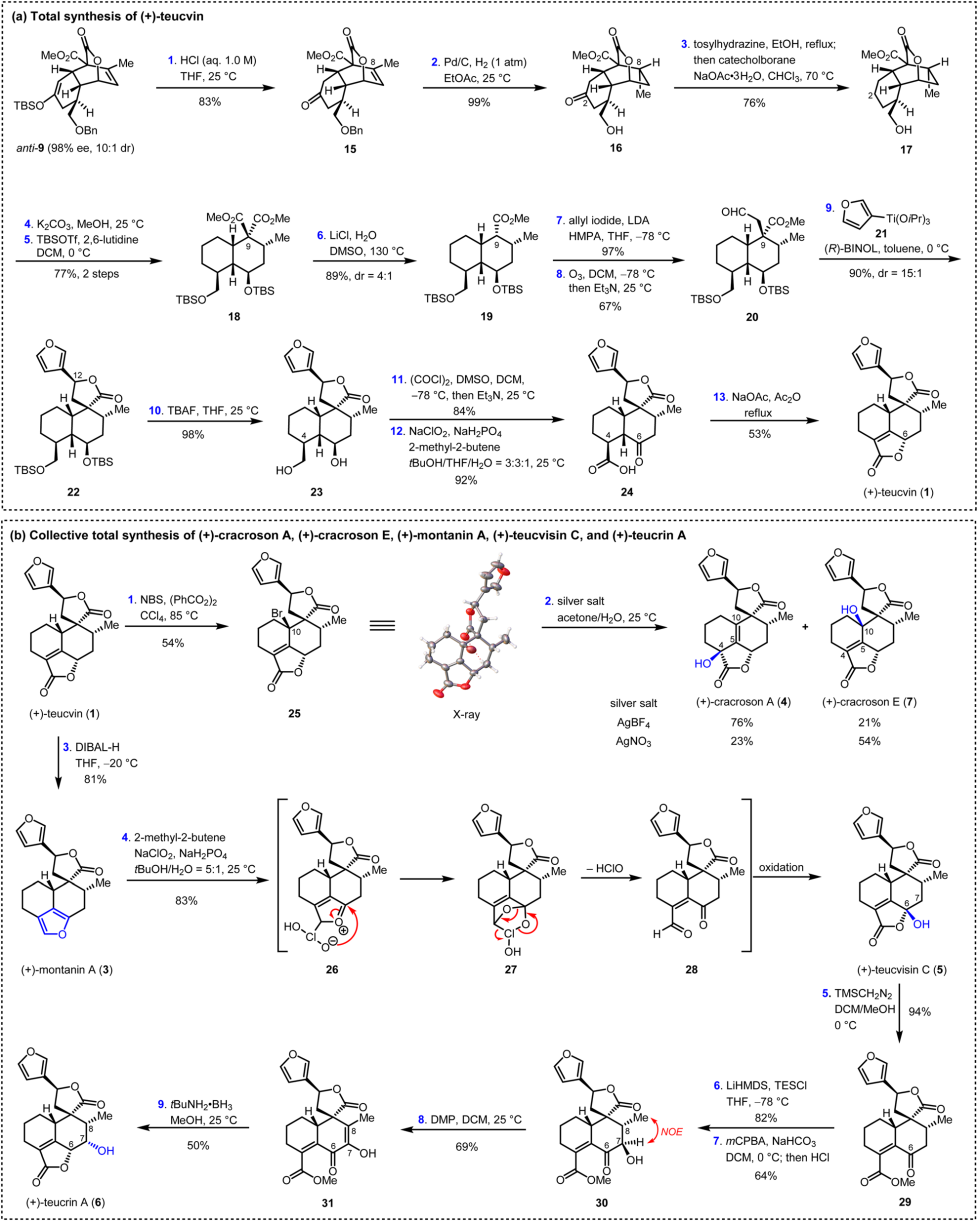
Total synthesis of six pentacyclic 19-*nor*-clerodane diterpenoids. THF, tetrahydrofudran; DMSO, dimethyl sulfoxide; TBSOTf, *tert*-butyldimethylsilyl trifluoromethanesulfonate; LDA, lithium diisopropylamide; BINOL, 1,1′-binaphthol; TBAF, tetrabutylammonium fluoride; NBS, *N*-bromosuccinimide; DIBAL-H, diisobutylaluminum hydride; *m*CPBA, *meta*-chloroperoxybenzoic acid; DMP, Dess–Martin periodinane.

Having succeeded in preparing the key intermediate *anti*-9 with high enantiopurity (98% ee), the stage was now set for the total synthesis of teucvin (1), as summarized in Scheme 3a. Thus, *anti*-9 was hydrolyzed under acidic conditions to afford ketone 15 in 83% yield. The stereoselective hydrogenation (Pd/C, 1 atm H_2_) of the trisubstituted olefin through the convex side of the *cis*-decalin framework of 15, with simultaneous deprotection of the benzyl group, gave lactone 16 in 99% yield with the required configuration at C8. Then, the C2-carbonyl group of 16 was removed by treatment with tosylhydrazine followed by the reduction of catecholborane to furnish 17 in 76% yield.^27^ Ring opening of the lactone group of 17 (K_2_CO_3_, MeOH) led to the diol intermediate, which was immediately protected with silyl groups (TBSOTf, 2,6-lutidine) to furnish diester 18 in 77% yield. Removal of one of the two ester groups in 18 by Krapcho decarboxylation^28^ gave 19 with 4 : 1 dr at C9 in 89% overall yield. Then, the addition of the anion generated from 19 and LDA to allyl iodide followed by ozonolysis (O_3_, Et_3_N) proceeded smoothly to afford aldehyde 20 in 65% yield over two steps. To construct the C12 stereogenic center, several approaches for the 1,2-addition of 3-furanyl metal reagent to aldehyde 20 were examined (see ESI for details†). To our great delight, it was found that the stereoselective BINOL-catalyzed 1,2-addition of (3-furyl)Ti(O*i*Pr)_3_ to aldehyde 20 along with spontaneous lactonization constructed the spiro γ-lactone motif very efficiently,^29^ generating 22 in 90% yield and 15 : 1 dr at C12. Global deprotection of the silyl groups by TBAF followed by sequential Swern oxidation [(COCl)_2_, DMSO]^30^ and Pinnick oxidation (NaClO_2_, NaH_2_PO_4_)^31^ led to γ-keto acid 24 in 76% yield over three steps. From 24, the α,β-unsaturated-γ-lactone E ring was constructed by treatment with NaOAc/Ac_2_O under reflux, thus affording (+)-teucvin (1) in 53% yield.

### Total synthesis of (+)-cracroson A, (+)-cracroson E, (+)-montanin A, (+)-teucvisin C, and (+)-teucrin A

Based on the reliable synthetic route to (+)-teucvin (1), we now embarked on the collective total synthesis of more complex pentacyclic 19-*nor*-clerodanes, including (+)-cracroson A (4), (+)-cracroson E (7), (+)-montanin A (3), (+)-teucvisin C (5), and (+)-teucrin A (6), by oxidative transformations (Scheme 3b). Initially, we planned to obtain (+)-cracroson A (4) and (+)-cracroson E (7) by direct allylic oxidation of (+)-teucvin (1). However, this strategy was unfruitful (see ESI for details†). Intriguingly, it was discovered that bromination of 1 by NBS in the presence of benzoyl peroxide in refluxing CCl_4_ regioselectively and stereoselectively generated allylic bromide 25 in 54% yield.^32^ The structure of 25 was unambiguously confirmed by X-ray crystallographic analysis (CCDC 2274928). With 25 in hand, we decided to accomplish the synthesis of (+)-cracroson A (4) and (+)-cracroson E (7) by the Ag-promoted hydrolysis.^32,33^ To our delight, in the presence of AgOTf (3.0 equiv.), hydrolysis of 25 in acetone/H_2_O at room temperature occurred to afford (+)-cracroson A (4) in 66% yield and (+)-cracroson E (7) in 28% yield (Table 2, entry 1). Since the hydroxyl group was installed with a reserved configuration in both (+)-cracroson A (4) and (+)-cracroson E (7), we envisaged an allylic carbocation intermediate was generated. Therefore, to improve the regioselectivity of this Ag-promoted hydrolysis, we investigated various silver salts with different counterions, which might influence the distribution of products owing to the different electronic or steric effect (Table 2, entries 1–7). Of note, when AgBF_4_ was used as the promoter, (+)-cracroson A (4) was generated as the major product in 76% yield, along with (+)-cracroson E (7) as the minor product in 21% yield (Table 2, entry 4). Very interestingly, the utilization of AgNO_3_ could reverse the regioselectivity, giving (+)-cracroson E (7) as the major product in 54% yield and (+)-cracroson A (4) as the minor product in 23% yield (Table 2, entry 6). The structure of (+)-cracroson E (7) was confirmed by X-ray crystallographic analysis (CCDC 2279407).

**Table 2:** Investigations of the hydrolysis reaction of allylic bromide 25^a^

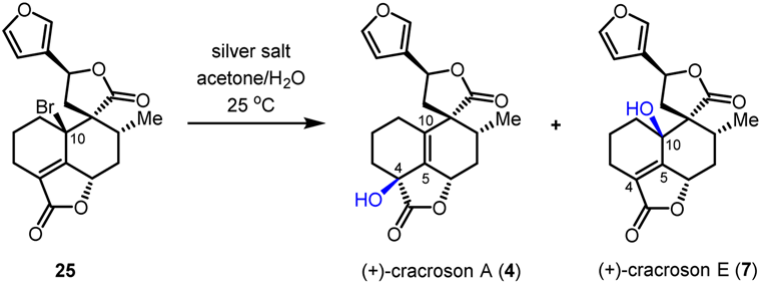				
Entry	Silver salt	Cracroson A^b^ (%)	Cracroson E^b^ (%)	Ratio
1	AgOTf	66%	28%	2.4 : 1
2	AgNTf_2_	49%	33%	1.5 : 1
3	AgSbF_6_	47%	15%	3.1 : 1
4	AgBF_4_	76%	21%	3.6 : 1
5	AgClO_4_	70%	17%	4.1 : 1
6	AgNO_3_	23%	54%	1 : 2.3
7	Ag_2_CO_3_	Trace	Trace	—

a Reaction conditions: 25 (0.01 mmol), silver salt (0.03 mmol), acetone/H_2_O (v/v = 9 : 1, 0.70 mL) at 25 °C.

b Isolated yield.

Additionally, selective reduction of the α,β-unsaturated-γ-lactone group in (+)-teucvin (1) to hemiacetal, which was followed by aromatic elimination, gave (+)-montanin A (3) in 81% yield (Scheme 3b).^10*b*,34^ After judicious selection of the oxidative reaction conditions, it was found that treatment of (+)-montanin A (3) with NaClO_2_ and NaH_2_PO_4_ directly afforded (+)-teucvisin C (5) in 83% yield.^35^ We proposed this transformation involved a [4 + 2] cycloaddition between 3 and *in situ* generated HClO_2_ to give intermediate 27. The latter underwent rapid ring opening to afford the γ-keto aldehyde intermediate 28, which was then converted to (+)-teucvisin C (5) through Pinnick oxidation and cyclization.

From (+)-teucvisin C (5), the total synthesis of (+)-teucrin A (6) with five continuous stereogenic centers was realized for the first time. As shown in Scheme 3b, the reaction of tecuvisin C (5) with TMSCH_2_N_2_ efficiently gave γ-keto ester 29 in 94% yield. Treating 29 with LiHMDS and TESCl followed by the Rubottom oxidation^36^ by *m*CPBA gave 30 in 52% yield over two steps, in which the C7-hydroxyl group exhibited the opposite configuration compared with (+)-teucrin A (6) (determined by NOE spectrum). To reverse the configuration, Dess–Martin periodinane was applied to oxidize the C7-hydroxyl group. The resulting intermediate 31 was then exposed to *t*BuNH_2_ BH_3_,^37^ and total synthesis of (+)-teucrin A (6) was accomplished by stereoselective reduction of the α-keto enol group followed by spontaneous lactonization.

### Total synthesis of (+)-2-hydroxyteuscorolide

**Scheme 4:**
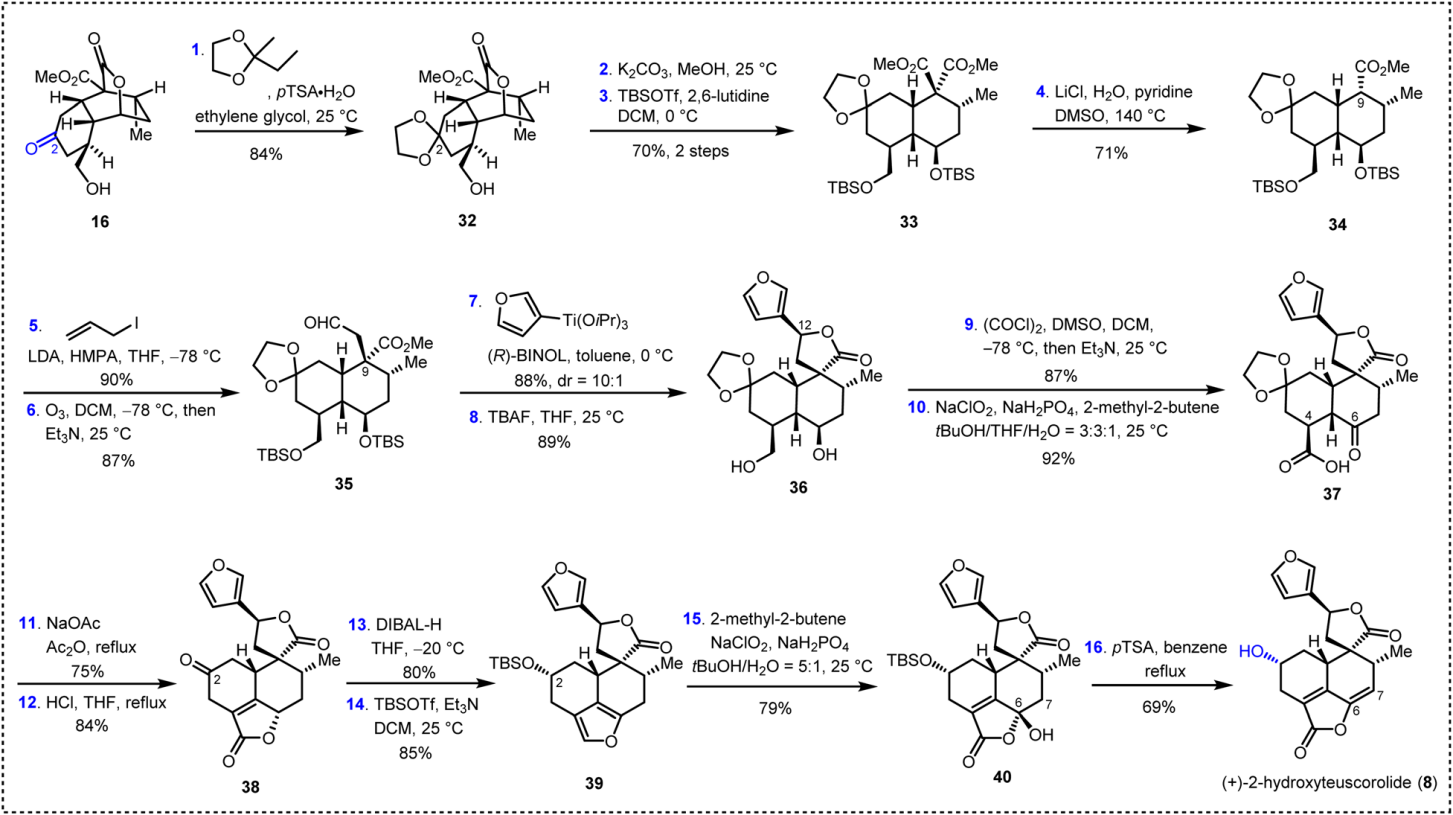
The first and enantioselective total synthesis of (+)-2-hydroxyteuscorolide (8).

We then turned our attention to the total synthesis of (+)-2-hydroxyteuscorolide (8) to demonstrate the versatility of our synthetic route. Compared with other family members, 2-hydroxyteuscorolide possesses a hydroxyl group on the C2 position of the decalin scaffold, which was located far away from the convertible α,β-unsaturated-γ-lactone structural motif. Therefore, late-stage installation of this essential hydroxyl group from (+)-teucvin (1) by oxidative transformations might require lengthy steps. Consequently, our synthesis commenced with the utilization of 16 as the key intermediate, of which the C2 carbonyl group served as a handle for the introduction of the hydroxyl functionality. As depicted in Scheme 4, protection of the carbonyl group in 16 with 2-methyl-2-ethyl-1,3-dioxolane resulted in the formation of ketal 32 in 84% yield. Ring opening of the lactone group and subsequent protection of the diol with TBS groups led to diester 33 in 70% yield. Krapcho decarboxylation of 33 gave 34 in 71% yield. Then, allyl substitution followed by ozonolysis of the resulting olefin afforded aldehyde 35 in 78% yield over two steps. The reaction of 35 with (3-furyl)Ti(O*i*Pr)_3_ in the presence of a BINOL ligand provided the desired spiro γ-lactone intermediate, which was then treated with TBAF to furnish 36 in 78% yield over two steps. Subsequent Swern oxidation and Pinnick oxidation led to γ-keto acid 37 (80% yield, two steps). Sequential exposure of the latter to NaOAc in refluxing Ac_2_O (ring closure) and then HCl (deprotection) led to 38 in 63% overall yield. Interestingly, treatment of 38 with DIBAL-H reduced the C2 ketone and the α,β-unsaturated-γ-lactone simultaneously to install the C2 hydroxyl group with the desired configuration and form the furan moiety. The resulting intermediate was then protected with the TBS group to afford 39 in 68% yield over two steps. Finally, oxidation of the latter with NaClO_2_ and NaH_2_PO_4_ followed by dehydration and concomitant deprotection with *p*TSA accomplished the first total synthesis of (+)-2-hydroxyteuscorolide (8).

## Conclusions

In conclusion, asymmetric total synthesis of seven pentacyclic 19-*nor*-clerodanes was achieved through a divergent approach. Among them, the total synthesis of four members, including (+)-cracroson A, (+)-cracroson E, (+)-teucrin A and (+)-2-hydroxyteuscorolide, was realized for the first time and enantioselectively. The efficient catalytic asymmetric inverse-electron-demand Diels–Alder reaction of 3-carbometh-oxyl-2- pyrone with a chiral C5-substituted cyclohex-a-1,3-dienol silyl ether was developed to construct the central highly functionalized decalin ring. The key diversifiable intermediate with five continuous stereocenters was prepared with excellent yield and stereoselectivity through the synergistic stereo-control of the chiral catalyst and substrate. From this common intermediate, total synthesis of (+)-teucvin and (+)-2-hydroxyteuscorolide was accomplished in 13 and 18 steps, respectively. Five other pentacyclic 19-*nor*-clerodanes were synthesized collectively through late-stage oxidative transformations of (+)-teucvin. We are currently applying the IEDDA reaction of 2-pyrones to the synthesis of other related diterpenoids, which will be reported in due course.

## Data availability

The experimental procedures, copies of all spectra data and full characterization have been deposited in the ESI.†

## Author contributions

Q. C. conceived and directed the project and wrote the manuscript with assistance from Z.-M. Z.; Q. C. and Z.-M. Z. designed the synthetic route. Z.-M. Z. performed the experiments and analyzed the results. Q. C. and Z.-M. Z. interpreted the results and comments on the paper.

## Conflicts of interest

There is no conflict of interest to report.

## Supplementary Material

SC-014-D3SC04335E-s001

SC-014-D3SC04335E-s002
