# Supplementary material for: Enantioselective and collective total synthesis of pentacyclic 19-nor-clerodanes
Source: Chem Sci. 2023 Oct 23;14(44):12598–605. doi: 10.1039/d3sc04335e (PMC10646913; doi:10.1039/d3sc04335e)
Supplement: SC-014-D3SC04335E-s001 [file SC-014-D3SC04335E-s001.pdf]

Electronic Supplementary Material (ESI) for Chemical Science.
This journal is © The Royal Society of Chemistry 2023

Supporting Information

Enantioselective and Collective Total Synthesis of Pentacyclic 19-*nor*-Clerodanes

Zhi-Mao Zhang, Junliang Zhang and Quan Cai*

Department of Chemistry and Research Center for Molecular Recognition and Synthesis
Fudan University, 220 Handan Rd., Shanghai 200433, China
E-mail: quan_cai@fudan.edu.cn

Table of Contents

1. General Methods ···································· 2

2. Synthesis of 1,3-Cyclohexadiene Silane Ether **11** ···································· 3

3. Investigation of the Pivotal IEDDA Reaction ···································· 9

4. Total Synthesis of (+)-Teucvin ···································· 15

5. Total Synthesis of (+)-Cracroson A and (+)-Cracroson E ···································· 25

6. Total Synthesis of (+)-Montanin A and (+)-Teucvisin C ···································· 28

7. Total Synthesis of (+)-Teucrin A ···································· 29

8. Enantioselective Synthesis of (+)-2-Hydroxyteuscorolide···································· 33

9. Comparison of Natural Products with Synthetic 19-*nor*-Clerodane Diterpenoids ···················· 44

10. X-ray Crystallographic Data of **25**, (+)-Cracroson E (**7**) and **38** ···································· 58

11. References ···································· 75

12. 2D NMR of (+)-Cracroson A, (+)-Teucvisin C, Compound **30** and (+)-Cracroson E ············ 77

13. Copies of NMR Spectra ···································· 91

1. General Methods

Unless stated otherwise, all solvents and reagents were purified and dried according to standard methods prior to use. All reactions were carried out in flame-dried glassware under an atmosphere of argon with magnetic stirring. Reactions were monitored by thin-layer chromatography (TLC) carried out on Huanghai silica gel HSGF254 pre-coated plates (0.2 mm ± 0.03 mm) using UV light as visualizing agent and an ethanolic solution of phosphomolybdic acid, an aqueous solution of cerium sulfate or a basic aqueous solution of potassium permanganate as developing agents. Silica gel (300-400 mesh) purchased from Yantai Xinnuo Chemical Co. LTD was used for flash chromatography. ^{1}H and ^{13}C NMR spectra were recorded on a Bruker AVANCE III HD (400 MHz and 100 MHz, respectively) and internally referenced to residual portion solvent signals ($CDCl_3$, δ_H = 7.26 ppm, δ_C = 77.00 ppm; CD_3CN, δ_H = 1.94 ppm, δ_C = 118.26 ppm; acetone-d_6, δ_H = 2.05 ppm, δ_C = 206.26 ppm or pyridine-d_5, δ_H = 7.20 ppm, δ_C = 123.44 ppm, DMSO-d_6, δ_H = 2.50 ppm, δ_C = 39.52 ppm). The following abbreviations were used to designate multiplicities: s = singlet, d = doublet, t = triplet, q = quartet, m = multiplet, and br = broad. High-resolution mass spectra (HRMS) were recorded on an Agilent Technologies 6224 TOF LC/MS using ESI (electrospray ionization) or an Agilent GCQTOF mass spectrometer using EI (electronic ionization). Optical Rotations were measured on a Rudolph Autopol III S2 polarimeter. ^{13}C NMR spectra of teucrin A (**6**) was recorded on an Agilent DD2 (100 MHz).

2. Synthesis of 1,3-Cyclohexadiene Silane Ether 11

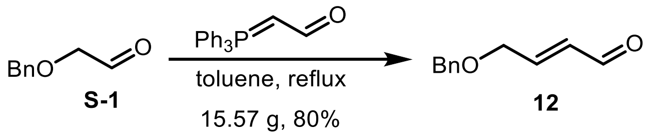

To a solution of **S-1** (16.5 g, 110 mmol) in toluene (500 mL) was added $Ph_3PCHCHO$ (33.5 g, 110 mmol). After stirring at reflux for 1 h, the reaction mixture was concentrated under reduced pressure. The residue was purified by flash column chromatography (petroleum ether: ethyl acetate = 30: 1 to 10: 1) to afford unsaturated aldehyde **12** (15.6 g, 80%) as a colorless oil.

12: **^{1}H NMR** (400 MHz, $CDCl_3$) δ 9.58 (d, *J* = 8.0 Hz, 1H), 7.40–7.29 (m, 6H), 6.85 (dt, *J* = 16.0, 4.0 Hz, 1H), 6.41 (dd, *J* = 16.0, 8.0 Hz, 1H), 4.59 (s, 2H), 4.29 (dd, *J* = 4.0, 1.6 Hz, 2H); **^{13}C NMR** (100 MHz, $CDCl_3$) δ 193.2, 153.0, 137.4, 131.7, 128.5, 127.9, 127.6, 72.9, 68.5. The spectroscopic data were identical with those reported in the literature.[1]

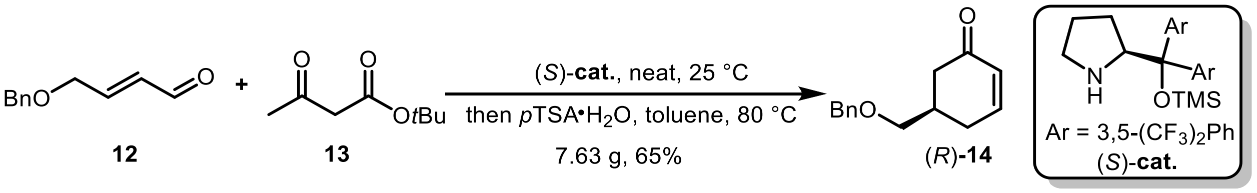

To a mixture of (*S*)-**cat.** (3.25 g, 5.43 mmol) and unsaturated aldehyde **12** (14.36 g, 81.5 mmol) was added *tert*-butyl acetoacetate **13** (8.59 g, 54.3 mmol). The resulting mixture was stirred at 25 °C for 18 h until **13** was consumed completely. Then, toluene (76 mL) and *p*TSA•H_2O (2.07 g, 10.9 mmol) were added. After stirring at 80 °C for 6 h to remove the *tert*-butyl carboxylate group, the reaction mixture was concentrated under reduced pressure. The residue was purified by flash column chromatography (petroleum ether: ethyl acetate = 20: 1 to 5: 1) to afford enone (*R*)-**14**.

(*R*)-**14**: Pale yellow oil (7.63 g, 65%, 93% ee); the ee value was determined by CHIRALPAK AD-H column, hexane/*i*PrOH = 99/1, 1.0 mL/min, λ = 210 nm. t_R(minor) = 20.86 min, t_R(major) = 22.25 min; $[\alpha]_D^{29}$ = −52.23 (*c* = 1.06, $CHCl_3$); **^{1}H NMR** (400 MHz, $CDCl_3$) δ 7.28–7.16 (m, 5H), 6.86 (ddd, *J* = 10.0, 5.2, 2.4 Hz, 1H), 5.98–5.87 (m, 1H), 4.42 (s, 2H), 3.38–3.29 (m, 2H), 2.47–2.28 (m, 3H), 2.27–2.11 (m, 2H); **^{13}C NMR** (100 MHz, $CDCl_3$) δ 199.2, 149.4, 138.1, 129.7, 128.4, 127.6, 127.5, 73.2, 73.1, 41.1, 35.6, 29.0. The spectroscopic data were identical with those reported in the literature.[2,3]

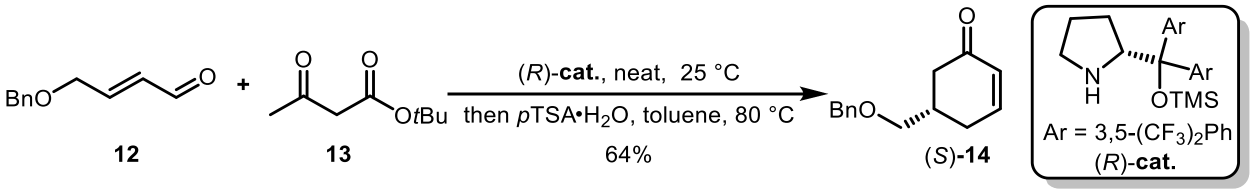

To a mixture of (*R*)-**cat.** (1.20 g, 8.0 mmol) and unsaturated aldehyde **12** (2.11 g, 12.0 mmol) was added *tert*-butyl acetoacetate **13** (1.20 g, 8.0 mmol). The resulting mixture was stirred at 25 °C for 18 h until **13** was completely consumed. Then, toluene (11 mL) and *p*TSA•H_2O (304 mg, 1.6 mmol) were added. After stirring at 80 °C for 6 h to remove the *tert*-butyl carboxylate group, the reaction mixture was concentrated under reduced pressure. The residue was purified by flash column chromatography (petroleum ether: ethyl acetate = 20: 1 to 5: 1) to afford enone (*S*)-**14**.

(*S*)-**14**: Pale yellow oil (1.10 g, 64%, 92% ee); the ee value was determined by CHIRALPAK AD-H column, hexane/*i*PrOH = 99/1, 1.0 mL/min, λ = 210 nm. t_R(major) = 20.44 min, t_R(minor) = 22.06 min; $[\alpha]_D^{20}$ = +51.15 (*c* = 1.03, $CHCl_3$); **^{1}H NMR** (400 MHz, $CDCl_3$) δ 7.38–7.27 (m, 5H), 6.97 (ddd, *J* = 10.0, 4.8, 2.4 Hz, 1H), 6.03 (dd, *J* = 10.0, 1.2 Hz, 1H), 4.52 (s, 2H), 3.48–3.38 (m, 2H), 2.57–2.39 (m, 3H), 2.35–2.22 (m, 2H); **^{13}C NMR** (100 MHz, $CDCl_3$) δ 199.3, 149.4, 138.1, 129.7, 128.4, 127.7, 127.5, 73.2, 73.1, 41.1, 35.6, 29.0. The spectroscopic data were identical with those reported in the literature.[4]

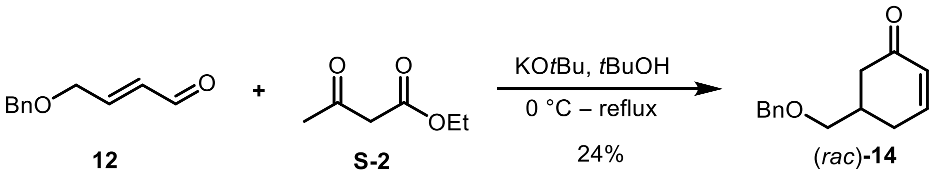

To a stirred solution of **12** (176 mg, 1.0 mmol) and ethyl acetoacetate **S-2** (130 mg, 1.0 mmol) in *t*BuOH (1.0 mL) was added a catalytic amount of KO*t*Bu (5.6 mg, 0.05 mmol) at 0 °C. After stirring at 0 °C for 30 min, to the reaction mixture was added the second batch of KO*t*Bu (22.4 mg, 0.20 mmol), and then heated at 90 °C for 7 h. Upon cooling to room temperature, the reaction mixture was quenched with HCl (1.0 mL, aq., 1.0 M), diluted with a 1: 1 mixture of Et_2O (5.0 mL) and benzene (5.0 mL), washed with NaOH (5.0 mL, aq., 1.0 M) and then brine (5.0 mL). The organic layer was dried over anhydrous Na_2SO_4, and concentrated under reduced pressure. The residue was purified by flash column chromatography (silica gel, petroleum ether: ethyl acetate = 15: 1 to 5: 1) to afford (*rac*)-**14** (52.1 mg, 24% yield) as a pale yellow oil.

(*rac*)-**14: ^{1}H NMR** (400 MHz, $CDCl_3$) δ 7.38–7.27 (m, 5H), 6.97 (ddd, *J* = 10.0, 5.4, 2.4 Hz, 1H), 6.03 (dd, *J* = 10.0, 2.4 Hz, 1H), 4.52 (s, 2H), 3.48–3.39 (m, 2H), 2.57–2.39 (m, 3H), 2.35–2.23 (m,

2H); **^{13}C NMR** (100 MHz, $CDCl_3$) δ 199.3, 149.4, 138.1, 129.7, 128.4, 127.7, 127.5, 73.2, 73.1, 41.1, 35.6, 29.0. The spectroscopic data were identical with those reported in the literature.[5]

(*rac*)-**14**: CHIRALPAK AD-H column, hexane/*i*PrOH, 99:1 v/v, ν = 1.0 mL/min, λ = 210 nm, 25 °C. t_R(minor) = 20.18 min, t_R(major) = 21.72 min.

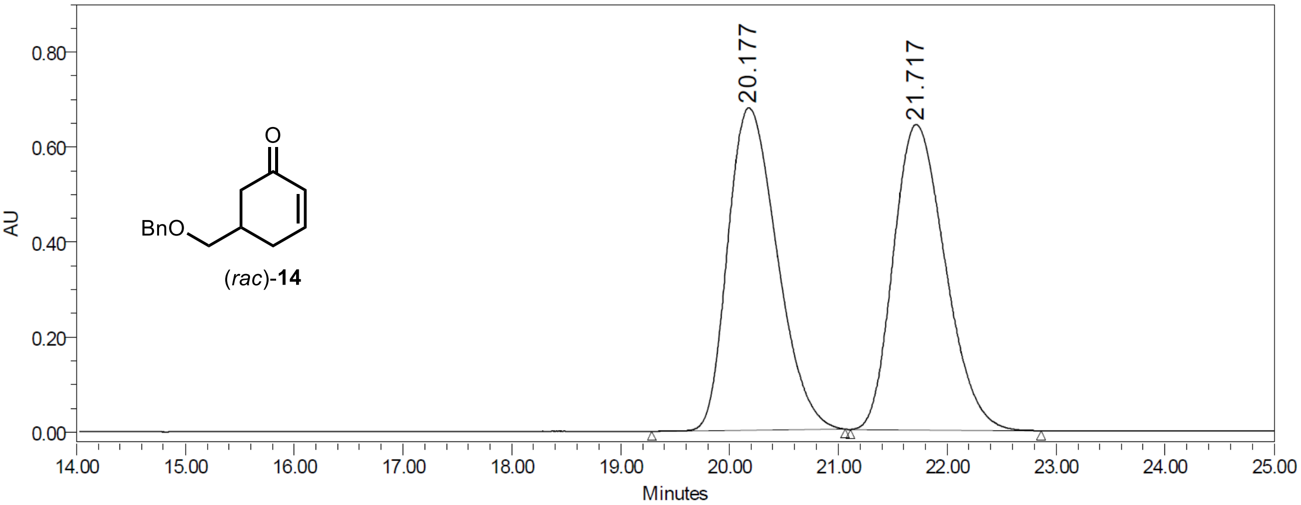

	RT	Area	% Area	Height
1	20.177	21332534	50.10	678194
2	21.717	21251075	49.90	642223

(*R*)-**14**: CHIRALPAK AD-H column, hexane/*i*PrOH, 99:1 v/v, ν = 1.0 mL/min, λ = 210 nm, 25 °C. t_R(minor) = 20.86 min, t_R(major) = 22.25 min.

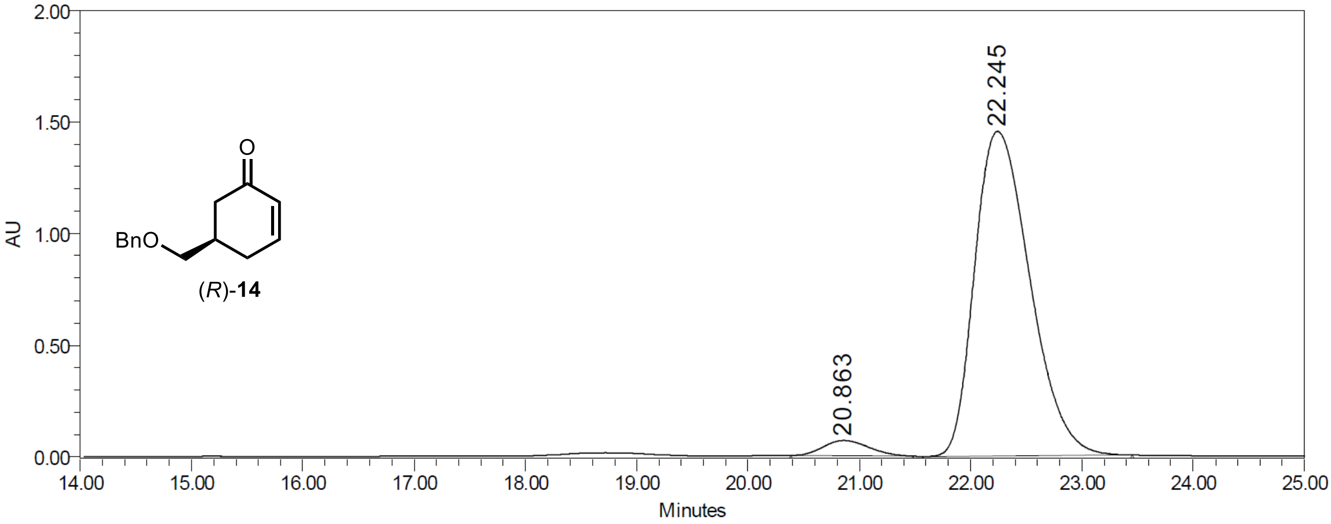

	RT	Area	% Area	Height
1	20.863	1954634	3.75	68148
2	22.245	50104590	96.25	1453990

(S)-**14**: CHIRALPAK AD-H column, hexane/*i*PrOH, 99:1 v/v, ν = 1.0 mL/min, λ = 210 nm, 25 °C. t_R(major) = 20.44 min, t_R(minor) = 22.06 min.

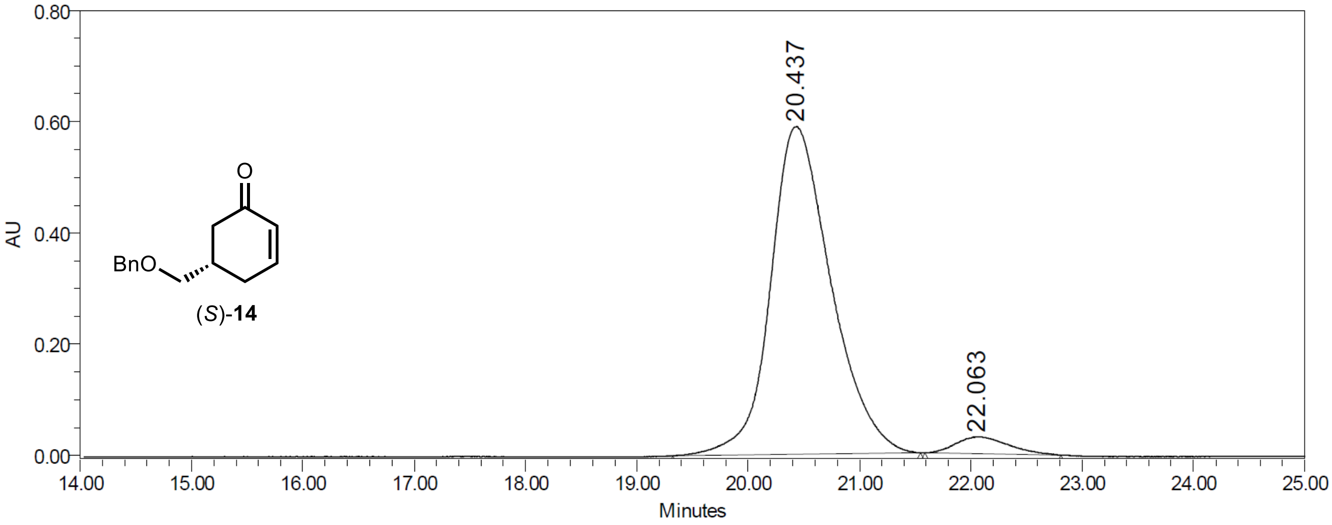

	RT	Area	% Area	Height
1	20.437	22304743	95.76	589467
2	22.063	988245	4.24	30104

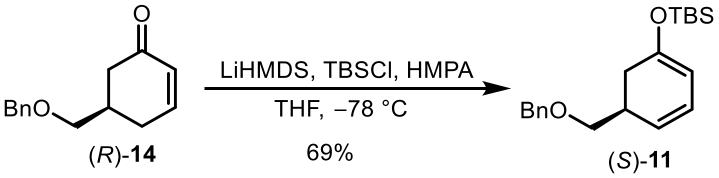

To a stirred solution of cyclohex-2-en-1-one (R)-**14** (93% ee, 8.65 g, 40.0 mmol) and HMPA (17.4 mL, 100 mmol) in THF (160 mL) was added LiHMDS (48 mL, 48.0 mmol, 1.0 M in THF) dropwise at −78 °C. The reaction was stirred at this temperature for 1 h, and then allowed to warm to 0 °C and maintained for another 1 h. After cooling to −78 °C, the reaction mixture was added TBSCl (9.04 g in 80 mL THF, 60.0 mmol) dropwise. After stirring at −78 °C for 3 h, the reaction was quenched with $NaHCO_3$ (200 mL, aq., sat.). The aqueous layer was extracted with petroleum ether (200 mL × 3). The combined organic extracts were washed with brine, dried over Na_2SO_4, and concentrated under reduced pressure. The residue was purified by flash column chromatography (silica gel, 100% petroleum ether) to afford (S)-**11** (9.07 g, 69% yield) as a colorless oil.

(S)-**11**: $[\alpha]_D^{29}$ = –77.57 (c = 1.02, $CHCl_3$); **^{1}H NMR** (400 MHz, $CDCl_3$) δ 7.43–7.27 (m, 5H), 5.89 (ddd, J = 9.2, 5.6, 1.6 Hz, 1H), 5.34 (dd, J = 9.2, 4.0 Hz, 1H), 5.11 (d, J = 5.6 Hz, 1H), 4.55 (s, 2H), 3.49–3.42 (m, 2H), 2.88–2.78 (m, 1H), 2.40–2.27 (m, 2H), 0.97 (s, 9H), 0.21 (s, 3H), 0.20 (s, 3H); **^{13}C NMR** (100 MHz, $CDCl_3$) δ 153.2, 138.5, 128.3, 127.54, 127.46, 125.3, 119.3, 101.6, 73.0, 72.3, 35.8,

31.8, 25.6, 18.0, −4.4, −4.5; **FT-IR** (neat): ν_{max} = 2928, 2857, 1647, 1585, 1472, 1362, 1252, 1219, 1028, 899, 833, 779, 694 cm^{-1}; **HRMS** (EI): exact mass calculated for: $C_{20}H_{30}O_2Si$ $[M]^+$: 330.2010, found: 330.2016.

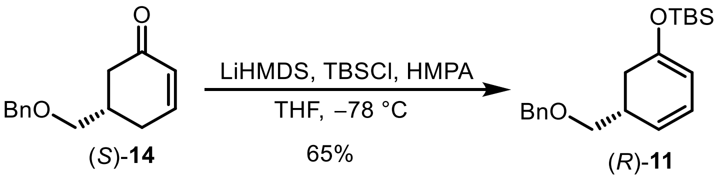

To a stirred solution of cyclohex-2-en-1-one (*S*)-**14** (92% ee, 626 mg, 2.89 mmol) and HMPA (1.26 mL, 7.23 mmol) in THF (12 mL) was added LiHMDS (3.45 mL, 3.45 mmol, 1.0 M in THF) dropwise at −78 °C. The reaction was stirred at this temperature for 1 h, and then allowed to warm to 0 °C and maintained for another 1 h. After cooling to −78 °C, the reaction mixture was added TBSCl (654 mg in 6.0 mL THF, 4.34 mmol) dropwise. After stirring at −78 °C for 3 h, the reaction was quenched with $NaHCO_3$ (20 mL, aq., sat.). The aqueous layer was extracted with petroleum ether (20 mL × 3). The combined organic extracts were washed with brine, dried over Na_2SO_4, and concentrated under reduced pressure. The residue was purified by flash column chromatography (silica gel, 100% petroleum ether) to afford (*R*)-**11** (624 mg, 65% yield) as a colorless oil.

(*R*)-**11**: $[\alpha]_D^{32}$ = +75.51 (*c* = 1.05, $CHCl_3$); **^{1}H NMR** (400 MHz, $CDCl_3$) δ 7.35–7.34 (m, 4H), 7.31–7.27 (m, 1H), 5.86 (ddd, *J* = 9.2, 5.6, 1.6 Hz, 1H), 5.31 (dd, *J* = 9.6, 4.0 Hz, 1H), 5.07 (d, *J* = 5.6 Hz, 1H), 4.52 (s, 2H), 3.46–3.39 (m, 2H), 2.84–2.75 (m, 1H), 2.36–2.23 (m, 2H), 0.94 (s, 9H), 0.17 (s, 3H), 0.16 (s, 3H); **^{13}C NMR** (100 MHz, $CDCl_3$) δ 153.2, 138.5, 128.4, 127.6, 127.5, 125.3, 119.4, 101.7, 73.0, 72.4, 35.8, 31.8, 25.3, 18.1, −4.36, −4.41; **FT-IR** (neat): ν_{max} = 2930, 2857, 1647, 1585, 1361, 1252, 899, 833, 779, 696 cm^{-1}; **HRMS** (EI): exact mass calculated for: $C_{20}H_{30}O_2Si$ $[M]^+$: 330.2010, found: 330.2011.

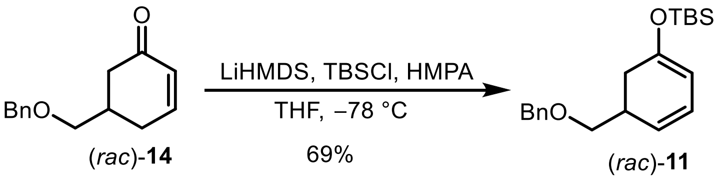

To a stirred solution of cyclohex-2-en-1-one (*rac*)-**14** (387 mg, 1.79 mmol) and HMPA (780 μL, 4.48 mmol) in THF (7.2 mL) was added LiHMDS (2.15 mL, 2.15 mmol, 1.0 M in THF) dropwise at −78 °C. The reaction was stirred at this temperature for 1 h, and then allowed to warm to 0 °C and maintained for another 1 h. After cooling to −78 °C, the reaction mixture was added TBSCl (405 mg in 3.6 mL THF, 2.69 mmol) dropwise. After stirring at −78 °C for 3 h, the reaction was quenched with

$NaHCO_3$ (10 mL, aq., sat.). The aqueous layer was extracted with petroleum ether (10 mL × 3). The combined organic extracts were washed with brine, dried over Na_2SO_4, and concentrated under reduced pressure. The residue was purified by flash column chromatography (silica gel, 100% petroleum ether) to afford (*rac*)-**11** (409 mg, 69% yield) as a colorless oil.

(*rac*)-**11**: **^{1}H NMR** (400 MHz, $CDCl_3$) δ 7.39–7.27 (m, 5H), 5.86 (ddd, *J* = 8.8, 6.0, 1.6 Hz, 1H), 5.31 (dd, *J* = 9.6, 4.0 Hz, 1H), 5.08 (d, *J* = 5.6 Hz, 1H), 4.52 (s, 2H), 3.48–3.38 (m, 2H), 2.87–2.74 (m, 1H), 2.38–2.22 (m, 2H), 0.94 (s, 9H), 0.18 (s, 3H), 0.17 (s, 3H); **^{13}C NMR** (100 MHz, $CDCl_3$) δ 153.2, 138.5, 128.3, 127.6, 127.5, 125.3, 119.3, 101.7, 73.0, 72.4, 35.8, 31.8, 25.6, 18.1, −4.37, −4.42; **FT-IR** (neat): ν_{max} = 2930, 2857, 1647, 1583, 1361, 1250, 899, 833, 779, 694 cm^{-1}; **HRMS** (EI): exact mass calculated for: $C_{20}H_{30}O_2Si$ $[M]^+$: 330.2010, found: 330.2010.

3. Investigation of the Pivotal IEDDA Reaction

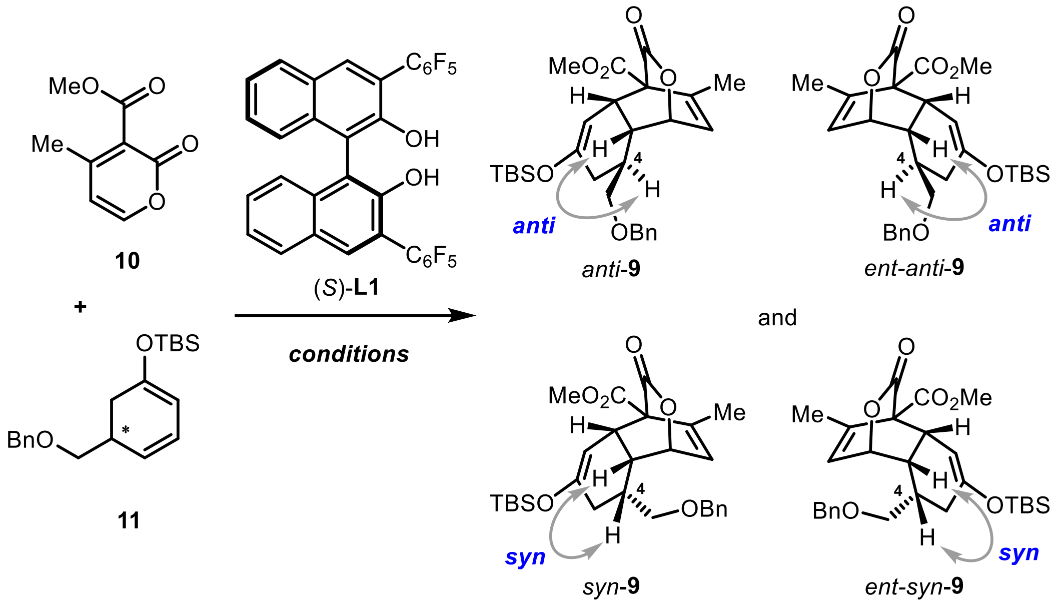

Entry	Conditions [a]	Results	Products
1[b]	(*S*)-**11** (1.5 equiv)<br>Yb(OTf)3	78%, 3.1:1 dr	*anti*-**9**, 94% ee<br>*ent-syn*-**9**, 94% ee
2	(*rac*)-**11** (2.0 equiv)<br>Yb(OTf)3, (*S*)-**L1**, DIPEA	73%, 1.2:1 dr	*anti*-**9**, 78% ee<br>*syn*-**9**, 90% ee
3	(*R*)-**11** (1.5 equiv)<br>Yb(OTf)3, (*S*)-**L1**, DIPEA	75%, 3.8:1 dr	*syn*-**9**, >99% ee<br>*ent-anti*-**9**, 72% ee
4	(*S*)-**11** (1.5 equiv)<br>Yb(OTf)3, (*S*)-**L1**, DIPEA	87%, 10:1 dr<br>(**4.34 g**)	*anti*-**9**, 98% ee<br>*ent-syn*-**9**, 19% ee

[a] Reaction conditions: **10** (0.10 mmol), **11** (0.15 mmol), $Yb(OTf)_3$ (10 mol%), (*S*)-**L1** (12 mol%), DIPEA (24 mol%), 4Å M.S. (25.0 mg), DCM (0.25 mL) at 25 °C. [b] (*S*)-**L1** was not added. DCM = dichloromethane, DIPEA = *N*,*N*-diisopropylethylamine, M.S. = molecular sieves.

(*rac*)-9: To a mixture of anhydrous $Yb(OTf)_3$ (6.2 mg, 0.010 mol) and freshly activated 4 Å molecular sieves (25.0 mg) in DCM (0.25 mL) was added 4-methyl-2-pyrone **10** (16.8 mg, 0.10 mmol). After the reaction mixture was stirred at 0 °C for 15 min, silyl cyclohexadienol ether (*rac*)-**11** (49.6 mg, 0.15 mmol) was added in one portion. The reaction mixture was stirred at 25 °C for 1 h until **10** was consumed completely (monitored by TLC). The crude mixture was purified directly by flash column chromatography (silica gel, petroleum ether: ethyl acetate = 10: 1) to afford lactone (*rac*)-**9** (41.6 mg, 84%, 3.0: 1 dr) as a colorless oil. This compound was seperated by CHIRALPAK IC column, hexane/*i*PrOH, 80: 20 v/v, ν = 1.0 mL/min, λ = 210 nm, 25 °C; t_R(*anti*-**9**) = 24.29 min, t_R(*syn*-**9**) = 29.22 min, t_R(*ent-syn*-**9**) = 33.45 min, t_R(*ent-anti*-**9**) = 39.55 min.

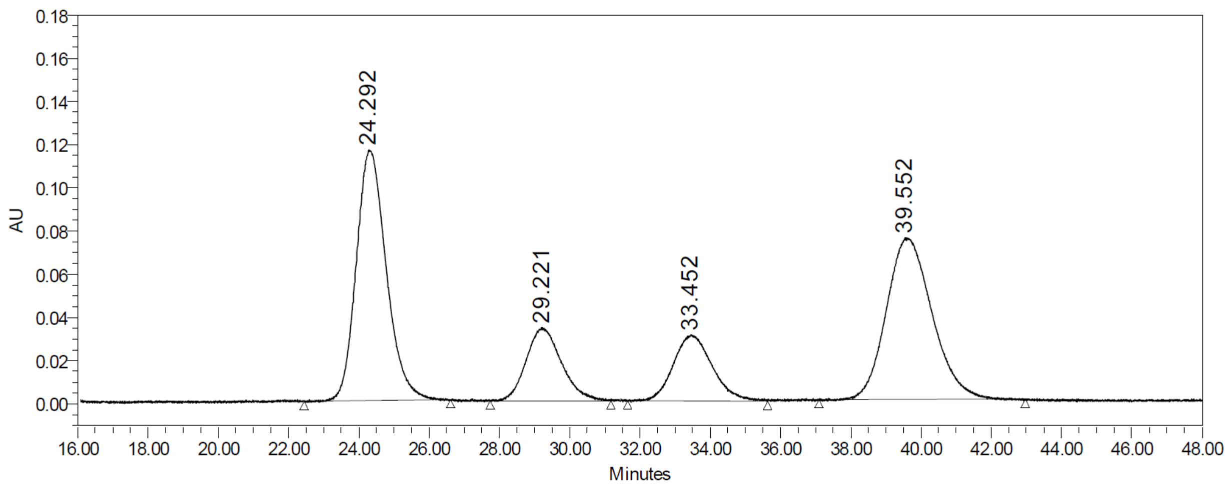

	RT	Area	% Area	Height
1	24.292	6857178	37.53	116055
2	29.221	2329160	12.75	33858
3	33.452	2326957	12.74	30534
4	39.552	6755590	36.98	74811

Entry 1: To a mixture of anhydrous Yb(OTf)$_3$ (6.2 mg, 0.010 mol) and freshly activated 4 Å molecular sieves (25.0 mg) in DCM (0.25 mL) was added 4-methyl-2-pyrone **10** (16.8 mg, 0.10 mmol) at 0 °C, and an orange color appeared immediately. After the mixture was stirred at 0 °C for another 15 min, silyl cyclohexadienol ether (*S*)-**11** (49.6 mg, 0.15 mmol) was added in one portion. The reaction mixture was stirred at 25 °C for 1 h until **10** was consumed completely (monitored by TLC). The crude mixture was purified directly by flash column chromatography (silica gel, petroleum ether: ethyl acetate = 10: 1) to afford *anti*-**9** and *ent-syn*-**9** (38.9 mg, 78%, 3.1: 1 dr, *anti*-**9**: 94% ee; *ent-syn*-**9**: 94% ee) as a colorless mixture. The ee values of *anti*-**9** and *ent-syn*-**9** were determined by CHIRALPAK IC column, hexane/*i*PrOH, 80: 20 v/v, ν = 1.0 mL/min, λ = 210 nm, 25 °C; *anti*-**9**: [t_R(major) = 23.62 min, t_R(minor) = 39.22 min], *ent-syn*-**9**: [t_R(minor) = 28.46 min, t_R(major) = 32.67 min].

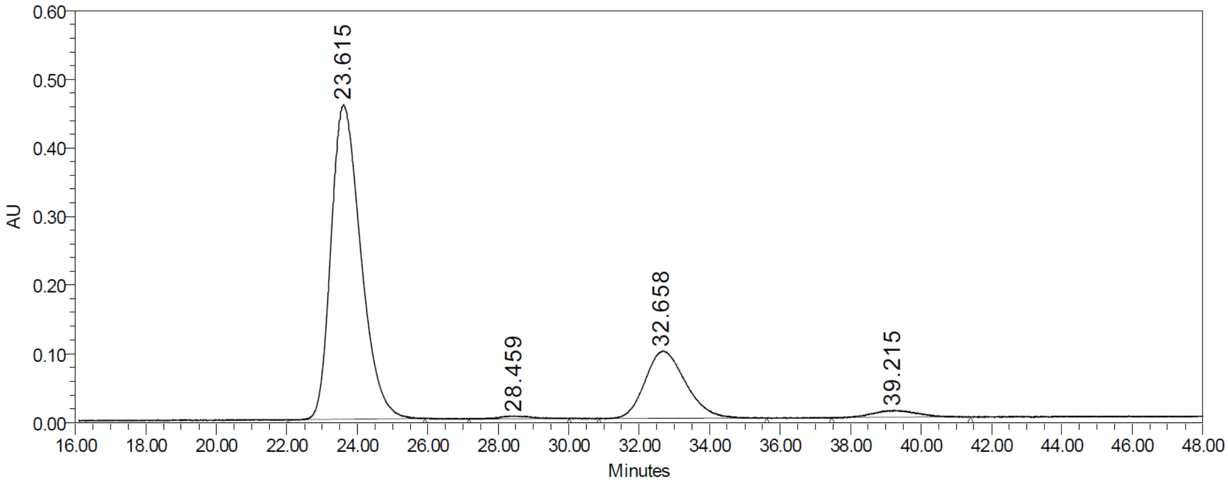

	RT	Area	% Area	Height
1	23.615	26716704	74.95	458614
2	28.459	237168	0.67	4116
3	32.658	7841987	22.00	97876
4	39.215	848114	2.38	10123

Entry 2: To a mixture of anhydrous Yb(OTf)$_3$ (6.2 mg, 0.010 mol), (*S*)-**L1** (7.4 mg, 0.012 mol) and freshly activated 4 Å molecular sieves (25.0 mg) in DCM (0.25 mL) was added DIPEA (4.0 μL, 0.024 mmol) at 0 °C. The mixture was then stirred at this temperature for 30 min to afford a pale-yellow suspension. Then, 4-methyl-2-pyrone **10** (16.8 mg, 0.10 mmol) was added and a dark-blue color appeared immediately. After the mixture was stirred at 0 °C for another 15 min, silyl cyclohexadienol ether (*rac*)-**11** (66.1 mg, 0.20 mmol) was added in one portion. The reaction mixture was stirred at 25 °C for 5 h until **10** was consumed completely (monitored by TLC). The crude mixture was purified directly by flash column chromatography (silica gel, petroleum ether: ethyl acetate = 10: 1) to afford lactone *anti*-**9** and *syn*-**9** (36.4 mg, 73%, 1.2: 1 dr, *anti*-**9**: 78% ee, *syn*-**9**: 90% ee) as a colorless mixture. The ee values of *anti*-**9** and *syn*-**9** were determined by CHIRALPAK IC column, hexane/*i*PrOH, 80: 20 v/v, ν = 1.0 mL/min, λ = 210 nm, 25 °C; *anti*-**9**: [t_R(major) = 23.52 min, t_R(minor) = 37.92 min], *syn*-**9**: [t_R(major) = 28.02 min, t_R(minor) = 32.11 min].

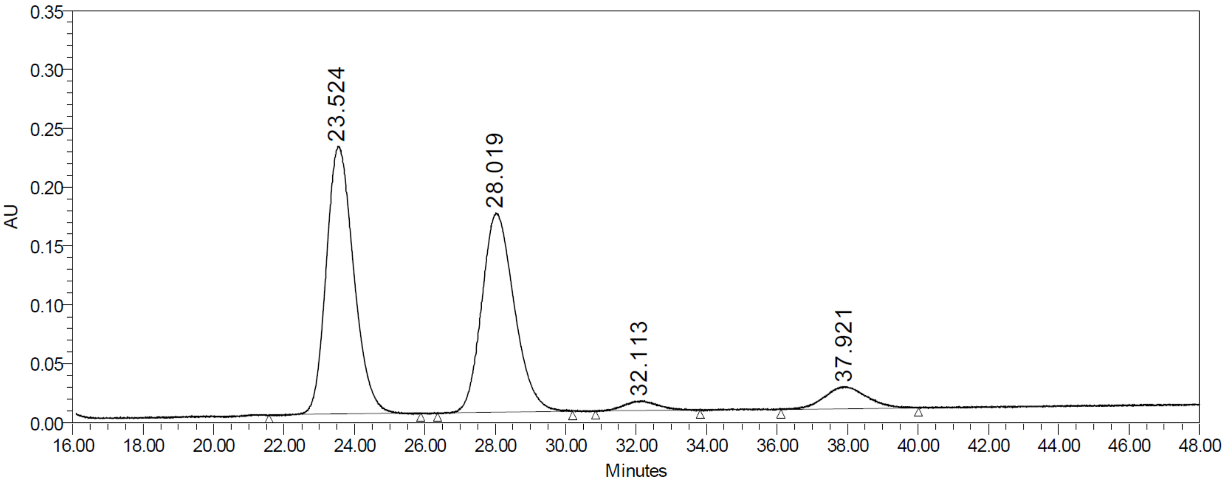

	RT	Area	% Area	Height
1	23.524	12239905	48.59	227656
2	28.019	10860385	43.11	169582
3	32.113	546170	2.17	8501
4	37.921	1545273	6.13	18840

Entry 3: To a mixture of anhydrous $Yb(OTf)_3$ (6.2 mg, 0.010 mol), (*S*)-**L1** (7.4 mg, 0.012 mol) and freshly activated 4 Å molecular sieves (25.0 mg) in DCM (0.25 mL) was added DIPEA (4.0 μL, 0.024 mmol) at 0 °C. The mixture was then stirred at this temperature for 30 min to afford a pale-yellow suspension. Then, 4-methyl-2-pyrone **10** (16.8 mg, 0.10 mmol) was added and a dark-blue color appeared immediately. After the mixture was stirred at 0 °C for another 15 min, silyl cyclohexadienol ether (*R*)-**11** (49.6 mg, 0.15 mmol) was added in one portion. The reaction mixture was stirred at 25 °C for 4 h until **10** was consumed completely (monitored by TLC). The crude mixture was purified directly by flash column chromatography (silica gel, petroleum ether: ethyl acetate = 10: 1) to afford lactone *syn*-**9** and *ent*-*anti*-**9** (37.2 mg, 75%, 3.8: 1 dr, *syn*-**9**: 99% ee, *ent*-*anti*-**9**: 72% ee) as a colorless mixture. The ee values of *syn*-**9** and *ent*-*anti*-**9** were determined by CHIRALPAK IC column, hexane/*i*PrOH, 80: 20 v/v, ν = 1.0 mL/min, λ = 210 nm, 25 °C; *syn*-**9**: [t_R(major) = 29.24 min, t_R(minor) = 33.91 min], *ent*-*anti*-**9**: [t_R(minor) = 24.45 min, t_R(major) = 40.00 min].

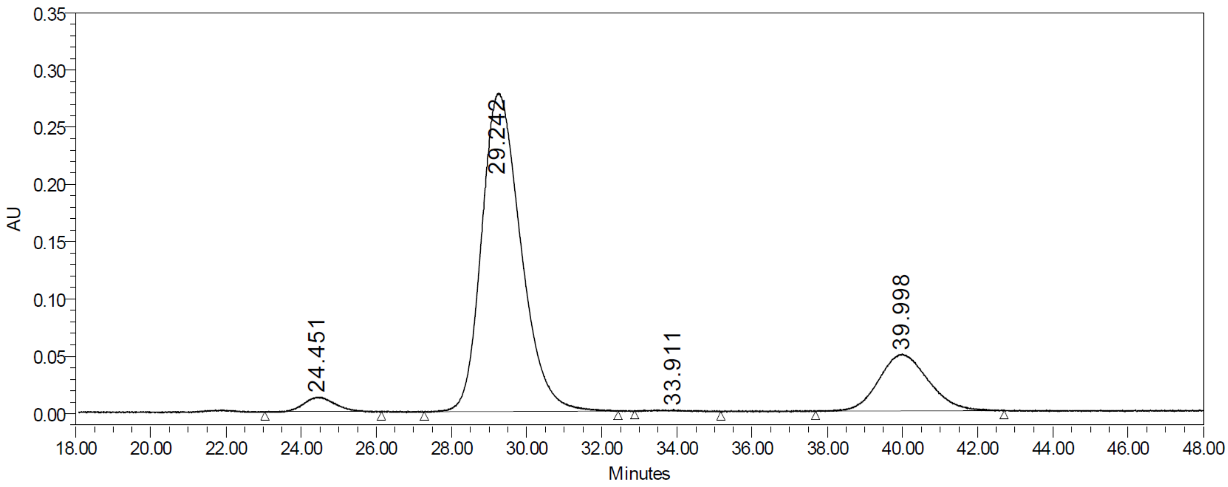

	RT	Area	% Area	Height
1	24.451	743957	2.94	13049
2	29.242	20051682	79.18	277943
3	33.911	44917	0.18	1161
4	39.998	4483845	17.71	49540

Entry 4: To a mixture of anhydrous $Yb(OTf)_3$ (620 mg, 1.0 mmol), (*S*)-**L1** (742 mg, 1.2 mmol) and freshly activated 4 Å molecular sieves (2.50 g) in DCM (25 mL) was added DIPEA (400 µL, 2.3 mmol) at 0 °C. The mixture was then stirred at this temperature for 30 min to afford a pale-yellow suspension. Then, 4-methyl-2-pyrone **10** (1.68 g, 10.0 mmol) was added and a dark-blue color appeared immediately. After the mixture was stirred at 0 °C for another 15 min, silyl cyclohexadienol ether (*S*)-**11** (4.96 g, 15.0 mmol) was added in one portion. The reaction mixture was stirred at 25 °C for 4 h until **10** was consumed completely (monitored by TLC). The reaction mixture was diluted with DCM (25 mL), filtered through a celite pad, and concentrated under reduced pressure. The residue was purified by flash column chromatography (silica gel, petroleum ether: ethyl acetate = 10: 1) to afford lactone *anti*-**9** and *ent*-*syn*-**9** (4.34 g, 87%, 10: 1 dr, *anti*-**9**: 98% ee, *ent*-*syn*-**9**: 19% ee) as a colorless mixture. The ee values of *anti*-**9** and *ent*-*syn*-**9** were determined by CHIRALPAK IC column, hexane/*i*PrOH, 80:20 v/v, ν = 1.0 mL/min, λ = 210 nm, 25 °C; major isomer: [t_R(major) = 24.05 min, t_R(minor) = 39.51 min], minor isomer: [t_R(minor) = 29.07 min, t_R(major) = 33.18 min].

anti-**9**: $[\alpha]_D^{23}$ = –35.70 (*c* = 1.02, $CHCl_3$); **^{1}H NMR** (400 MHz, $CDCl_3$) δ 7.38–7.29 (m, 5H), 6.07–6.05 (m, 1H), 5.11 (dd, *J* = 5.2, 3.2 Hz, 1H), 4.71 (d, *J* = 3.2 Hz, 1H), 4.56–4.48 (m, 2H), 3.90 (s, 3H), 3.46–3.39 (m, 2H), 3.27–3.24 (m, 1H), 2.52 (ddd, *J* = 10.2, 6.4, 2.8 Hz, 1H), 1.98 (d, *J* = 1.6 Hz, 3H), 1.93–1.82 (m, 3H), 0.89 (s, 9H), 0.10 (s, 3H), 0.08 (s, 3H); **^{13}C NMR** (100 MHz, $CDCl_3$) δ 171.0, 168.1, 153.1, 141.4, 138.0, 128.5, 127.8, 127.6, 124.6, 100.7, 76.9, 73.6, 73.3, 62.9, 52.5, 40.9, 36.5,

36.2, 30.5, 25.6, 20.7, 18.0 , −4.35, −4.44; **FT-IR** (neat): ν_{max} = 2957, 2860, 1740, 1668, 1364, 1271, 1207, 1082, 1003, 835, 779, 735, 698 cm^{-1}; **HRMS** (ESI): exact mass calculated for: $C_{28}H_{38}NaO_6Si$ $[M+Na]^+$: 521.2335, found: 521.2352.

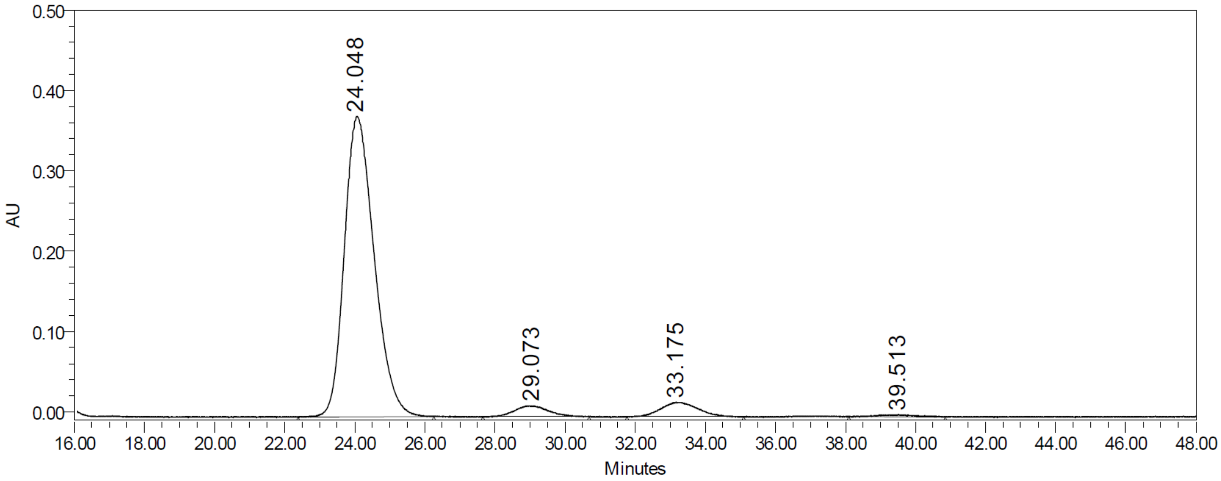

	RT	Area	% Area	Height
1	24.048	21955663	90.26	373356
2	29.073	887467	3.65	13504
3	33.175	1299683	5.34	17987
4	39.513	181508	0.75	2653

4. Total Synthesis of (+)-Teucvin

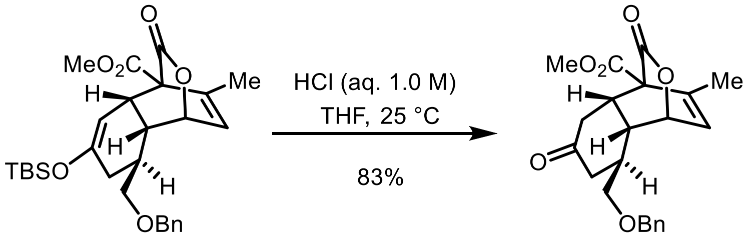

To a solution of *anti*-**9** (4.61g, 9.24 mmol) in THF (92 mL) was added HCl (4.62 mL, 4.62 mmol, aq., 1.0 M) dropwise at 25 °C. The reaction mixture was stirred at this temperature for 4 h. After the reaction was complete, the reaction mixture was diluted with water (200 mL) and extracted with DCM (100 mL × 3). The combined extracts were washed with brine, dried over Na_2SO_4 and concentrated under reduced pressure. The residue was purified by flash column chromatography (silica gel, petroleum ether: ethyl acetate = 1: 1) to afford **15** (2.93 g, 83% yield) as a white solid.

15: the m.p. could not be determined because this compound will undergo retro-Diels-Alder extrusion of CO_2 before melting; $[\alpha]_D^{27}$ = +121.07 (*c* = 1.05, $CHCl_3$); **^{1}H NMR** (400 MHz, $CDCl_3$) δ 7.38–7.28 (m, 5H), 6.25–6.23 (m, 1H), 5.35 (dd, *J* = 5.2, 3.2 Hz, 1H), 4.52 (dd, *J* = 20.0, 12.0 Hz, 2H), 3.86 (s, 3H), 3.51–3.44 (m, 2H), 2.93 (ddd, *J* = 13.6, 9.6, 4.4 Hz, 1H), 2.58–2.52 (m, 2H), 2.35 (dd, *J* = 18.6, 3.6 Hz, 1H), 2.13 (dd, *J* = 18.6, 13.6 Hz, 1H), 2.04 (d, *J* = 1.6 Hz, 3H), 1.91 (dd, *J* = 15.2, 13.6 Hz, 1H), 1.85–1.75 (m, 1H); **^{13}C NMR** (100 MHz, $CDCl_3$) δ 208.8, 169.9, 167.1, 140.8, 137.5, 128.5, 127.9, 127.5, 125.3, 74.7, 73.3, 72.2, 61.7, 52.7, 42.6, 40.9, 38.9, 35.1, 33.3, 20.7; **FT-IR** (neat): ν_{max} = 2963, 1759, 1713, 1443, 1366, 1327, 1125, 1098, 1074, 991, 920, 818, 741, 700 cm^{-1}; **HRMS** (ESI): exact mass calculated for: $C_{22}H_{24}O_6Na$ $[M+Na]^+$: 407.1465, found: 407.1457.

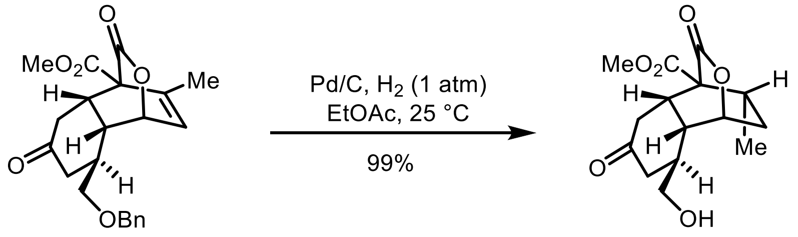

To a solution of **15** (2.33 g, 6.06 mmol) in EtOAc (303 mL) was added Pd/C (1.29 g, 1.21 mmol, 10% w/w in carbon). The mixture was stirred at 25 °C under H_2 (1 atm) for 24 h. The reaction mixture was filtered through a short pad of Celite and concentrated under reduced pressure. The residue was purified by flash column chromatography (silica gel, petroleum ether: ethyl acetate = 1: 2) to afford **16** (1.77 g, 99 % yield) as a white solid.

16: m.p. 164.8-166.0 °C; $[\alpha]_D^{28}$ = +80.16 (*c* = 1.14, $CHCl_3$); **^{1}H NMR** (400 MHz, $CDCl_3$) δ 4.94 (dd, *J* = 5.2, 3.2 Hz, 1H), 3.81 (s, 3H), 3.69–3.63 (h, *J* = 2.0 Hz, 2H), 2.96–2.89 (m, 1H), 2.75–2.57 (m, 3H), 2.54–2.36 (m, 4H), 2.22–2.13 (m, 2H), 1.72 (dd, *J* = 14.4, 8.0 Hz, 1H), 1.28 (d, *J* = 7.2 Hz, 3H); **^{13}C NMR** (100 MHz, $CDCl_3$) δ 209.6, 172.9, 168.6, 76.3, 64.0, 55.5, 52.7, 41.1, 40.0, 39.7, 35.3, 31.5, 30.0, 28.4, 19.3; **FT-IR** (neat): ν_{max} = 3541, 2965, 1734, 1705, 1435, 1332, 1260, 1111, 1057, 991, 893 cm^{-1}; **HRMS** (ESI): exact mass calculated for: $C_{15}H_{20}O_6Na$ $[M+Na]^+$: 319.1152, found: 319.1155.

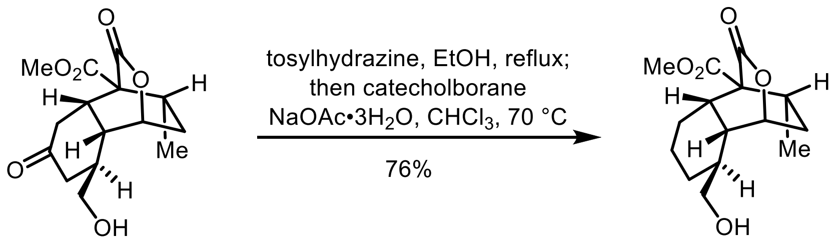

16 **17**

To a suspension of tosylhydrazine (512 mg, 2.75 mmol) in EtOH (12 mL) was added ketone **16** (740 mg, 2.50 mmol) at 25 °C. The resulting mixture was stirred under reflux for 2 h, and then concentrated under reduced pressure to afford the corresponding tosylhydrazone intermediate as a crude mixture. The residue was dissolved in $CHCl_3$ (42 mL) and catecholborane (2.75 mL, 2.75 mmol) was added slowly at 0 °C. The reaction mixture was stirred at this temperature for 2 h. Then NaOAc•3H_2O (680 mg, 5.00 mmol) was added and the resulting mixture was stirred under 70 °C for 50 min. After cooling to 25 °C, the reaction mixture was filtered. The solid was washed with $CHCl_3$ (30 mL). The combined filtrates were concentrated under reduced pressure. The residue was purified by flash column chromatography (silica gel, petroleum ether: ethyl acetate = 1: 1) to afford **17** (539 mg, 76 % yield) as a colorless oil.

17: $[\alpha]_D^{26}$ = −58.41 (*c* = 1.02, $CHCl_3$); **^{1}H NMR** (400 MHz, $CDCl_3$) δ 4.87 (dd, *J* = 5.2, 3.2 Hz, 1H), 3.77 (s, 3H), 3.50 (d, *J* = 4.8 Hz, 2H), 2.61–2.45 (m, 2H), 2.33–2.25 (m, 2H), 2.04–1.99 (m, 1H), 1.81–1.68 (m, 3H), 1.66–1.59 (m, 3H), 1.47–1.37 (m, 1H), 1.23 (d, *J* = 7.2 Hz, 3H); **^{13}C NMR** (100 MHz, $CDCl_3$) δ 174.1, 169.6, 77.3, 65.9, 56.1, 52.2, 41.0, 35.3, 33.8, 30.1, 28.4, 23.2, 22.3, 20.1, 19.2; **FT-IR** (neat): ν_{max} = 3512, 2940, 2882, 1732, 1460, 1381, 1331, 1256, 1209, 1099, 1065, 993, 816, 735 cm^{-1}; **HRMS** (ESI): exact mass calculated for: $C_{15}H_{22}NaO_5$ $[M+Na]^+$: 305.1365, found: 305.1358.

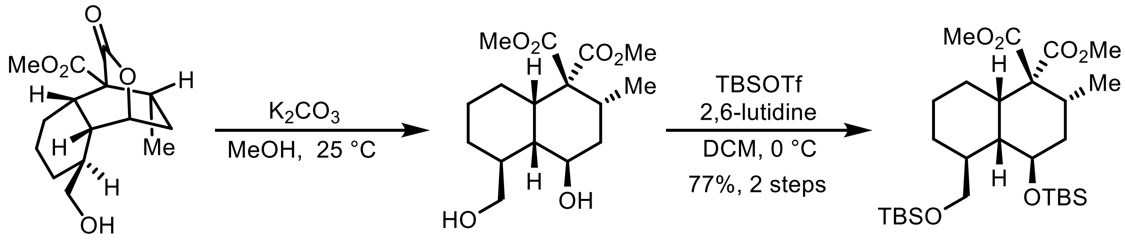

17 **S-3** **18**

To a solution of lactone **17** (1.01 g, 3.59 mmol) in anhydrous MeOH (36 mL) was added K_2CO_3 (1.24 g, 8.99 mmol) at 25 °C. After stirring at this temperature for 6 h, the reaction mixture was neutralized with AcOH (18 mL, aq., 0.5 M). The mixture was extracted with EtOAc (20 mL × 3). The combined extracts were washed with brine, dried over Na_2SO_4 and concentrated under reduced pressure. The crude **S-3** was used directly in the next step without further purification.

To a solution of crude **S-3** in DCM (90 mL) was added 2,6-lutidine (2.51 mL, 21.6 mmol) and TBSOTf (2.48 mL, 10.8 mmol) at 0 °C. The reaction mixture was stirred for 1 h at 0 °C, and then quenched with $NaHCO_3$ (50 mL, aq., sat.). The aqueous layer was extracted with DCM (50 mL × 3). The combined extracts were dried over Na_2SO_4 and concentrated under reduced pressure. The residue was purified by flash column chromatography (silica gel, petroleum ether: ethyl acetate = 50: 1) to afford **18** (1.50 g, 77%) as a colorless oil.

18: $[\alpha]_D^{24}$ = −24.00 (*c* = 1.01, $CHCl_3$); **^{1}H NMR** (400 MHz, $CDCl_3$) δ 4.04 (td, *J* = 10.4, 4.4 Hz, 1H), 3.69 (s, 3H), 3.72–3.68 (m, 1H), 3.66 (s, 3H), 3.55 (dd, *J* = 10.0, 6.4 Hz, 1H), 2.86 (br, 1H), 2.78 (d, *J* = 11.2 Hz, 1H), 2.19 (br, 1H), 1.81–1.77 (m, 2H), 1.67–1.60 (m, 2H), 1.51–1.39 (m, 5H), 1.18 (d, *J* = 7.2 Hz, 3H), 0.861 (s, 9H), 0.855 (s, 9H), 0.044 (s, 3H), 0.037 (s, 3H), 0.01 (s, 6H); **^{13}C NMR** (100 MHz, $CDCl_3$) δ 171.6, 170.5, 64.6, 64.4, 62.3, 52.8, 52.1, 43.2, 39.8, 38.1, 35.1, 32.3, 26.1, 25.9, 25.8, 22.6, 22.1, 18.4, 18.2, 18.0, −4.1, −4.7, −5.29, −5.32; **FT-IR** (neat): ν_{max} = 2928, 2856, 1734, 1472, 1431, 1256, 1219, 1200, 1084, 1040, 831, 772, 667 cm^{-1}; **HRMS** (ESI): exact mass calculated for: $C_{28}H_{54}NaO_6Si_2$ $[M+Na]^+$: 565.3357, found: 565.3350.

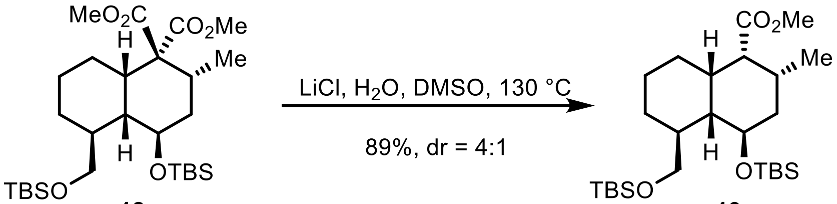

18 **19**

To a solution of **18** (496 mg, 0.91 mmol) in DMSO (91 mL) was added LiCl (388 mg, 9.15 mmol) and H_2O (824 mg, 45.7 mmol) at 25 °C. After stirring at 130 °C for 18 h, the reaction mixture was cooled to 25 °C, and diluted with water (500 mL). The resulting mixture was extracted with Et_2O (200

mL × 3). The combined extracts were washed with water and brine, dried over Na_2SO_4 and concentrated under reduced pressure. The residue was purified by flash column chromatography (silica gel, petroleum ether: ethyl acetate = 50: 1) to afford **19** (394 mg for both isomers, 89% yield, 4: 1 dr) as a colorless oil.

19: $[\alpha]_D^{27}$ = −10.62 (*c* = 1.01, $CHCl_3$); **^{1}H NMR** (400 MHz, $CDCl_3$) δ 4.21–4.14 (m, 1H), 3.69–3.44 (m, 5H), 2.52 (t, *J* = 5.2 Hz, 1H), 2.45–2.28 (m, 2H), 2.22–2.14 (m, 1H), 1.96–1.92 (m, 1H), 1.70–1.59 (m, 1H), 1.53–1.23 (m, 7H), 1.03 (d, *J* = 2.8 Hz, 3H), 0.89–0.87 (m, 18H), 0.05–0.02 (m, 12H); **^{13}C NMR** (100 MHz, $CDCl_3$) δ 176.6, 67.3, 66.7, 51.1, 51.0, 49.9, 49.4, 46.3, 46.2, 37.0, 36.6, 35.1, 32.6, 30.5, 29.5, 28.9, 26.0, 25.9, 21.7, 20.7, 20.5, 18.5, 18.3, 18.0, −4.3, −4.7, −4.475, −4.478, −5.3, −5.4, −5.5; **FT-IR** (neat): ν_{max} = 2928, 2857, 1734, 1472, 1387, 1360, 1252, 1165, 1088, 1053, 1005, 974, 833, 772, 669 cm^{-1}; **HRMS** (ESI): exact mass calculated for: $C_{26}H_{52}NaO_4Si_2$ $[M+Na]^+$: 507.3302, found: 507.3299.

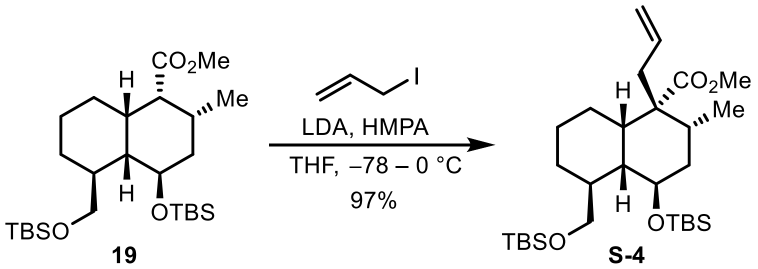

To a solution of **19** (382 mg, 0.79 mmol) in THF (16 mL) was added LDA (3.15 mL, 3.15 mmol, 1.0 M in THF) at –78 °C. After stirring at this temperature for 5 h, the reaction mixture was added HMPA (565 mg, 3.15 mmol) and allyl iodide (265 mg, 1.58 mmol). After stirring at 0 °C for 2 h, the reaction mixture was quenched with NH_4Cl (16 mL, aq., sat.). The aqueous layer was extracted with Et_2O (20 mL × 3). The combined extracts were washed with brine, dried over Na_2SO_4, and concentrated under reduced pressure. The residue was purified by flash column chromatography (silica gel, petroleum ether: ethyl acetate = 50: 1) to afford **S-4** (402 mg, 97% yield) as a colorless oil.

S-4: $[\alpha]_D^{29}$ = –26.29 (*c* = 0.99, $CHCl_3$); **^{1}H NMR** (400 MHz, $CDCl_3$) δ 5.72–5.61 (m, 1H), 5.05–5.01 (m, 2H), 4.16–4.10 (m, 1H), 3.65–3.54 (m, 5H), 2.52 (ddd, *J* = 21.6, 14.4, 7.2 Hz, 2H), 2.23–2.17 (m, 2H), 2.12–2.10 (m, 1H), 1.83–1.75 (m, 2H), 1.71–1.62 (m, 2H), 1.56–1.46 (m, 3H), 1.43–1.33 (m, 2H), 1.12 (d, *J* = 6.8 Hz, 3H), 0.90 (s, 18H), 0.08–0.05 (m, 12H); **^{13}C NMR** (100 MHz, $CDCl_3$) δ 175.7, 134.2, 117.6, 65.6, 65.3, 53.4, 50.8, 41.9, 38.2, 35.2, 32.1, 32.0, 26.9, 26.0, 22.3, 19.1, 18.3, 18.1, −4.1, −4.6, −5.3; **FT-IR** (neat): ν_{max} = 2928, 2860, 1734, 1472, 1252, 1198, 1092, 1005, 837, 773, 743 cm^{-1}; **HRMS** (ESI): exact mass calculated for: $C_{29}H_{56}NaO_4Si_2$ $[M+Na]^+$: 547.3615, found: 547.3609.

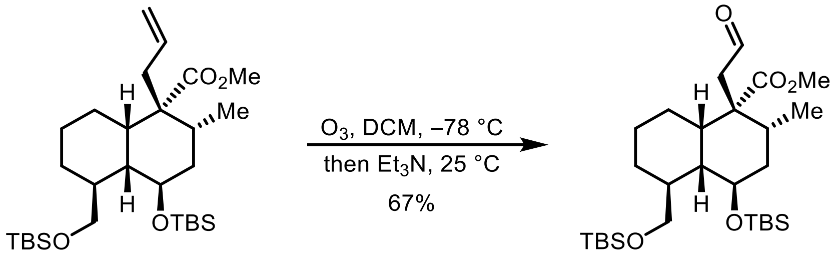

To a solution of **S-4** (468 mg, 0.89 mmol) in DCM (89 mL) was bubbled with ozone at −78 °C until the solution turned blue. After the excess ozone was excluded by the argon stream, Et_3N (247 μL, 1.78 mmol) was added dropwise at −78 °C. The reaction mixture was stirred at 25 °C for 2 h and then concentrated under reduced pressure. The residue was purified by flash column chromatography (silica gel, petroleum ether: ethyl acetate = 20: 1) to afford aldehyde **20** (312 mg, 67% yield) as a colorless oil.

20: $[\alpha]_D^{28}$= −25.52 (*c* = 1.07, $CHCl_3$); **^{1}H NMR** (400 MHz, $CDCl_3$) δ 9.73 (s, 1H), 4.16 (br, 1H), 3.68 (s, 3H), 3.59–3.50 (m, 2H), 2.78 (dd, *J* = 15.6, 3.2 Hz, 1H), 2.70 (dd, *J* = 15.6, 2.4 Hz, 1H), 2.20–2.15 (m, 3H), 1.97 (br, 1H), 1.74–1.48 (m, 5H), 1.37–1.36 (m, 3H), 1.09 (d, *J* = 5.2 Hz, 3H), 0.88 (s, 9H), 0.87 (s, 9H), 0.05–0.03 (m, 12H); **^{13}C NMR** (100 MHz, $CDCl_3$) δ 201.7, 175.1, 65.7, 65.3, 51.5, 51.4, 50.3, 38.4, 37.4, 35.1, 32.6, 26.8, 25.9, 25.8, 21.9, 18.2, 18.0, −4.3, −4.7, −5.35, −5.36; **FT-IR** (neat): ν_{max} = 2934, 2855, 1721, 1472, 1250, 1194, 1084, 1061, 833, 772, 667 cm^{-1}; **HRMS** (ESI): exact mass calculated for: $C_{28}H_{54}O_5NaSi_2$ $[M+Na]^+$: 549.3402, found: 549.3403.

Table S1 1,2-Addition of 3-Furanyl Metal Reagent to Aldehyde **20**

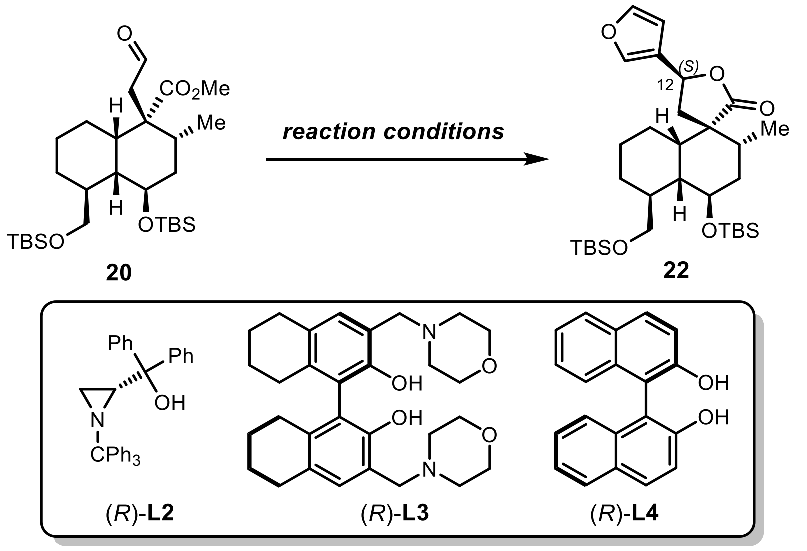

Entry	Conditions [a]	Yield [b]	dr [c]
1	3-bromofuran (3.0 equiv.), *n*BuLi (3.0 equiv.), THF, −78 °C	72%	1.5:1.0
2	3-bromofuran (3.0 equiv.), Mg (3.0 equiv.), THF, 25 °C; then **20**, 0 °C	68%	1.8:1.0
3	3-bromofuran (3.0 equiv.), *n*BuLi (3.0 equiv.), CuI (1.5 equiv.), THF, −78 °C; then **20**, 0 °C	59%	2.0:1.0
4	3-furanboronic acid (6.0 equiv.), $ZnEt_2$ (18.0 equiv.), toluene 60 °C; then (*R*)-**L2** (20 mol%), **20**, 0 °C - 25 °C	41%	13:1.0
5	3-furanboronic acid (6.0 equiv.), $ZnEt_2$ (18.0 equiv.), toluene 60 °C; then (*S*)-**L2** (20 mol%), **20**, 0 °C - 25 °C	46%	1.0:8.1
6	3-furanboronic acid (6.0 equiv.), $ZnEt_2$ (18.0 equiv.), toluene 60 °C; then (*R*)-**L3** (20 mol%), **20**, 0 °C - 25 °C	55%	1.9:1.0
7	(3-furyl)Ti(O*i*Pr)$_3$ (2.0 equiv.), toluene, 0 °C	85%	5.3:1.0
8	(3-furyl)Ti(O*i*Pr)$_3$ (2.0 equiv.), (*R*)-**L4** (20 mol%), toluene, 0 °C	90%	15:1.0
9	(3-furyl)Ti(O*i*Pr)$_3$ (2.0 equiv.), (*S*)-**L4** (20 mol%), toluene, 0 °C	80%	2.2:1.0

[a] reaction conditions: **20** (0.019 mmol), 3-furyl nucleophile (x equiv.), Ligand (20 mol%), solvent. [b] Isolated yield. [c] The dr value was determined by crude ^{1}H NMR.

Entry 8: To a solution of (3-furyl)Ti(O*i*Pr)$_3$[6] (11.1 mg, 0.038 mmol) and (*R*)-**L3** (1.0 mg, 20 mol%) in toluene (0.38 mL)was added aldehyde **20** (10.0 mg in 0.10 mL toluene, 0.019 mmol) dropwise at 0 °C. After stirring at this temperature for 2 h, the reaction mixture was quenched with NaOH (1.0 mL, aq., 1.0 M). The aqueous layer was extracted with EtOAc (2.0 mL × 3). The combined extracts were washed with brine, dried over Na_2SO_4, and concentrated under reduced pressure. The crude was purified by flash column chromatography (silica gel, petroleum ether: ethyl acetate = 30: 1) to afford **22** (9.6 mg, 90%, 15: 1 dr) as a white foam.

22: m.p. 79.6-80.2 °C; $[\alpha]_D^{25}$= +11.80 (*c* = 0.57, $CHCl_3$); **^{1}H NMR** (400 MHz, $CDCl_3$) δ 7.45 (s, 1H), 7.424–7.420 (m, 1H), 6.41–6.40 (m, 1H), 5.41 (t, *J* = 8.4 Hz, 1H), 4.30 (br, 1H), 3.59 (dd, *J* = 10.4, 4.4 Hz, 1H), 3.48 (dd, *J* = 9.2, 4.4 Hz, 1H), 2.48–2.43 (m, 1H), 2.35–2.30 (m, 3H), 2.22–2.16 (m, 1H), 2.11–1.95 (m, 2H), 1.63–1.50 (m, 5H), 1.38 (d, *J* = 13.6 Hz, 1H), 1.21–1.16 (m, 1H), 1.02 (br, 3H), 0.89 (s, 9H), 0.87 (s, 9H), 0.05–0.04 (m, 12H); **^{13}C NMR** (100 MHz, $CDCl_3$) δ 179.5, 143.9, 139.5, 125.5, 108.2, 71.6, 66.9, 66.3, 50.3, 44.4, 40.0, 34.7, 33.2, 29.7, 28.1, 26.0, 25.8, 21.1, 18.3, 18.0, 16.4, −4.66, −4.70, −5.36, −5.40; **FT-IR** (neat): ν_{max} = 2951, 2855, 1761, 1472, 1252, 1155, 1084, 833, 772 cm^{-1}; **HRMS** (ESI): exact mass calculated for: $C_{31}H_{54}O_5NaSi_2$ $[M+Na]^+$: 585.3402, found: 585.3394.

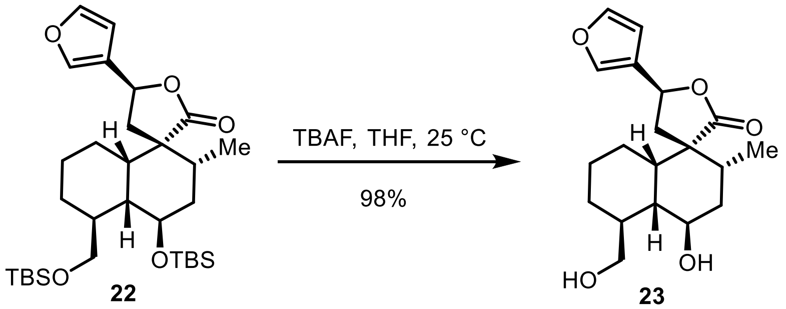

To a solution of **22** (128 mg, 0.23 mmol) in THF (9.1 ml) was added TBAF (0.68 mL, 0.68 mmol, 1.0 M in THF) dropwise at 25 °C. After stirring at this temperature for 12 h, the reaction mixture was quenched with NH_4Cl (10 mL, aq., sat.). The aqueous layer was extracted with EtOAc (10 mL × 3). The combined extracts were washed with brine, dried over $MgSO_4$ and concentrated under reduced pressure. The residue was purified by flash column chromatography (silica gel, dichloromethane: methanol = 30: 1) to afford diol **23** (74.6 mg, 98% yield) as a white foam.

23: m.p. 72.0-73.5 °C; $[\alpha]_D^{24}$ = +2.04 (*c* = 1.07, $CHCl_3$); **^{1}H NMR** (400 MHz, $CDCl_3$) δ 7.47 (s, 1H), 7.43 (s, 1H), 6.41 (s, 1H), 5.41 (dd, *J* = 5.2, 3.6 Hz, 1H), 4.28 (d, *J* = 3.6 Hz, 1H), 3.70 (dd, *J* = 11.2, 4.8 Hz, 1H), 3.56 (dd, *J* = 11.2, 5.2 Hz, 1H), 2.49 (dd, *J* = 13.6, 7.6 Hz, 1H), 2.36–2.21 (m, 5H), 2.09–2.02 (m, 3H), 1.93–1.88 (m, 1H), 1.77 (dt, *J* = 9.2, 4.4 Hz, 1H), 1.69–1.64 (m, 1H), 1.61–1.49

(m, 3H), 1.38–1.30 (m, 1H), 1.07 (d, J = 6.8 Hz, 3H); **^{13}C NMR** (100 MHz, $CDCl_3$) δ 179.1, 144.0, 139.7, 125.0, 108.2, 71.6, 66.0, 65.3, 50.4, 44.1, 44.0, 39.4, 35.0, 34.7, 33.2, 27.9, 27.8, 21.2, 16.4; **FT-IR** (neat): ν_{max} = 3424, 2924, 1751, 1323, 1155, 1022, 874, 731 cm^{-1}; **HRMS** (ESI): exact mass calculated for: $C_{19}H_{26}O_5Na$ [M+Na]$^+$: 357.1673, found: 357.1668.

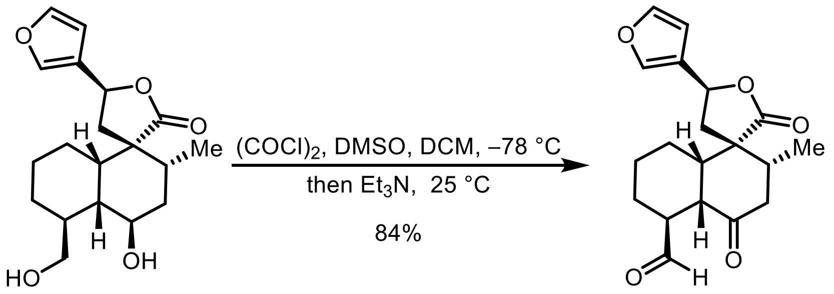

23 **S-5**

To a stirred solution of DCM (1.7 mL) and oxalyl chloride (34.7 µL, 0.41 mmol) was added DMSO (58.3 µL, 0.82 mmol) dropwise at −78 °C. After stirring at this temperature for 10 min, the reaction mixture was added **23** (56.9 mg, 0.17 mmol) slowly and stirred at −78 °C for another 30 min. Then Et_3N (284 µL, 2.04 mmol) was added and the resulting mixture was stirred at −78 °C for 5 min. After warming to 25 °C slowly and stirring at this temperature for 2 h, the reaction mixture was quenched with $NaHCO_3$ (5.0 mL, aq., sat.). The aqueous layer was extracted with DCM (5.0 mL × 3). The combined extracts were washed with brine, dried over Na_2SO_4, and concentrated under reduced pressure. The residue was purified by flash column chromatography (silica gel, petroleum ether: ethyl acetate = 4: 1) to afford ketoaldehyde **S-5** (47.2 mg, 84% yield) as a white foam.

S-5: m.p. 59.0-60.5 °C; $[\alpha]_D^{24}$ = −195.65 (c = 0.96, $CHCl_3$); **^{1}H NMR** (400 MHz, $CDCl_3$) δ 9.69 (s, 1H), 7.50 (s, 1H), 7.44 (t, J = 1.6 Hz, 1H), 6.42 (d, J = 1.6 1H), 5.38 (dd, J = 10.8, 5.6 Hz, 1H), 3.33 (d, J = 4.4 Hz, 2H), 2.84 (dd, J = 18.4, 13.2 Hz, 1H), 2.56 (dd, J = 12.8, 5.6 Hz, 1H), 2.41–2.31 (m, 2H), 2.25–2.16 (m, 2H), 2.07 (d, J = 13.6 Hz, 1H), 1.77–1.64 (m, 2H), 1.60–1.56 (m, 1H), 1.31–1.20 (m, 1H), 1.14 (d, J = 6.8 Hz, 3H), 1.08 (dt, J = 12.8, 3.6 Hz, 1H); **^{13}C NMR** (100 MHz, $CDCl_3$) δ 210.7, 204.6, 175.8, 144.0, 140.0, 123.4, 108.2, 71.1, 51.0, 46.1, 44.5, 43.9, 42.8, 40.3, 32.5, 30.2, 23.7, 21.2, 15.5; **FT-IR** (neat): ν_{max} = 2955, 2919, 1758, 1704, 1504, 1457, 1146, 1021, 871, 751, 725 cm^{-1}; **HRMS** (ESI): exact mass calculated for: $C_{19}H_{22}O_5Na$ [M+Na]$^+$: 353.1359, found: 353.1356.

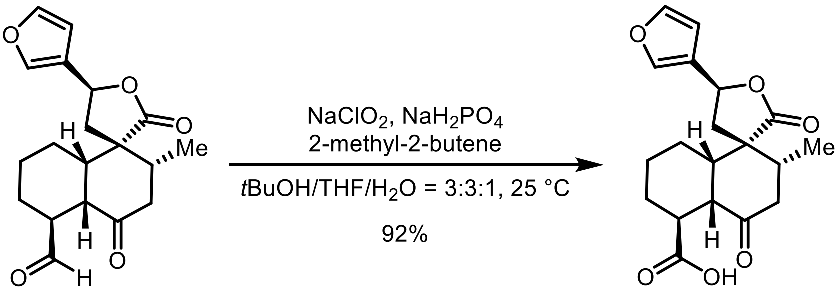

S-5 **24**

To a solution of ketoaldehyde **S-5** (20.4 mg, 0.062 mmol) and NaH_2PO_4 (26.6 mg, 0.222 mmol) in *t*BuOH/THF/H_2O (v:v:v = 3:3:1, 2.1 mL) was added 2-methyl-2-butene (98 μL, 1.167 mmol) and $NaClO_2$ (26.8 mg, 0.296 mmol) sequentially at 25 °C. After stirring at this temperature for 1 h, the reaction mixture was diluted with NH_4Cl (2.0 mL, aq., sat.). The mixture was extracted with EtOAc (2.0 mL × 3). The combined extracts were washed with brine, dried over Na_2SO_4 and concentrated under reduced pressure. The residue was purified by flash column chromatography (silica gel, dichloromethane: methanol = 30: 1) to afford carboxylic acid **24** (19.6 mg, 92% yield) as a white solid.

24: m.p. 141.1-142.3 °C; $[\alpha]_D^{21}$ = −80.21 (*c* = 0.19, acetone); **^{1}H NMR** (400 MHz, acetone-d$_6$) δ 7.76 (s, 1H), 7.62 (s, 1H), 6.62 (s, 1H), 5.65 (dd, *J* = 10.0, 6.0 Hz, 1H), 3.42–3.35 (m, 2H), 2.71 (dd, *J* = 18.0, 13.2 Hz, 1H), 2.56–2.47 (m, 3H), 2.44–2.36 (m, 1H), 2.29 (dd, *J* = 18.0, 3.8 Hz, 1H), 1.98 (d, *J* = 12.8 Hz, 1H), 1.64–1.57 (m, 2H), 1.50–1.38 (m, 2H), 1.29–1.21 (m, 1H), 1.13 (d, *J* = 6.8 Hz, 3H); **^{13}C NMR** (100 MHz, acetone-d$_6$) δ 210.5, 176.7, 175.8, 145.1, 141.9, 125.0 109.6, 71.5, 52.0, 47.7, 45.0, 43.5, 41.3, 38.9 33.2, 31.2, 24.0, 23.9, 15.9; **FT-IR** (neat): ν_{max} = 2939, 1763, 1712, 1676, 1203, 1164, 1145, 1009, 939, 876, 600 cm^{-1}; **HRMS** (ESI): exact mass calculated for: $C_{19}H_{22}O_6Na$ $[M+Na]^+$: 369.1309, found: 369.1300.

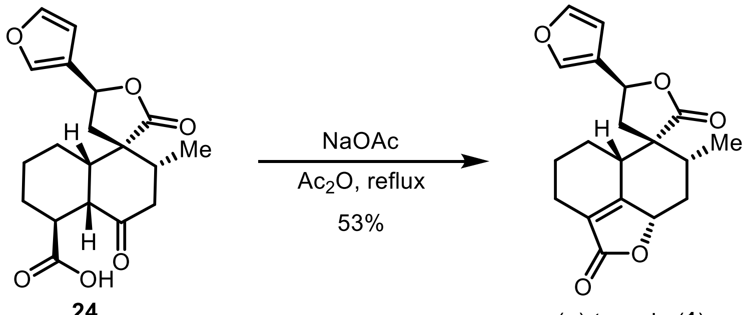

24 (+)-teucvin (**1**)

To a solution of **24** (16.0 mg, 0.046 mmol) in Ac_2O (0.35 mL) was added NaOAc (1.9 mg, 0.023 mmol) at 25 °C. The reaction mixture was stirred at 150 °C for 15 h. After cooling to 25 °C, the mixture was diluted with EtOAc (5.0 mL). The organic layer was washed with $NaHCO_3$ (5.0 mL, aq., sat.), dried over Na_2SO_4, and concentrated under reduced pressure. The residue was purified by column

chromatography (silica gel, petroleum ether: ethyl acetate = 1: 1) to afford (+)-teucvin (**1**) (8.0 mg, 53%) as a white solid.

(+)-Teucvin (**1**): m.p. 199.5-201.4 °C; $[\alpha]_D^{23}$ = +177.46 (*c* = 1.05, $CHCl_3$); **^{1}H NMR** (400 MHz, $CDCl_3$) δ 7.45 (s, 1H), 7.44 (s, 1H), 6.38 (s, 1H), 5.44 (t, *J* = 8.4 Hz, 1H), 4.76 (t, *J* = 8.8 Hz, 1H), 2.69 (br, 1H), 2.55 (d, *J* = 8.8 Hz, 2H), 2.28–2.08 (m, 5H), 2.01–1.98 (m, 1H), 1.93–1.86 (m, 1H), 1.58–1.39 (m, 2H), 1.05 (d, *J* = 6.8 Hz, 3H); **^{13}C NMR** (100 MHz, $CDCl_3$) δ 175.5, 172.9, 161.3, 144.3 , 139.5, 126.6, 124.9, 107.9, 78.2, 71.7, 53.5, 42.1, 40.9, 36.0, 35.2, 24.8, 21.7, 19.6, 17.0; **FT-IR** (neat): ν_{max} = 2934, 1744, 1694, 1350, 1207, 1020, 966, 874, 741 cm^{-1}; **HRMS** (EI): exact mass calculated for: $C_{19}H_{20}O_5$ $[M]^+$: 328.1305, found: 328.1303. The spectroscopic data were identical with those reported in the literature.[7]

5. Total Synthesis of (+)-Cracroson A and (+)-Cracroson E

Table S2 The Allylic Oxidation of (+)-Teucvin

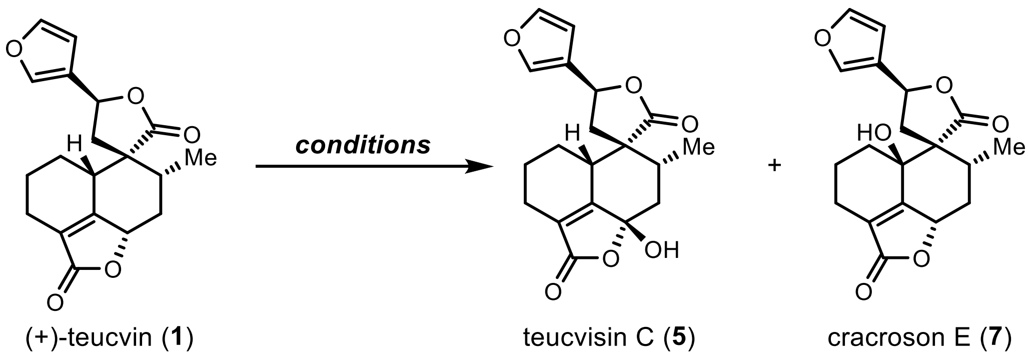

Entry	Conditions [a]	Result
1	SeO_2 (4.0 equiv.), DMSO, 120 °C	**7**, trace
2	SeO_2 (10.0 equiv.), 1,4-dioxane/ formic acid, 80 °C	decomposed
3	SeO_2 (5.0 equiv.), benzene, reflux	**5**, 4%; **7**, 9%
4	SeO_2 (0.5 equiv.), TBHP (2.5 equiv.), DCM, 25 °C	N.R. [b]
5	CrO_3 (1.0 equiv.), TBHP (10 equiv.), pyridine (2.0 equiv.), DCM, 40 °C	**7**, trace

[a] Reaction conditions: teucvin (**1**) (0.01 mmol), oxidant, solvent. [b] N.R. = No reaction. TBHP, *tert*-butyl hydroperoxide.

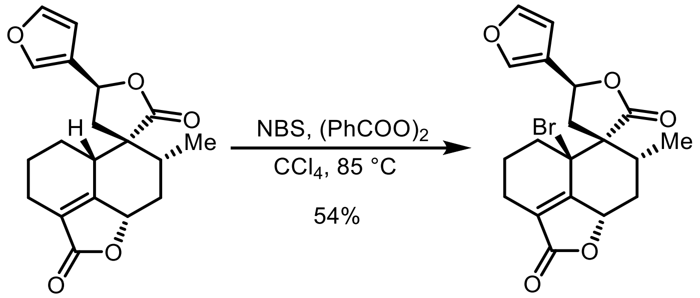

To a mixture of (+)-teucvin (**1**) (24.3 mg, 0.074 mmol) and NBS (15.8 mg, 0.089 mmol) in CCl_4 (7.4 mL) was added dibenzoylperoxide [1.8 mg in 300 μL CCl_4, 10 mol%, (degassed by freeze-pump-thaw cycles for three times)]. After stirring at 85 °C for 20 h, the reaction mixture was concentrated under reduced pressure. The residue was purified by flash column chromatography (silica gel, petroleum ether: ethyl acetate = 3: 1) to afford bromide **25** (16.3 mg, 54%) as a white solid.

25: m.p. 174.5-175.5 °C; $[\alpha]_D^{24}$ = +61.06 (*c* = 0.40, $CHCl_3$); **^{1}H NMR** (400 MHz, $CDCl_3$) δ 7.47 (d, *J* = 4.2 Hz, 2H), 6.40 (s, 1H), 5.50 (t, *J* = 8.4 Hz, 1H), 5.21–5.15 (m, 1H), 3.32 (dd, *J* = 14.8, 8.8 Hz, 1H), 2.62–2.58 (m, 1H), 2.54–2.45 (m, 2H), 2.43–2.35 (m, 1H), 2.26–2.17 (m, 3H), 2.05–1.93 (m, 3H),

1.03 (d, J = 6.8 Hz, 3H); **^{13}C NMR** (100 MHz, $CDCl_3$) δ 172.5, 171.9, 160.0, 144.3, 139.5, 126.2, 124.6, 107.9, 76.4, 71.6, 66.4, 59.9, 39.6, 36.3, 34.8, 34.6, 19.6, 19.3, 17.3. **FT-IR** (neat): ν_{max} = 2928, 2859, 1755, 1462, 1159, 1045, 874, 791, 745, 602 cm^{-1}; **HRMS** (ESI): exact mass calculated for: $C_{19}H_{19}O_5NaBr$ $[M+Na]^+$: 429.0308, found: 429.0304.

Table 2. Investigations of the Hydrolysis Reaction of Allylic Bromide **25**. [a]

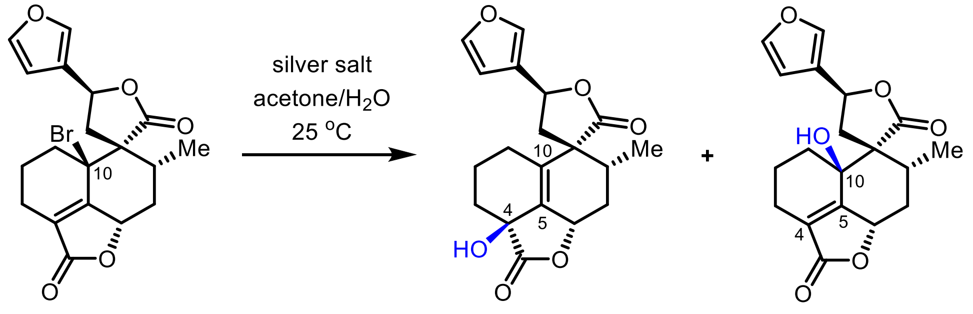

25   cracroson A (**4**)   cracroson E (**7**)

Entry	silver salt	cracroson A [b]	cracroson E [b]	ratio
1	AgOTf	66%	28%	2.4:1
2	$AgNTf_2$	49%	33%	1.5:1
3	$AgSbF_6$	47%	15%	3.1:1
4	$AgBF_4$	76%	21%	3.6:1
5	$AgClO_4$	70%	17%	4.1:1
6	$AgNO_3$	23%	54%	1:2.3
7	Ag_2CO_3	trace	trace	--

[a] Reaction conditions: **25** (0.01 mmol), silver salt (0.03 mmol), acetone/H_2O (v/v = 9:1, 0.7 mL) at 25 °C. [b] Isolated yield.

Entry 4: To a solution of bromide **25** (4.2 mg, 0.01 mmol) in acetone/H_2O (v/v = 9:1, 0.7 mL) was added $AgBF_4$ (6.0 mg, 0.03 mmol) at 25 °C. After stirring at this temperature for 3 h, the reaction mixture was diluted with EtOAc (2.0 mL). The resulting mixture was washed with H_2O (2.0 mL) and brine (2.0 mL), dried over Na_2SO_4 and concentrated under reduced pressure. The residue was purified by column chromatography (silica gel, dichloromethane: diethyl ether = 2: 1) giving (+)-cracroson A (**4**) (2.7 mg, 76%) and (+)-cracroson E (**7**) (0.8 mg, 21%) as a white solid.

Entry 6: To a solution of bromide **25** (4.1 mg, 0.01 mmol) in acetone/H_2O (v/v = 9:1, 0.7 mL) was added $AgNO_3$ (5.3 mg, 0.03 mmol) at 25 °C. After stirring at this temperature for 24 h, the reaction mixture was diluted with EtOAc (2.0 mL). The resulting mixture was washed with H_2O (2.0 mL) and brine (2.0 mL), dried over Na_2SO_4 and concentrated under reduced pressure. The residue was purified

by column chromatography (silica gel, dichloromethane: diethyl ether = 2: 1) giving (+)-cracroson A (**4**) (0.8 mg, 23%) and (+)-cracroson E (**7**) (1.9 mg, 54%) as a white solid.

(+)-Cracroson A (**4**): m.p. 231.6-233.0 °C; $[\alpha]_D^{28}$ = +25.35 (*c* = 0.43, MeOH); **^{1}H NMR** (400 MHz, $CDCl_3$) δ 7.50 (s, 1H), 7.47 (s, 1H), 6.42 (s, 1H), 5.50 (t, *J* = 8.8 Hz, 1H), 5.14 (t, *J* = 8. 0 Hz, 1H), 2.74 (dd, *J* = 14.0, 8.4 Hz, 1H), 2.59 (br s, 1H), 2.41–2.37 (m, 1H), 2.30 (dd, *J* = 14.0, 9.2 Hz, 1H), 2.19–1.95 (m, 6H), 1.87–1.78 (m, 1H), 1.53–1.46 (m, 1H), 1.14 (d, *J* = 7.2 Hz, 3H); **^{13}C NMR** (100 MHz, $CDCl_3$) δ 176.0, 175.9, 144.3, 139.7, 137.4, 132.8, 124.4, 108.0, 76.9, 72.5, 69.1, 52.5, 39.5, 34.9, 32.5, 29.8, 24.9 17.2, 16.1; **FT-IR** (neat): ν_{max} = 3472, 2943, 1755, 1335, 1169, 1146, 974, 947, 874, 741, 604 cm^{-1}; **HRMS** (ESI): exact mass calculated for: $C_{19}H_{20}O_6Na$ $[M+Na]^+$: 367.1152, found: 367.1147. The spectroscopic data were identical with those reported in the literature.[11]

(+)-Cracroson E (**7**): m.p. 187.6-189.1 °C; $[\alpha]_D^{23}$ = +95.79 (*c* = 0.20, MeOH); **^{1}H NMR** (400 MHz, $CDCl_3$) δ 7.46–7.45 (m, 2H), 6.40 (s, 1H), 5.44 (t, *J* = 8.4 Hz, 1H), 5.09–5.03 (m, 1H), 3.23 (dd, *J* = 14.8, 8.8 Hz, 1H), 2.59 (br, 1H), 2.35–2.29 (m, 1H), 2.27–2.17 (m, 4H), 2.14–2.03 (m, 2H), 1.98–1.93 (m, 1H), 1.76–1.62 (m, 2H), 1.02 (d, *J* = 6.0 Hz, 3H); **^{13}C NMR** (100 MHz, $CDCl_3$) δ 175.5, 172.8, 160.9, 144.2, 139.5, 128.6, 125.0, 108.0, 77.1, 72.2, 69.4, 58.9, 35.3, 34.8, 33.8, 32.5, 20.0, 19.4, 17.2; **FT-IR** (neat): ν_{max} = 3397, 2926, 1746, 1215, 1175, 1018, 951, 802, 658 cm^{-1}; **HRMS** (ESI): exact mass calculated for: $C_{19}H_{20}O_6Na$ $[M+Na]^+$: 367.1152, found: 367.1147. The spectroscopic data were identical with those reported in the literature.[15]

6. Total Synthesis of (+)-Montanin A and (+)-Teucvisin C

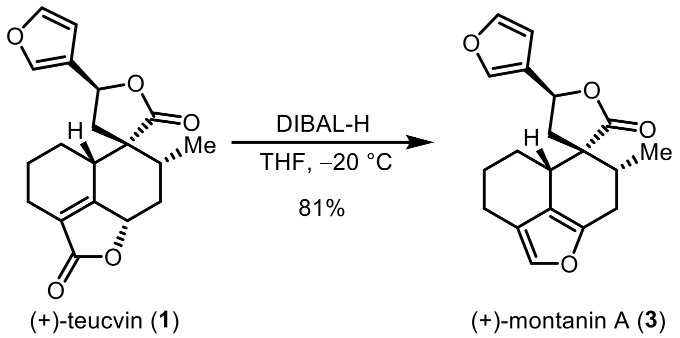

To a solution of (+)-teucvin (**1**) (5.7 mg, 0.017 mmol) in THF (0.43 mL) was added DIBAL-H (52 μL, 0.052 mmol, 1.0 M in hexane) dropwise at −20 °C. After stirring at this temperature for 30 min, the reaction was quenched with HCl (50 μL, aq., 2.0 M) slowly to adjust the pH value to 1~2. Then the resulting mixture was allowed to warm to 25 °C and extracted with EtOAc (2.0 mL × 3). The combined extracts were washed with brine, dried over Na_2SO_4, and concentrated under reduced pressure. The residue was purified by flash chromatography column (silica gel, petroleum ether: ethyl acetate = 4: 1) to afford (+)-montanin A (**3**) (4.4 mg, 81%) as a white solid.

(+)-Montanin A (**3**): m.p. 126.1-127.7 °C; $[\alpha]_D^{16}$ = +103.26 (*c* = 0.31, $CHCl_3$); **^{1}H NMR** (400 MHz, $CDCl_3$) δ 7.47 (s, 1H), 7.44 (t, *J* = 2.0 Hz, 1H), 7.06 (s, 1H), 6.41 (br, 1H), 5.44 (t, *J* = 8.4 Hz, 1H), 2.88–2.75 (m, 3H), 2.69 (dd, *J* = 16.4, 6.0 Hz, 1H), 2.62 (dd, *J* = 13.6, 8.8 Hz, 1H), 2.52–2.39 (m, 2H), 2.31–2.21 (m, 1H), 2.15–2.05 (m, 2H), 1.71–1.58 (m, 1H), 1.30–1.24 (m, 1H), 1.15 (d, *J* = 6.8 Hz, 3H); **^{13}C NMR** (100 MHz, $CDCl_3$) δ 175.5, 147.9, 144.1, 139.6, 136.2, 125.5, 119.7, 116.9, 108.1, 71.6, 50.7, 43.2, 39.7, 36.1, 30.0, 25.6, 23.9, 19.1, 17.8; **FT-IR** (neat): ν_{max} = 2963, 1748, 1175, 1152, 1013, 874, 806, 727, 600 cm^{-1}; **HRMS** (ESI): exact mass calculated for: $C_{19}H_{20}O_4Na$ $[M+Na]^+$: 335.1254, found: 335.1252. The spectroscopic data were identical with those reported in the literature.[9,10]

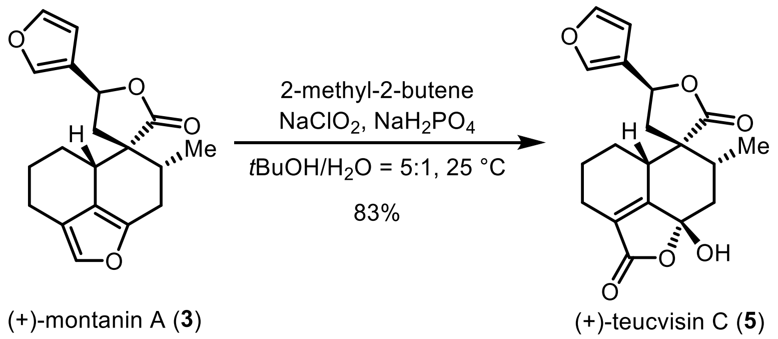

To a solution of (+)-montanin A (**3**) (9.6 mg, 0.031 mmol) in *t*BuOH/H_2O (v:v = 5:1, 1.0 mL) was added NaH_2PO_4 (7.4 mg, 0.061 mmol), 2-methyl-2-butene (25.8 μL, 0.307 mmol), and $NaClO_2$ (8.9

mg, 0.098 mmol) sequentially at 25 °C. After stirring at this temperature for 2 h, the reaction mixture was diluted with H_2O (3.0 mL). The resulting mixture was extracted with EtOAc (3.0 mL × 3). The combined extracts were washed with brine, dried over Na_2SO_4, and concentrated under reduced pressure. The residue was purified by column chromatography (silica gel, petroleum ether: ethyl acetate = 1: 2) to afford (+)-teucvisin C (**5**) (8.8 mg, 83%) as a white solid.

(+)-Teucvisin C (**5**): m.p. 212.3-213.5 °C; $[\alpha]_D^{23}$ = +176.92 (*c* = 0.10, MeOH); **^{1}H NMR** (400 MHz, $CDCl_3$) δ 7.46 (s, 1H), 7.45 (t, *J* = 1.6 Hz, 1H), 6.40 (s, 1H), 5.45 (t, *J* = 8.4 Hz, 1H), 3.71 (br s, 1H), 2.87–2.82 (m, 1H), 2.59–2.53 (m, 2H), 2.47 (t, *J* = 12.4 Hz, 1H), 2.27–2.09 (m, 5H), 2.01–1.96 (m, 1H), 1.60–1.41 (m, 2H), 1.04 (d, *J* = 6.4 Hz, 3H); **^{13}C NMR** (100 MHz, $CDCl_3$) δ 175.8, 170.8, 159.8, 144.3, 139.6, 128.4, 124.8, 108.0, 102.2, 72.0, 54.0, 40.6, 40.2, 39.8, 35.8, 24.5, 21.4, 19.4, 16.6; **FT-IR** (neat): ν_{max} = 3360, 2949, 1757, 1717, 1236, 1155, 1090, 907, 847, 737 cm^{-1}; **HRMS** (ESI): exact mass calculated for: $C_{19}H_{20}O_6Na$ $[M+Na]^+$: 367.1152, found: 367.1143. The spectroscopic data were identical with those reported in the literature.[12]

7. Total Synthesis of (+)-Teucrin A

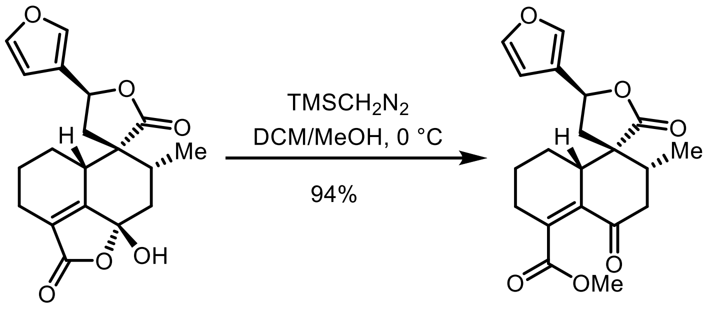

To a solution of (+)-teucvisin C (**6**) (30.5 mg, 0.089 mmol) in DCM (11 mL) and MeOH (3.7 mL) was added $TMSCH_2N_2$ (0.71 mL, 1.42 mmol, 2.0 M in hexane) at 0 °C. After stirring at this temperature for 2 h, the reaction mixture was quenched with AcOH (0.1 mL) until no bubbles emerged. The resulting mixture was washed with H_2O and brine, dried over Na_2SO_4, and concentrated under reduced pressure. The residue was purified by flash chromatography column (silica gel, petroleum ether: ethyl acetate = 1: 1) to afford **29** (29.8 mg, 94%) as a white solid.

29: m.p. 155.5-156.8 °C; $[\alpha]_D^{26}$ = +184.09 (*c* = 0.69, $CHCl_3$); **^{1}H NMR** (400 MHz, $CDCl_3$) δ 7.48 (s, 1H), 7.45 (t, *J* = 1.6 Hz, 1H), 6.41–6.40 (m, 1H), 5.46 (t, *J* = 8.4 Hz, 1H), 3.72 (s, 3H), 3.13 (dd, *J* = 16.4, 12.8 Hz, 1H), 2.68–2.63 (m, 1H), 2.59–2.45 (m, 4H), 2.31–2.21 (m, 2H), 2.19–2.09 (m, 1H), 2.02–1.96 (m, 1H), 1.62–1.51 (m, 1H), 1.46–1.40 (m, 1H), 1.10 (d, *J* = 6.8 Hz, 3H); **^{13}C NMR** (100 MHz, $CDCl_3$) δ 199.9, 175.9, 170.3, 144.3, 139.6, 138.8, 137.3, 124.9, 108.0, 72.0, 52.4, 52.2 , 46.0, 44.3, 39.4, 37.0, 27.1, 25.1, 21.0, 17.8; **FT-IR** (neat): ν_{max} = 2947, 1746, 1721, 1697, 1223, 1074, 1018,

943, 874, 808, 601 cm^{-1}; **HRMS** (ESI): exact mass calculated for: $C_{20}H_{22}O_6Na$ $[M+Na]^+$: 381.1039, found: 381.1309.

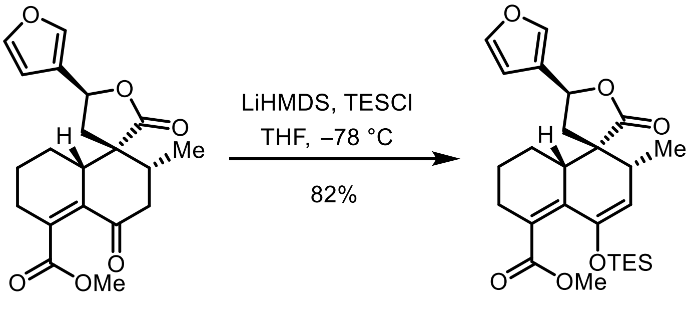

29 **S-6**

To a solution of **29** (21.1 mg, 0.059 mmol) in THF (1.2 mL) was added LiHMDS (68 μL, 0.088 mmol, 1.3 M in THF) dropwise at –78 °C. After stirring at this temperature for 50 min, the reaction mixture was added TESCl (11.8 μL, 0.071 mmol) and stirred for another 30 min. The reaction mixture was quenched with $NaHCO_3$ (1.0 mL, aq., sat.). The resulting mixture was extracted with EtOAc (5.0 mL × 3). The combined extracts were washed with brine, dried over Na_2SO_4, and concentrated under reduced pressure. The residue was purified by flash chromatography column (silica gel, petroleum ether: ethyl acetate = 4: 1) to afford enol ether **S-6** (22.8 mg, 82%) as a colorless oil.

S-6: $[\alpha]_D^{26}$ = +89.01 (*c* = 0.89, $CHCl_3$); ^{1}**H NMR** (400 MHz, $CDCl_3$) δ 7.47 (s, 1H), 7.44 (s, 1H), 6.41 (s, 1H), 5.34 (t, *J* = 8.4 Hz, 1H), 4.78 (d, *J* = 2.0 Hz, 1H), 3.73 (s, 3H), 2.60–2.51 (m, 3H), 2.43 (dd, *J* = 18.0, 4.4 Hz, 1H), 2.35–2.25 (m, 2H), 2.24–2.17 (m, 1H), 1.99–1.95 (m, 1H), 1.57–1.46 (m, 1H), 1.38–1.28 (m, 1H), 1.12 (d, *J* = 7.2 Hz, 3H), 0.97 (t, *J* = 8.0 Hz, 9H), 0.74–0.67 (m, 6H); ^{13}**C NMR** (100 MHz, $CDCl_3$) δ 174.6, 172.7, 146.5, 144.1, 139.6, 129.9, 129.0, 125.3, 110.1, 108.1, 71.2, 52.0, 51.9, 43.8, 40.7, 38.0, 28.6, 25.6, 21.6, 16.7, 6.7, 4.6; **FT-IR** (neat): ν_{max} = 2953, 2876, 1765, 1719, 1229, 1163, 1142, 1016, 806, 725 cm^{-1}; **HRMS** (ESI): exact mass calculated for: $C_{26}H_{36}O_6NaSi$ $[M+Na]^+$: 495.2173, found: 495.2177.

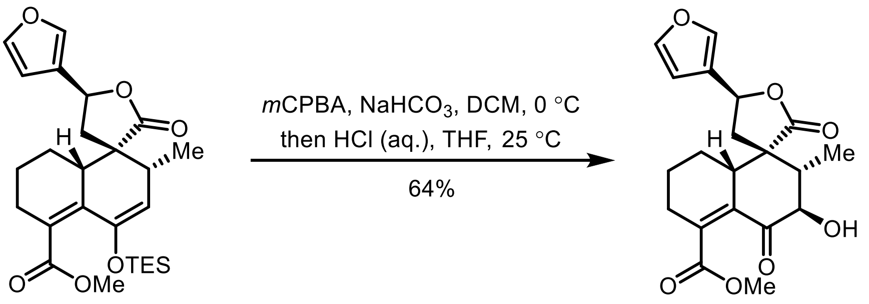

S-6 **30**

To a solution of **S-6** (11.3 mg, 0.024 mmol) in DCM (0.96 mL) was added $NaHCO_3$ (3.0 mg, 0.036 mmol) and *m*CPBA (7.3 mg, 0.036 mmol, 85% w/w) at 0 °C. After stirring at this temperature for 1 h, the reaction mixture was quenched with Na_2SO_3 (1.0 mL, aq., sat.). The resulting mixture was extracted with EtOAc (2.0 mL × 3). The combined extracts were washed with brine, dried over Na_2SO_4, and concentrated under reduced pressure. The residue was used in the next step without further purification.

To a solution of the above residue in THF (0.96 mL) was added HCl (190 μL, aq., 1.0 M) at 25 °C. After stirring at this temperature for 1 h, the reaction mixture was quenched with $NaHCO_3$ (1.0 mL, aq., sat.). The resulting mixture was extracted with EtOAc (2.0 mL× 3). The combined extracts were washed with brine, dried over Na_2SO_4, and concentrated under reduced pressure. The residue was purified by flash chromatography column (silica gel, petroleum ether: ethyl acetate = 1: 1) to afford **30** (5.8 mg, 64%) as a white solid.

30: m.p. 170.3-171.8 °C; $[\alpha]_D^{27}$ = +193.40 (*c* = 0.37, $CHCl_3$); **^{1}H NMR** (400 MHz, $CDCl_3$) δ 7.49 (s, 1H), 7.47 (t, *J* = 1.6 Hz, 1H), 6.43 (s, 1H), 5.48 (t, *J* = 8.4 Hz, 1H), 4.89 (dd, *J* = 11.2, 3.6 Hz, 1H), 3.68 (s, 3H), 3.56 (d, *J* = 3.6 Hz, 1H), 2.72–2.60 (m, 2H), 2.60–2.49 (m, 2H), 2.25–2.15 (m, 2H), 2.00–1.96 (m, 1H), 1.89–1.81 (m, 1H), 1.57–1.42 (m, 2H), 1.30 (d, *J* = 6.4 Hz, 3H); **^{13}C NMR** (100 MHz, $CDCl_3$) δ 203.6, 176.1, 168.0, 144.4, 140.3, 139.7, 136.8, 124.7, 107.9, 75.7, 72.6, 54.0, 52.4, 47.0, 45.9, 39.6, 26.1, 25.0, 20.7, 14.4; **FT-IR** (neat): ν_{max} = 3470, 2920, 1738, 1697, 1279, 1254, 1163, 1018, 991, 872, 793 cm^{-1}; **HRMS** (ESI): exact mass calculated for: $C_{20}H_{22}O_7Na$ $[M+Na]^+$: 397.1258, found: 397.1257.

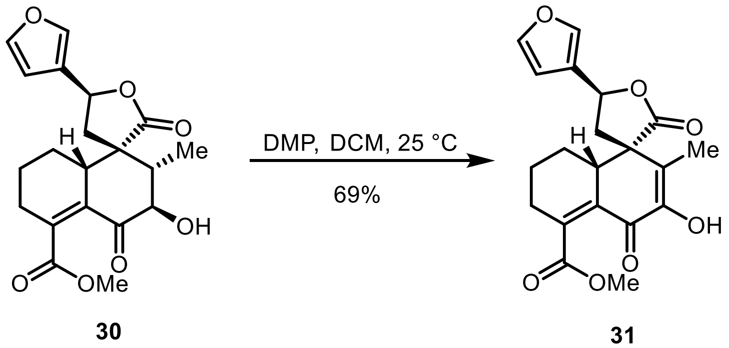

30 31

To a solution of **30** (14.8 mg, 0.04 mmol) in DCM (0.8 mL) was added Dess–Martin periodinane (25.1 mg, 0.06 mmol) at 25 °C. After stirring at this temperature for 1 h, the reaction mixture was quenched with $Na_2S_2O_3$ (0.5 mL, aq., sat.) and $NaHCO_3$ (0.5 mL, aq., sat.). The resulting mixture was extracted with EtOAc (2.0 mL × 3). The combined extracts were washed with brine, dried over Na_2SO_4, and concentrated under reduced pressure. The residue was purified by flash chromatography column (silica gel, petroleum ether: ethyl acetate = 1: 1) to afford **31** (10.1 mg, 69%) as a white solid.

31: m.p. 64.4-65.2 °C; $[\alpha]_D^{32}$ = –50.27 (c = 0.37, $CHCl_3$); **^{1}H NMR** (400 MHz, $CDCl_3$) δ 7.52 (s, 1H), 7.49 (t, J = 1.2 Hz, 1H), 6.55 (s, 1H), 6.44 (s, 1H), 5.53 (t, J = 8.4 Hz, 1H), 3.81 (s, 3H), 2.95–2.85 (m, 2H), 2.61 (dd, J = 14.4, 8.4 Hz, 1H), 2.57–2.51 (m, 1H), 2.42–2.32 (m, 1H), 2.27–2.24 (m, 1H), 2.07–2.02 (m, 1H), 1.91 (s, 3H), 1.67–1.45 (m, 3H); **^{13}C NMR** (100 MHz, $CDCl_3$) δ 180.1, 174.8, 171.0, 146.8, 144.5, 143.5, 139.8, 128.3, 126.9, 124.8, 107.9, 72.5, 54.3, 52.6, 44.1, 38.8, 28.0, 24.8, 20.6, 14.5; **FT-IR** (neat): ν_{max} = 3393, 2951, 1761, 1724, 1653, 1614, 1281, 1253, 1159, 873, 729 cm^{-1}; **HRMS** (ESI): exact mass calculated for: $C_{20}H_{20}O_7Na$ $[M+Na]^+$: 395.1101, found: 395.1105.

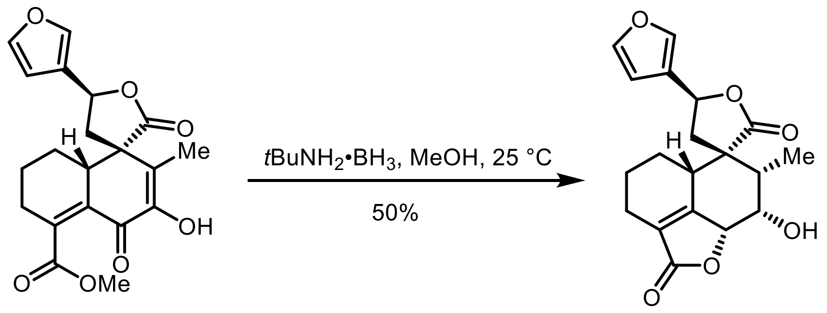

31 (+)-teucrin A (**6**)

To a solution of **31** (3.8 mg, 0.010 mmol) in MeOH (0.2 mL) was added $tBuNH_2{\bullet}BH_3$ (2.6 mg, 0.031 mmol) at 25 °C. After stirring at this temperature for 10 h, the reaction mixture was quenched with HCl (20 μL, aq., 2.0 M). The resulting mixture was extracted with EtOAc (1.0 mL × 3). The combined extracts were washed with brine, dried over Na_2SO_4, and concentrated under reduced pressure. The residue was purified by flash column chromatography (silica gel, dichloromethane: acetone = 5: 1) to afford (+)-teucrin A (**6**) (1.7 mg, 50%) as a white solid.

(+)-Teucrin A (**6**): m.p. 222.0-222.9 °C; $[\alpha]_D^{32}$ = +91.03 (c = 0.11, $CHCl_3$); **^{1}H NMR** (400 MHz, DMSO-d_6) δ 7.90 (s, 1H), 7.75 (s, 1H), 6.59 (d, J = 0.8 Hz, 1H), 5.78 (t, J = 8.4 Hz, 1H), 5.01–5.00 (m, 1H), 4.54 (d, J = 10.8 Hz, 1H), 4.02 (ddd, J = 10.8, 4.4, 2.4 Hz, 1H), 3.09–3.05 (m, 1H), 2.73 (dd, J = 14.0, 8.0 Hz, 1H), 2.63 (dd, J = 14.0, 9.6 Hz, 1H), 2.37–2.30 (m, 1H), 2.24–2.16 (m, 1H), 2.12–2.08 (m, 1H), 2.02–1.86 (m, 2H), 1.56–1.45 (m, 1H), 1.31–1.27 (m, 1H), 1.12 (d, J = 7.8 Hz, 3H); **^{13}C NMR** (100 MHz, DMSO-d_6) δ 180.5, 172.6, 159.3, 144.7, 141.3, 126.6, 123.8, 108.7, 80.2, 74.4, 71.4, 56.2, 41.0, 40.9, 37.5, 24.1, 21.2, 19.2, 13.4; **^{1}H NMR** (400 MHz, $CDCl_3$) δ 7.50 (s, 1H), 7.48 (s, 1H), 6.41 (s, 1H), 5.60 (t, J = 8.4 Hz, 1H), 4.77 (d, J = 2.4 Hz, 1H), 4.65 (d, J = 11.6 Hz, 1H), 4.20 (ddd, J = 11.6, 4.8, 2.8 Hz, 1H), 2.80–2.76 (m, 1H), 2.69 (dd, J = 14.0, 9.2 Hz, 1H), 2.59 (dd, J = 14.0, 8.0 Hz, 1H), 2.37–2.31 (m, 1H), 2.29–2.15 (m, 2H), 2.07–1.99 (m, 2H), 1.66–1.60 (m, 1H), 1.57–1.50 (m, 1H), 1.28 (d, J = 7.2 Hz, 3H); **^{13}C NMR** (100 MHz, $CDCl_3$) δ 179.5, 172.6, 156.2, 144.6, 139.9, 129.4, 124.0, 107.8, 80.4, 74.2, 72.1, 56.3, 42.9, 42.4, 39.1, 24.7, 21.7, 19.6, 13.7; **FT-IR** (neat): ν_{max} = 3366, 2924, 1740, 1697, 1204, 1161, 1020, 908, 800, 727 cm^{-1}; **HRMS** (ESI): exact mass calculated for:

$C_{19}H_{20}O_6Na$ [M+Na]$^+$: 367.1152, found: 367.1154. The spectroscopic data were identical with those reported in the literature.[14]

8. Enantioselective Synthesis of (+)-2-Hydroxyteuscorolide

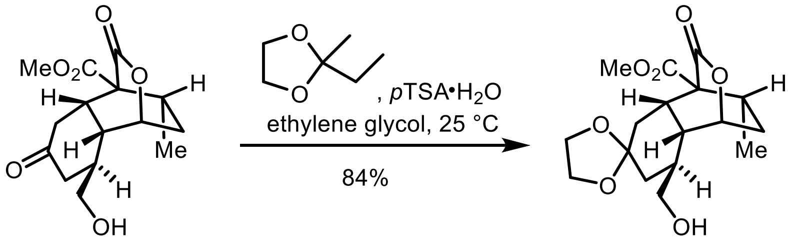

16 **32**

To a solution of ketone **16** (1.77 g, 5.97 mmol) in 2-methyl-2-ethyl-1,3-dioxolane (6.0 mL) was added *p*TSA•H_2O (56.8 mg, 0.30 mmol) and ethylene glycol (50 µL, 0.90 mmol) at 25 °C. After stirring at this temperature for 8 h, the reaction mixture was diluted with H_2O (10 mL). The resulting mixture was extracted with Et_2O (10 mL × 3). The combined extracts were washed with $NaHCO_3$ (10 mL, aq., sat.), dried over Na_2SO_4, and concentrated under reduced pressure. The residue was purified by flash column chromatography (silica gel, petroleum ether: ethyl acetate = 1: 2) to afford ketal **32** (1.71 g, 84%) as a white foam.

32: m.p. 49.1-50.5 °C; $[\alpha]_D^{23} = -20.18$ (c = 1.00, $CHCl_3$); **^{1}H NMR** (400 MHz, $CDCl_3$) δ 4.87 (dd, J = 5.6, 3.2 Hz, 1H), 3.98–3.84 (m, 4H), 3.80 (s, 3H), 3.54 (d, J = 5.2 Hz, 2H), 2.81–2.73 (m, 1H), 2.66–2.56 (m, 1H), 2.37–2.30 (m, 2H), 2.13–2.07 (m, 1H), 2.03 (dd, J = 14.8, 4.0 Hz, 1H), 1.94–1.82 (m, 3H), 1.66–1.55 (m, 2H), 1.23 (d, J = 7.2 Hz, 3H); **^{13}C NMR** (100 MHz, $CDCl_3$) δ 173.5, 169.2, 109.0, 76.8, 65.1, 64.0, 63.9, 55.6, 52.4, 40.4, 38.0, 34.9, 32.6, 31.5, 30.1, 28.2, 19.0; **FT-IR** (neat): ν_{max} = 3549, 2957, 2887, 1732, 1435, 1333, 1258, 1098, 1065, 1032, 947, 908 cm^{-1}; **HRMS** (EI): exact mass calculated for: $C_{17}H_{24}O_7$ [M]$^+$: 340.1517, found: 340.1516.

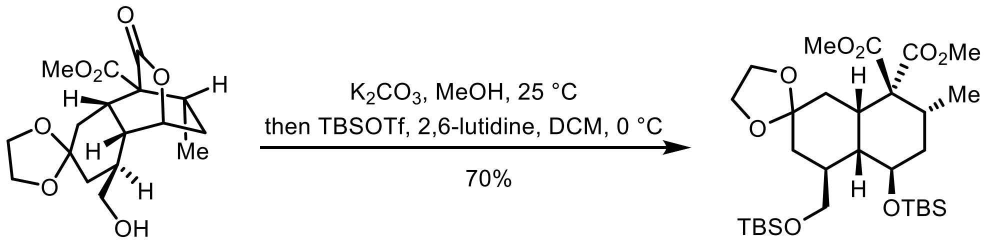

32 **33**

To a solution of lactone **32** (1.82 g, 5.35 mmol) in anhydrous MeOH (54 mL) was added K_2CO_3 (1.85 g, 13.37 mmol) at 25 °C. After stirring at this temperature for 16 h, the reaction mixture was neutralized with dilute AcOH (30 mL, aq., 0.5 M). The resulting mixture was extracted with EtOAc

(50 mL × 3). The combined extracts were washed with brine, dried over Na_2SO_4, and concentrated under reduced pressure. The residue was used directly in the next step without further purification.

To a solution of the above crude product in DCM (139 mL) was added 2,6-lutidine (3.74 mL, 32.08 mmol) and TBSOTf (3.69 mL, 16.04 mmol) at 0 °C. After stirring at this temperature for 2 h, the reaction mixture was quenched with $NaHCO_3$ (100 mL, aq., sat.). The resulting mixture was extracted with DCM (100 mL × 3). The combined extracts were washed with brine, dried over Na_2SO_4, and concentrated under reduced pressure. The residue was purified by flash column chromatography (silica gel, petroleum ether: ethyl acetate = 25: 1) to afford **33** (2.26 g, 70%) as a colorless liquid.

33: $[\alpha]_D^{20}$ = −33.96 (*c* = 1.03, $CHCl_3$); **^{1}H NMR** (400 MHz, $CDCl_3$) δ 4.04–3.94 (m, 3H), 3.88 (t, *J* = 6.4 Hz, 2H), 3.71 (s, 3H), 3.63 (s, 3H), 3.63 (dd, *J* = 18.8, 9.2 Hz, 8H), 3.06 (d, *J* = 14.0 Hz, 1H), 2.96–2.93 (m, 1H), 2.37 (dd, *J* = 13.6, 6.8 Hz, 1H), 2.21 (d, *J* = 12.8 Hz, 1H), 1.86–1.63 (m, 5H), 1.51–1.47 (m, 1H), 1.12 (d, *J* = 7.2 Hz, 3H), 0.86 (s, 18H), 0.05–0.01 (m, 12H); **^{13}C NMR** (100 MHz, $CDCl_3$) δ 171.2, 170.1, 109.0, 64.8, 64.6 63.9, 63.7, 61.7, 53.0, 52.3, 41.6, 39.8, 35.62, 35.56, 33.7, 32.4, 31.3, 25.9, 25.8, 18.6, 18.2, 18.0, −4.0, −4.7, −5.25 , −5.32; **FT-IR** (neat): ν_{max} = 2928, 2855, 1735, 1472, 1256, 1213, 1084, 1024, 980, 835 cm^{-1}; **HRMS** (ESI): exact mass calculated for: $C_{30}H_{56}O_8NaSi_2$ $[M+Na]^+$: 623.3406, found: 623.3407.

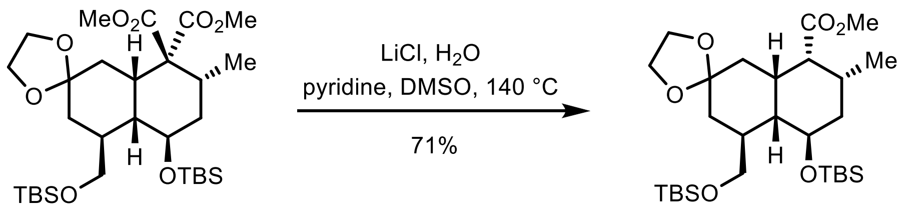

33 **34**

To a mixture of diester **33** (1.01 g, 1.68 mmol) in DMSO (4.2 ml) was added anhydrous LiCl (214 mg, 5.04 mmol), H_2O (91 μL, 5.04 mmol) and pyridine (133 mg, 1.68 mmol) at 25 °C. The reaction mixture was stirred at 140 °C for 12 h. The resulting mixture was diluted with H_2O (50 ml) and extracted with EtOAc (50 mL × 4). The combined extracts were washed with brine, dried over Na_2SO_4, and concentrated under reduced pressure. The residue was purified by flash column chromatography (silica gel, petroleum ether: ethyl acetate = 30: 1) to afford **34** (652 mg, 71%, single isomer) as a colorless oil.

34: $[\alpha]_D^{21}$ = −29.93 (*c* = 1.08, $CHCl_3$); **^{1}H NMR** (400 MHz, $CDCl_3$) δ 3.99–3.93 (m, 3H), 3.90–3.84 (m, 2H), 3.68–3.62 (m, 5H), 2.64–2.57 (m, 2H), 2.51–2.39 (m, 2H), 1.95–1.88 (m, 2H), 1.77–1.60 (m, 3H), 1.53–1.46 (m, 2H), 1.07 (d, *J* = 7.6 Hz, 3H), 0.87 (s, 18H), 0.05 (s, 3H), 0.04 (s, 3H), 0.01 (s, 6H); **^{13}C NMR** (100 MHz, $CDCl_3$) δ 173.8, 109.1, 65.1, 64.4, 64.1, 63.6, 51.3, 48.7, 44.6, 42.2, 35.6,

34.8, 32.4, 31.8, 29.9, 25.9, 25.8, 18.3, 18.2, 18.0, −4.1, −4.7, −5.2, −5.4; **FT-IR** (neat): ν_{max} = 2930, 2855, 1736, 1472, 1360, 1250, 1202, 1076, 970, 833, 772 cm^{-1}; **HRMS** (ESI): exact mass calculated for: $C_{28}H_{54}O_6NaSi_2$ $[M+Na]^+$: 565.3351, found: 565.3344.

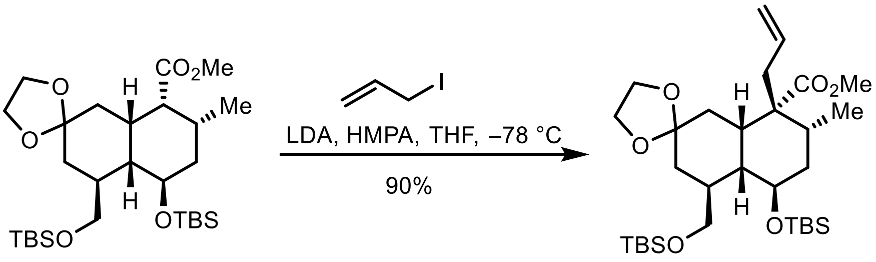

34 **S-7**

To a solution of **34** (968 mg, 1.78 mmol) in THF (18 mL) was added LDA (8.92 mL, 8.92 mmol, 1.0 M in THF) at –78 °C. After stirring at this temperature for 6 h, the reaction mixture was added HMPA (1.60 g, 8.92 mmol) and allyl iodide (449 mg, 2.67 mmol) sequentially. The reaction mixture was allowed to warm to 0 °C. After stirring at this temperature for 2 h, the reaction mixture was quenched with NH_4Cl (20 mL, aq., sat.). The resulting mixture was extracted with Et_2O (20 mL × 3). The combined extracts were washed with brine, dried over Na_2SO_4, and concentrated under reduced pressure. The residue was purified by flash column chromatography (silica gel, petroleum ether: ethyl acetate = 25: 1) to afford allylation product **S-7** (932 mg, 90% yield) as a colorless oil.

S-7: $[\alpha]_D^{20}$ = −27.03 (*c* = 1.00, $CHCl_3$); **^{1}H NMR** (400 MHz, $CDCl_3$) δ 5.68–5.57 (m, 1H), 5.06 (s, 1H), 5.02 (d, *J* = 6.0 Hz, 1H), 4.03–3.94 (m, 3H), 3.85 (t, *J* = 6.4 Hz, 2H), 3.66 (s, 3H), 3.61 (d, *J* = 7.6 Hz, 2H), 2.59 (dd, *J* = 14.0, 7.6 Hz, 1H), 2.47 (dd, *J* = 14.0, 7.2 Hz, 1H), 2.41–2.33 (m, 2H), 2.20 (br, 1H), 1.93–1.83 (m, 2H), 1.77–1.62 (m, 5H), 1.12 (d, *J* = 7.2 Hz, 3H), 0.88 (s, 18H), 0.06 (s, 3H), 0.04 (s, 3H), 0.02 (s, 6H); **^{13}C NMR** (100 MHz, $CDCl_3$) δ 175.1, 133.7, 118.0, 109.3, 65.0, 64.6, 64.5, 63.6, 53.2, 51.0, 42.7, 39.0, 38.5, 35.6, 35.5, 34.8, 33.3, 31.5, 25.89, 25.86, 19.7, 18.2, 18.0, −4.0, −4.7, −5.25, −5.31; **FT-IR** (neat): ν_{max} = 2926, 2855, 1732, 1472, 1256, 1088, 835, 773 cm^{-1}; **HRMS** (ESI): exact mass calculated for: $C_{31}H_{58}O_6NaSi_2$ $[M+Na]^+$: 605.3664, found: 605.3671.

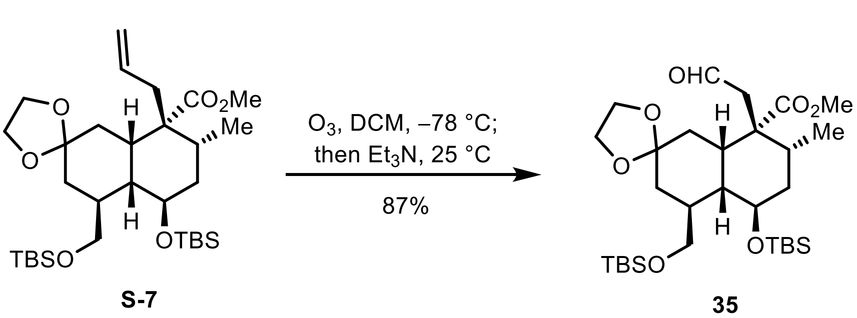

S-7 **35**

To a solution of **S-7** (885 mg, 1.52 mmol) in DCM (152 mL) at −78 °C was bubbled with ozone at −78 °C until the solution turned blue. After the excess ozone was excluded by the argon stream, Et_3N (422 μL, 3.03 mmol) was added. After stirring at 25 °C for 2 h, the reaction mixture was concentrated under reduced pressure. The residue was purified by flash column chromatography (silica gel, petroleum ether: ethyl acetate = 8: 1) to afford aldehyde **35** (772 mg, 87% yield) as a colorless oil.

35: $[\alpha]_D^{22}$ = −34.19 (*c* = 1.01, $CHCl_3$); **^{1}H NMR** (400 MHz, $CDCl_3$) δ 9.68 (s, 1H), 4.04–3.98 (m, 1H), 3.95–3.83 (m, 4H), 3.69 (s, 3H), 3.63–3.55 (m, 2H), 2.80–2.70 (m, 2H), 2.41–2.38 (m, 2H), 2.18–2.15 (m, 1H), 1.89–1.82 (m, 3H), 1.74–1.64 (m, 4H), 1.13 (d, *J* = 7.2 Hz, 3H), 0.87 (s, 18H), 0.05 (s, 3H), 0.04 (s, 3H), 0.02–0.01 (m, 6H); **^{13}C NMR** (100 MHz, $CDCl_3$) δ 200.7, 174.1 108.9, 64.7, 64.6, 64.5, 63.7, 51.5, 50.7, 39.1, 38.4, 36.8, 35.6, 35.3, 33.6, 31.7, 25.9, 25.9, 19.0, 18.1, 17.9, −4.1, −4.7, −5.32, −5.34; **FT-IR** (neat): ν_{max} = 2953, 2859, 1722, 1472, 1250, 1086, 976, 835, 773, 669 cm^{-1}; **HRMS** (ESI): exact mass calculated for: $C_{30}H_{56}O_7NaSi_2$ $[M+Na]^+$: 607.3457, found: 607.3463.

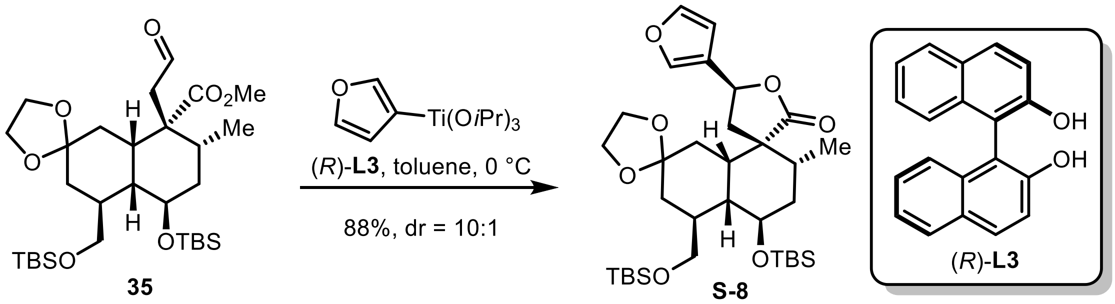

To a solution of (3-furyl)Ti(O*i*Pr)$_3$[6] (99.9 mg, 0.34 mmol) and (*R*)-**L3** (9.8 mg, 20 mol%) in toluene (3.4 mL) was added aldehyde **35** (100 mg in 0.3 mL toluene, 0.17 mmol) dropwise at 0 °C. After stirring at this temperature for 2 h, the reaction mixture was quenched with NaOH (5.0 mL, aq., 1.0 M). The resulting mixture was extracted with EtOAc (5.0 mL × 3). The combined extracts were washed with brine, dried over Na_2SO_4, and concentrated under reduced pressure. The residue was purified by flash chromatography column (silica gel, petroleum ether: ethyl acetate = 20: 1) to afford **S-8** (93.7 mg, 88%, 10: 1 dr) as a white foam.

S-8: m.p. 50.1-51.5 °C; $[\alpha]_D^{21}$ = −12.57 (*c* = 1.01, $CHCl_3$); **^{1}H NMR** (400 MHz, $CDCl_3$) δ 7.45 (s, 1H), 7.42 (s, 1H), 6.39 (s, 1H), 5.35–5.31 (m, 1H), 4.07 (br s, 1H), 3.90 (s, 4H), 3.66–3.58 (m, 2H), 2.60 (dd, *J* = 12.0, 6.0 Hz, 1H), 2.45 (d, *J* = 3.2 Hz, 1H), 2.30 (br s, 1H), 2.21–2.16 (m, 1H), 2.05–1.97 (m, 2H), 1.90–1.78 (m, 3H), 1.72 (dd, *J* = 13.6, 5.6 Hz, 1H), 1.62–1.58 (m, 1H), 1.48 (t, *J* = 8.0 Hz, 1H), 1.24 (d, *J* = 12.0 Hz, 3H), 0.88 (s, 9H), 0.87 (s, 9H), 0.06–0.04 (m, 12H); **^{13}C NMR** (100 MHz, $CDCl_3$) δ 177.1, 143.9, 139.6, 124.6, 108.9, 108.2, 70.6, 65.2, 64.6, 64.5, 64.1, 50.1, 44.8, 36.2, 35.4, 35.1, 25.9, 25.8, 18.2, 17.9, 16.8, −4.3, −4.7, −5.3, −5.4; **FT-IR** (neat): ν_{max} = 2928, 2857, 1763, 1472,

1252, 1086, 1024, 874, 835, 773 cm^{-1}; **HRMS** (ESI): exact mass calculated for: $C_{33}H_{56}O_7NaSi_2$ $[M+Na]^+$: 643.3457, found: 643.3462.

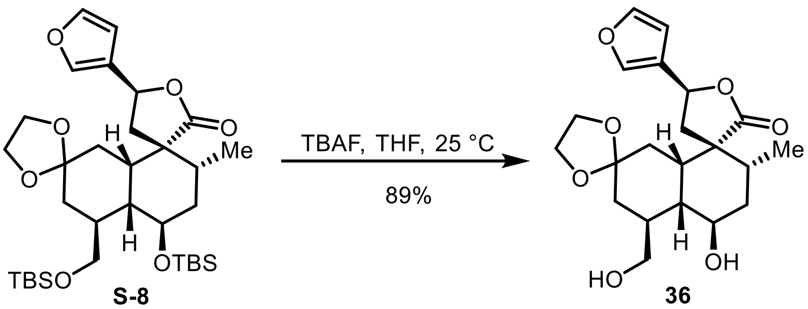

S-8 36

To a solution of **S-8** (5.6 mg, 0.009 mmol) in THF (0.3 ml) was added TBAF (27 μL, 0.027 mmol, 1.0 M in THF) dropwise at 25 °C. After stirring at this temperature for 12 h, the reaction mixture was quenched with NH_4Cl (1.0 mL, aq., sat.). The resulting mixture was extracted with EtOAc (1.0 mL × 3). The combined extracts were washed with brine, dried over Na_2SO_4, and concentrated under reduced pressure. The residue was purified by flash column chromatography (silica gel, dichloromethane: methanol = 25: 1) to afford diol **36** (3.1 mg, 89% yield) as a white foam.

36: m.p. 61.3-62.3 °C; $[\alpha]_D^{22}$ = −11.68 (*c* = 1.04, $CHCl_3$); **^{1}H NMR** (400 MHz, $CDCl_3$) δ 7.47 (s, 1H), 7.42 (t, *J* = 1.6 Hz, 1H), 6.40 (d, *J* = 1.2 Hz, 1H), 5.35 (dd, *J* = 9.6, 6.4 Hz, 1H), 4.03 (ddd, *J* = 9.2, 7.2, 4.8 Hz, 1H), 3.92 (s, 4H), 3.75 (dd, *J* = 11.2, 6.8 Hz, 1H), 3.68 (dd, *J* = 11.2, 5.4 Hz, 1H), 2.67 (dd, *J* = 13.3, 6.4 Hz, 1H), 2.52–2.39 (m, 4H), 2.18 (dd, *J* = 13.2, 10.0 Hz, 1H), 2.09 (ddd, *J* = 13.2, 6.8, 4.8 Hz, 1H), 2.05–1.97 (m, 1H), 1.92–1.76 (m, 4H), 1.63 (d, *J* = 14.0 Hz, 1H), 1.54 (ddd, *J* = 13.6, 7.2, 4.0 Hz, 1H), 1.25 (d, *J* = 7.2 Hz, 3H); **^{13}C NMR** (100 MHz, $CDCl_3$) δ 176.9, 144.0, 139.8, 124.3, 108.6, 108.2, 70.7, 66.4, 64.63, 64.61, 64.1, 50.1, 44.6, 41.0, 38.2, 36.6, 35.9, 35.7, 34.8, 33.8, 16.7; **FT-IR** (neat): ν_{max} = 3468, 2934, 1757, 1506, 1269, 1182, 1157, 1022, 968, 874, 795, 734 cm^{-1}; **HRMS** (ESI): exact mass calculated for: $C_{21}H_{28}O_7Na$ $[M+Na]^+$: 415.1727, found: 415.1721.

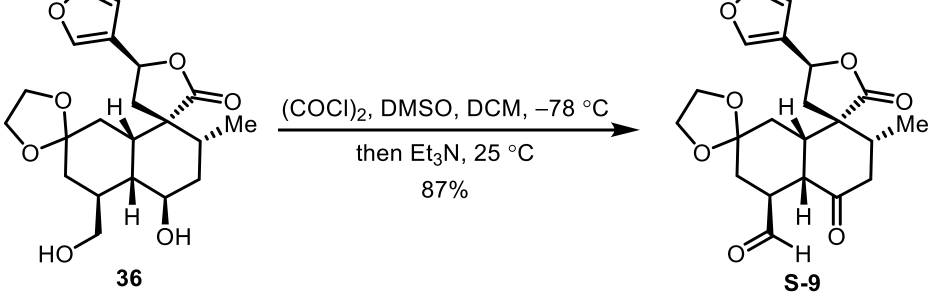

36 S-9

To a stirred solution of DCM (5.2 mL) and oxalyl chloride (105 μL, 1.24 mmol) was added DMSO (177 μL, 2.49 mmol) dropwise at −78 °C. After stirring at this temperature for 10 min, the reaction

mixture was added **36** (203 mg, 0.52 mmol) slowly and stirred for another 30 min. Then Et_3N (865 μL, 6.22 mmol) was added and the resulting mixture was stirred at −78 °C for 5 min. After warming to 25 °C slowly and stirring at this temperature for 2 h, the reaction mixture was quenched with $NaHCO_3$ (5.0 mL, aq., sat.). The resulting mixture was extracted with DCM (5.0 mL × 3). The combined extracts were washed brine, dried over Na_2SO_4, and concentrated under reduced pressure. The residue was purified by flash column chromatography (silica gel, petroleum ether: ethyl acetate = 2: 1) to afford ketoaldehyde **S-9** (176 mg, 87% yield) as a colorless oil.

S-9: $[\alpha]_D^{30}$ = −156.77 (*c* = 1.01, $CHCl_3$); **^{1}H NMR** (400 MHz, $CDCl_3$) δ 9.64 (s, 1H), 7.51 (d, *J* = 0.4 Hz, 1H), 7.45 (t, *J* = 1.6 Hz, 1H), 6.43 (s, 1H), 5.40 (dd, *J* = 10.4, 5.6 Hz, 1H), 4.02–3.87 (m, 4H), 3.43 (d, *J* = 5.2 Hz, 1H), 3.32–3.30 (m, 1H), 2.84 (dd, *J* = 18.4, 12.8 Hz, 1H), 2.65 (dd, *J* = 12.8, 5.6 Hz, 1H), 2.55 (dt, *J* = 12.8, 5.6 Hz, 1H), 2.42–2.34 (m, 2H), 2.32–2.23 (m, 1H), 2.19 (d, *J* = 14.0 Hz, 1H), 1.96 (dd, *J* = 14.0, 7.2 Hz, 1H), 1.62–1.58 (m, 1H), 1.55–1.49 (m, 1H), 1.16 (d, *J* = 6.8 Hz, 3H); **^{13}C NMR** (100 MHz, $CDCl_3$) δ 210.3, 202.7, 175.6, 144.1, 140.1, 123.4, 108.2, 107.5, 71.3, 64.8, 64.7, 50.3, 45.3, 44.6, 42.9, 42.6, 39.2 37.4, 33.0, 31.3, 15.6; **FT-IR** (neat): ν_{max} = 2962, 1755, 1707, 1506, 1354, 1157, 1117, 1026, 874, 812, 733 cm^{-1}; **HRMS** (EI): exact mass calculated for: $C_{21}H_{24}O_7$ $[M]^+$: 388.1517, found: 388.1520.

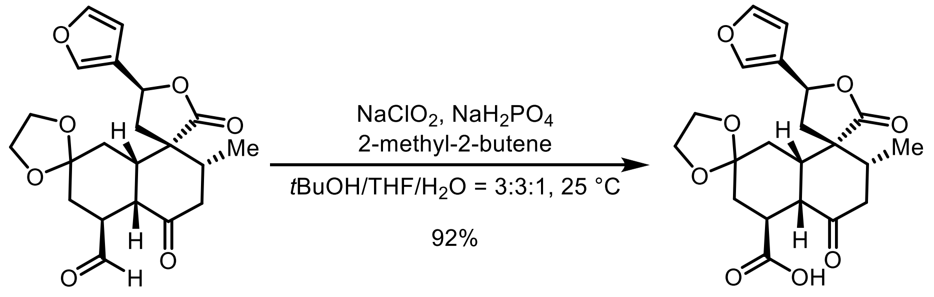

S-9 **37**

To a mixture of ketoaldehyde **S-9** (354 mg, 0.91 mmol) and NaH_2PO_4 (394 mg, 3.28 mmol) in *t*BuOH/THF/H_2O (v:v:v = 3:3:1, 30.3 mL) was added 2-methyl-2-butene (1.45 mL, 17.2 mmol) and $NaClO_2$ (346 mg, 3.83 mmol) sequentially at 25 °C. After stirring at this temperature for 1 h, the reaction mixture was diluted with NH_4Cl (30 mL, aq., sat.). The resulting mixture was extracted with EtOAc (40 mL × 3). The combined extracts were washed with brine, dried over Na_2SO_4, and concentrated under reduced pressure. The residue was purified by flash column chromatography (silica gel, dichloromethane: methanol = 20: 1) to afford carboxylic acid **37** (338 mg, 92% yield) as a white solid.

37: m.p. 215.0-215.9 °C; $[\alpha]_D^{21}$ = −2.49 (*c* = 0.64, $CHCl_3$); **^{1}H NMR** (400 MHz, CD_3CN) δ 7.66 (s, 1H), 7.54 (t, *J* = 1.6 Hz, 1H), 6.56 (d, *J* = 1.2 Hz, 1H), 5.47 (dd, *J* = 10.8, 5.6 Hz, 1H), 3.94–3.80 (m,

4H), 3.40–3.35 (m, 2H), 2.87 (dt, J = 13.2, 5.2 Hz, 1H), 2.67 (dd, J = 18.0, 12.4 Hz, 1H), 2.58 (dd, J = 12.8, 6.0 Hz, 1H), 2.44 (dd, J = 12.8, 10.8 Hz, 1H), 2.40–2.28 (m, 2H), 2.20 (d, J = 14.0 Hz, 1H), 1.61 (dd, J = 14.0, 6.4 Hz, 1H), 1.52–1.41 (m, 2H), 1.10 (d, J = 6.4 Hz, 3H); **^{13}C NMR** (100 MHz, CD_3CN) δ 210.9, 177.1, 175.5, 145.2, 142.1, 124.8, 109.5, 108.4, 72.1, 65.2, 65.1, 51.4, 46.0, 45.0, 43.3, 40.4, 38.7, 37.6, 33.5, 32.4, 15.9; **FT-IR** (neat): ν_{max} = 2965, 1765, 1717, 1686, 1506, 1159, 1142, 1016, 874, 791, 781, 735, 683 cm^{-1}; **HRMS** (EI): exact mass calculated for: $C_{21}H_{24}O_8$ [M]$^+$: 404.1466, found: 404.1469.

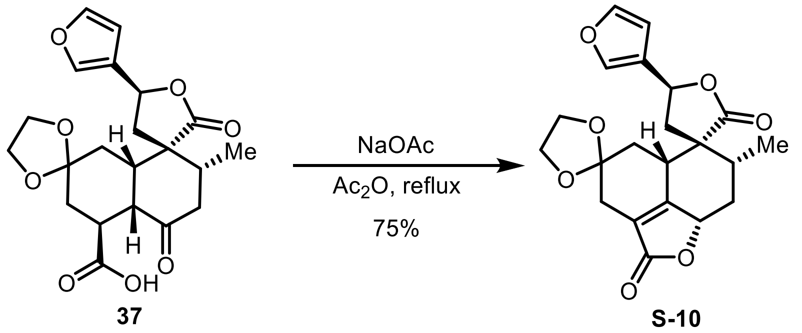

To a solution of **37** (209 mg, 0.52 mmol) in Ac_2O (4.0 mL) was added NaOAc (19.0 mg, 0.23 mmol) at 25 °C. After stirring at 150 °C for 20 h, the reaction mixture was cooled to 25 °C and diluted with EtOAc (50 mL). The resulting mixture was washed with brine, dried over Na_2SO_4, and concentrated under reduced pressure. The residue was purified by column chromatography (silica gel, dichloromethane: methanol = 20: 1) to afford **S-10** (150 mg, 75%) as a white solid.

S-10: the m.p. could not be determined because this compound will be decomposed at 270 °C; $[\alpha]_D^{26}$ = +96.18 (c = 0.25, $CHCl_3$); **^{1}H NMR** (400 MHz, $CDCl_3$) δ 7.45 (s, 2H), 6.38 (s, 1H), 5.41 (t, J = 8.4 Hz, 1H), 4.83–4.79 (m, 1H), 4.14–3.90 (m, 4H), 2.96–2.92 (m, 1H), 2.55 (d, J = 8.4 Hz, 2H), 2.45–2.38 (m, 2H), 2.31 (dd, J = 24.0, 12.4 Hz, 1H), 2.23–2.15 (m, 2H), 1.96–1.87 (m, 1H), 1.76 (dd, J = 12.4, 10.8 Hz, 1H), 1.09 (d, J = 6.8 Hz, 3H); **^{13}C NMR** (100 MHz, $CDCl_3$) δ 175.4, 172.2, 160.8, 144.3, 139.6, 124.7, 124.3, 108.7, 107.9, 78.0, 71.7, 65.0, 64.7, 53.4, 41.6, 40.6, 36.0, 34.9, 34.4, 30.6, 17.0; **FT-IR** (neat): ν_{max} = 1761, 1740, 1694, 1344, 1292, 1180, 1042, 1013, 962, 872, 754 cm^{-1}; **HRMS** (EI): exact mass calculated for: $C_{21}H_{22}O_7$ [M]$^+$: 386.1360, found: 386.1360.

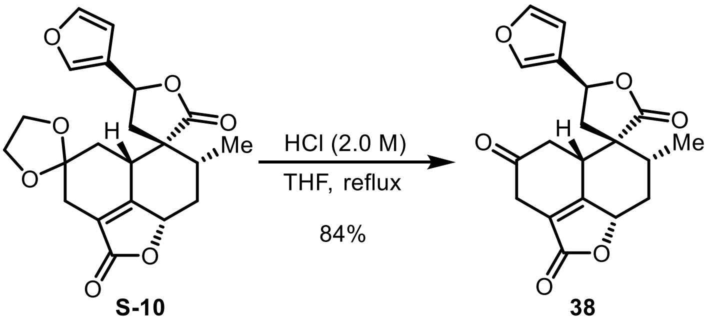

To a solution of **S-10** (176 mg, 0.45 mmol) in THF (4.6 mL) was added HCl (0.91 mL, aq., 2.0 M). After stirring under reflux for 20 h, the reaction mixture was quenched with $NaHCO_3$ (5.0 mL, aq., sat.) at 25 °C. The resulting mixture was extracted with EtOAc (5.0 mL × 3). The combined extracts were washed with brine, dried over Na_2SO_4, and concentrated under reduced pressure. The residue was purified by flash column chromatography (silica gel, petroleum ether: ethyl acetate = 1: 2) to afford ketone **38** (131 mg, 84%) as a white solid.

38: m.p. 207.5-209.3 °C; $[\alpha]_D^{26}$ = +127.73 (*c* = 0.90, $CHCl_3$); **^{1}H NMR** (400 MHz, $CDCl_3$) δ 7.43–7.39 (m, 2H), 6.32 (s, 1H), 5.42 (t, *J* = 8.4 Hz, 1H), 4.82 (t, *J* = 8.8 Hz, 1H), 3.15–3.10 (m, 2H), 3.03–2.97 (m, 1H), 2.89 (dd, *J* = 15.2, 8.0 Hz, 1H), 2.51–2.41 (m, 3H), 2.27–2.21 (m, 1H), 2.13 (dd, *J* = 23.6, 12.4 Hz, 1H), 1.94–1.85 (m, 1H), 0.99 (d, *J* = 6.8 Hz, 3H); **^{13}C NMR** (100 MHz, $CDCl_3$) δ 202.8, 175.1, 171.1, 159.6, 144.4, 139.5, 124.6, 123.4, 107.8, 78.0, 72.0, 53.8, 42.7, 40.0, 39.4, 36.5, 36.0, 34.9, 17.1; **FT-IR** (neat): ν_{max} = 2930, 1736, 1701, 1510, 1175, 1148, 1015, 874, 806 cm^{-1}; **HRMS** (EI): exact mass calculated for: $C_{19}H_{18}O_6$ $[M]^+$: 342.1098, found: 342.1101.

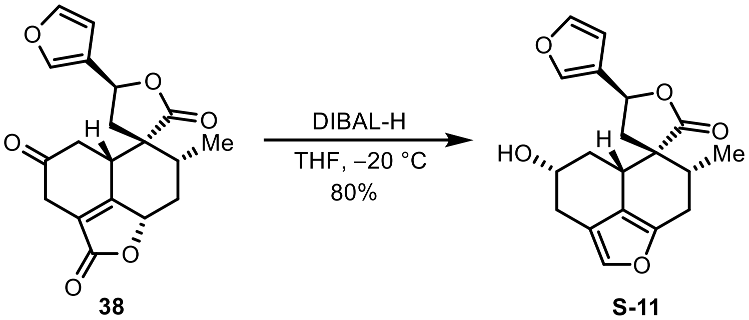

38 **S-11**

To a solution of compound **38** (13.4 mg, 0.039 mmol) in THF (1.0 mL) was added DIBAL-H (117 µL, 0.117 mmol, 1.0 M in hexane) dropwise at −20 °C. After stirring at this temperature for 30 min, the reaction mixture was quenched with HCl (60 µL, aq., 2.0 M) to adjust the pH value to 1~2. The resulting mixture was extracted with EtOAc (1.0 mL× 3). The combined extracts were washed with brine, dried over Na_2SO_4, and concentrated under reduced pressure. The residue was purified by flash chromatography column (silica gel, petroleum ether: ethyl acetate = 1: 1) to afford **S-11** (10.3 mg, 80%) as a colorless oil.

S-11: $[\alpha]_D^{25}$ = +86.05 (*c* = 0.44, $CHCl_3$); **^{1}H NMR** (400 MHz, $CDCl_3$) δ 7.46 (d, *J* = 0.8 Hz, 1H), 7.44 (t, *J* = 1.6 Hz, 1H), 7.07 (s, 1H), 6.401–6.395 (m, 1H), 5.46 (t, *J* = 8.4 Hz, 1H), 4.13–4.06 (m, 1H), 3.07 (dd, *J* = 15.6, 6.0 Hz, 1H), 2.92–2.87 (m, 1H), 2.84–2.74 (m, 2H), 2.62 (dd, *J* = 13.6, 8.8 Hz, 1H), 2.48 (dd, *J* = 13.6, 8.4 Hz, 1H), 2.35–2.23 (m, 3H), 1.90 (br s, 1H), 1.43 (dd, *J* = 23.6, 11.2 Hz, 1H), 1.16 (d, *J* = 6.8 Hz, 3H); **^{13}C NMR** (100 MHz, $CDCl_3$) δ 175.3, 148.1, 144.1, 139.7, 136.4, 125.2, 118.5, 116.0, 108.0, 71.6, 70.0, 50.5, 41.4, 39.5, 36.4, 35.5, 29.9, 29.8, 17.7; **FT-IR** (neat): ν_{max} = 3445,

2969, 1754, 1502, 1266, 1177, 1151, 1014, 871, 807, 732 cm^{-1}; **HRMS** (ESI): exact mass calculated for: $C_{19}H_{20}O_5Na$ [M+Na]$^+$: 351.1203, found: 351.1197.

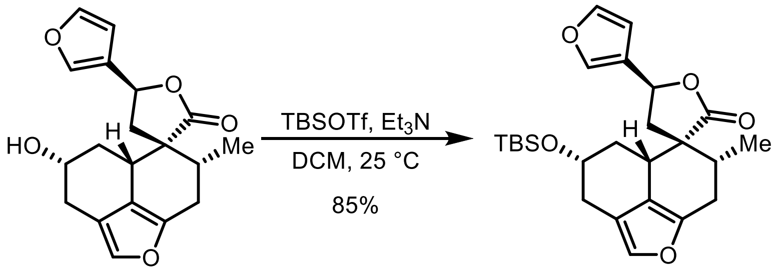

S-11 **39**

To a solution of **S-11** (8.7 mg, 0.027 mmol) in DCM (0.54 mL) were added Et_3N (7.4 μL, 0.053 mmol) and TBSOTf (9.2 μL, 0.040 mmol) at 25 °C. After stirring at this temperature for 1 h, the reaction mixture was quenched with $NaHCO_3$ (1.0 mL, aq., sat.). The resulting mixture was extracted with EtOAc (1.0 mL × 3). The combined extracts were washed with brine, dried over Na_2SO_4, and concentrated under reduced pressure. The residue was purified by flash chromatography column (silica gel, petroleum ether: ethyl acetate = 10: 1) to afford **39** (9.9 mg, 85%) as a colorless oil.

39: $[\alpha]_D^{22}$ = +61.15 (*c* = 0.33, $CHCl_3$); **^{1}H NMR** (400 MHz, $CDCl_3$) δ 7.49 (s, 1H), 7.45 (t, *J* = 4.6 Hz, 1H), 7.05 (s, 1H), 6.42 (s, 1H), 5.43 (t, *J* = 8.4 Hz, 1H), 4.11–4.03 (m, 1H), 2.93 (dd, *J* = 16.0, 6.0 Hz, 1H), 2.89–2.85 (m, 1H), 2.84–2.73 (m, 2H), 2.62 (dd, *J* = 13.6, 8.8 Hz, 1H), 2.49 (dd, *J* = 13.6, 8.4 Hz, 1H), 2.40–2.34 (m, 1H), 2.30–2.21 (m, 1H), 2.13–2.09 (m, 1H), 1.49 (q, *J* = 11.6 Hz, 1H), 1.15 (d, *J* = 6.8 Hz, 3H), 0.92 (s, 9H), 0.121 (s, 3H), 0.116 (s, 3H); **^{13}C NMR** (100 MHz, $CDCl_3$) δ 175.1, 148.0, 144.1, 139.7, 136.2, 125.4, 119.0, 116.1, 108.1, 71.4, 70.6, 50.5, 41.5, 39.4, 36.6, 35.9, 30.1, 30.0, 25.9, 18.1, 17.7, −4.4, −4.6; **FT-IR** (neat): ν_{max} = 2929, 2854, 1763, 1471, 1255, 1087, 1012, 871, 776, 741 cm^{-1}; **HRMS** (ESI): exact mass calculated for: $C_{25}H_{34}O_5NaSi$ [M+Na]$^+$: 465.2068, found: 465.2062.

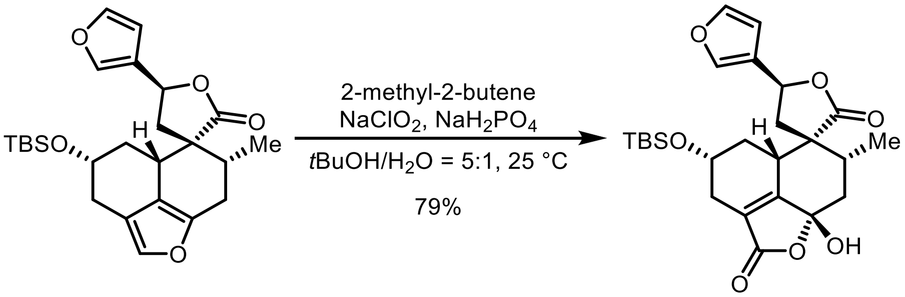

39 **40**

To a solution of **39** (7.9 mg, 0.018 mmol) in *t*BuOH/H_2O (v:v = 5:1, 0.6 mL) was added NaH_2PO_4 (8.5 mg, 0.071 mmol), 2-methyl-2-butene (30 μL, 0.357 mmol), and $NaClO_2$ (10.3 mg, 0.114 mmol)

sequentially at 25 °C. After stirring at this temperature for 2 h, the reaction mixture was diluted with H_2O (2.0 mL) and extracted with EtOAc (2.0 mL × 3). The combined extracts were washed with brine, dried over Na_2SO_4, and concentrated under reduced pressure. The residue was purified by column chromatography (silica gel, petroleum ether: ethyl acetate = 3: 2) to afford **40** (6.7 mg, 79%) as a white solid.

40: m.p. 252.8-253.1 °C; $[\alpha]_D^{26}$ = +89.24 (*c* = 0.22, $CHCl_3$); **^{1}H NMR** (400 MHz, $CDCl_3$) δ 7.48 (br s, 1H), 7.45 (br s, 1H), 6.404–6.400 (m, 1H), 5.44 (t, *J* = 8.4 Hz, 1H), 3.94–3.87 (m, 1H), 3.65 (br s, 1H), 2.99–2.94 (m, 1H), 2.62–2.46 (m, 4H), 2.29–2.07 (m, 4H), 1.52 (dd, *J* = 23.2, 11.8 Hz, 1H), 1.04 (d, *J* = 6.8 Hz, 3H), 0.90 (s, 9H), 0.08 (s, 6H); **^{13}C NMR** (100 MHz, $CDCl_3$) δ 175.5, 170.2, 159.4, 144.3, 139.6, 126.4, 124.8, 108.0, 102.0, 71.8, 68.2, 54.2, 40.21, 40.19, 39.8, 35.9, 34.4, 29.4, 25.8, 18.0, 16.7, −4.5, −4.7; **FT-IR** (neat): ν_{max} = 3393, 2934, 2865, 1761, 1469, 1259, 1182, 1101, 1043, 915, 835, 736 cm^{-1}; **HRMS** (ESI): exact mass calculated for: $C_{25}H_{34}O_7NaSi$ $[M+Na]^+$: 497.1966, found: 497.1958.

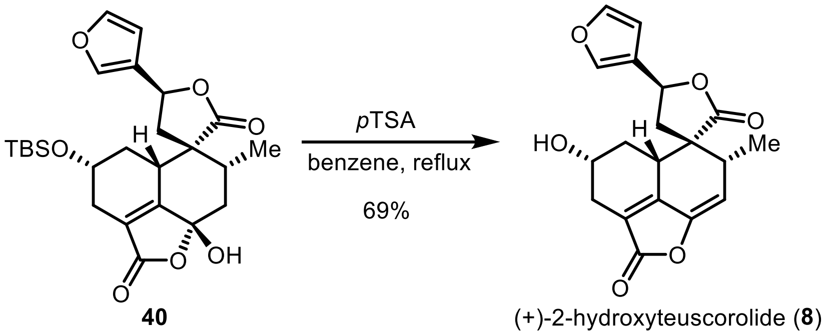

40 (+)-2-hydroxyteuscorolide (**8**)

To a solution of **40** (4.9 mg, 0.010 mmol) in benzene (2.5 mL) was added *p*TSA (3.5 mg, 0.021 mmol) at 25 °C. After stirring under reflux for 2 h, the reaction mixture was cooled to 25 °C, and concentrated under reduced pressure. The residue was purified by flash chromatography column (silica gel, petroleum ether: ethyl acetate = 1: 3) to afford (+)-2-hydroxyteuscorolide (**8**) (2.4 mg, 69%) as a white solid.

(+)-2-Hydroxyteuscorolide (**8**): m.p. 249.2-250.3 °C; $[\alpha]_D^{25}$ = +10.40 (*c* = 0.20, $CHCl_3$:C_5H_5N = 9:1); **^{1}H NMR** (400 MHz, pyridine-d_5) δ 7.81 (s, 1H), 7.71 (t, *J* = 2.0 Hz, 1H), 6.61 (s, 1H), 5.62 (t, *J* = 8.4 Hz, 1H), 5.46 (s, 1H), 4.43–4.34 (m, 1H), 3.15–3.12 (m, 1H), 2.99 (dd, *J* = 17.2, 5.2 Hz, 1H), 2.86–2.79 (m, 2H), 2.69 (dd, *J* = 14.0, 8.4 Hz, 1H), 2.55 (dd, *J* = 14.0, 9.2 Hz, 1H), 2.44 (ddd, *J* = 16.8, 9.2, 3.6 Hz, 1H), 1.86 (dd, *J* = 22.8, 11.6 Hz, 1H), 1.23 (d, *J* = 7.2 Hz, 3H); **^{13}C NMR** (100 MHz, pyridine-d_5) δ 175.1, 169.1, 151.3, 147.2, 144.7, 140.6, 125.5, 122.8, 109.1, 108.8, 71.7, 67.5, 53.7, 40.1, 39.0, 37.7, 34.6, 30.1, 16.7; **FT-IR** (neat): ν_{max} = 3488, 2929, 1753, 1736, 1663, 1181, 1154,

1026, 958, 769, 742 cm^{-1}; **HRMS** (ESI): exact mass calculated for: $C_{19}H_{18}O_6Na$ $[M+Na]^+$: 365.0996, found: 365.0987. The spectroscopic data were identical with those reported in the literature.[16]

9. Comparison of Natural Products with Synthetic 19-*nor*-Clerodane Diterpenoids

1) (+)-Teucvin (1)

Natural product: $[\alpha]_D^{25}$ = +68 (c = 0.15, $CHCl_3$);[7]

Synthetic product: $[\alpha]_D^{23}$ = +177.46 (c = 1.05, $CHCl_3$).

Table S3 ^{1}H NMR Data of (+)-Teucvin (**1**)

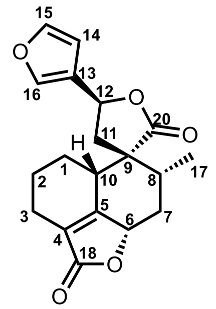

(+)-teucvin (**1**)

Carbon No.	This work ($CDCl_3$, 400 MHz)	Natural (ref. 7) ($CDCl_3$, 400 MHz)	Δδ (ppm)
1a	2.28–2.08 (m)	2.20 (m)	
1b	1.58–1.39 (m)	1.45 (m)	
2a	2.01–1.98 (m)	1.98 (m)	
2b	1.58–1.39 (m)	1.54 (m)	
3a	2.28–2.08 (m)	2.20 (m)	
3b	2.28–2.08 (m)	2.15 (m)	
6	4.76 (t, 8.8 Hz)	4.74 (dd, 9.8, 8.0 Hz)	+0.02
7	2.28–2.08 (m)	2.20 (m)	
8	1.93–1.86 (m)	1.86 (m)	
10	2.69 (br)	2.66 (m)	+0.03
11	2.55 (d, 8.8 Hz)	2.53 (d, 8.5 Hz)	+0.02
12	5.44 (t, 8.4 Hz)	5.43 (dd, 8.5, 8.5 Hz)	+0.01
14	6.38 (s)	6.37 (br s)	+0.01
15	7.45 (s)	7.44 (br s)	+0.01
16	7.44 (s)	7.44 (br s)	0.00
17	1.05 (d, 6.8 Hz)	1.05 (d, 6.7 Hz)	0.00

Table S4 ^{13}C NMR Data of (+)-Teucvin (**1**)

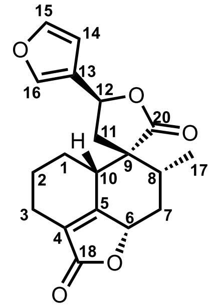

(+)-teucvin (**1**)

**Carbon No.**	**This work** (CDCl$_3$, 100 MHz)	**Natural (ref. 7)** (CDCl$_3$, 100 MHz)	Δδ (ppm)
1	24.8	24.8	0.0
2	21.7	21.7	0.0
3	19.6	19.6	0.0
4	126.6	126.6	0.0
5	161.3	161.3	0.0
6	78.2	78.2	0.0
7	35.2	35.3	−0.1
8	36.0	36.0	0.0
9	53.5	53.5	0.0
10	42.1	42.1	0.0
11	40.9	40.9	0.0
12	71.7	71.8	−0.1
13	124.9	125.0	−0.1
14	107.9	107.9	0.0
15	144.3	144.3	0.0
16	139.5	139.5	0.0
17	17.0	17.0	0.0
18	172.9	172.9	0.0
20	175.5	175.5	0.0

2) (+)-Montanin A (3)

Natural product: $[\alpha]_D^{21}$ = +120.2 (c = 0.32, $CHCl_3$);[8]

Synthetic product: $[\alpha]_D^{16}$ = +103.26 (c = 0.31, $CHCl_3$).

Table S5 ^{1}H NMR Data of (+)-Montanin A (**3**)

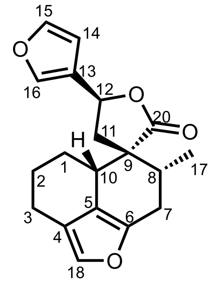

(+)-montanin A (**3**)

**Carbon No.**	**This work** (CDCl3, 400 MHz)	**Liu (ref. 9)** (CDCl3, 400 MHz)	**Natural (ref. 10)** (CDCl3, 400 MHz)	Δδ (ppm) **(**our synthetic–natural**)**
1a	1.30–1.24 (m)		1.23 (dddd)	
1b	2.15–2.05 (m)	2.17–2.05 (m)	2.10 (m)	
2a	2.15–2.05 (m)	2.17–2.05 (m)	2.08 (m)	
2b	1.71–1.58 (m)		1.63 (ddddd)	
3a	2.52–2.39 (m)	2.69–2.38 (m)	2.42 (dddd)	
3b	2.69 (dd, 16.4, 6.0)	2.69–2.38 (m)	2.68 (br dd)	+0.01
7	2.88–2.75 (m)	2.78 (m)	2.80 (m)	
8	2.31–2.21 (m)	2.28–2.21 (m)	2.25 (ddq)	
10	2.88–2.75 (m)	2.78 (m)	2.76 (m)	
11a	2.49 (dd, 8.4, 13.6 Hz)	2.69–2.38 (m)	2.48 (dd)	+0.01
11b	2.62 (dd, 8.8, 13.6 Hz)	2.69–2.38 (m)	2.61 (dd)	+0.01
12	5.44 (t, 8.8 Hz)	5.42 (t, 8.5 Hz)	5.43 (br t)	+0.01
14	6.41 (br)	6.39 (m)	6.40 (dd)	+0.01
15	7.44 (t, 2.0Hz)	7.42 (m)	7.43 (t)	+0.01
16	7.47 (s)	7.45 (s)	7.46 (dt)	+0.01
17	1.15 (d, 6.8 Hz)	1.13 (d, 6.8 Hz)	1.14 (d)	+0.01
18	7.06 (s)	7.04 (br s)	7.05 (td)	+0.01

Table S6 ^{13}C NMR Data of (+)-Montanin A (**3**)

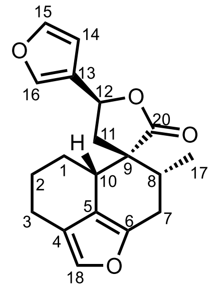

(+)-montanin A (**3**)

**Carbon No.**	**This work** (CDCl$_3$, 100 MHz)	**Liu (ref. 9)** (CDCl$_3$, 100 MHz)	**Natural (ref. 10)** (CDCl$_3$, 100 MHz)	Δδ (ppm) **(**our synthetic–natural**)**
1	25.6	25.6	25.6	0.0
2	23.9	23.9	23.9	0.0
3	19.1	19.1	19.1	0.0
4	119.7	119.7	119.7	0.0
5	116.9	116.9	116.9	0.0
6	147.9	147.9	147.9	0.0
7	30.0	30.0	30.0	0.0
8	36.1	36.1	36.1	0.0
9	50.7	50.7	50.7	0.0
10	43.2	43.2	43.2	0.0
11	39.7	39.7	39.7	0.0
12	71.6	71.6	71.6	0.0
13	125.5	125.5	125.5	0.0
14	108.1	108.1	108.1	0.0
15	144.1	144.1	144.1	0.0
16	139.6	139.6	139.6	0.0
17	17.8	17.7	17.7	+0.1
18	136.2	136.2	136.1	+0.1
20	175.5	175.5	175.5	0.0

3) (+)-Cracroson A (4)

Natural product: $[\alpha]_D^{25}$ = +27 (*c* = 1.0, MeOH);[11]

Synthetic product: $[\alpha]_D^{28}$ = +25.35 (*c* = 0.43, MeOH).

Table S7 ^{1}H NMR Data of (+)-Cracroson A (**4**)

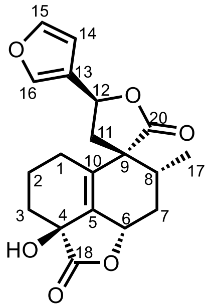

(+)-cracroson A (**4**)

Carbon No.	This work (CDCl3, 400 MHz)	Natural (ref. 11) (CDCl3, 400 MHz)	Δδ (ppm)
1a	2.19–1.95 (m)	2.00 (m)	
1b	2.41–2.37 (m)	2.37 (dd, 14.0, 9.2 Hz)	
2a	1.53–1.46 (m)	1.48 (dt, 14.0, 2.8 Hz)	
2b	2.19–1.95 (m)	2.08 (m)	
3a	2.19–1.95 (m)	1.95 (m)	
3b	2.19–1.95 (m)	2.21 (m)	
6	5.14 (t, 8.0 Hz)	5.14 (t, 7.6 Hz)	0.00
7	2.19–1.95 (m)	2.12 (m)	
8	1.87–1.78 (m)	1.82 (m)	
11a	2.30 (dd, 14.0, 9.2 Hz)	2.29 (dd, 14.0, 9.2 Hz)	+0.01
11b	2.74 (dd, 14.0, 8.4 Hz)	2.73 (dd, 14.0, 8.4 Hz)	+0.01
12	5.50 (t, 8.8 Hz)	5.49 (t, 8.4 Hz)	+0.01
14	6.42 (s)	6.42 (s)	0.00
15	7.47 (s)	7.46 (s)	+0.01
16	7.50 (s)	7.49 (s)	+0.01
17	1.14 (d, 6.8 Hz)	1.14 (d, 7.6 Hz)	0.00
-OH	2.59 (br s)	3.48 (s)	−0.89

Table S8 ^{13}C NMR Data of (+)-Cracroson A (**4**)

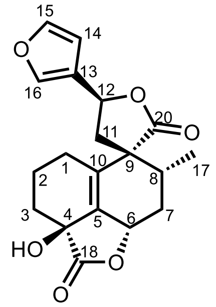

(+)-cracroson A (**4**)

**Carbon No.**	**This work** (CDCl3, 100 MHz)	**Natural (ref. 11)** (CDCl3, 100 MHz)	Δδ (ppm)
1	24.9	25.1	−0.2
2	29.8	29.9	−0.1
3	17.2	17.3	−0.1
4	69.1	69.2	−0.1
5	137.4	137.4	0.0
6	76.9	77.0	−0.1
7	32.5	32.6	−0.1
8	34.9	35.1	−0.2
9	52.5	52.7	−0.2
10	132.8	133.1	−0.3
11	39.5	39.7	−0.2
12	72.5	72.7	−0.2
13	124.4	124.6	−0.2
14	108.0	108.1	−0.1
15	144.3	144.5	−0.2
16	139.7	139.9	−0.2
17	16.1	16.2	−0.1
18	175.9	176.1	−0.2
20	176.0	176.2	−0.2

4) (+)-Teucvisin C (5)

Natural product: $[\alpha]_D^{25}$ = +176.4 (c = 0.11, MeOH);[12]

Synthetic product: $[\alpha]_D^{23}$ = +176.92 (c = 0.10, MeOH).

Table S9 ^{1}H NMR Data of (+)-Teucvisin C (**5**)

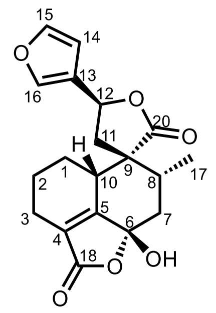

(+)-teucvisin C (**5**)

**Carbon No.**	**This work** (CDCl3, 400 MHz)	**Natural (ref. 12)** (CDCl3, 500 MHz)	Δδ (ppm)
1a	2.27–2.09 (m)	2.33 (m)	
1b	1.50–1.41 (m)	1.48 (m)	
2a	2.01–1.96 (m)	1.98 (m)	
2b	1.59–1.51 (m)	1.54 (m)	
3a	2.27–2.09 (m)	2.19 (m)	
3b	2.27–2.09 (m)	2.27 (m)	
7a	2.47 (t, 12.4 Hz)	2.50 (t, 13.0 Hz)	−0.03
7b	2.27–2.09 (m)	2.15 (dd, 13.0, 4.0 Hz)	
8	2.27–2.09 (m)	2.22 (m)	
10	2.87–2.82 (m)	2.83 (m)	
11a	2.59–2.53 (m)	2.58 (dd, 14.0, 8.5 Hz)	
11b	2.59–2.53 (m)	2.54 (dd, 14.0, 8.5 Hz)	
12	5.45 (t, 8.4 Hz)	5.44 (t, 8.5 Hz)	+0.01
14	6.40 (s)	6.39 (s)	+0.01
15	7.46 (s)	7.46 (s)	0.00
16	7.45 (t, 1.6 Hz)	7.45 (s)	0.00
17	1.04 (d, 6.4 Hz)	1.04 (d, 7.0 Hz)	0.00
-OH	3.71 (br s)	3.37 (br s)	+0.34

Table S10 ^{13}C NMR Data of (+)-Teucvisin C (**5**)

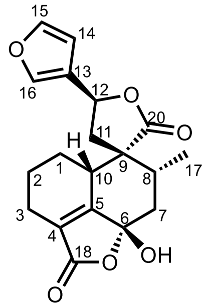

(+)-teucvisin C (**5**)

Carbon No.	This work (CDCl$_3$, 100 MHz)	Natural (ref. 12) (CDCl$_3$, 125 MHz)	Δδ (ppm)
1	24.5	24.7	−0.2
2	21.4	21.6	−0.2
3	19.4	19.6	−0.2
4	128.4	128.7	−0.3
5	159.8	159.7	+0.1
6	102.2	102.1	+0.1
7	39.8	40.1	−0.3
8	35.8	36.0	−0.2
9	54.0	54.2	−0.2
10	40.2	40.4	−0.2
11	40.6	40.8	−0.2
12	72.0	72.1	−0.1
13	124.8	125.1	−0.3
14	108.0	108.1	−0.1
15	144.3	144.4	−0.1
16	139.6	139.7	−0.1
17	16.6	16.8	−0.2
18	170.8	170.5	+0.3
20	175.8	175.8	0.0

5) (+)-Teucrin A (6)

Natural product: $[\alpha]_D^{20}$ = +190.0 (c = 0.38, pyridine);[13]

Synthetic product: $[\alpha]_D^{31}$ = +91.03 (c = 0.11, $CHCl_3$).

Table S11 ^{1}H NMR Data of (+)-Teucrin A (**6**)

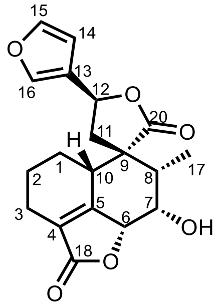

(+)-teucrin A (**6**)

**Carben No.**	**This work** (DMSO, 400 MHz)	**Natural (ref. 14)** (DMSO, 400 MHz)	Δδ (ppm)
1a	1.56–1.45 (m)		
1b	2.03–1.86 (m)	1.90–1.88 (m)	
2a	2.03–1.86 (m)	1.98–1.93 (m)	
2b	2.12–2.08 (m)	2.16–2.12 (m)	
3a	1.31–1.27 (m)		
3b	2.37–2.30 (m)	2.34–2.33 (m)	
6	5.01–5.00 (m)	5.02 (m)	–0.01
7	4.02 (ddd, 10.8, 4.4, 2.4)	4.03 (dd, 10.8, 2.4)	–0.01
8	2.24–2.16 (m)	2.26–2.19 (m)	
10	3.09–3.05 (m)	3.08 (m)	
11a	2.73 (dd, 14.0, 8.0)	2.73 (dd, 14.0, 8.0)	0.00
11b	2.63 (dd, 14.0, 9.6)	2.64 (dd, 14.0, 9.6)	–0.01
12	5.78 (t, 8.4)	5.79 (t, 8.4)	–0.01
14	6.59 (d, 0.8)	6.60 (d, 0.8)	–0.01
15	7.75 (s)	7.76 (s)	–0.01
16	7.90 (s)	7.92 (s)	–0.02
17	1.12 (d, 7.8)		
-OH	4.54 (d, 10.8)	4.57 (d, 10.8)	–0.03

Table S12 ^{13}C NMR Data of (+)-Teucrin A (**6**)

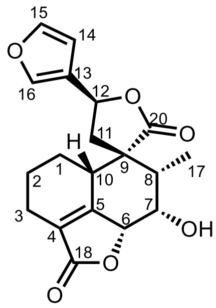

(+)-teucrin A (**6**)

Carben No.	This work (DMSO, 100 MHz)	Natural (ref. 14) (DMSO, 100 MHz)	Δδ (ppm)
1	21.2	21.7	–0.5
2	19.2	19.6	–0.4
3	24.1	24.6	–0.5
4	126.6	127.1	–0.5
5	159.3	159.8	–0.5
6	80.2	80.7	–0.5
7	71.4	71.9	–0.5
8	37.5	38.0	–0.5
9	56.2	56.7	–0.5
10	40.9	41.3	–0.4
11	41.0	41.4	–0.4
12	74.4	74.9	–0.5
13	123.8	124.3	–0.5
14	108.7	109.2	–0.5
15	144.7	145.2	–0.5
16	141.3	141.8	–0.5
17	13.4	13.9	–0.5
18	172.6	173.2	–0.6
20	180.5	181.1	–0.6

6) (+)-Cracroson E (7)

Natural product: $[\alpha]_D^{25}$ = +112 (c = 1.0, MeOH);[15]

Synthetic product: $[\alpha]_D^{23}$ = +95.79 (c = 0.20, MeOH).

Table S13 ^{1}H NMR Data of (+)-Cracroson E (**7**)

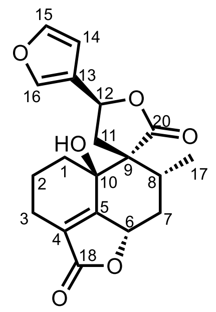

(+)-cracroson E (**7**)

Carbon No.	This work (CDCl$_3$, 400 MHz)	Natural (ref. 15) (CDCl$_3$, 400 MHz)	Δδ (ppm)
1a	2.14–2.03 (m)	2.08 (m)	
1b	1.68–1.62 (m)	1.67 (m)	
2a	2.35–2.29 (m)	2.25 (m)	
2b	2.14–2.03 (m)	2.06 (m)	
3a	1.98–1.93 (m)	1.94 (m)	
3b	1.76–1.62 (m)	1.68 (m)	
6	5.09–5.03 (m)	5.07 (m)	
7a	2.27–2.17 (m)	2.20 (m)	
7b	2.27–2.17 (m)	2.24 (m)	
8	2.27–2.17 (m)	2.18 (m)	
11a	3.23 (dd, 14.8, 8.8 Hz)	3.24 (dd, 14.8, 8.6 Hz)	−0.01
11b	2.26 (dd, 14.8, 8.4 Hz)	2.28 (m)	−0.02
12	5.44 (t, 8.4 Hz)	5.44 (t, 8.6 Hz)	0.00
14	6.40 (s)	6.40 (dd, 1.7, 0.8 Hz)	0.00
15	7.45 (t, 1.6 Hz)	7.45 (t, 1.7 Hz)	0.00
16	7.46 (s)	7.46 (m)	0.00
17	1.02 (d, 6.0 Hz)	1.01 (d, 6.5 Hz)	+0.01
-OH	2.59 (br s)		

Table S14 ^{13}C NMR Data of (+)-Cracroson E (**7**)

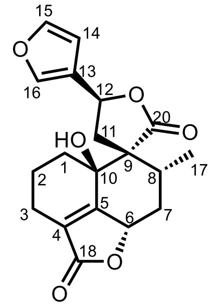

(+)-cracroson E (**7**)

**Carbon No.**	**This work** (CDCl3, 100 MHz)	**Natural (ref. 15)** (CDCl3, 100 MHz)	Δδ (ppm)
1	33.8	33.6	+0.2
2	20.0	19.8	+0.2
3	19.4	19.2	+0.2
4	128.6	128.1	+0.5
5	160.9	161.4	−0.5
6	77.1	77.3	−0.2
7	35.3	35.3	0.0
8	32.5	32.3	+0.2
9	58.9	58.9	0.0
10	69.4	69.2	+0.2
11	34.8	34.6	+0.2
12	72.2	72.2	0.0
13	125.0	124.9	+0.1
14	108.0	107.9	+0.1
15	144.2	144.1	+0.1
16	139.5	139.4	+0.1
17	17.2	17.1	+0.1
18	172.8	173.2	−0.4
20	175.5	175.7	−0.2

7) (+)-2-Hydroxyteuscorolide A (8)

Natural product: $[\alpha]_D^{25}$ = +20.8 (*c* = 0.260, $CHCl_3$:C_5H_5N = 9:1);[16]

Synthetic product: $[\alpha]_D^{25}$ = +10.40 (*c* = 0.20, $CHCl_3$:C_5H_5N = 9:1).

Table S15 ^{1}H NMR Data of (+)-2-Hydroxyteuscorolide A (**8**)

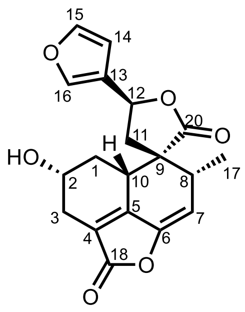

(+)-2-hydroxyteuscorolide A (**8**)

**Carbon No.**	**This work** (pyridine-d$_5$, 400 MHz)	**Natural (ref. 16)** (pyridine-d$_5$, 90 MHz)	Δδ (ppm)
1a	1.86 (dd, 22.8, 11.6 Hz)	1.77 (t, 10.0 Hz)	+0.09
1b			
2	4.43–4.34 (m)	4.33 (m)	
3a			
3b			
7	5.46 (d, 2.0 Hz)	5.40 (d, 1.5 Hz)	+0.06
8			
10			
11a	2.69 (dd, 14.0, 8.0 Hz)	2.70 (dd, 13.5, 8.5 Hz)	−0.01
11b	2.55 (dd, 14.0, 9.2 Hz)	2.50 (dd, 13.5, 8.5 Hz)	+0.05
12	5.62 (t, 8.4 Hz)	5.53 (t, 8.5 Hz)	+0.09
14	6.61 (s)	6.55 (m)	+0.06
15	7.71 (t, 2.0 Hz)	7.63 (t, 1.5 Hz)	+0.08
16	7.81 (s)	7.73 (m)	+0.08
17	1.23 (d, 7.2 Hz)	1.23 (d, 7.5 Hz)	0.00

Table S16 ^{13}C NMR Data of (+)-2-Hydroxyteuscorolide A (**8**)

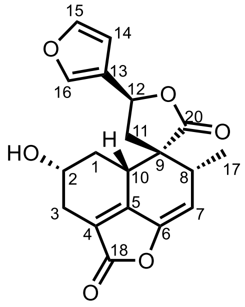

(+)-2-hydroxyteuscorolide A (**8**)

**Carbon No.**	**This work** (pyridine-d$_5$, 100 MHz)	**Natural (ref. 16)** (pyridine-d$_5$, 20.15 MHz)	Δδ (ppm)
1	34.6	34.7	−0.1
2	67.5	67.7	−0.2
3	30.1	30.3	−0.2
4	122.8	123.0	−0.2
5	151.3	151.4	−0.1
6	147.2	147.3	−0.1
7	109.1	109.2	−0.1
8	37.7	37.9	−0.2
9	53.7	53.9	−0.2
10	40.1	40.3	−0.2
11	39.0	39.2	−0.2
12	71.7	72.0	−0.3
13	125.5	125.7	−0.2
14	108.8	108.9	−0.1
15	144.7	144.8	−0.1
16	140.6	140.7	−0.1
17	16.7	16.9	−0.2
18	169.1	169.1	0.0
20	175.1	175.2	−0.1

10. X-ray Crystallographic Data of 25, (+)-Cracroson E (7) and 38

1) X-ray structure of 25 (CCDC 2274928)

The sample of **25** was prepared by recrystallization in petroleum ether/ethyl acetate.

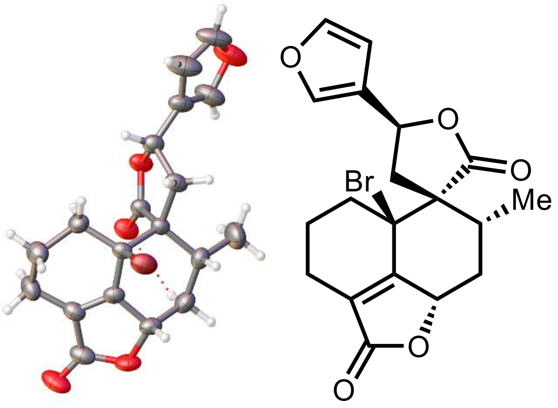

Table 1. Crystal data and structure refinement for 25.

Identification code	mo_d8v23026_0m	
Empirical formula	$C_{19}H_{19}BrO_5$	
Formula weight	407.25	
Temperature	213(2) K	
Wavelength	0.71073 Å	
Crystal system	Monoclinic	
Space group	P 21	
Unit cell dimensions	a = 6.7480(7) Å	α = 90° .
	b = 13.0022(13) Å	β = 96.872(3)° .
	c = 10.0405(11) Å	γ = 90° .
Volume	874.61(16) $Å^3$	
Z	2	
Density (calculated)	1.546 Mg/m^3	
Absorption coefficient	2.377 mm^{-1}	
F(000)	416	
Crystal size	0.200 x 0.150 x 0.100 mm^3	
Theta range for data collection	2.575 to 25.999° .	
Index ranges	-8<=h<=8, -16<=k<=16, -12<=l<=12	
Reflections collected	15392	
Independent reflections	3412 [R(int) = 0.0553]	
Completeness to theta = 25.242°	99.90%	
Absorption correction	Semi-empirical from equivalents	
Max. and min. transmission	0.7456 and 0.4722	
Refinement method	Full-matrix least-squares on F^2	
Data / restraints / parameters	3412 / 1 / 228	
Goodness-of-fit on F2	1.017	
Final R indices [I>2sigma(I)]	R1 = 0.0314, wR2 = 0.0782	
R indices (all data)	R1 = 0.0366, wR2 = 0.0816	
Absolute structure parameter	0.076(6)	
Extinction coefficient	0.015(3)	
Largest diff. peak and hole	0.505 and -0.319 e.$Å^{-3}$	

Table 2. Atomic coordinates (x 10^4) and equivalent isotropic displacement parameters (Å2x 10^3) for 26. U(eq) is defined as one third of the trace of the orthogonalized Uij tensor.

Atom	x	y	z	U(eq)
Br(1)	7396(1)	6761(1)	4032(1)	46(1)
O(1)	7846(6)	3179(3)	7616(4)	62(1)
O(2)	8837(5)	3494(3)	5598(4)	50(1)
O(3)	3237(5)	3574(3)	3245(3)	45(1)
O(4)	1603(5)	4719(3)	1913(3)	42(1)
O(5)	-400(7)	5573(4)	-2042(4)	75(1)
C(1)	7650(7)	3673(4)	6583(5)	45(1)
C(2)	6239(7)	4502(4)	6157(5)	39(1)
C(3)	4628(9)	4930(4)	6871(5)	50(1)
C(4)	4061(9)	5986(4)	6270(5)	50(1)
C(5)	3679(7)	5945(4)	4740(5)	42(1)
C(6)	5499(6)	5575(3)	4099(4)	31(1)
C(7)	6645(6)	4813(3)	4960(4)	33(1)
C(8)	8336(6)	4238(4)	4525(5)	40(1)
C(9)	7725(7)	3751(4)	3182(5)	45(1)
C(10)	6786(7)	4542(4)	2163(5)	41(1)
C(11)	4990(6)	5137(3)	2657(4)	32(1)
C(12)	3251(6)	4381(3)	2671(4)	35(1)
C(13)	4102(7)	5960(4)	1643(5)	40(1)
C(14)	1848(6)	5784(4)	1472(5)	38(1)
C(15)	817(7)	5890(4)	91(5)	45(1)
C(16)	579(10)	5176(5)	-882(6)	64(2)
C(17)	-765(9)	6559(5)	-1771(6)	70(2)
C(18)	-40(8)	6783(6)	-499(6)	71(2)
C(19)	6195(11)	3993(6)	808(5)	66(2)

Table 3. Bond lengths [Å] and angles [°] for 25.

Br(1)-C(6)	2.010(4)
O(1)-C(1)	1.214(6)
O(2)-C(1)	1.365(7)
O(2)-C(8)	1.458(5)
O(3)-C(12)	1.197(6)
O(4)-C(12)	1.344(5)
O(4)-C(14)	1.470(5)
O(5)-C(17)	1.338(8)
O(5)-C(16)	1.369(7)
C(1)-C(2)	1.468(7)
C(2)-C(7)	1.327(6)
C(2)-C(3)	1.481(7)
C(3)-C(4)	1.529(7)
C(3)-H(3A)	0.98
C(3)-H(3B)	0.98
C(4)-C(5)	1.529(7)

C(4)-H(4A)	0.98
C(4)-H(4B)	0.98
C(5)-C(6)	1.530(6)
C(5)-H(5A)	0.98
C(5)-H(5B)	0.98
C(6)-C(7)	1.472(6)
C(6)-C(11)	1.556(6)
C(7)-C(8)	1.473(6)
C(8)-C(9)	1.502(7)
C(8)-H(8)	0.99
C(9)-C(10)	1.534(7)
C(9)-H(9A)	0.98
C(9)-H(9B)	0.98
C(10)-C(19)	1.546(7)
C(10)-C(11)	1.567(6)
C(10)-H(10)	0.99
C(11)-C(12)	1.532(6)
C(11)-C(13)	1.547(6)
C(13)-C(14)	1.527(6)
C(13)-H(13A)	0.98
C(13)-H(13B)	0.98
C(14)-C(15)	1.482(7)
C(14)-H(14)	0.99
C(15)-C(16)	1.343(8)
C(15)-C(18)	1.397(9)
C(16)-H(16)	0.94
C(17)-C(18)	1.344(8)
C(17)-H(17)	0.94
C(18)-H(18)	0.94
C(19)-H(19A)	0.97
C(19)-H(19B)	0.97
C(19)-H(19C)	0.97
C(1)-O(2)-C(8)	108.5(3)
C(12)-O(4)-C(14)	111.3(3)
C(17)-O(5)-C(16)	105.6(5)
O(1)-C(1)-O(2)	121.1(5)
O(1)-C(1)-C(2)	129.6(5)
O(2)-C(1)-C(2)	109.3(4)
C(7)-C(2)-C(1)	106.9(4)
C(7)-C(2)-C(3)	124.5(4)
C(1)-C(2)-C(3)	128.6(4)
C(2)-C(3)-C(4)	108.1(4)
C(2)-C(3)-H(3A)	110.1
C(4)-C(3)-H(3A)	110.1
C(2)-C(3)-H(3B)	110.1
C(4)-C(3)-H(3B)	110.1
H(3A)-C(3)-H(3B)	108.4
C(5)-C(4)-C(3)	111.7(4)

C(5)-C(4)-H(4A)	109.3
C(3)-C(4)-H(4A)	109.3
C(5)-C(4)-H(4B)	109.3
C(3)-C(4)-H(4B)	109.3
H(4A)-C(4)-H(4B)	108
C(4)-C(5)-C(6)	112.7(4)
C(4)-C(5)-H(5A)	109.1
C(6)-C(5)-H(5A)	109.1
C(4)-C(5)-H(5B)	109.1
C(6)-C(5)-H(5B)	109.1
H(5A)-C(5)-H(5B)	107.8
C(7)-C(6)-C(5)	110.9(4)
C(7)-C(6)-C(11)	110.0(4)
C(5)-C(6)-C(11)	114.0(3)
C(7)-C(6)-Br(1)	104.2(3)
C(5)-C(6)-Br(1)	108.6(3)
C(11)-C(6)-Br(1)	108.7(3)
C(2)-C(7)-C(6)	125.9(4)
C(2)-C(7)-C(8)	111.4(4)
C(6)-C(7)-C(8)	122.5(4)
O(2)-C(8)-C(7)	103.7(4)
O(2)-C(8)-C(9)	113.4(4)
C(7)-C(8)-C(9)	109.9(4)
O(2)-C(8)-H(8)	109.9
C(7)-C(8)-H(8)	109.9
C(9)-C(8)-H(8)	109.9
C(8)-C(9)-C(10)	111.2(4)
C(8)-C(9)-H(9A)	109.4
C(10)-C(9)-H(9A)	109.4
C(8)-C(9)-H(9B)	109.4
C(10)-C(9)-H(9B)	109.4
H(9A)-C(9)-H(9B)	108
C(9)-C(10)-C(19)	108.6(4)
C(9)-C(10)-C(11)	113.2(4)
C(19)-C(10)-C(11)	112.2(4)
C(9)-C(10)-H(10)	107.5
C(19)-C(10)-H(10)	107.5
C(11)-C(10)-H(10)	107.5
C(12)-C(11)-C(13)	102.3(3)
C(12)-C(11)-C(6)	108.1(3)
C(13)-C(11)-C(6)	112.6(4)
C(12)-C(11)-C(10)	108.0(4)
C(13)-C(11)-C(10)	112.7(4)
C(6)-C(11)-C(10)	112.5(3)
O(3)-C(12)-O(4)	120.3(4)
O(3)-C(12)-C(11)	128.1(4)
O(4)-C(12)-C(11)	111.6(4)
C(14)-C(13)-C(11)	105.9(4)
C(14)-C(13)-H(13A)	110.6

C(11)-C(13)-H(13A)	110.6
C(14)-C(13)-H(13B)	110.6
C(11)-C(13)-H(13B)	110.6
H(13A)-C(13)-H(13B)	108.7
O(4)-C(14)-C(15)	108.2(4)
O(4)-C(14)-C(13)	104.7(4)
C(15)-C(14)-C(13)	116.2(4)
O(4)-C(14)-H(14)	109.2
C(15)-C(14)-H(14)	109.2
C(13)-C(14)-H(14)	109.2
C(16)-C(15)-C(18)	105.0(5)
C(16)-C(15)-C(14)	128.2(5)
C(18)-C(15)-C(14)	126.7(5)
C(15)-C(16)-O(5)	111.2(6)
C(15)-C(16)-H(16)	124.4
O(5)-C(16)-H(16)	124.4
O(5)-C(17)-C(18)	110.3(6)
O(5)-C(17)-H(17)	124.9
C(18)-C(17)-H(17)	124.9
C(17)-C(18)-C(15)	107.9(7)
C(17)-C(18)-H(18)	126.1
C(15)-C(18)-H(18)	126.1
C(10)-C(19)-H(19A)	109.5
C(10)-C(19)-H(19B)	109.5
H(19A)-C(19)-H(19B)	109.5
C(10)-C(19)-H(19C)	109.5
H(19A)-C(19)-H(19C)	109.5
H(19B)-C(19)-H(19C)	109.5

Table 4. Anisotropic displacement parameters ($Å^2$x 10^3) for 25. The anisotropic displacement factor exponent takes the form: $-2p^2[h^2 a^{*2}U^{11} + ... + 2 h k a^* b^* U^{12}]$

	U^{11}	U^{22}	U^{33}	U^{23}	U^{13}	U^{12}
Br(1)	46(1)	35(1)	54(1)	5(1)	-8(1)	-13(1)
O(1)	90(3)	44(2)	46(2)	12(2)	-14(2)	5(2)
O(2)	45(2)	43(2)	58(2)	11(2)	-8(2)	12(2)
O(3)	46(2)	39(2)	46(2)	9(2)	-3(1)	-13(1)
O(4)	33(2)	48(2)	44(2)	13(2)	-5(1)	-12(1)
O(5)	81(3)	74(3)	60(2)	16(2)	-30(2)	-20(2)
C(1)	54(3)	33(2)	45(3)	5(2)	-10(2)	-1(2)
C(2)	46(2)	34(2)	36(2)	3(2)	-4(2)	-2(2)
C(3)	71(3)	46(3)	34(3)	-1(2)	11(2)	4(2)
C(4)	60(3)	43(3)	45(3)	-4(2)	7(2)	6(2)
C(5)	39(2)	41(3)	44(3)	0(2)	4(2)	6(2)
C(6)	31(2)	28(2)	32(2)	2(2)	-3(2)	-5(2)
C(7)	29(2)	30(2)	40(2)	-2(2)	-4(2)	-3(2)
C(8)	31(2)	36(2)	49(3)	8(2)	-1(2)	1(2)
C(9)	39(2)	46(3)	52(3)	-4(2)	11(2)	6(2)
C(10)	37(2)	44(3)	43(3)	-2(2)	12(2)	-1(2)

C(11)	30(2)	34(2)	31(2)	2(2)	-1(2)	-4(2)
C(12)	32(2)	36(2)	34(2)	1(2)	-1(2)	-7(2)
C(13)	38(2)	41(2)	38(2)	9(2)	-5(2)	-10(2)
C(14)	33(2)	40(2)	41(3)	3(2)	-1(2)	-6(2)
C(15)	34(2)	46(3)	51(3)	13(2)	-8(2)	-7(2)
C(16)	75(4)	60(4)	50(3)	6(3)	-23(3)	-3(3)
C(17)	63(3)	57(4)	81(4)	29(3)	-35(3)	-11(3)
C(18)	71(3)	49(3)	84(4)	17(4)	-29(3)	-7(4)
C(19)	82(4)	78(4)	41(3)	-8(3)	12(3)	11(3)

Table 5. Hydrogen coordinates (x 10^4) and isotropic displacement parameters (Å^{2}x 10^3) for 25.

	x	y	z	U(eq)
H(3A)	5089	4997	7830	60
H(3B)	3466	4472	6765	60
H(4A)	2857	6234	6626	60
H(4B)	5141	6474	6539	60
H(5A)	3302	6632	4397	50
H(5B)	2556	5481	4475	50
H(8)	9477	4708	4467	47
H(9A)	6761	3200	3281	54
H(9B)	8897	3443	2849	54
H(10)	7828	5055	2026	49
H(13A)	4422	6652	1988	47
H(13B)	4636	5879	784	47
H(14)	1226	6256	2075	46
H(16)	1026	4493	-780	77
H(17)	-1432	7026	-2382	84
H(18)	-99	7427	-80	85
H(19A)	5220	3462	921	100
H(19B)	7370	3684	504	100
H(19C)	5625	4489	149	100

Table 6. Torsion angles [°] for 25.

C(8)-O(2)-C(1)-O(1)	-176.7(5)
C(8)-O(2)-C(1)-C(2)	3.4(5)
O(1)-C(1)-C(2)-C(7)	178.5(5)
O(2)-C(1)-C(2)-C(7)	-1.6(5)
O(1)-C(1)-C(2)-C(3)	-2.3(9)
O(2)-C(1)-C(2)-C(3)	177.6(5)
C(7)-C(2)-C(3)-C(4)	-19.7(7)
C(1)-C(2)-C(3)-C(4)	161.2(5)
C(2)-C(3)-C(4)-C(5)	49.9(6)
C(3)-C(4)-C(5)-C(6)	-59.6(6)
C(4)-C(5)-C(6)-C(7)	33.6(5)
C(4)-C(5)-C(6)-C(11)	158.4(4)
C(4)-C(5)-C(6)-Br(1)	-80.3(4)
C(1)-C(2)-C(7)-C(6)	174.7(4)
C(3)-C(2)-C(7)-C(6)	-4.5(7)

C(1)-C(2)-C(7)-C(8)	-0.9(5)
C(3)-C(2)-C(7)-C(8)	179.8(5)
C(5)-C(6)-C(7)-C(2)	-2.5(6)
C(11)-C(6)-C(7)-C(2)	-129.5(4)
Br(1)-C(6)-C(7)-C(2)	114.2(4)
C(5)-C(6)-C(7)-C(8)	172.7(4)
C(11)-C(6)-C(7)-C(8)	45.7(5)
Br(1)-C(6)-C(7)-C(8)	-70.6(4)
C(1)-O(2)-C(8)-C(7)	-3.7(4)
C(1)-O(2)-C(8)-C(9)	-123.0(4)
C(2)-C(7)-C(8)-O(2)	2.9(5)
C(6)-C(7)-C(8)-O(2)	-173.0(4)
C(2)-C(7)-C(8)-C(9)	124.4(4)
C(6)-C(7)-C(8)-C(9)	-51.4(5)
O(2)-C(8)-C(9)-C(10)	167.6(4)
C(7)-C(8)-C(9)-C(10)	52.1(5)
C(8)-C(9)-C(10)-C(19)	179.9(4)
C(8)-C(9)-C(10)-C(11)	-54.7(5)
C(7)-C(6)-C(11)-C(12)	77.4(4)
C(5)-C(6)-C(11)-C(12)	-47.8(5)
Br(1)-C(6)-C(11)-C(12)	-169.2(3)
C(7)-C(6)-C(11)-C(13)	-170.4(3)
C(5)-C(6)-C(11)-C(13)	64.4(5)
Br(1)-C(6)-C(11)-C(13)	-56.9(4)
C(7)-C(6)-C(11)-C(10)	-41.7(5)
C(5)-C(6)-C(11)-C(10)	-166.9(4)
Br(1)-C(6)-C(11)-C(10)	71.8(4)
C(9)-C(10)-C(11)-C(12)	-69.7(5)
C(19)-C(10)-C(11)-C(12)	53.6(5)
C(9)-C(10)-C(11)-C(13)	178.0(4)
C(19)-C(10)-C(11)-C(13)	-58.6(5)
C(9)-C(10)-C(11)-C(6)	49.4(5)
C(19)-C(10)-C(11)-C(6)	172.8(4)
C(14)-O(4)-C(12)-O(3)	171.3(4)
C(14)-O(4)-C(12)-C(11)	-9.3(5)
C(13)-C(11)-C(12)-O(3)	175.0(5)
C(6)-C(11)-C(12)-O(3)	-66.0(6)
C(10)-C(11)-C(12)-O(3)	55.9(6)
C(13)-C(11)-C(12)-O(4)	-4.3(5)
C(6)-C(11)-C(12)-O(4)	114.7(4)
C(10)-C(11)-C(12)-O(4)	-123.4(4)
C(12)-C(11)-C(13)-C(14)	15.4(5)
C(6)-C(11)-C(13)-C(14)	-100.4(4)
C(10)-C(11)-C(13)-C(14)	131.1(4)
C(12)-O(4)-C(14)-C(15)	143.5(4)
C(12)-O(4)-C(14)-C(13)	19.0(5)
C(11)-C(13)-C(14)-O(4)	-20.7(5)
C(11)-C(13)-C(14)-C(15)	-140.0(4)
O(4)-C(14)-C(15)-C(16)	-31.9(7)

C(13)-C(14)-C(15)-C(16)	85.3(7)
O(4)-C(14)-C(15)-C(18)	151.4(5)
C(13)-C(14)-C(15)-C(18)	-91.3(6)
C(18)-C(15)-C(16)-O(5)	-0.7(7)
C(14)-C(15)-C(16)-O(5)	-177.9(5)
C(17)-O(5)-C(16)-C(15)	0.2(7)
C(16)-O(5)-C(17)-C(18)	0.4(7)
O(5)-C(17)-C(18)-C(15)	-0.8(7)
C(16)-C(15)-C(18)-C(17)	0.9(7)
C(14)-C(15)-C(18)-C(17)	178.1(5)

2) X-ray structure of (+)-Cracroson E (7) (CCDC 2279407)

The sample of (+)-cracroson E (**7**) was prepared by recrystallization in methanol/water.

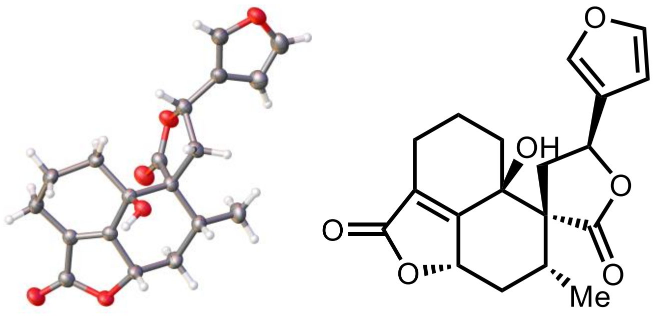

Table 1 Crystal data and structure refinement for (+)-cracroson E (7).

Identification code	exp_3388
Empirical formula	$C_{19}H_{20}O_6$
Formula weight	344.35
Temperature/K	172.95(13)
Crystal system	orthorhombic
Space group	$P2_12_12_1$
a/Å	6.68840(10)
b/Å	7.0394(2)
c/Å	35.4914(7)
α/°	90
β/°	90
γ/°	90
Volume/Å^3	1671.02(6)
Z	4
ρ_{calc}g/cm^3	1.369
μ/mm^{-1}	0.849
F(000)	728.0
Crystal size/mm^3	0.36 × 0.14 × 0.12
Radiation	Cu Kα (λ = 1.54184)
2Θ range for data collection/°	9.968 to 134.136
Index ranges	$-7 \leq h \leq 7$, $-8 \leq k \leq 8$, $-42 \leq l \leq 42$
Reflections collected	35120
Independent reflections	2978 [R_{int} = 0.1301, R_{sigma} = 0.0474]
Data/restraints/parameters	2978/0/228
Goodness-of-fit on F^2	1.047
Final R indexes [I>=2σ (I)]	R_1 = 0.0340, wR_2 = 0.0857
Final R indexes [all data]	R_1 = 0.0358, wR_2 = 0.0870
Largest diff. peak/hole / e Å^{-3}	0.13/-0.13
Flack parameter	-0.05(12)

Table 2 Fractional Atomic Coordinates (×10^4) and Equivalent Isotropic Displacement Parameters (Å^2×10^3) for (+)-cracroson E (7). U_{eq} is defined as 1/3 of the trace of the orthogonalised U_{IJ} tensor.

Atom	*x*	*y*	*z*	U(eq)
O1	4030(3)	3649(3)	4569.9(5)	43.8(5)
O2	6230(2)	5611(3)	5699.7(4)	35.8(4)
O3	6817(2)	7431(3)	6198.1(4)	38.1(4)
O4	2828(2)	2705(2)	6594.0(4)	27.1(4)
O5	7610(2)	6635(3)	7637.4(4)	38.2(4)
O6	4748(2)	7251(2)	7329.6(4)	31.1(4)
C1	3289(4)	5415(4)	4633.5(7)	42.6(6)
C2	3469(4)	5889(4)	4996.8(7)	38.7(6)
C3	4390(3)	4306(4)	5179.3(6)	31.1(5)
C4	4697(4)	3001(4)	4908.6(6)	39.4(6)
C5	4895(4)	4068(3)	5586.4(6)	31.3(5)
C6	3133(3)	4186(3)	5860.3(6)	29.1(5)
C7	3930(3)	5269(3)	6206.3(6)	24.4(5)
C8	5797(3)	6240(3)	6049.9(6)	28.7(5)
C9	2421(3)	6790(3)	6353.0(6)	28.3(5)
C10	3108(4)	7829(3)	6713.7(6)	31.2(5)
C11	3645(3)	6417(3)	7017.6(6)	25.7(5)
C12	6495(3)	6276(3)	7376.0(6)	28.8(5)
C13	6685(3)	4869(3)	7074.6(6)	25.3(5)
C14	5036(3)	4961(3)	6870.0(5)	22.5(4)
C15	4570(3)	3849(3)	6525.7(6)	23.0(5)
C16	6391(3)	2591(3)	6424.8(6)	28.9(5)
C17	7462(4)	1833(3)	6776.0(6)	32.1(5)
C18	8313(3)	3453(4)	7014.6(6)	31.2(5)
C19	1932(4)	8300(4)	6056.5(7)	38.6(6)

Table 3 Anisotropic Displacement Parameters (Å^2×10^3) for (+)-cracroson E (7). The Anisotropic displacement factor exponent takes the form: $-2\pi^2[h^2a^{*2}U_{11}+2hka^*b^*U_{12}+\ldots]$.

Atom	U_{11}	U_{22}	U_{33}	U_{23}	U_{13}	U_{12}
O1	44.0(10)	61.1(12)	26.3(8)	-1.4(8)	-1.1(7)	2.4(9)
O2	33.1(9)	47.3(11)	27.0(8)	-0.6(7)	1.8(7)	-9.7(8)
O3	37.7(9)	39.8(10)	36.7(9)	0.5(8)	-2.0(7)	-14.7(8)
O4	29.6(8)	26.0(8)	25.6(7)	0.3(6)	-0.3(6)	-6.6(7)
O5	33.4(8)	49.4(11)	31.8(8)	-9.1(8)	-7.8(7)	-5.9(8)
O6	32.4(8)	32.9(8)	28.0(8)	-10.2(6)	-4.7(7)	1.3(7)
C1	39.5(14)	51.6(17)	36.6(13)	13.0(12)	-1.0(11)	-2.2(13)
C2	42.5(14)	36.4(14)	37.3(13)	5.5(10)	1.4(11)	-1.2(11)
C3	29.2(11)	37.9(14)	26.3(11)	1.5(10)	2.3(9)	-0.4(10)
C4	37.1(13)	50.6(16)	30.7(13)	-1.0(11)	-1.9(10)	7.7(12)
C5	33.0(12)	33.1(13)	27.8(11)	0.2(9)	-2.3(9)	-1.2(10)

C6	32.2(12)	30.8(12)	24.4(11)	-0.8(9)	-3.4(9)	-5.3(10)
C7	25.8(10)	22.8(11)	24.5(11)	-0.4(9)	-3.1(8)	-3.4(9)
C8	29.4(11)	29.8(12)	27.0(11)	2.3(9)	-1.9(9)	-3.1(9)
C9	28.0(11)	26.2(12)	30.6(11)	0.3(9)	-3.9(9)	1.8(9)
C10	36.3(13)	22.9(11)	34.3(12)	-2.6(9)	-3.3(10)	4.6(10)
C11	27.0(10)	25.5(11)	24.6(10)	-6.4(8)	-3.4(8)	0.7(9)
C12	28.2(11)	31.3(12)	26.9(11)	0.0(9)	-0.7(9)	-5.0(9)
C13	24.8(10)	25.9(11)	25.2(10)	2.3(9)	1.2(8)	-3.6(9)
C14	23.6(10)	20.8(11)	23.2(10)	2.1(8)	2.6(8)	-3.5(9)
C15	24.1(10)	18.9(10)	26.2(11)	0.0(8)	0.2(8)	-1.4(8)
C16	33.0(12)	26.1(11)	27.7(11)	-2.1(9)	1.8(9)	5.7(10)
C17	31.7(11)	30.1(12)	34.4(12)	-0.5(10)	-0.9(9)	8.9(10)
C18	24.2(10)	37.7(13)	31.7(11)	-0.3(10)	-1.3(9)	3.1(10)
C19	45.3(14)	32.8(13)	37.7(13)	5.9(11)	-8.3(11)	6.0(11)

Table 4 Bond Lengths for (+)-cracroson E (7).

**Atom**	**Atom**	**Length/Å**	**Atom**	**Atom**	**Length/Å**
O1	C1	1.357(3)	C7	C8	1.528(3)
O1	C4	1.361(3)	C7	C9	1.561(3)
O2	C5	1.462(3)	C7	C15	1.571(3)
O2	C8	1.351(3)	C9	C10	1.544(3)
O3	C8	1.202(3)	C9	C19	1.531(3)
O4	C15	1.436(2)	C10	C11	1.510(3)
O5	C12	1.217(3)	C11	C14	1.480(3)
O6	C11	1.454(2)	C12	C13	1.464(3)
O6	C12	1.365(3)	C13	C14	1.322(3)
C1	C2	1.337(4)	C13	C18	1.491(3)
C2	C3	1.429(3)	C14	C15	1.484(3)
C3	C4	1.345(3)	C15	C16	1.548(3)
C3	C5	1.493(3)	C16	C17	1.533(3)
C5	C6	1.530(3)	C17	C18	1.530(3)
C6	C7	1.540(3)			

Table 5 Bond Angles for (+)-cracroson E (7).

**Atom**	**Atom**	**Atom**	**Angle/°**	**Atom**	**Atom**	**Atom**	**Angle/°**
C1	O1	C4	106.26(19)	C19	C9	C10	107.75(19)
C8	O2	C5	111.43(17)	C11	C10	C9	110.57(18)
C12	O6	C11	108.88(16)	O6	C11	C10	113.52(18)
C2	C1	O1	110.8(2)	O6	C11	C14	103.29(16)
C1	C2	C3	106.3(2)	C14	C11	C10	110.64(17)
C2	C3	C5	128.6(2)	O5	C12	O6	120.8(2)
C4	C3	C2	105.9(2)	O5	C12	C13	130.1(2)

C4	C3	C5	125.5(2)	O6	C12	C13	109.04(17)
C3	C4	O1	110.6(2)	C12	C13	C18	128.33(19)
O2	C5	C3	108.72(19)	C14	C13	C12	107.20(19)
O2	C5	C6	104.82(17)	C14	C13	C18	124.3(2)
C3	C5	C6	115.8(2)	C11	C14	C15	121.65(18)
C5	C6	C7	105.45(18)	C13	C14	C11	111.34(19)
C6	C7	C9	112.42(17)	C13	C14	C15	127.0(2)
C6	C7	C15	110.78(17)	O4	C15	C7	104.93(16)
C8	C7	C6	102.41(17)	O4	C15	C14	109.10(16)
C8	C7	C9	110.01(17)	O4	C15	C16	110.88(16)
C8	C7	C15	108.94(17)	C14	C15	C7	108.41(16)
C9	C7	C15	111.83(17)	C14	C15	C16	109.08(18)
O2	C8	C7	111.26(18)	C16	C15	C7	114.28(17)
O3	C8	O2	120.6(2)	C17	C16	C15	112.25(17)
O3	C8	C7	128.1(2)	C18	C17	C16	111.37(19)
C10	C9	C7	114.14(17)	C13	C18	C17	107.80(18)
C19	C9	C7	112.65(18)				

Table 6 Torsion Angles for (+)-cracroson E (7).

A	B	C	D	Angle/°	A	B	C	D	Angle/°
O1	C1	C2	C3	-0.2(3)	C8	C7	C9	C19	53.3(2)
O2	C5	C6	C7	-21.6(2)	C8	C7	C15	O4	-167.16(16)
O4	C15	C16	C17	-84.2(2)	C8	C7	C15	C14	76.4(2)
O5	C12	C13	C14	176.8(2)	C8	C7	C15	C16	-45.5(2)
O5	C12	C13	C18	1.5(4)	C9	C7	C8	O2	-128.61(19)
O6	C11	C14	C13	2.8(2)	C9	C7	C8	O3	50.7(3)
O6	C11	C14	C15	-175.15(17)	C9	C7	C15	O4	71.02(19)
O6	C12	C13	C14	-3.1(2)	C9	C7	C15	C14	-45.4(2)
O6	C12	C13	C18	-178.4(2)	C9	C7	C15	C16	-167.32(18)
C1	O1	C4	C3	0.1(3)	C9	C10	C11	O6	166.00(17)
C1	C2	C3	C4	0.2(3)	C9	C10	C11	C14	50.4(2)
C1	C2	C3	C5	-178.6(2)	C10	C11	C14	C13	124.6(2)
C2	C3	C4	O1	-0.2(3)	C10	C11	C14	C15	-53.3(2)
C2	C3	C5	O2	-58.1(3)	C11	O6	C12	O5	-175.0(2)
C2	C3	C5	C6	59.5(3)	C11	O6	C12	C13	4.9(2)
C3	C5	C6	C7	-141.4(2)	C11	C14	C15	O4	-64.0(2)
C4	O1	C1	C2	0.1(3)	C11	C14	C15	C7	49.8(2)
C4	C3	C5	O2	123.3(3)	C11	C14	C15	C16	174.76(18)
C4	C3	C5	C6	-119.1(3)	C12	O6	C11	C10	-124.52(19)
C5	O2	C8	O3	175.6(2)	C12	O6	C11	C14	-4.7(2)
C5	O2	C8	C7	-5.0(2)	C12	C13	C14	C11	0.0(2)
C5	C3	C4	O1	178.7(2)	C12	C13	C14	C15	177.84(19)
C5	C6	C7	C8	18.4(2)	C12	C13	C18	C17	156.9(2)

C5	C6	C7	C9	136.42(19)	C13	C14	C15	O4	118.4(2)
C5	C6	C7	C15	-97.7(2)	C13	C14	C15	C7	-127.8(2)
C6	C7	C8	O2	-8.9(2)	C13	C14	C15	C16	-2.8(3)
C6	C7	C8	O3	170.4(2)	C14	C13	C18	C17	-17.6(3)
C6	C7	C9	C10	176.60(18)	C14	C15	C16	C17	36.0(2)
C6	C7	C9	C19	-60.1(2)	C15	C7	C8	O2	108.5(2)
C6	C7	C15	O4	-55.2(2)	C15	C7	C8	O3	-72.2(3)
C6	C7	C15	C14	-171.70(17)	C15	C7	C9	C10	51.2(2)
C6	C7	C15	C16	66.4(2)	C15	C7	C9	C19	174.54(18)
C7	C9	C10	C11	-53.3(2)	C15	C16	C17	C18	-62.4(2)
C7	C15	C16	C17	157.52(19)	C16	C17	C18	C13	50.1(2)
C8	O2	C5	C3	141.3(2)	C18	C13	C14	C11	175.55(19)
C8	O2	C5	C6	16.9(2)	C18	C13	C14	C15	-6.7(3)
C8	C7	C9	C10	-70.0(2)	C19	C9	C10	C11	-179.19(19)

Table 7 Hydrogen Atom Coordinates (Å×10^4) and Isotropic Displacement Parameters (Å^2×10^3) for (+)-cracroson E (7).

**Atom**	***x***	***y***	***z***	**U(eq)**
H4	2825.36	2341.84	6819.39	41
H1	2718.04	6207.23	4445.33	51
H2	3065.24	7048.15	5110.79	46
H4A	5297.05	1793.97	4948.3	47
H5	5595.75	2826.98	5622.42	38
H6A	2676.92	2898.6	5932.69	35
H6B	1999.49	4877.14	5744.56	35
H9	1146.55	6115.12	6414.39	34
H10A	4284.24	8630.91	6655.88	37
H10B	2021.93	8667.82	6804.63	37
H11	2404.42	5791.3	7114.23	31
H16A	5935.68	1506.08	6269.41	35
H16B	7345.97	3342.95	6272.56	35
H17A	6509.4	1084.95	6929.42	38
H17B	8562.21	978.22	6697.97	38
H18A	8789.6	2958.42	7259.78	37
H18B	9454.37	4054.15	6883.01	37
H19A	3162.35	8956.54	5982.04	58
H19B	986.57	9220.06	6162.22	58
H19C	1334.58	7689.62	5835.3	58

3) X-ray structure of 38 (CCDC 2275140)

The sample of **38** was prepared by recrystallization in chloroform.

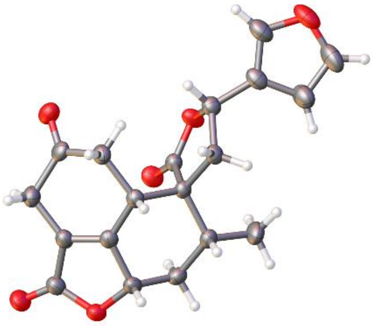

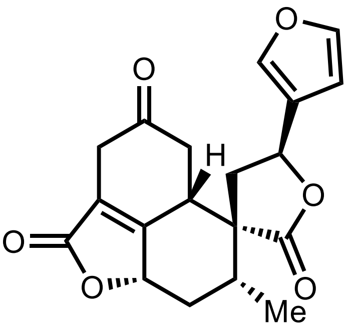

Table 1 Crystal data and structure refinement for 38.

Identification code	exp_2245
Empirical formula	$C_{19}H_{18}O_6$
Formula weight	342.33
Temperature/K	173.00(10)
Crystal system	orthorhombic
Space group	$P2_12_12_1$
a/Å	6.95740(10)
b/Å	10.4076(2)
c/Å	22.6105(4)
α/°	90
β/°	90
γ/°	90
Volume/Å^3	1637.22(5)
Z	4
ρ_{calc}g/cm^3	1.389
μ/mm^{-1}	0.866
F(000)	720.0
Crystal size/mm^3	0.36 × 0.28 × 0.04
Radiation	CuKα (λ = 1.54184)
2Θ range for data collection/°	7.82 to 134.144
Index ranges	$-8 \leq h \leq 8$, $-12 \leq k \leq 12$, $-27 \leq l \leq 27$
Reflections collected	34372
Independent reflections	2918 [R_{int} = 0.0935, R_{sigma} = 0.0373]
Data/restraints/parameters	2918/0/227
Goodness-of-fit on F^2	1.063
Final R indexes [I>=2σ (I)]	R_1 = 0.0328, wR_2 = 0.0835
Final R indexes [all data]	R_1 = 0.0351, wR_2 = 0.0847
Largest diff. peak/hole / e Å^{-3}	0.11/-0.15
Flack parameter	-0.15(14)

Table 2 Fractional Atomic Coordinates (×10^4) and Equivalent Isotropic Displacement Parameters (Å^2×10^3) for 38. U_{eq} is defined as 1/3 of of the trace of the orthogonalised U_{IJ} tensor.

Atom	*x*	*y*	*z*	U(eq)
O1	8793(4)	6175(2)	5549.6(8)	55.3(6)
O2	6376(2)	4321.9(17)	3980.1(7)	37.4(4)
O3	5083(2)	2860.3(16)	3387.3(7)	37.1(4)
O4	-316(2)	2686.3(17)	2447.1(7)	37.9(4)
O5	914(3)	2344.3(19)	1541.1(8)	47.9(5)
O6	6935(3)	5167.6(19)	2219.5(8)	45.5(5)
C1	7278(5)	5533(3)	5784.3(13)	56.4(8)
C2	5992(5)	5227(3)	5367.0(12)	50.0(7)
C3	6739(4)	5710(2)	4823.3(11)	37.1(6)
C4	8417(5)	6278(3)	4960.2(12)	47.9(7)
C5	5925(4)	5604(2)	4216.0(10)	34.0(5)
C6	3739(3)	5718(2)	4160.9(10)	30.5(5)
C7	4905(3)	3836(2)	3666.7(9)	28.5(5)
C8	3116(3)	4674(2)	3721.2(9)	25.7(5)
C9	1355(3)	3884(2)	3948.9(9)	31.8(5)
C10	601(3)	2892(2)	3501.8(10)	34.2(5)
C11	33(3)	3567(2)	2933.1(9)	32.0(5)
C12	923(4)	2952(3)	1999.8(10)	35.6(5)
C13	2184(3)	4008(2)	2181.2(10)	31.8(5)
C14	1657(3)	4358(2)	2724.5(9)	28.9(5)
C15	2615(3)	5303(2)	3112.6(10)	28.3(5)
C16	4295(4)	5959(2)	2780.1(10)	33.5(5)
C17	5207(4)	5196(2)	2285.2(10)	32.2(5)
C18	3877(4)	4528(3)	1857.3(10)	38.6(6)
C19	1752(5)	3205(3)	4534.7(11)	49.1(8)

Table 3 Anisotropic Displacement Parameters (Å^2×10^3) for 38. The Anisotropic displacement factor exponent takes the form: -2π^2[h^{2}a*^{2}U$_{11}$+2hka*b*U$_{12}$+…].

Atom	U_{11}	U_{22}	U_{33}	U_{23}	U_{13}	U_{12}
O1	63.5(14)	51.4(11)	50.9(11)	-12.8(9)	-26.1(10)	-3.2(11)
O2	25.5(8)	47.2(10)	39.6(9)	-14.6(8)	-5.0(7)	5.4(8)
O3	35.8(9)	34.7(8)	40.9(9)	-12.1(7)	-0.1(8)	6.7(8)
O4	28.6(9)	50.2(10)	34.7(9)	-10.6(8)	1.0(7)	-8.9(8)
O5	47.1(11)	59.5(12)	37.1(9)	-15.9(9)	2.1(8)	-12.7(10)
O6	33.6(10)	63.3(12)	39.6(10)	6.4(9)	4.1(8)	-1.4(9)
C1	79(2)	51.0(16)	39.2(15)	0.4(13)	-23.8(15)	1.8(17)
C2	57.2(18)	52.7(16)	40.0(14)	5.7(12)	-12.4(13)	-7.2(15)
C3	40.5(14)	33.4(12)	37.3(13)	-7.4(10)	-9.0(11)	4.6(11)
C4	50.5(17)	49.9(16)	43.5(15)	-13.5(13)	-14.0(13)	-0.6(14)
C5	34.6(13)	35.0(12)	32.4(12)	-5.5(10)	-0.2(10)	-1.8(11)
C6	32.6(13)	31.8(11)	27.2(11)	-5.6(9)	0.1(10)	2.5(10)
C7	26.8(12)	33.7(11)	24.9(10)	0.1(9)	-0.2(9)	-0.1(10)

C8	24.6(11)	28.4(11)	24.2(10)	-2.0(8)	1.1(9)	2.9(9)
C9	28.1(12)	39.1(12)	28.1(11)	-1.4(10)	4.4(9)	0.3(10)
C10	28.2(12)	39.4(12)	34.9(12)	-0.9(10)	3.3(9)	-6.6(10)
C11	22.7(12)	43.2(13)	30.2(11)	-7.3(9)	1.0(10)	-0.9(11)
C12	30.4(12)	43.4(13)	32.9(12)	-7.0(11)	-1.8(10)	-1.7(11)
C13	28.2(12)	40.1(13)	27.0(11)	-3.2(10)	-1.4(9)	1.7(10)
C14	23.9(11)	34.1(12)	28.9(11)	-0.8(9)	-1.0(9)	5.1(10)
C15	27.9(12)	29.6(11)	27.5(11)	-0.7(9)	-1.1(9)	2.9(9)
C16	38.2(14)	33.8(12)	28.6(11)	1.2(10)	-1.1(10)	-3.8(10)
C17	33.0(13)	35.0(11)	28.7(11)	9.5(9)	2.6(10)	-1.7(10)
C18	42.1(14)	47.0(14)	26.7(11)	-3.2(10)	3.9(11)	-5.9(12)
C19	57.8(19)	59.4(18)	30.1(13)	6.6(12)	2.5(12)	-17.8(15)

Table 4 Bond Lengths for 38.

**Atom**	**Atom**	**Length/Å**	**Atom**	**Atom**	**Length/Å**
O1	C1	1.356(4)	C7	C8	1.525(3)
O1	C4	1.362(3)	C8	C9	1.562(3)
O2	C5	1.471(3)	C8	C15	1.563(3)
O2	C7	1.344(3)	C9	C10	1.537(3)
O3	C7	1.203(3)	C9	C19	1.526(3)
O4	C11	1.452(3)	C10	C11	1.518(3)
O4	C12	1.357(3)	C11	C14	1.475(3)
O5	C12	1.215(3)	C12	C13	1.466(3)
O6	C17	1.212(3)	C13	C14	1.333(3)
C1	C2	1.339(4)	C13	C18	1.489(3)
C2	C3	1.426(4)	C14	C15	1.477(3)
C3	C4	1.344(4)	C15	C16	1.549(3)
C3	C5	1.490(3)	C16	C17	1.512(3)
C5	C6	1.531(3)	C17	C18	1.508(3)
C6	C8	1.536(3)			

Table 5 Bond Angles for 38.

**Atom**	**Atom**	**Atom**	**Angle/°**	**Atom**	**Atom**	**Atom**	**Angle/°**
C1	O1	C4	105.8(2)	C19	C9	C8	112.8(2)
C7	O2	C5	111.70(18)	C19	C9	C10	108.8(2)
C12	O4	C11	109.22(17)	C11	C10	C9	109.6(2)
C2	C1	O1	111.2(3)	O4	C11	C10	113.10(19)
C1	C2	C3	106.2(3)	O4	C11	C14	103.81(17)
C2	C3	C5	129.0(2)	C14	C11	C10	109.24(19)
C4	C3	C2	105.8(2)	O4	C12	C13	108.93(19)
C4	C3	C5	125.1(3)	O5	C12	O4	121.8(2)
C3	C4	O1	110.9(3)	O5	C12	C13	129.2(2)

O2	C5	C3	108.7(2)	C12	C13	C18	127.5(2)
O2	C5	C6	104.65(19)	C14	C13	C12	107.3(2)
C3	C5	C6	116.6(2)	C14	C13	C18	124.9(2)
C5	C6	C8	106.17(19)	C11	C14	C15	121.81(19)
O2	C7	C8	111.37(17)	C13	C14	C11	110.7(2)
O3	C7	O2	121.1(2)	C13	C14	C15	127.3(2)
O3	C7	C8	127.5(2)	C14	C15	C8	110.15(18)
C6	C8	C9	112.37(17)	C14	C15	C16	110.24(18)
C6	C8	C15	109.65(18)	C16	C15	C8	116.34(19)
C7	C8	C6	103.09(17)	C17	C16	C15	116.37(19)
C7	C8	C9	111.46(18)	O6	C17	C16	121.3(2)
C7	C8	C15	110.50(17)	O6	C17	C18	121.2(2)
C9	C8	C15	109.62(18)	C18	C17	C16	117.4(2)
C10	C9	C8	113.84(17)	C13	C18	C17	109.70(19)

Table 6 Hydrogen Atom Coordinates (Å×10^4) and Isotropic Displacement Parameters (Å^2×10^3) for 38.

Atom	*x*	*y*	*z*	U(eq)
H1	7149.88	5332.15	6183.06	68
H2	4840.96	4787.68	5420.86	60
H4	9213.71	6686.67	4688.85	58
H5	6527.44	6254.82	3963.25	41
H6A	3384.78	6562.71	4015.46	37
H6B	3130.5	5584.7	4541.93	37
H9	311.58	4496.89	4020.27	38
H10A	1590.81	2260.04	3420.34	41
H10B	-503.43	2449.11	3665.99	41
H11	-1099.64	4108.1	2999.84	38
H15	1666.08	5976.4	3194.06	34
H16A	3828.38	6763.09	2617.04	40
H16B	5286.59	6167.97	3065.9	40
H18A	3452.06	5130.22	1557.29	46
H18B	4555.27	3832.43	1662.17	46
H19A	2788.81	2606.56	4484.32	74
H19B	619.77	2753.43	4659.5	74
H19C	2097.53	3829.43	4828.76	74

11. References

1. J. Peed, I. R. Davies, L. R. Peacock, J. E. Taylor, G. Kociok-Köhn and S. D. Bull, Dihydroxylation-based approach for the asymmetric syntheses of hydroxy-γ-butyrolactones, *J. Org. Chem.*, **2012**, *77*, 543–555.
2. M. Carda and J. A. Marco, Total synthesis of the (−)-mintlactone and (+)-isomintlactone, *Tetrahedron*, **1992**, *48*, 9789–9800.
3. B. Hong, D. Hu, J. Wu, J. Zhang, H. Li, Y. Pan and X. Lei, Divergent total syntheses of (−)-huperzine Q, (+)-lycopladine B, (+)-lycopladine C, and (−)-4-*epi*-lycopladine D, *Chem. Asian J.*, **2017**, *12*, 1557–1567.
4. D. F. Taber, K. Kanai, Q. Jiang and G. Bui, Enantiomerically pure cyclohexenones by Fe-mediated carbonylation of alkenyl cyclopropanes, *J. Am. Chem. Soc.*, **2000**, *122*, 6807–6808.
5. P. C. Roest, N. W. Michel and R. A. Batey, DABO boronate promoted conjugate allylation of α,β-unsaturated aldehydes using copper(II) catalysis, *J. Org. Chem.*, **2016**, *81*, 6774–6778.
6. S. Zhou, C.-R. Chen and H.-M. Gau, Highly enantioselective 3-furylation of ketones using (3-furyl)titanium nucleophile, *Org. Lett.*, **2010**, *12*, 48–51.
7. Z. H. Mbwambo, K. Foubert, M. Chacha, M. C. Kapingu, J. J. Magadula, M. M. Moshi, F. Lemiere, K. Goubitz, J. Fraanje, R. Peschar, A. Vlietinck, S. Apers and L. Pieters, New furanoditerpenoids from *Croton jatrophoides*, *Planta Med.*, **2009**, *75*, 262–267.
8. M. Bruno, C. Fazio, F. Piozzi, B. Rodriguez and M. C. de la Torre, *Neo*-clerodane diterpenoids from *Teucrium corymbosum*, *Phytochemistry*, **1995**, *40*, 1481–1483.
9. I.-C. Chen, Y.-K. Wu, H.-J. Liu and J.-L. Zhu, Total syntheses of (±)-montanin A and (±)-teuscorolide, *Chem. Commun.*, **2008**, 4720–4722.
10. B. Rodriguez and M. L. Jimeno, ^{1}H and ^{13}C NMR spectral assignments and conformational analysis of 14 19-*nor*-neoclerodane diterpenoids, *Magn. Reson. Chem.*, **2004**, *42*, 605–616.
11. M. Qiu, D. Cao, Y. Gao, S. Li, J. Zhu, B. Yang, L. Zhou, Y. Zhou, J. Jin and Z. Zhao, New clerodane diterpenoids from *Croton crassifolius*, *Fitoterapia*, **2016**, *108*, 81–86.
12. H.-W. Lv, J.-G. Luo, M.-D. Zhu, S.-M. Shan and L.-Y. Kong, Teucvisins A–E, five new *neo*-clerodane diterpenes from *Teucrium viscidum*, *Chem. Pharm. Bull.*, **2014**, *62*, 472–476.
13. G. Savona, M. C. García-Alvarez and B. Rodríguez, Dihydroteugin, a *neo*-clerodane diterpenoid from *Teucrium chamaedrys*, *Phytochemistry*, **1982**, *21*, 721–723.
14. M. Elmastas, R. Erenler, B. Isnac, H. Aksit, O. Sen, N. Genc and I. Demirtas, Isolation and identification of a new *neo*-clerodane diterpenoid from *Teucrium chamaedrys* L. *Nat. Prod. Res.*, **2016**, *30*, 299–304.

15. M. Qiu, J. Jin, L. Zhou, W. Zhou, Y. Liu, Q. Tan, D. Cao and Z. Zhao, Diterpenoids from *Croton crassifolius* include a novel skeleton possibly generated via an intramolecular [2+2]-photocycloaddition reaction, *Phytochemistry,* **2018**, *145*, 103–110.
16. J. L. Marco, B. Rodriguez, C. Pascual, G. Savona and F. Piozzi, Teuscorodin, teuscorodonin and 2-hydroxyteuscorolide, *neo*-clerodane diterpenoids from *Teucrium scorodonia*, *Phytochemistry*, **1983**, *22*, 727–731.

12. 2D NMR of (+)-Cracroson A, (+)-Teucvisin C, Compound 30 and (+)-Cracroson E

1) (+)-Cracroson A (4)

COSY

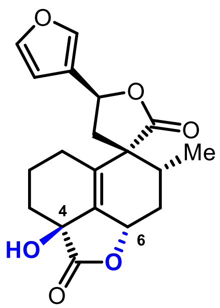

(+)-cracroson A (**4**)

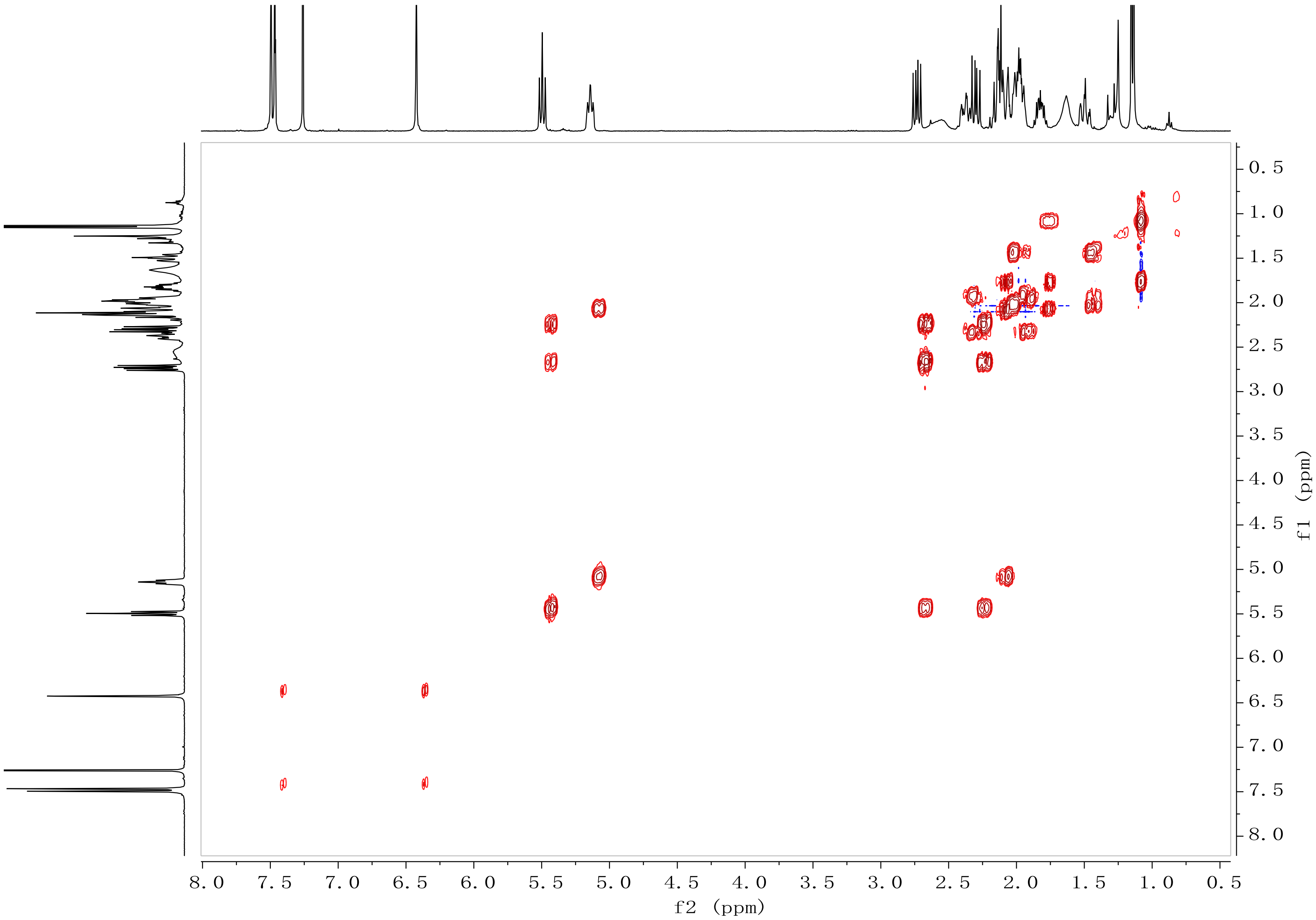

HSQC

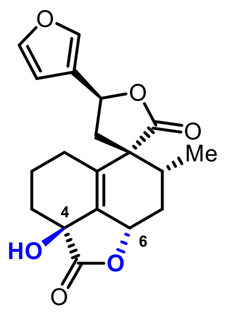

(+)-cracroson A (**4**)

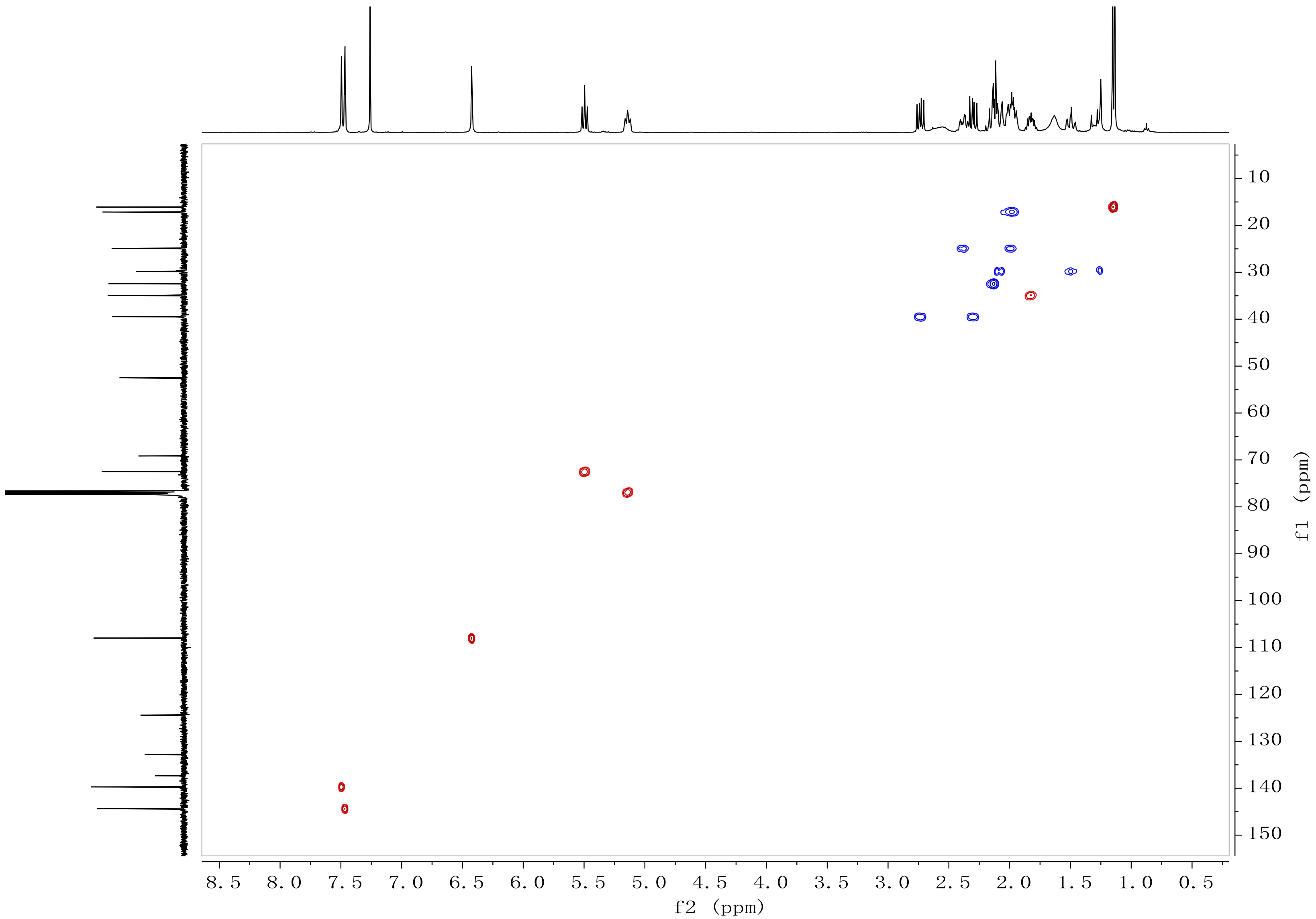

NOESY

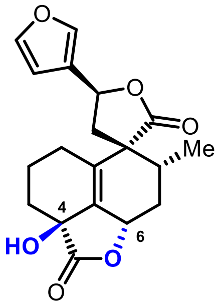

(+)-cracroson A (**4**)

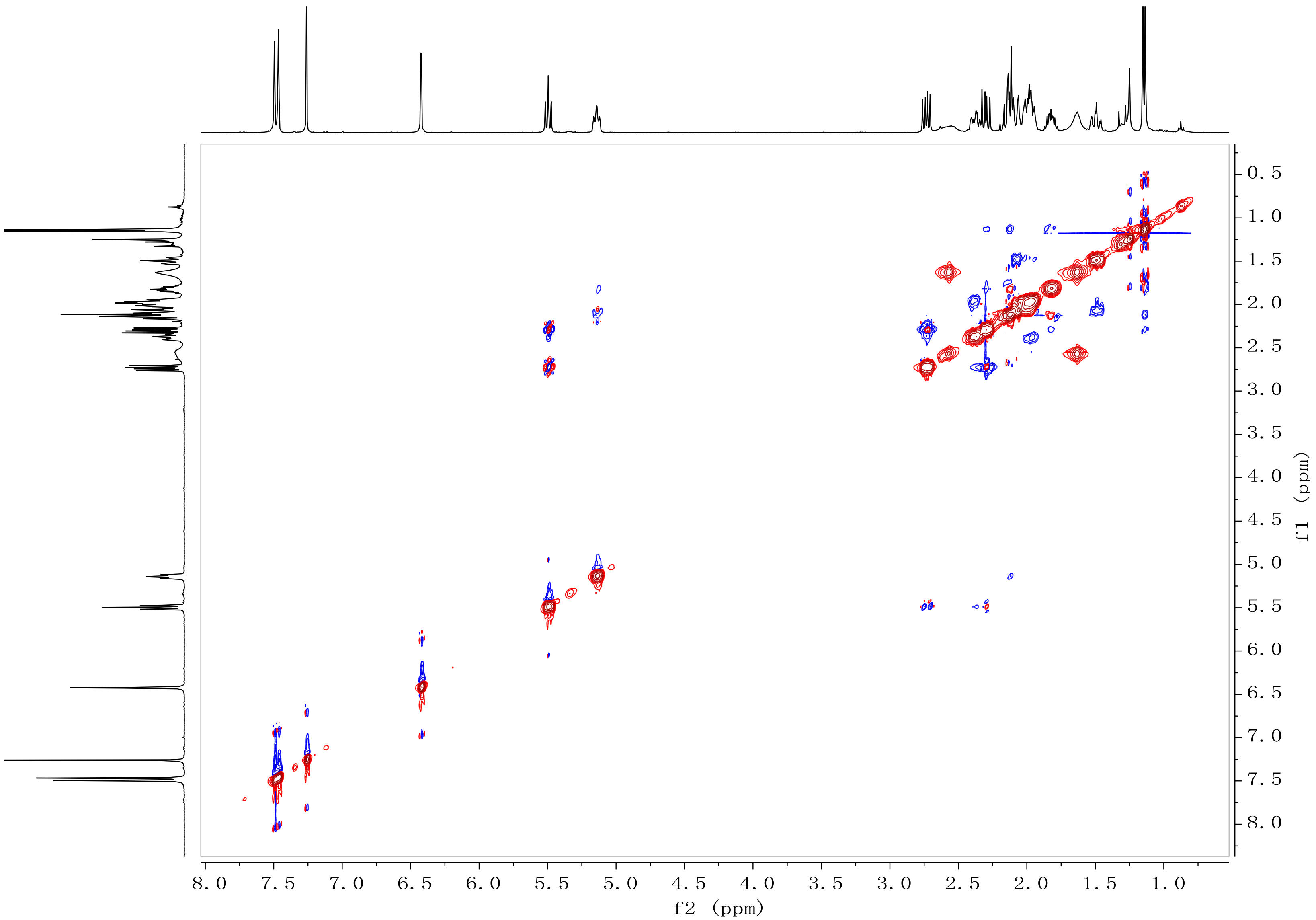

2) (+)-Teucvisin C (5)

COSY

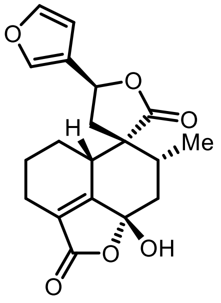

teucvisin C (**5**)

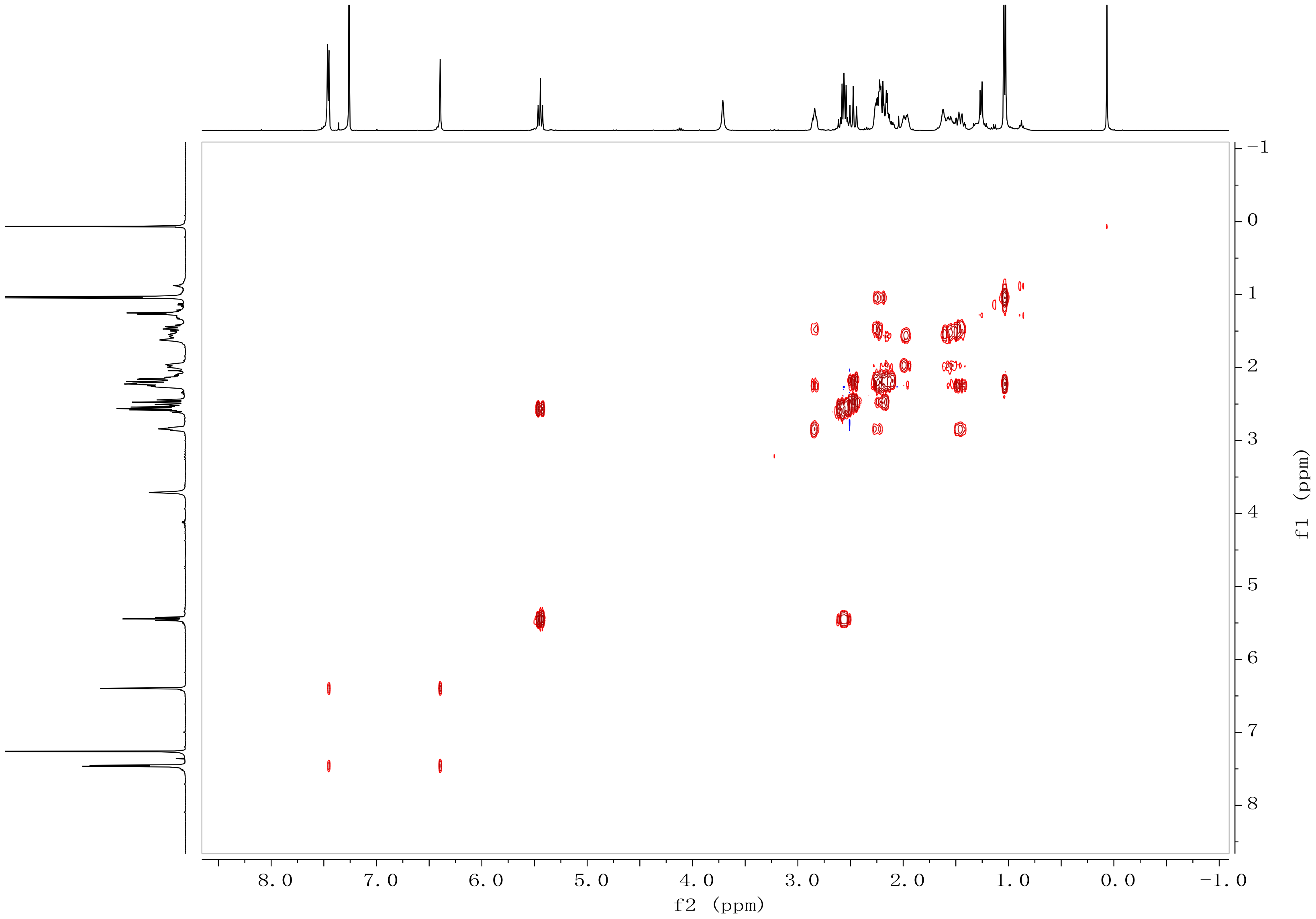

HSQC

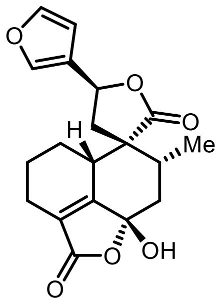

teucvisin C (**5**)

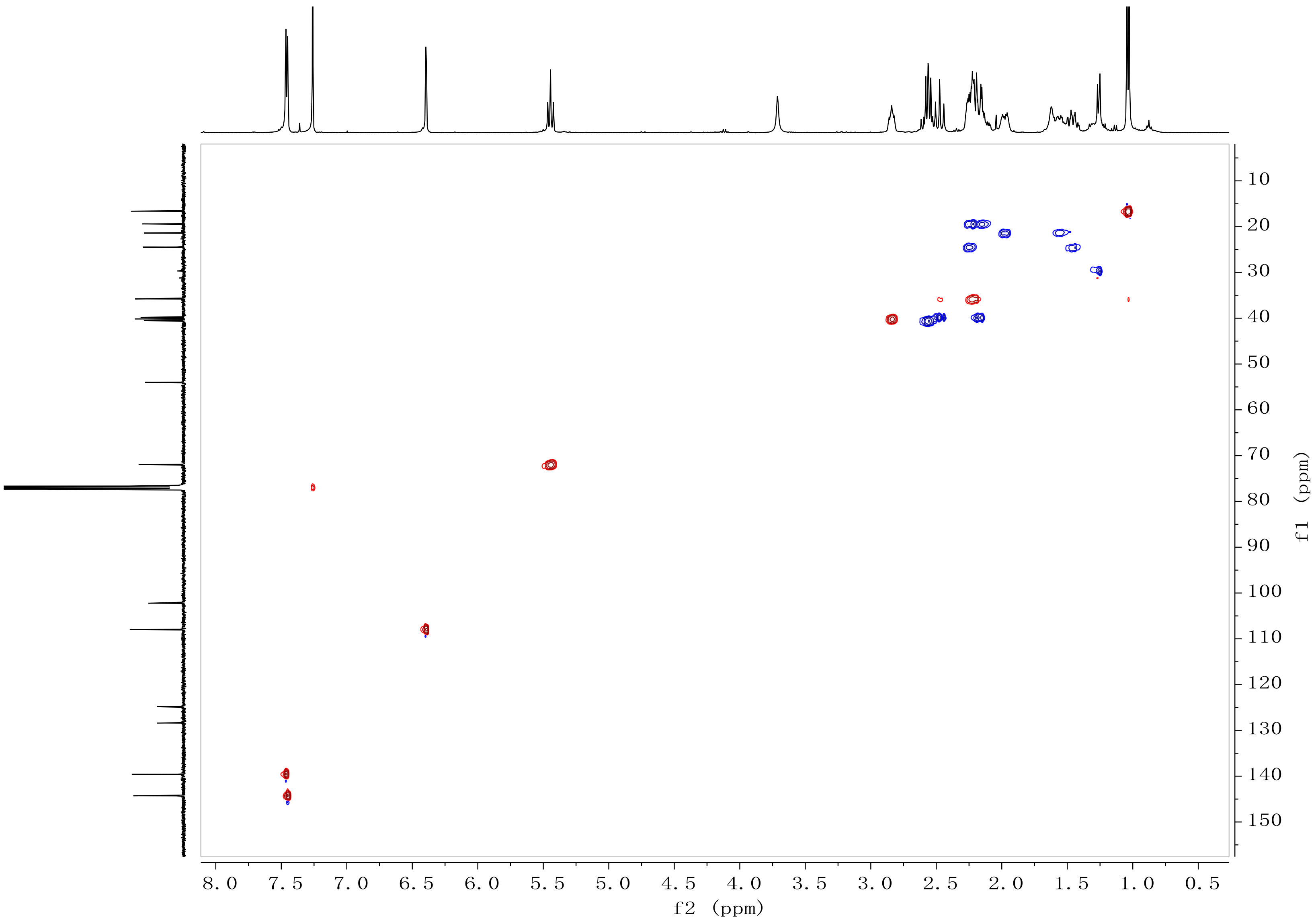

HMBC

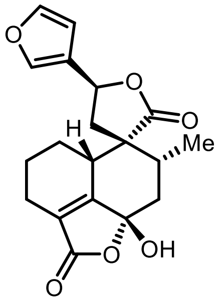

teucvisin C (**5**)

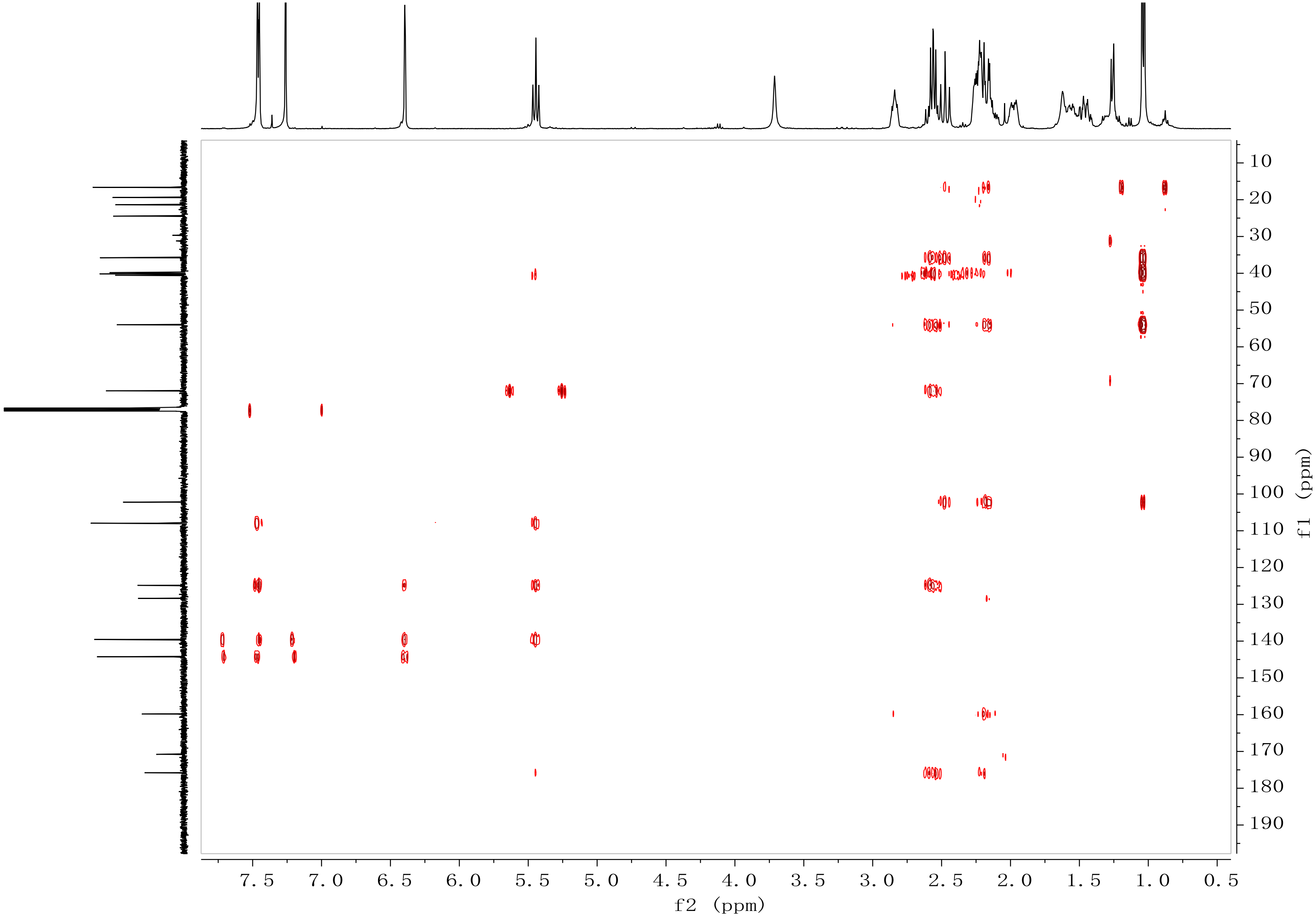

NOESY

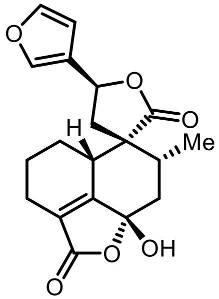

teucvisin C (**5**)

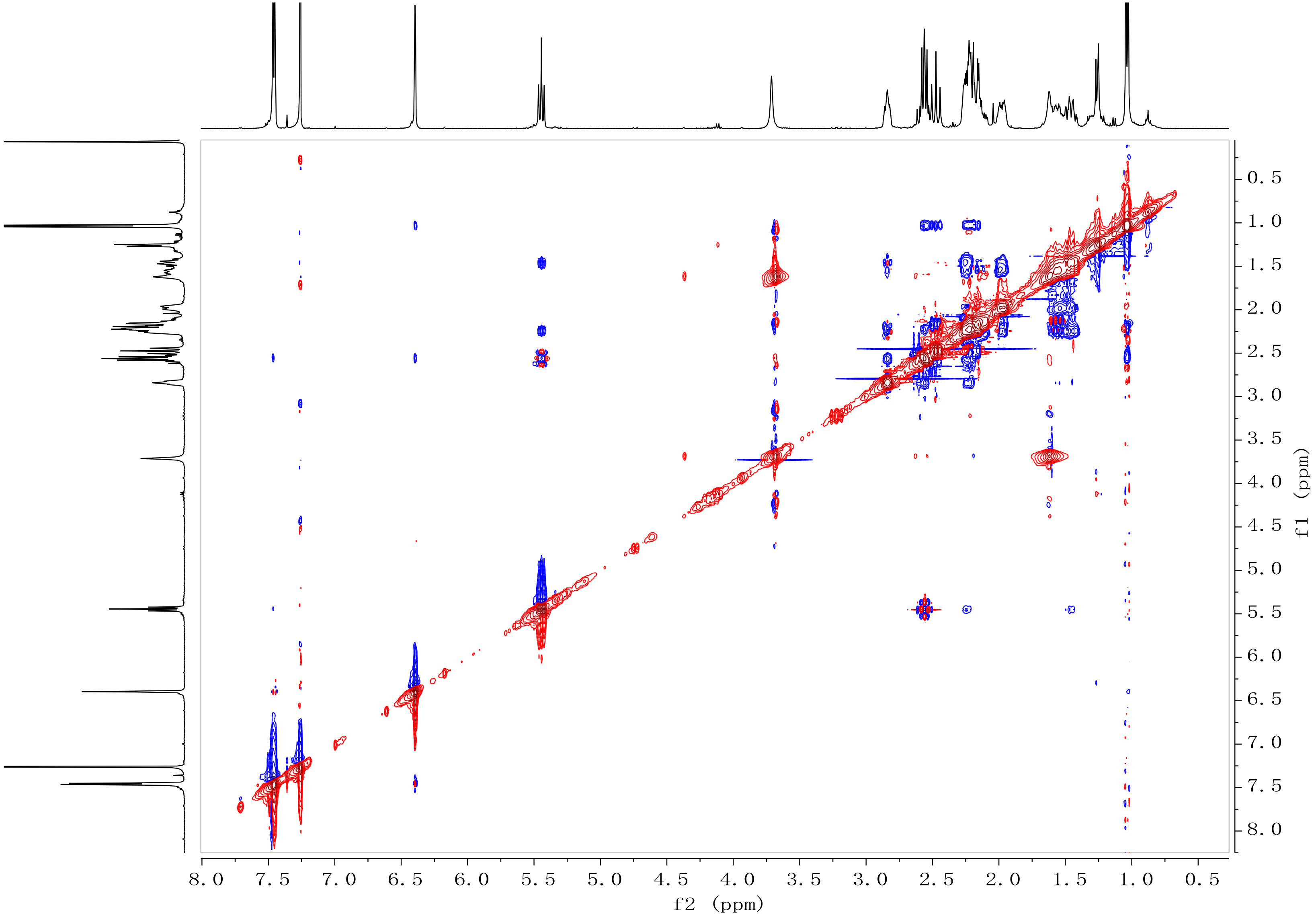

3) Compound 30

COSY

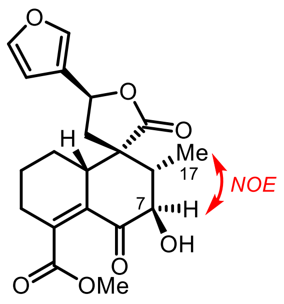

30

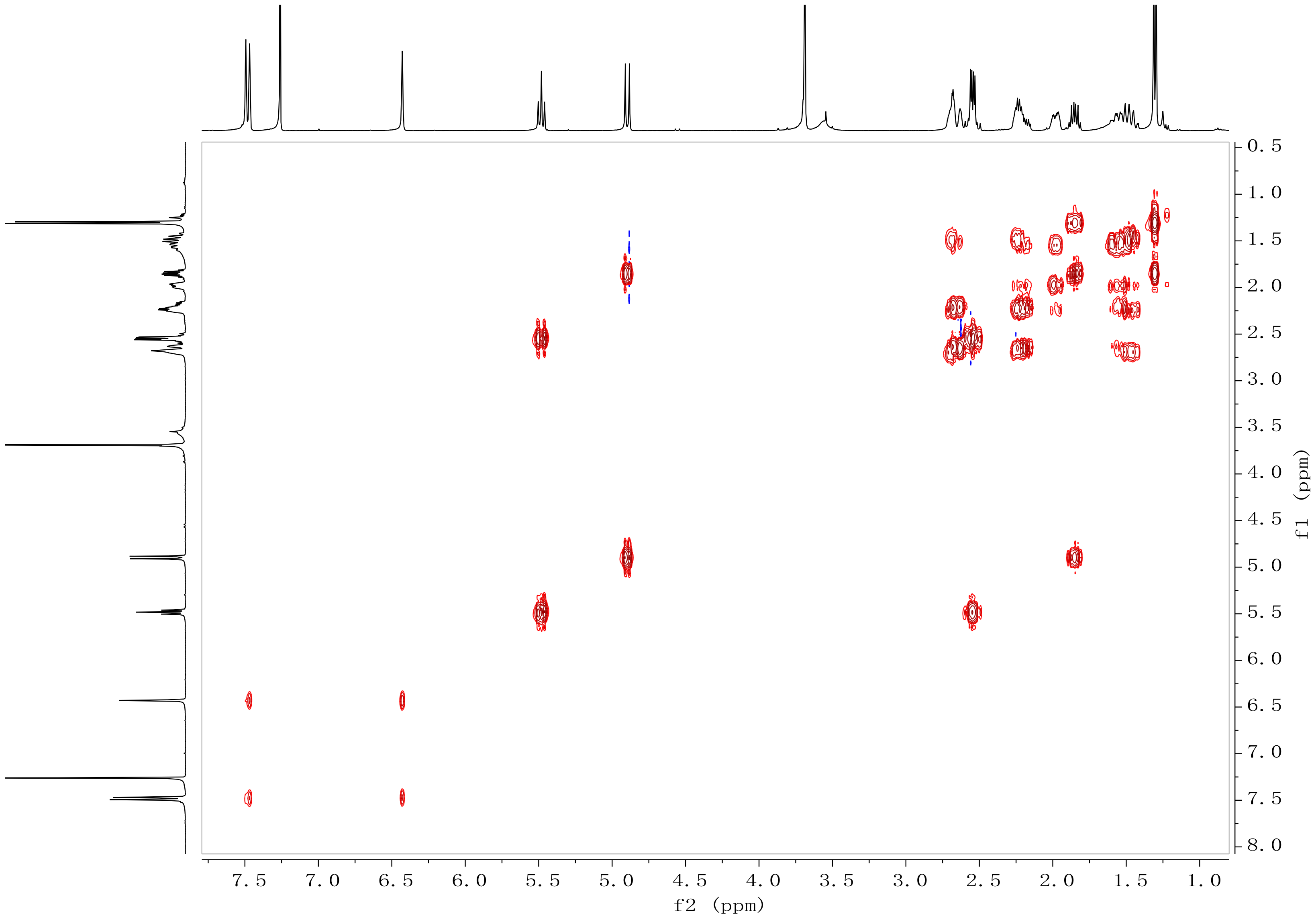

HSQC

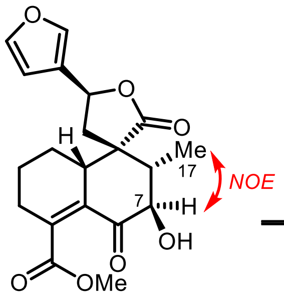

O
O
O
H
Me
17
NOE
7
H
OH
O
O
OMe

30

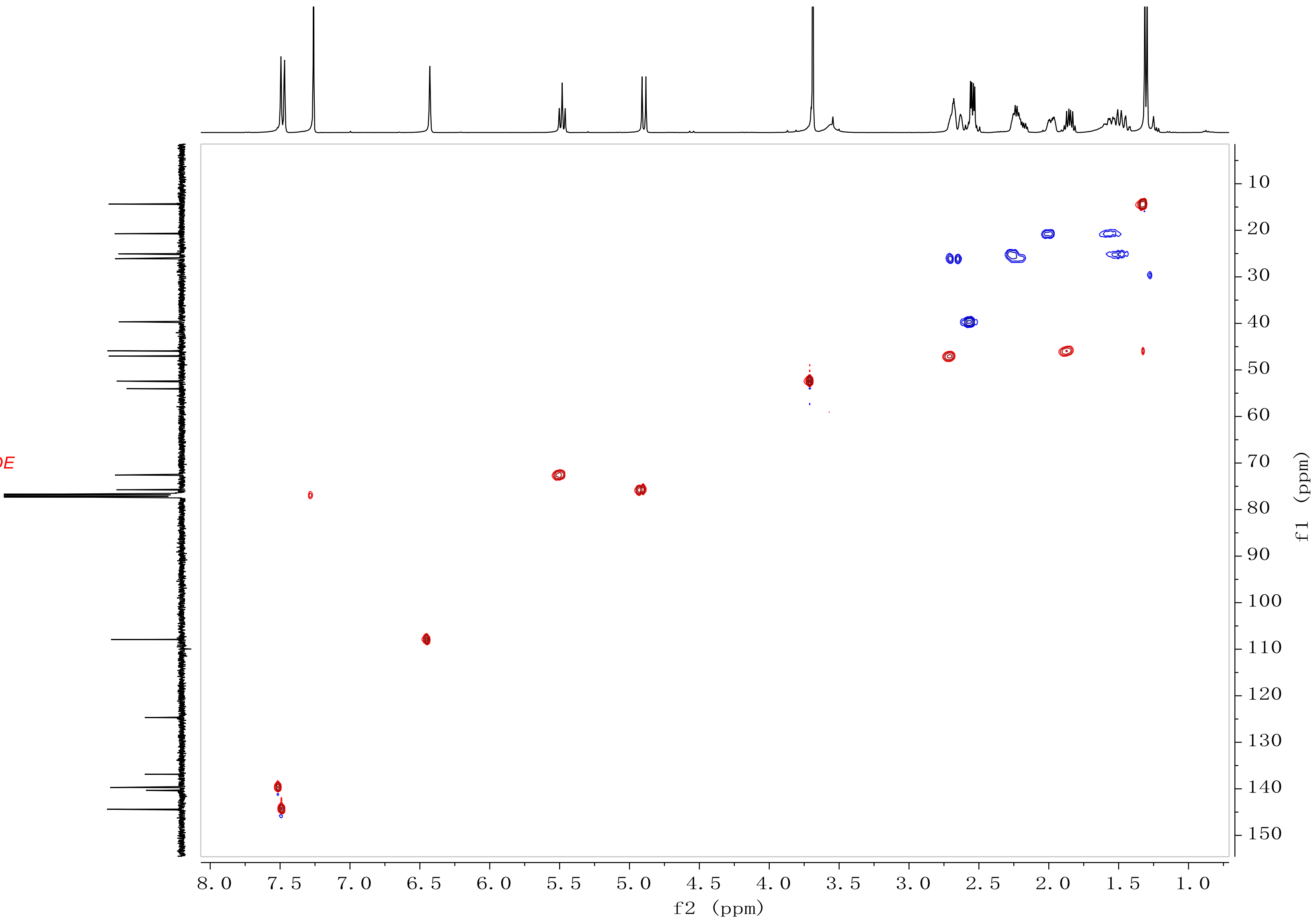

8.0
7.5
7.0
6.5
6.0
5.5
5.0
4.5
4.0
3.5
3.0
2.5
2.0
1.5
1.0
f2 (ppm)
10
20
30
40
50
60
70
80
90
100
110
120
130
140
150
f1 (ppm)

NOESY

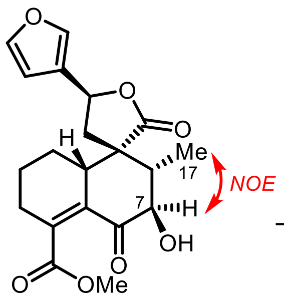

30

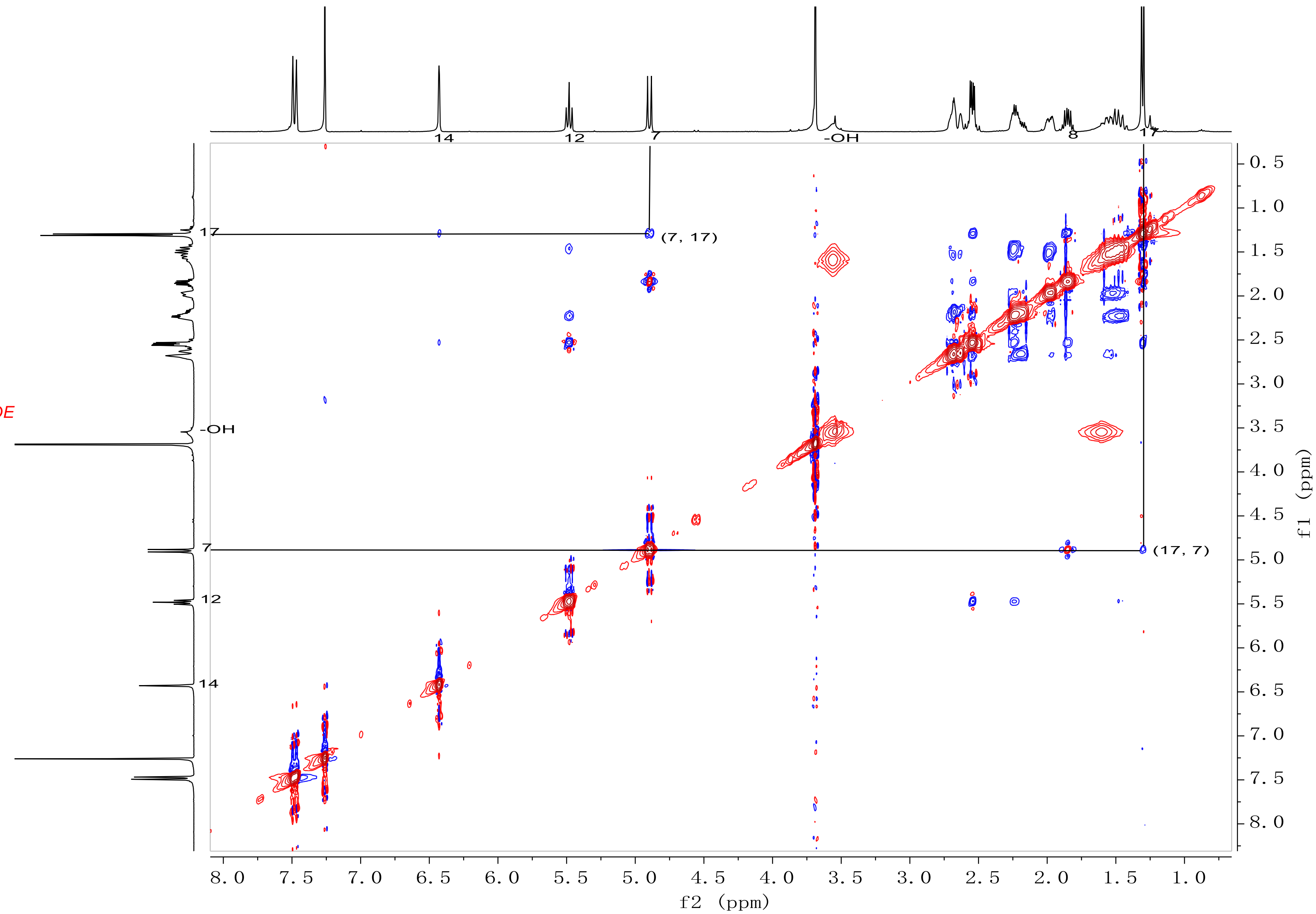

4) (+)-Cracroson E (7)

COSY

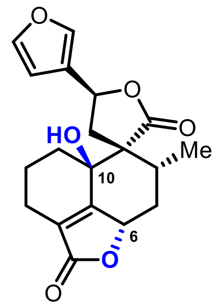

(+)-cracroson E (**7**)

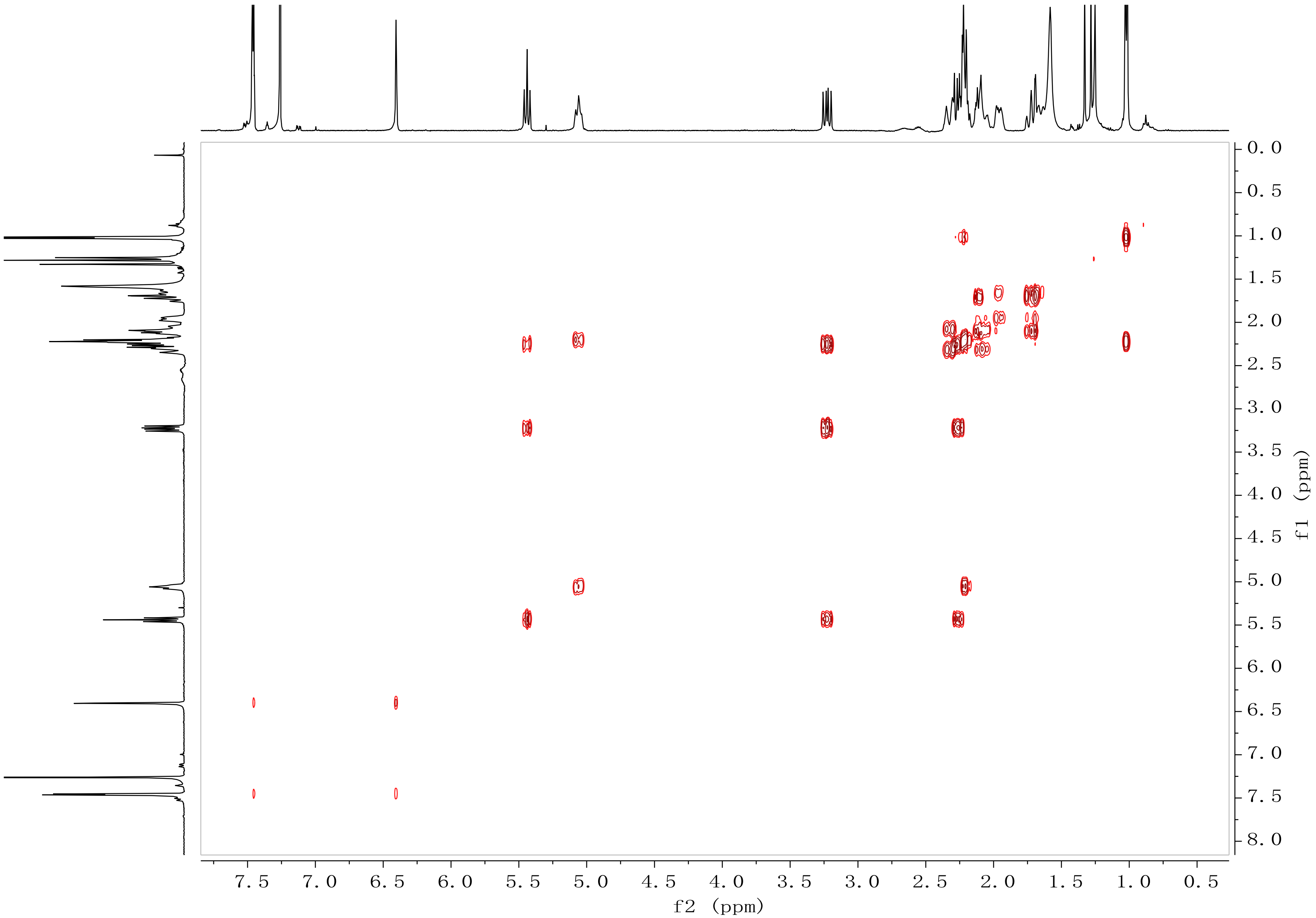

HSQC

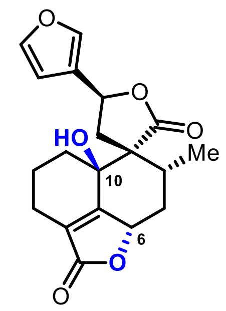

(+)-cracroson E (**7**)

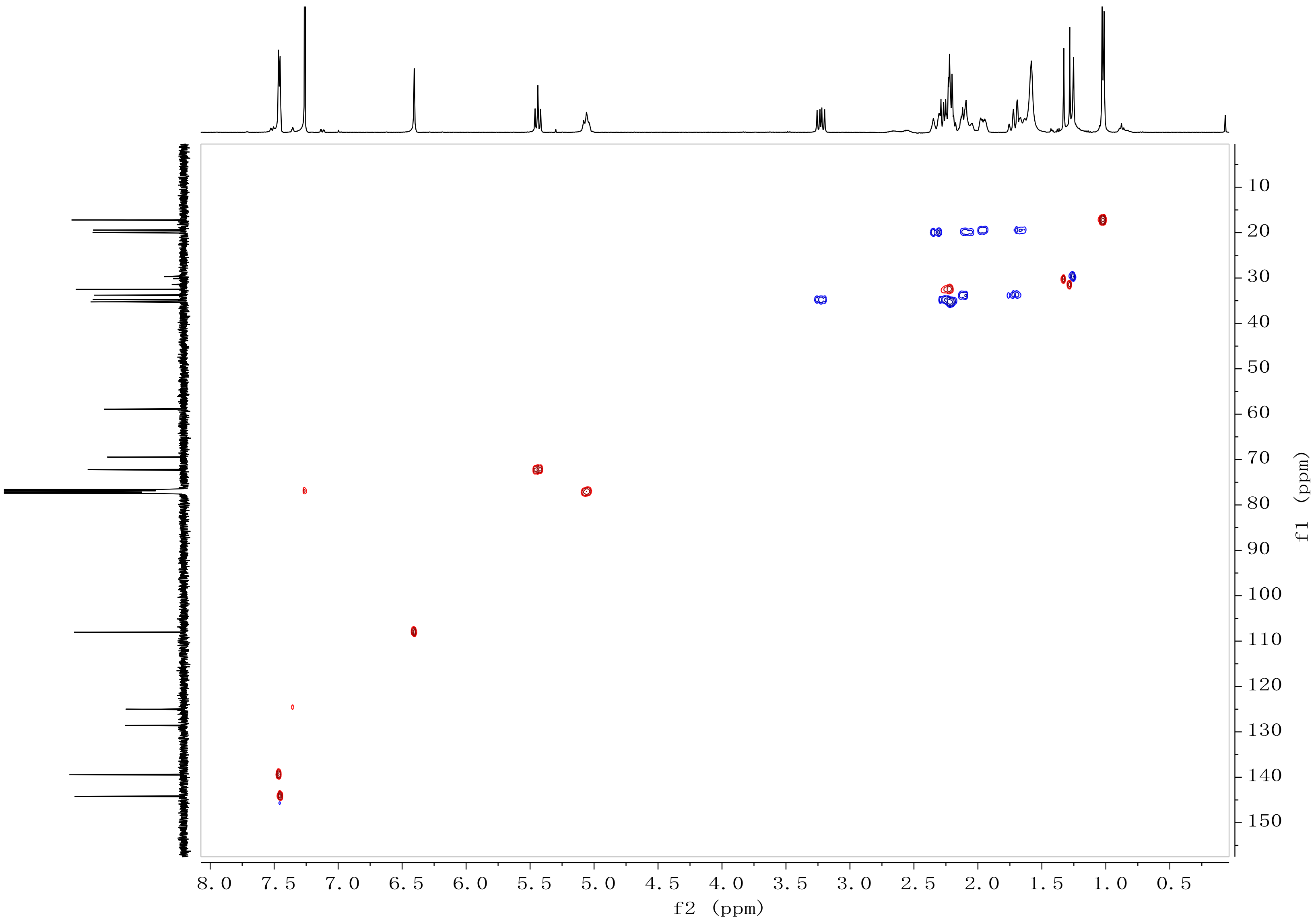

HMBC

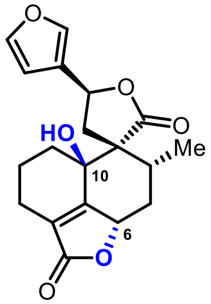

(+)-cracroson E (**7**)

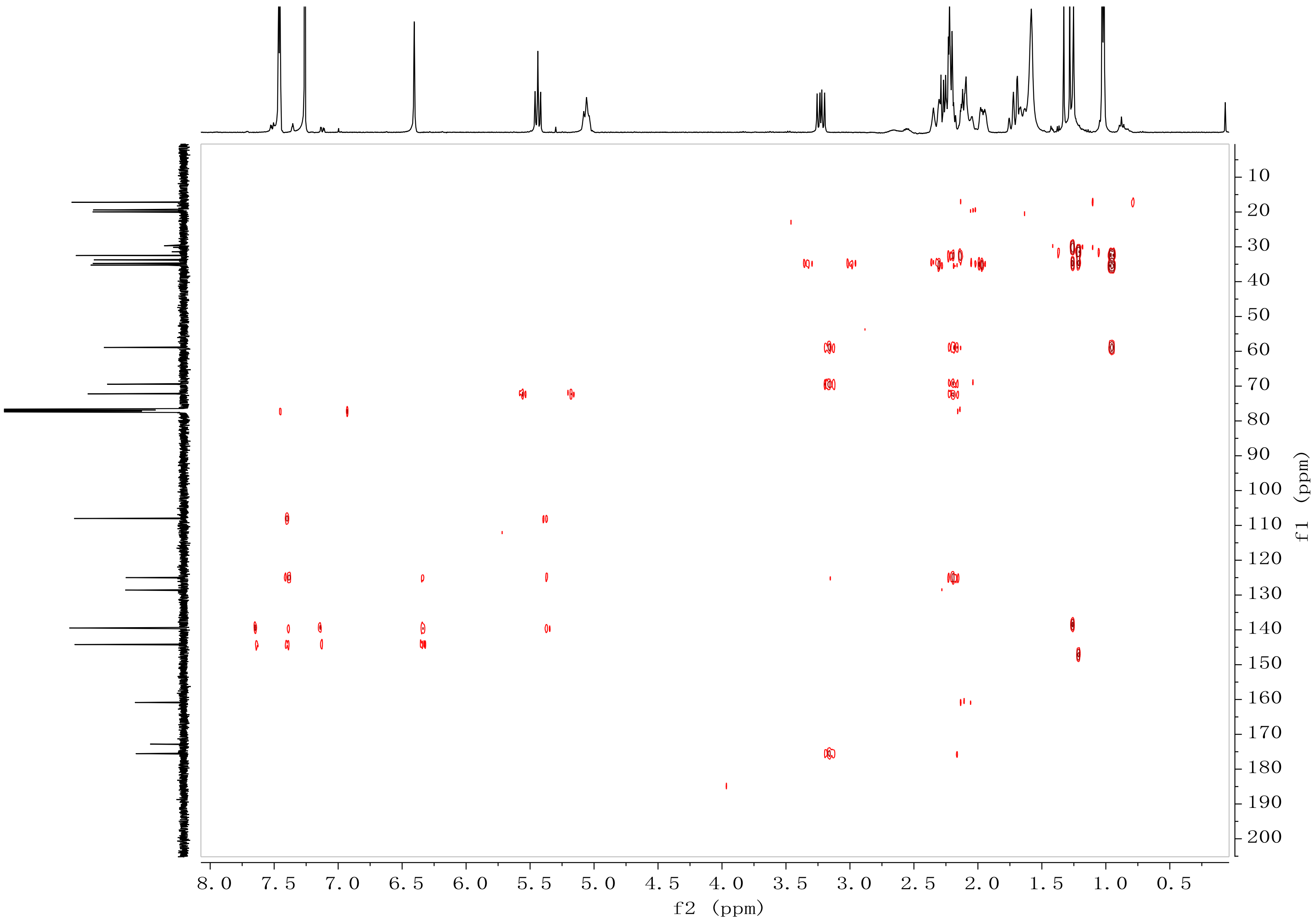

NOESY

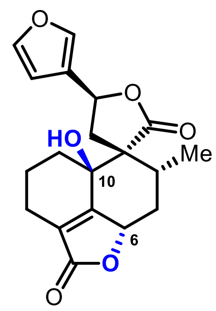

(+)-cracroson E (**7**)

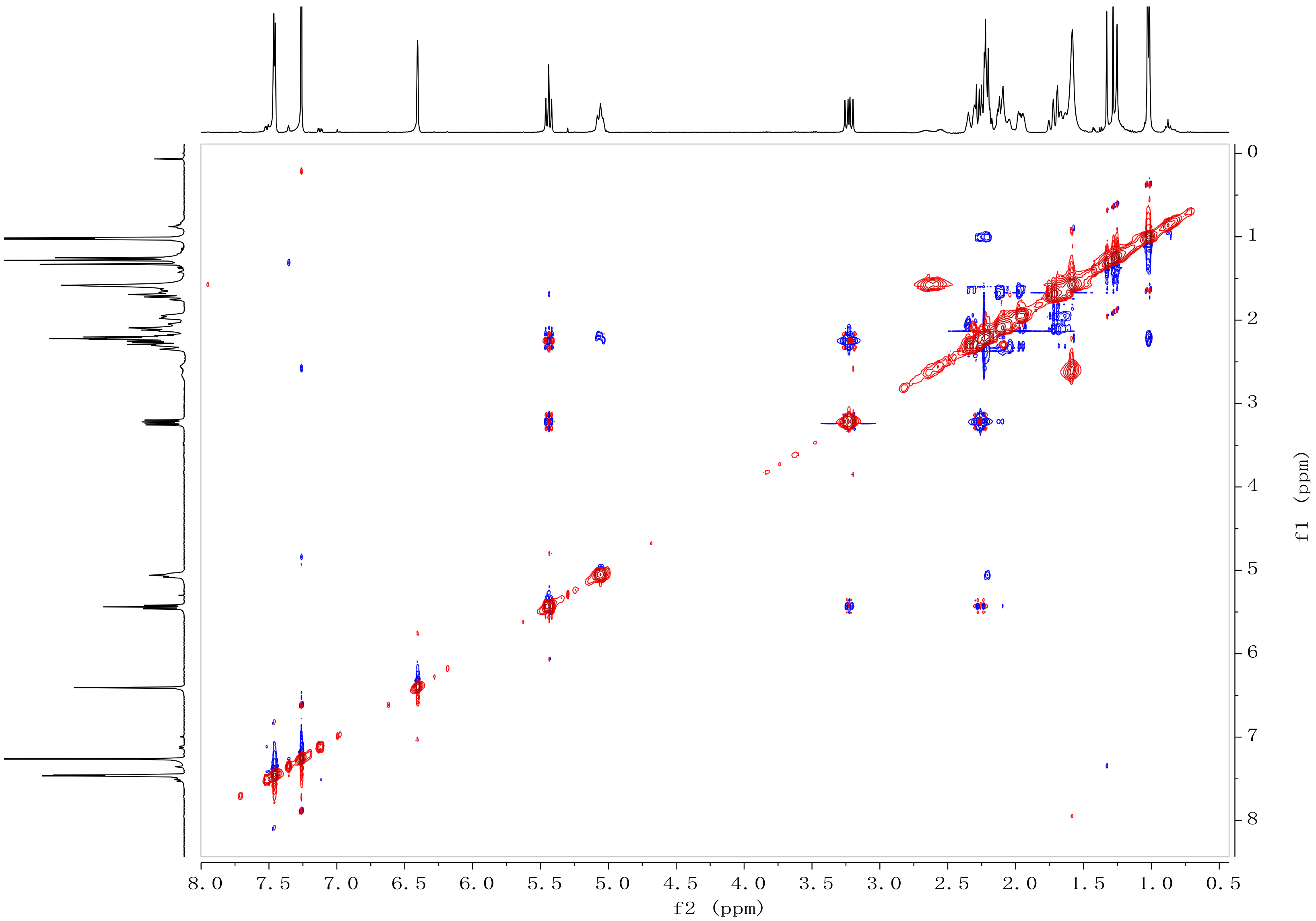

13. Copies of NMR Spectra

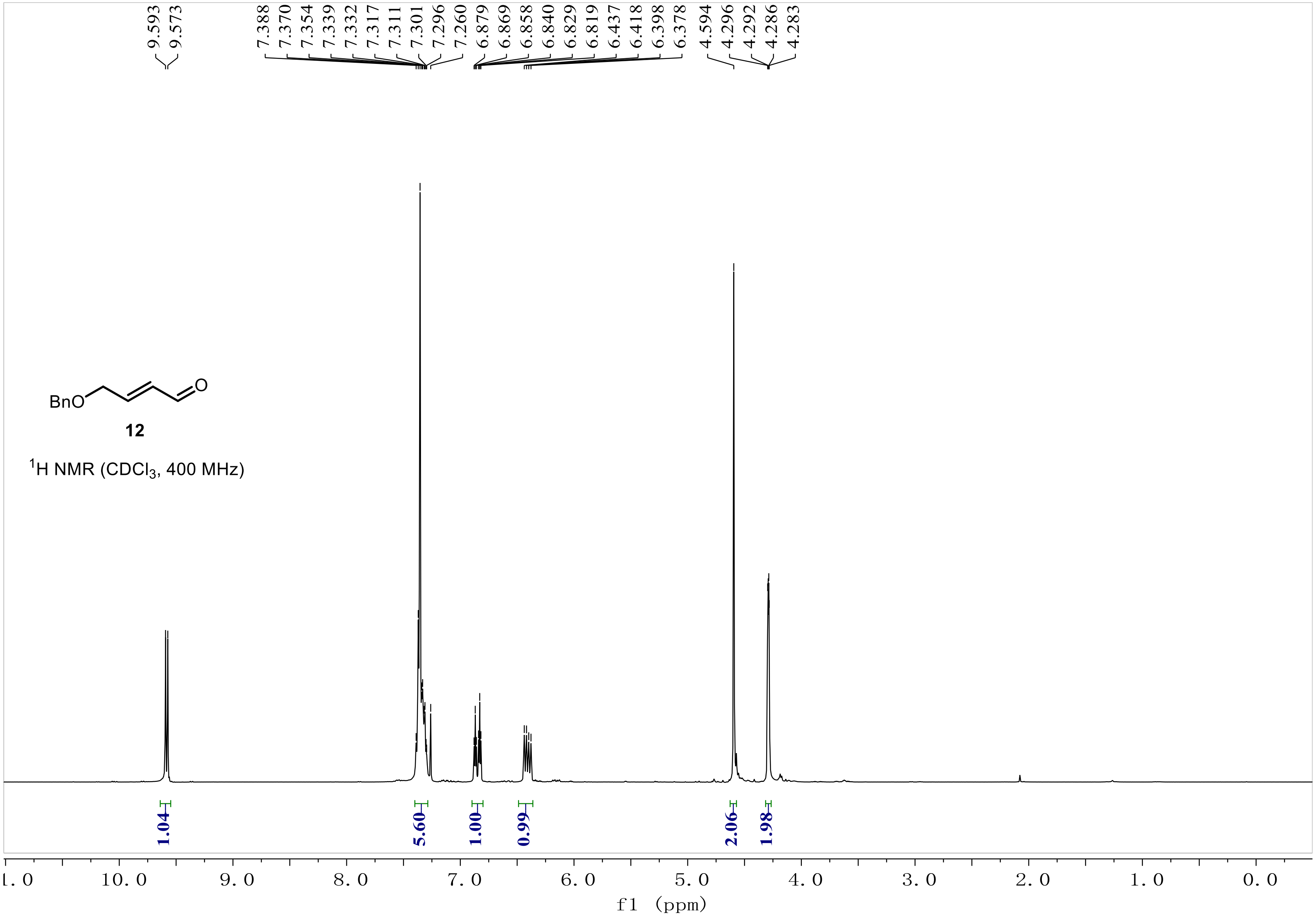

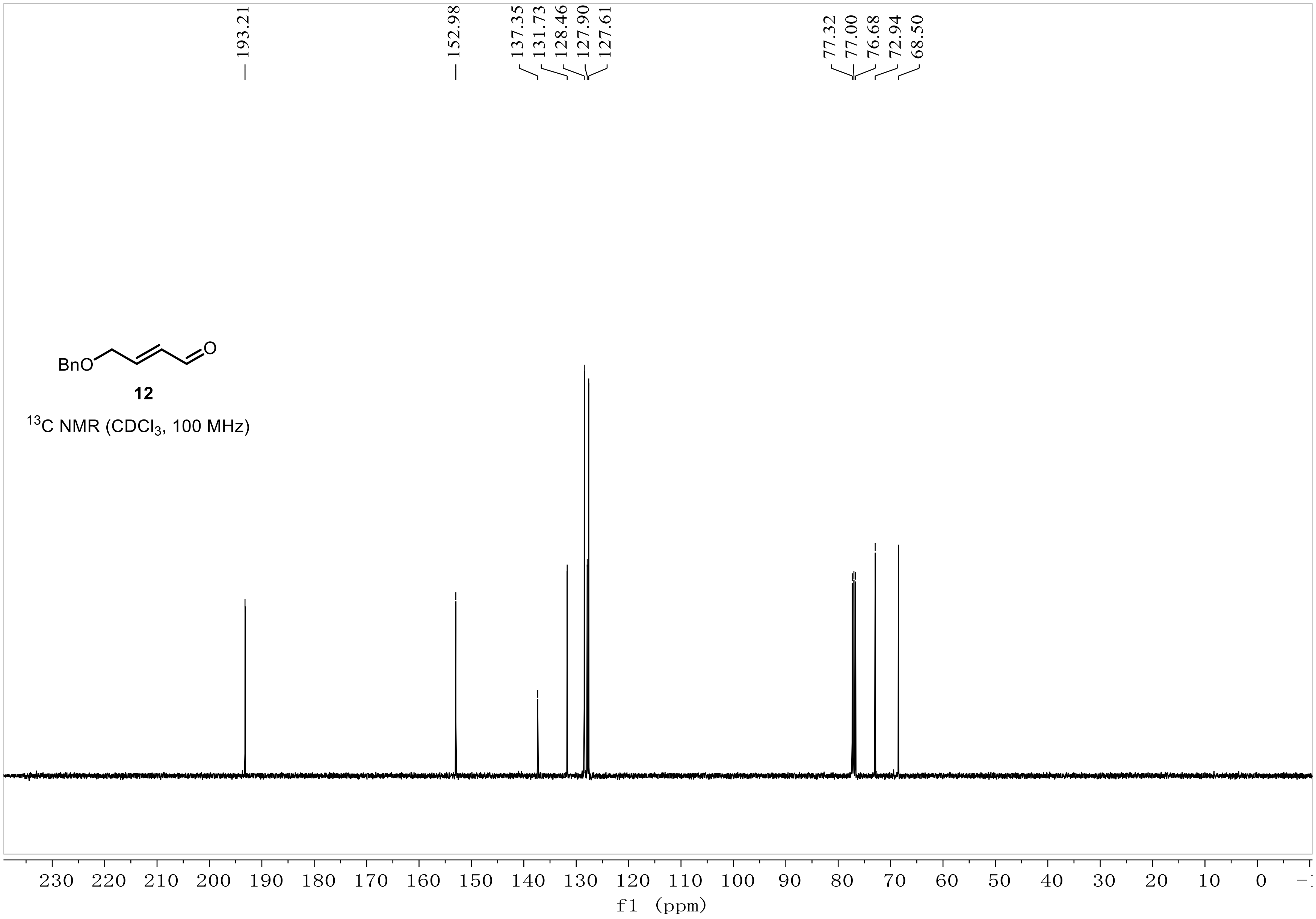
193.21
152.98
137.35
131.73
128.46
127.90
127.61
77.32
77.00
76.68
72.94
68.50
BnO
O
12
13C NMR (CDCl3, 100 MHz)
230 220 210 200 190 180 170 160 150 140 130 120 110 100 90 80 70 60 50 40 30 20 10 0
f1 (ppm)

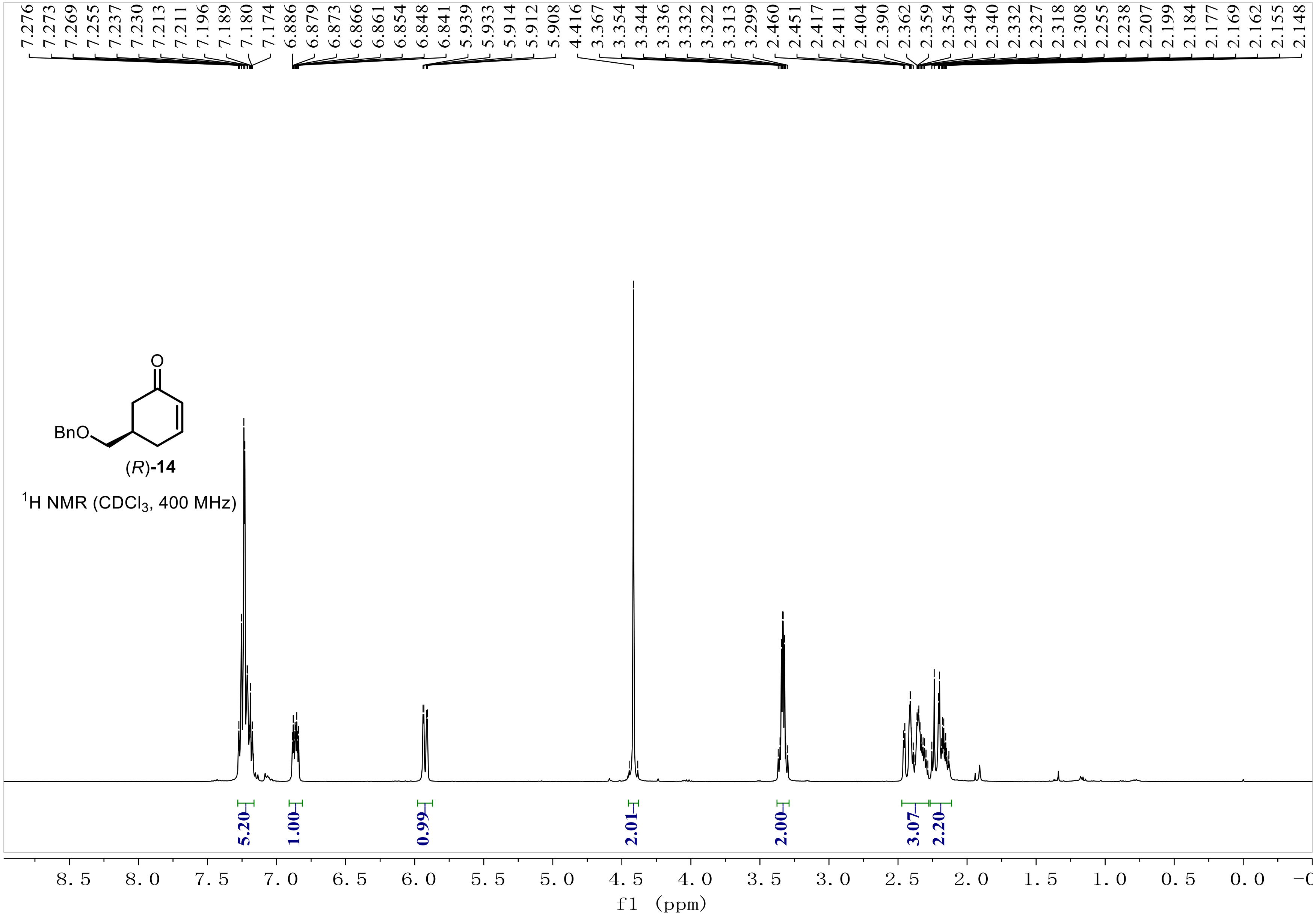
BnO
O
(R)-14
1H NMR (CDCl3, 400 MHz)
7.276 7.273 7.269 7.255 7.237 7.230 7.213 7.211 7.196 7.189 7.180 7.174 6.886 6.879 6.873 6.866 6.861 6.854 6.848 6.841 5.939 5.933 5.914 5.912 5.908 4.416 3.367 3.354 3.344 3.336 3.332 3.322 3.313 3.299 2.460 2.451 2.417 2.411 2.404 2.390 2.362 2.359 2.354 2.349 2.340 2.332 2.327 2.318 2.308 2.255 2.238 2.207 2.199 2.184 2.177 2.169 2.162 2.155 2.148
5.20 1.00 0.99 2.01 2.00 3.07 2.20
8.5 8.0 7.5 7.0 6.5 6.0 5.5 5.0 4.5 4.0 3.5 3.0 2.5 2.0 1.5 1.0 0.5 0.0
f1 (ppm)

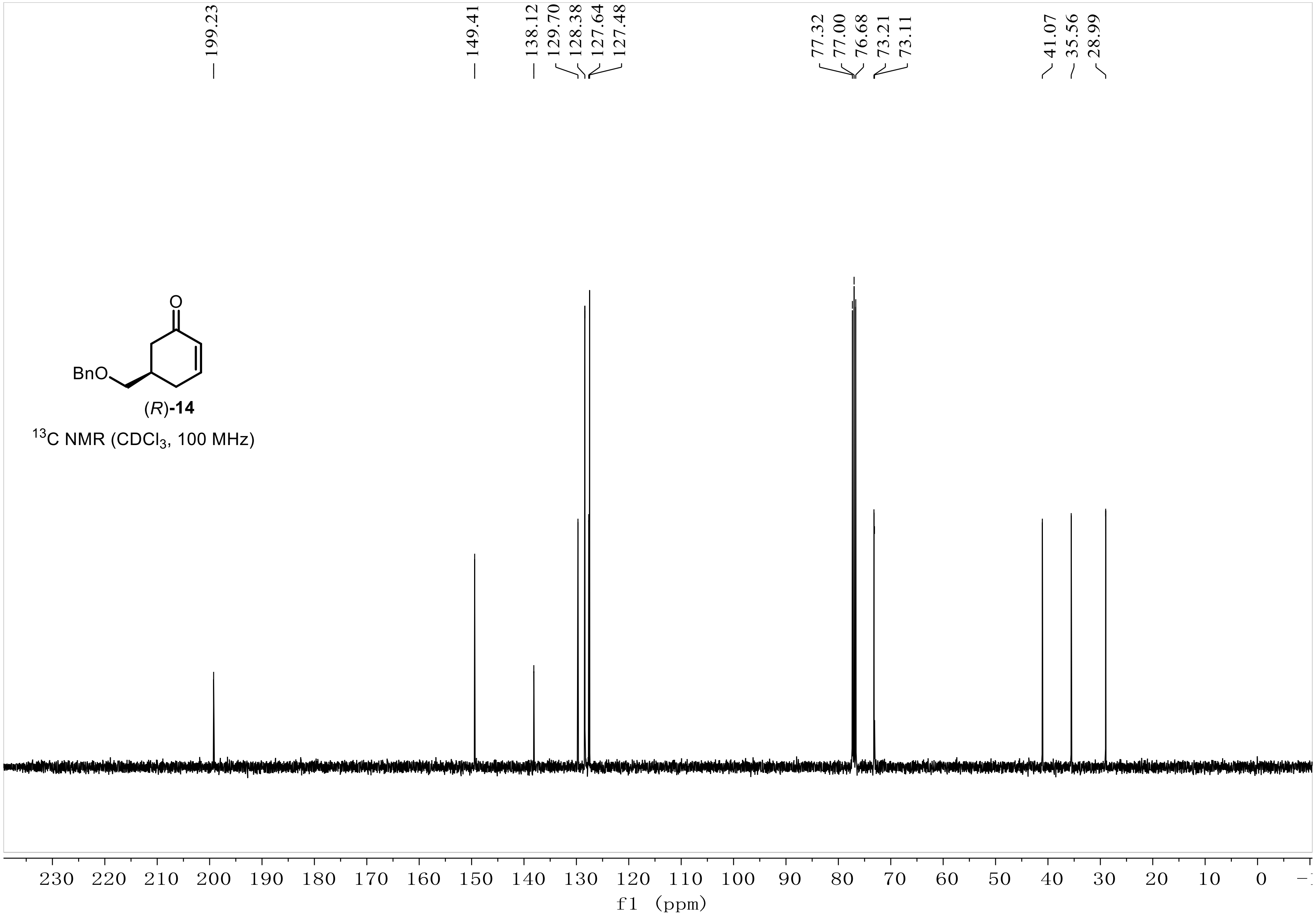
199.23
149.41
138.12
129.70
128.38
127.64
127.48
77.32
77.00
76.68
73.21
73.11
41.07
35.56
28.99
BnO
O
(R)-14
13C NMR (CDCl3, 100 MHz)
230 220 210 200 190 180 170 160 150 140 130 120 110 100 90 80 70 60 50 40 30 20 10 0
f1 (ppm)

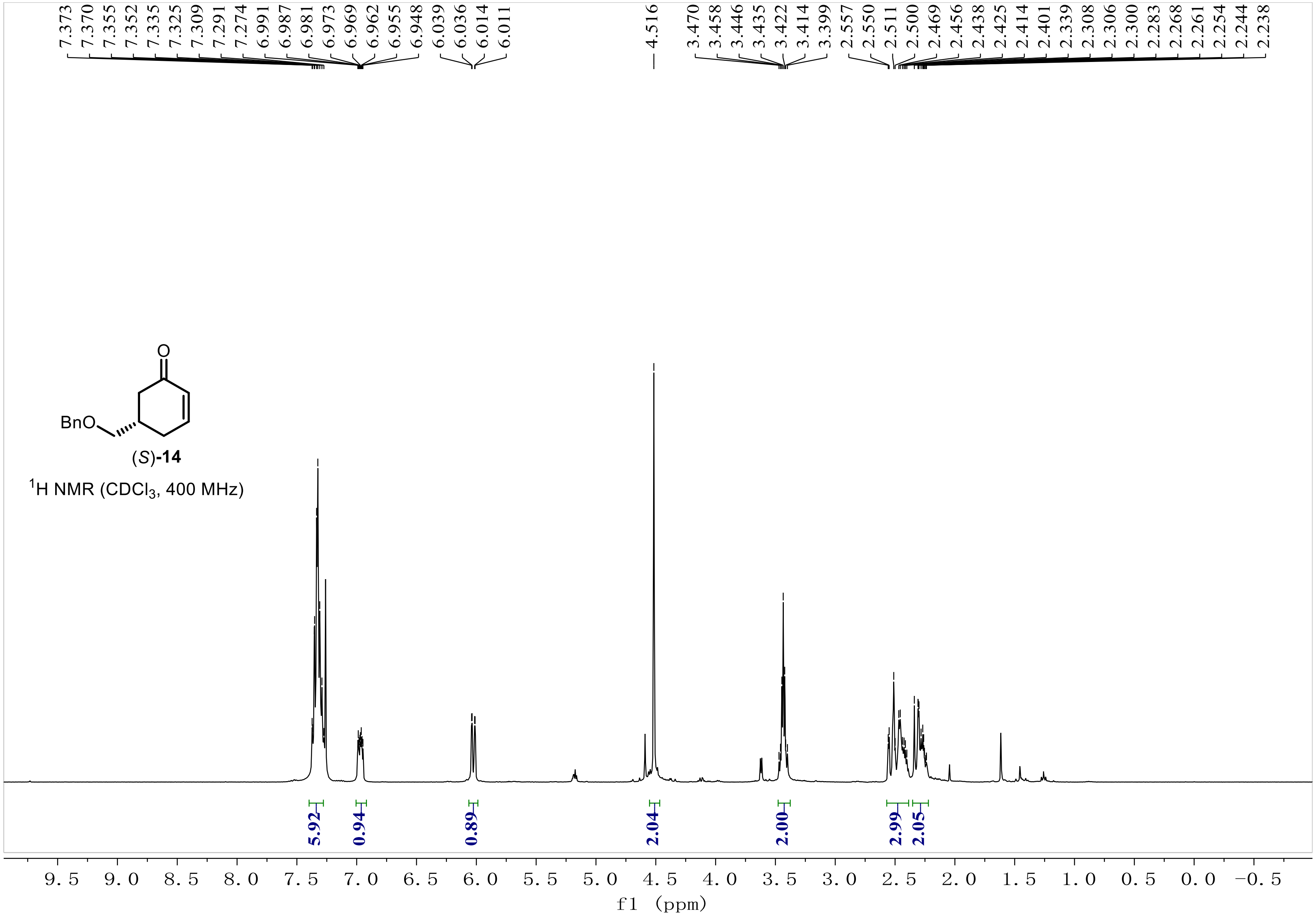

7.373
7.370
7.355
7.352
7.335
7.325
7.309
7.291
7.274
6.991
6.987
6.981
6.973
6.969
6.962
6.955
6.948
6.039
6.036
6.014
6.011
4.516
3.470
3.458
3.446
3.435
3.422
3.414
3.399
2.557
2.550
2.511
2.500
2.469
2.456
2.438
2.425
2.414
2.401
2.339
2.308
2.306
2.300
2.283
2.268
2.261
2.254
2.244
2.238
O
BnO
(S)-14
1H NMR (CDCl3, 400 MHz)
5.92
0.94
0.89
2.04
2.00
2.99
2.05
9.5 9.0 8.5 8.0 7.5 7.0 6.5 6.0 5.5 5.0 4.5 4.0 3.5 3.0 2.5 2.0 1.5 1.0 0.5 0.0 −0.5
f1 (ppm)

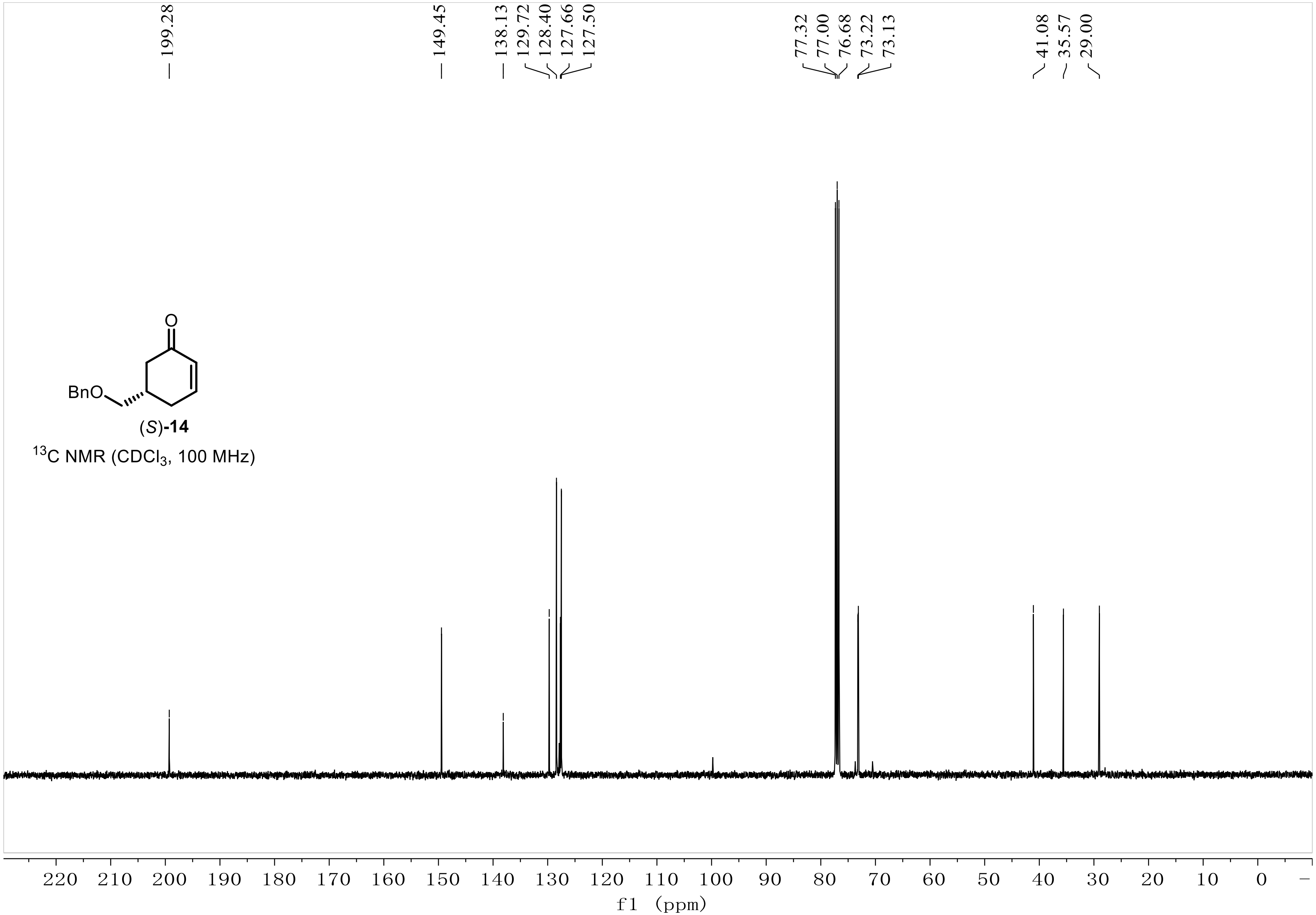
199.28
149.45
138.13
129.72
128.40
127.66
127.50
77.32
77.00
76.68
73.22
73.13
41.08
35.57
29.00
O
BnO
(S)-14
^{13}C NMR ($CDCl_3$, 100 MHz)
220 210 200 190 180 170 160 150 140 130 120 110 100 90 80 70 60 50 40 30 20 10 0
f1 (ppm)

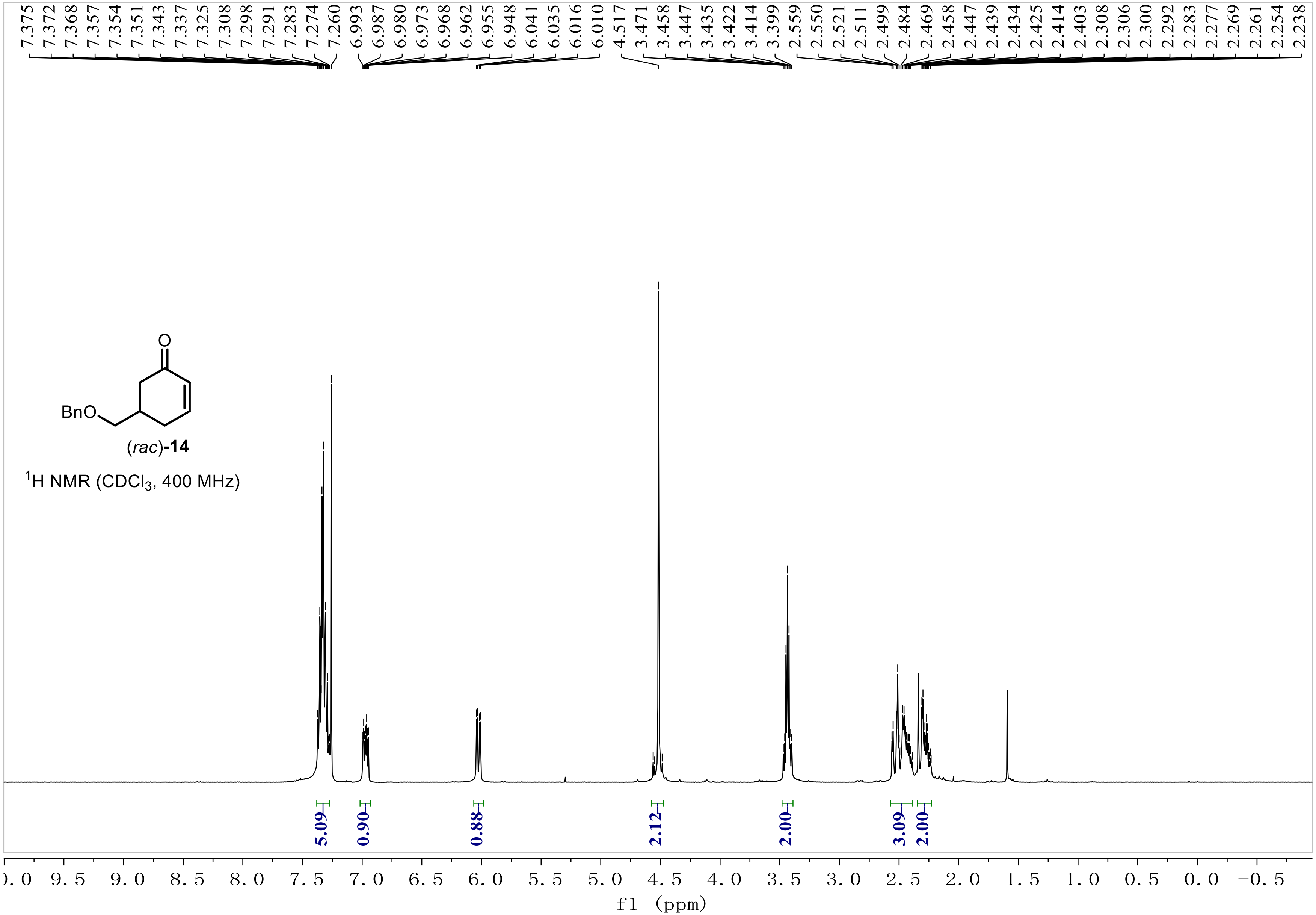
7.375 7.372 7.368 7.357 7.354 7.351 7.343 7.337 7.325 7.308 7.298 7.291 7.283 7.274 7.260 6.993 6.987 6.980 6.973 6.968 6.962 6.955 6.948 6.041 6.035 6.016 6.010 4.517 3.471 3.458 3.447 3.435 3.422 3.414 3.399 2.559 2.550 2.521 2.511 2.499 2.484 2.469 2.458 2.447 2.439 2.434 2.425 2.414 2.403 2.308 2.306 2.300 2.292 2.283 2.277 2.269 2.261 2.254 2.238
O
BnO
(rac)-14
1H NMR (CDCl3, 400 MHz)
5.09
0.90
0.88
2.12
2.00
3.09
2.00
f1 (ppm)

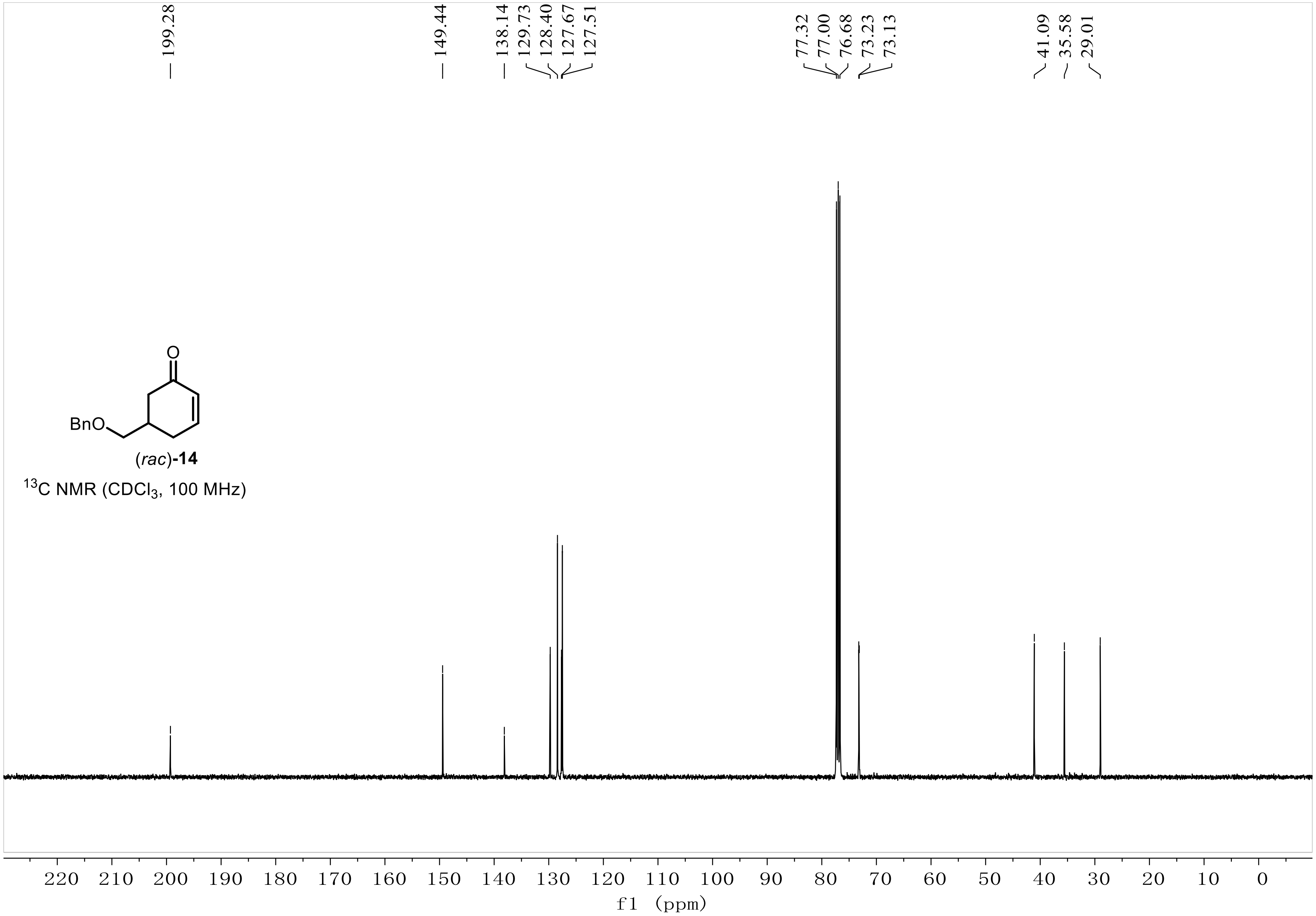
199.28
149.44
138.14
129.73
128.40
127.67
127.51
77.32
77.00
76.68
73.23
73.13
41.09
35.58
29.01
O
BnO
(rac)-14
13C NMR (CDCl3, 100 MHz)
220 210 200 190 180 170 160 150 140 130 120 110 100 90 80 70 60 50 40 30 20 10 0
f1 (ppm)

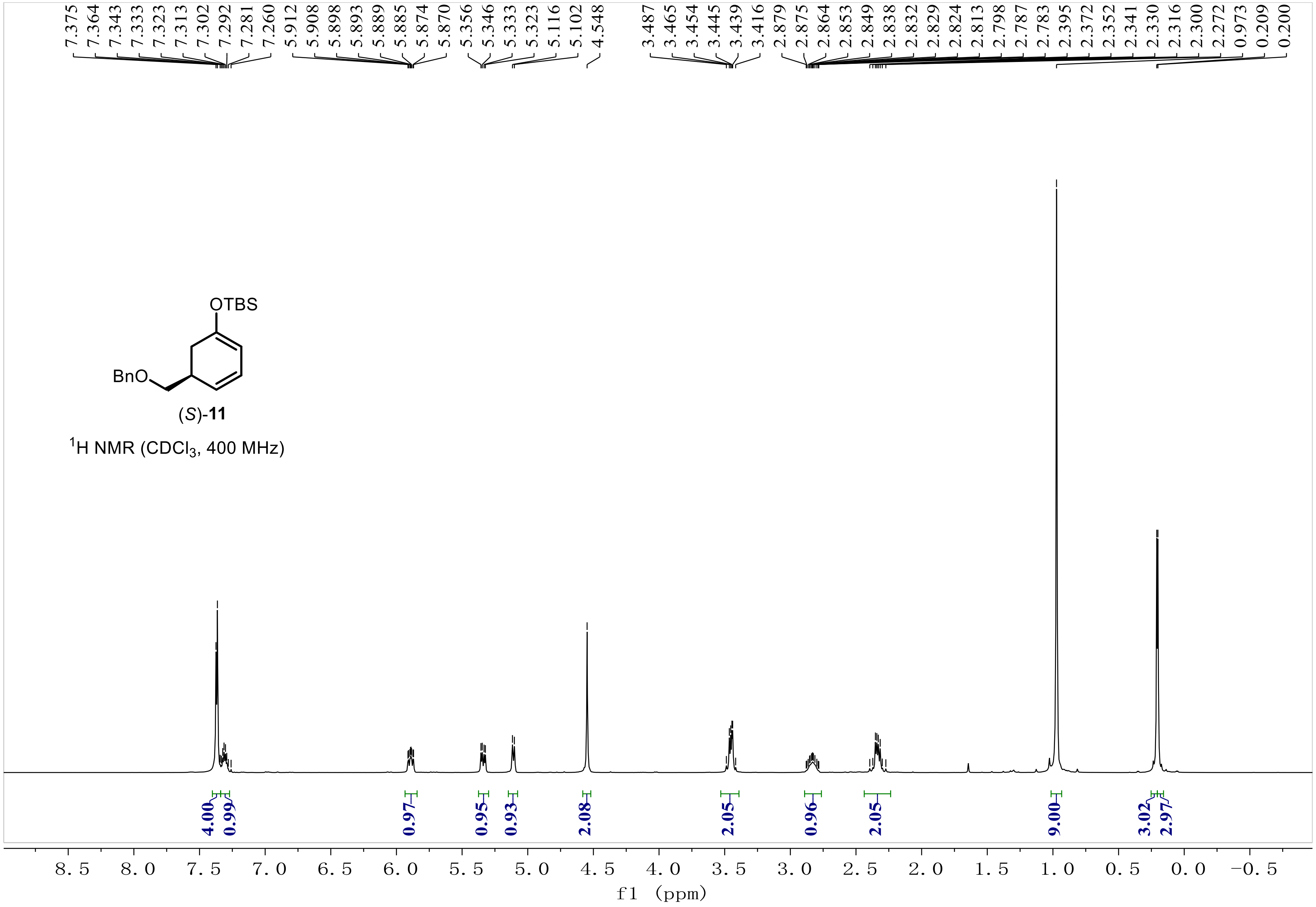

OTBS
BnO
(S)-11
1H NMR (CDCl3, 400 MHz)
7.375 7.364 7.343 7.333 7.323 7.313 7.302 7.292 7.281 7.260 5.912 5.908 5.898 5.893 5.889 5.885 5.874 5.870 5.356 5.346 5.333 5.323 5.116 5.102 4.548 3.487 3.465 3.454 3.445 3.439 3.416 2.879 2.875 2.864 2.853 2.849 2.838 2.832 2.829 2.824 2.813 2.798 2.787 2.783 2.395 2.372 2.352 2.341 2.330 2.316 2.300 2.272 0.973 0.209 0.200
4.00 0.99 0.97 0.95 0.93 2.08 2.05 0.96 2.05 9.00 3.02 2.97
8.5 8.0 7.5 7.0 6.5 6.0 5.5 5.0 4.5 4.0 3.5 3.0 2.5 2.0 1.5 1.0 0.5 0.0 -0.5
f1 (ppm)

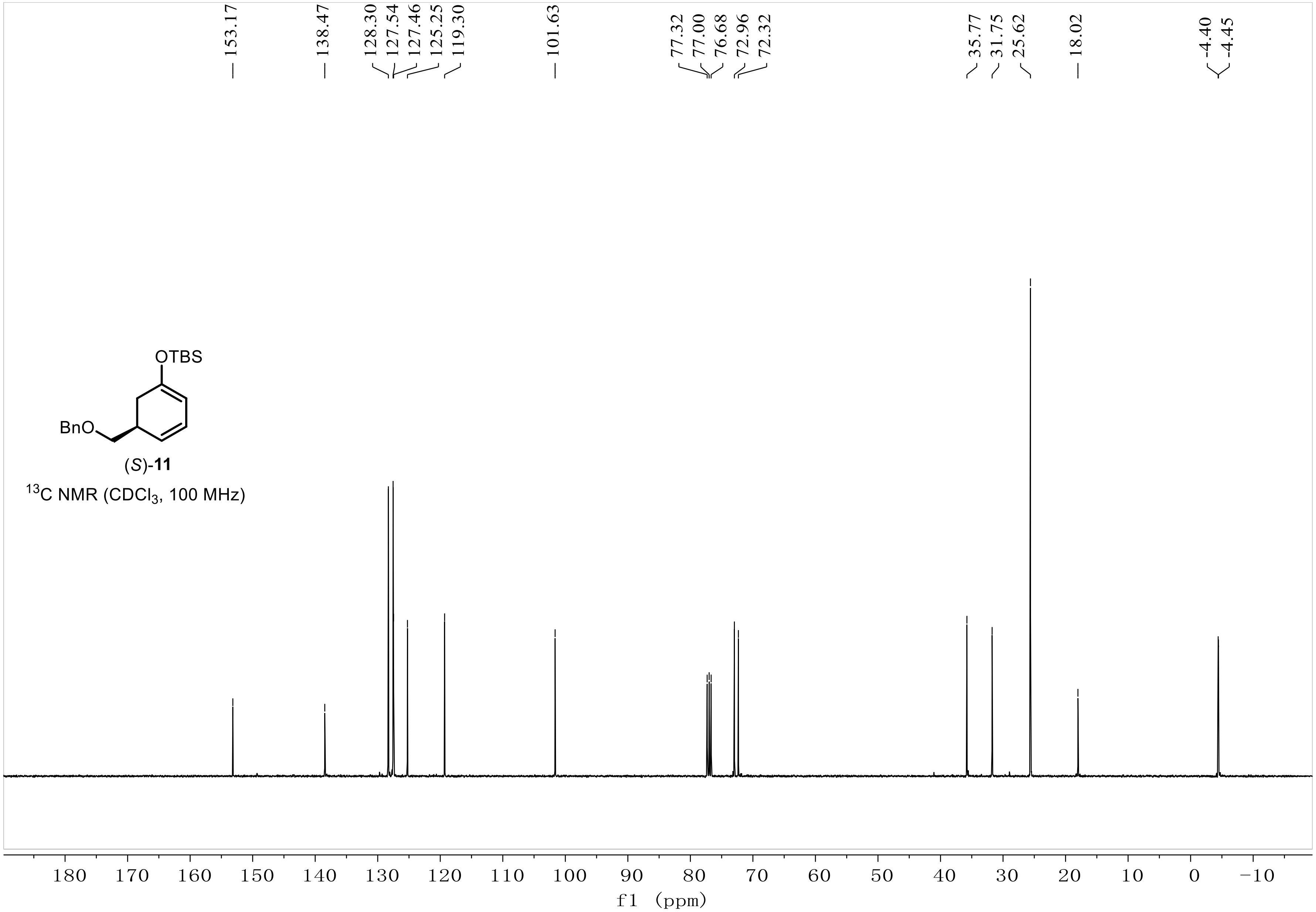

(*S*)-**11**

^{13}C NMR ($CDCl_3$, 100 MHz)

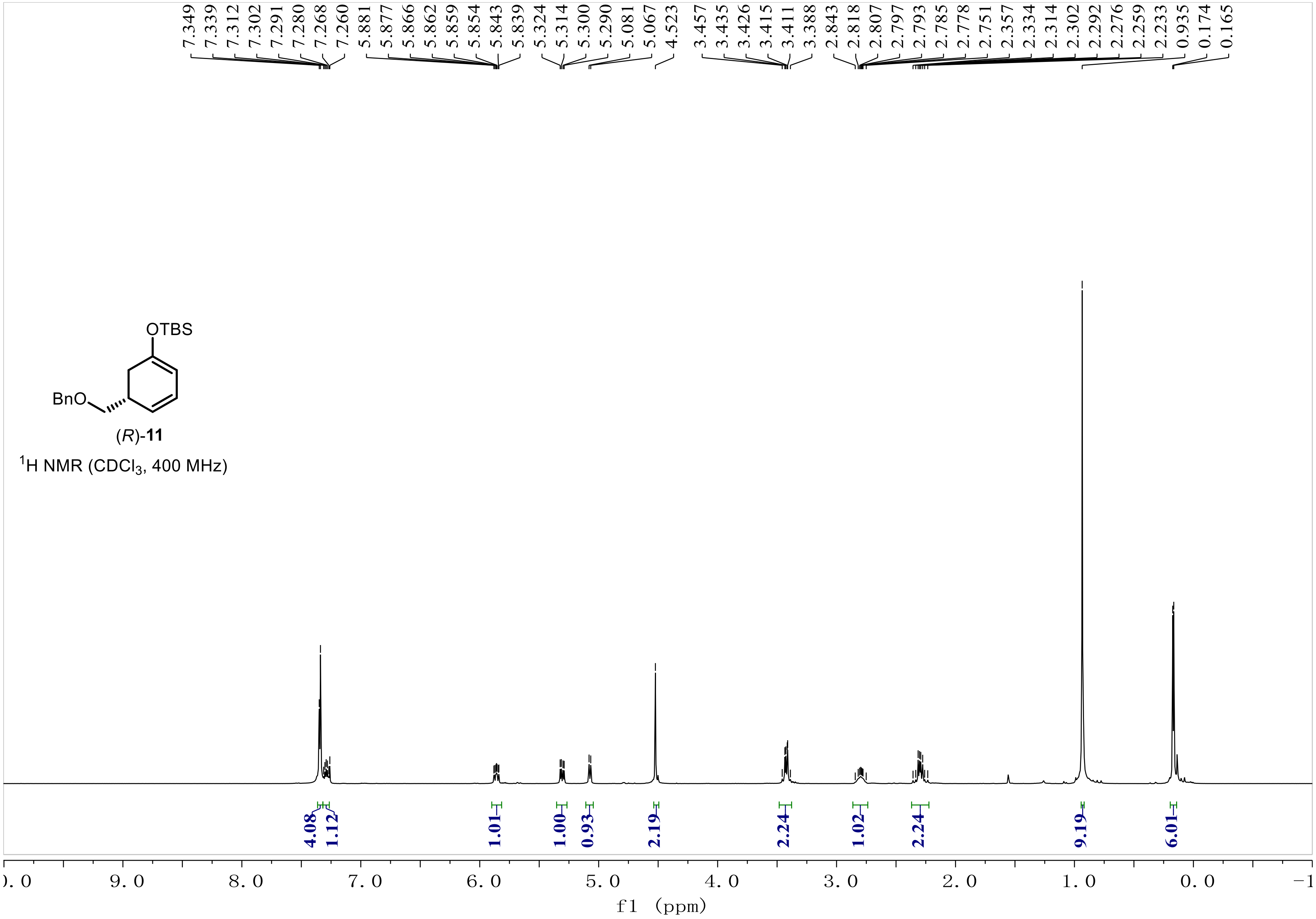
7.349 7.339 7.312 7.302 7.291 7.280 7.268 7.260 5.881 5.877 5.866 5.862 5.859 5.854 5.843 5.839 5.324 5.314 5.300 5.290 5.081 5.067 4.523 3.457 3.435 3.426 3.415 3.411 3.388 2.843 2.818 2.807 2.797 2.793 2.785 2.778 2.751 2.357 2.334 2.314 2.302 2.292 2.276 2.259 2.233 0.935 0.174 0.165
OTBS
BnO
(R)-11
1H NMR (CDCl3, 400 MHz)
4.08 1.12 1.01 1.00 0.93 2.19 2.24 1.02 2.24 9.19 6.01
9.0 8.0 7.0 6.0 5.0 4.0 3.0 2.0 1.0 0.0
f1 (ppm)

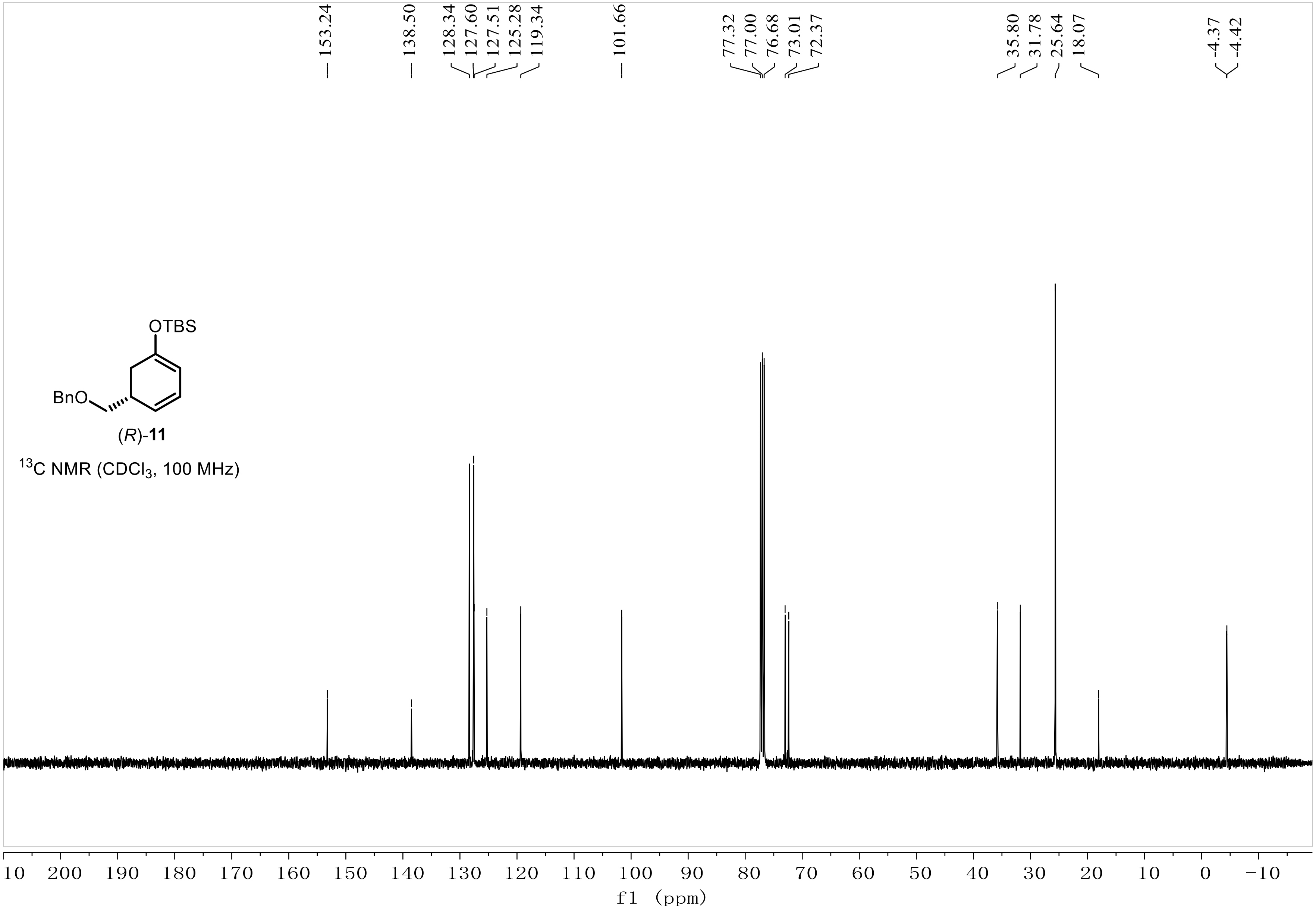
153.24
138.50
128.34
127.60
127.51
125.28
119.34
101.66
77.32
77.00
76.68
73.01
72.37
35.80
31.78
25.64
18.07
-4.37
-4.42
OTBS
BnO
(R)-11
13C NMR (CDCl3, 100 MHz)
10 200 190 180 170 160 150 140 130 120 110 100 90 80 70 60 50 40 30 20 10 0 −10
f1 (ppm)

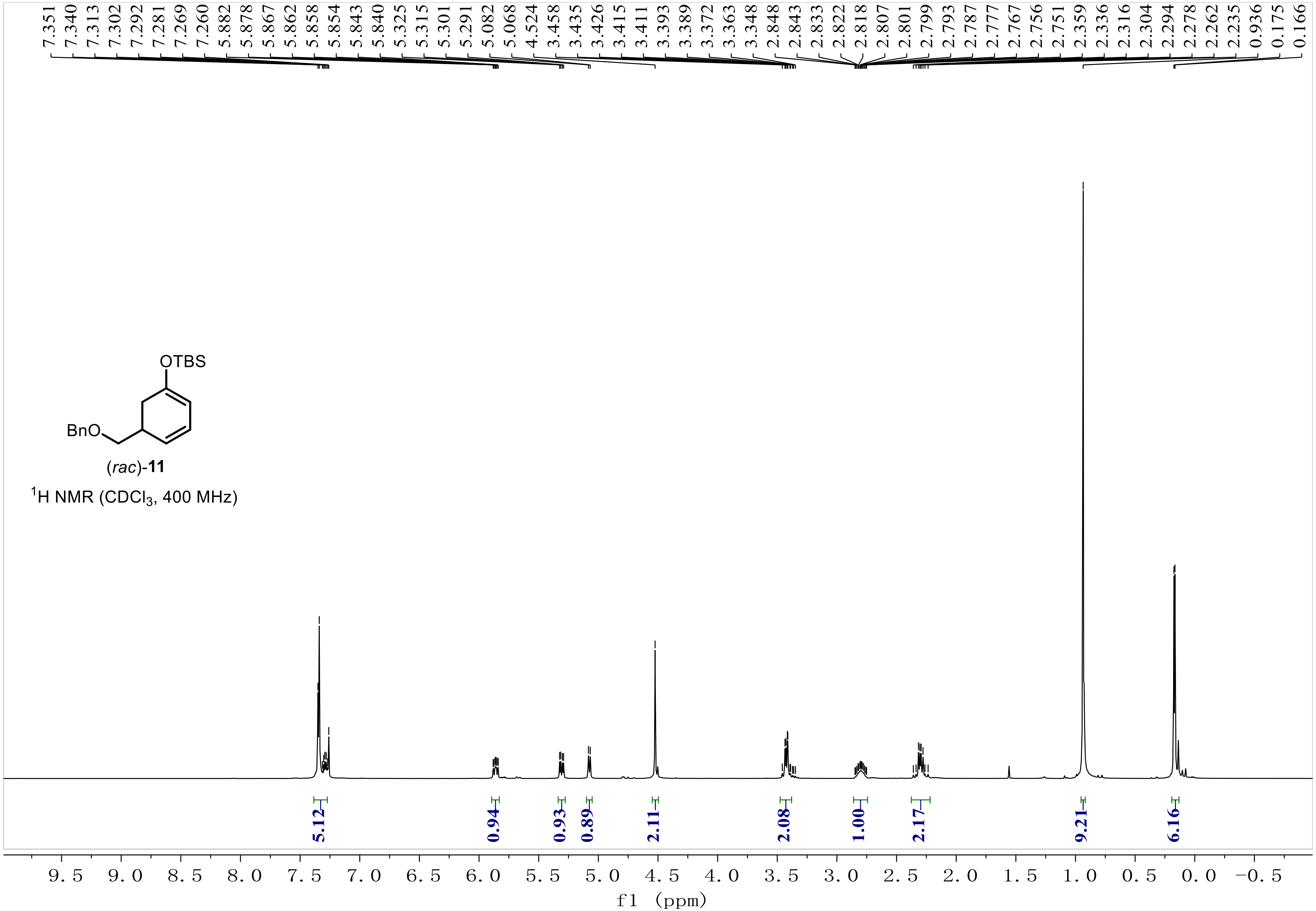
7.351 7.340 7.313 7.302 7.292 7.281 7.269 7.260 5.882 5.878 5.867 5.862 5.858 5.854 5.843 5.840 5.325 5.315 5.301 5.291 5.082 5.068 4.524 3.458 3.435 3.426 3.415 3.411 3.393 3.389 3.372 3.363 3.348 2.848 2.843 2.833 2.822 2.818 2.807 2.801 2.799 2.793 2.787 2.777 2.767 2.756 2.751 2.359 2.336 2.316 2.304 2.294 2.278 2.262 2.235 0.936 0.175 0.166
OTBS
BnO
(rac)-11
1H NMR (CDCl3, 400 MHz)
5.12 0.94 0.93 0.89 2.11 2.08 1.00 2.17 9.21 6.16
9.5 9.0 8.5 8.0 7.5 7.0 6.5 6.0 5.5 5.0 4.5 4.0 3.5 3.0 2.5 2.0 1.5 1.0 0.5 0.0 −0.5
f1 (ppm)

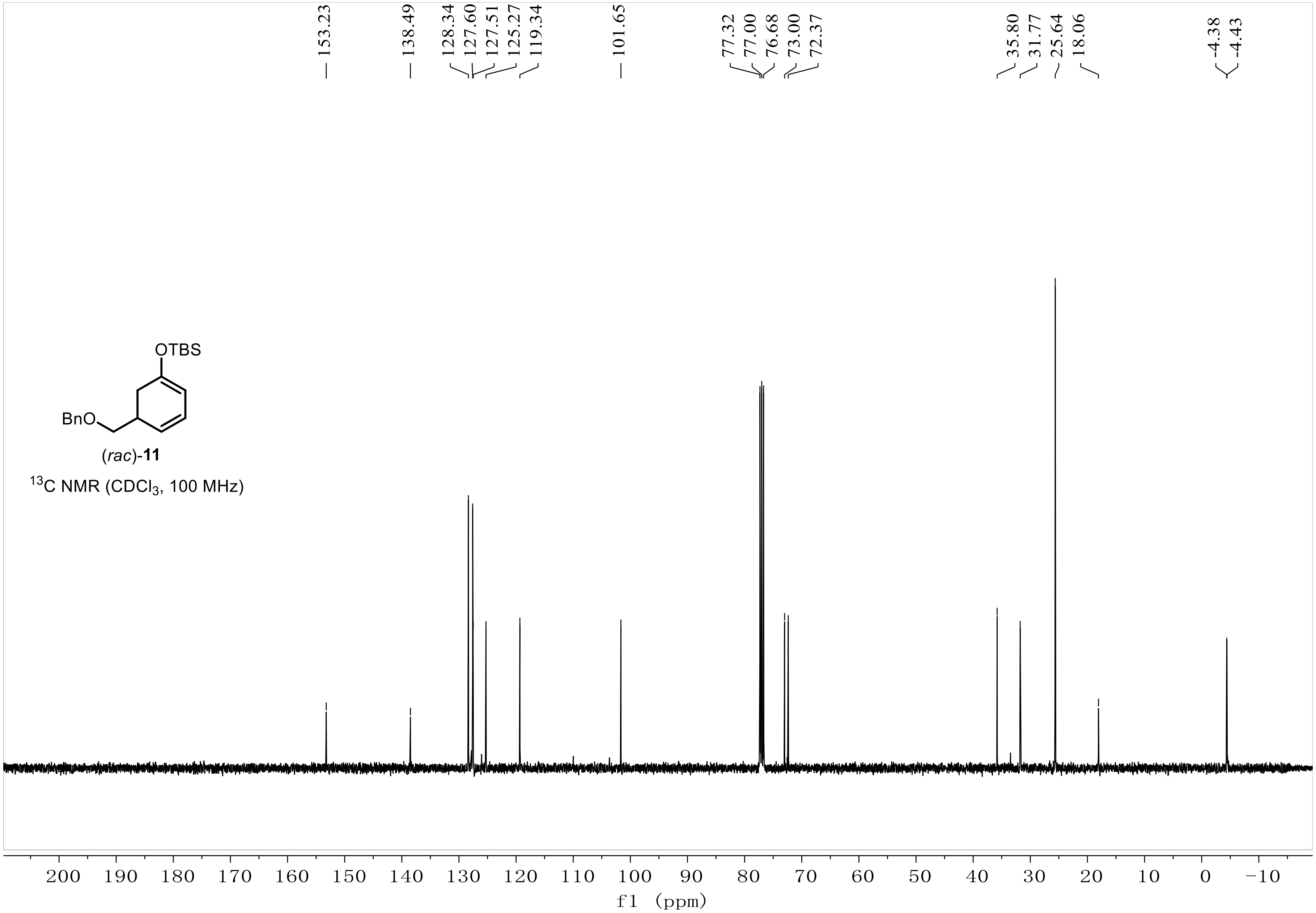
153.23
138.49
128.34
127.60
127.51
125.27
119.34
101.65
77.32
77.00
76.68
73.00
72.37
35.80
31.77
25.64
18.06
-4.38
-4.43
OTBS
BnO
(*rac*)-**11**
^{13}C NMR (CDCl$_3$, 100 MHz)
200 190 180 170 160 150 140 130 120 110 100 90 80 70 60 50 40 30 20 10 0 −10
f1 (ppm)

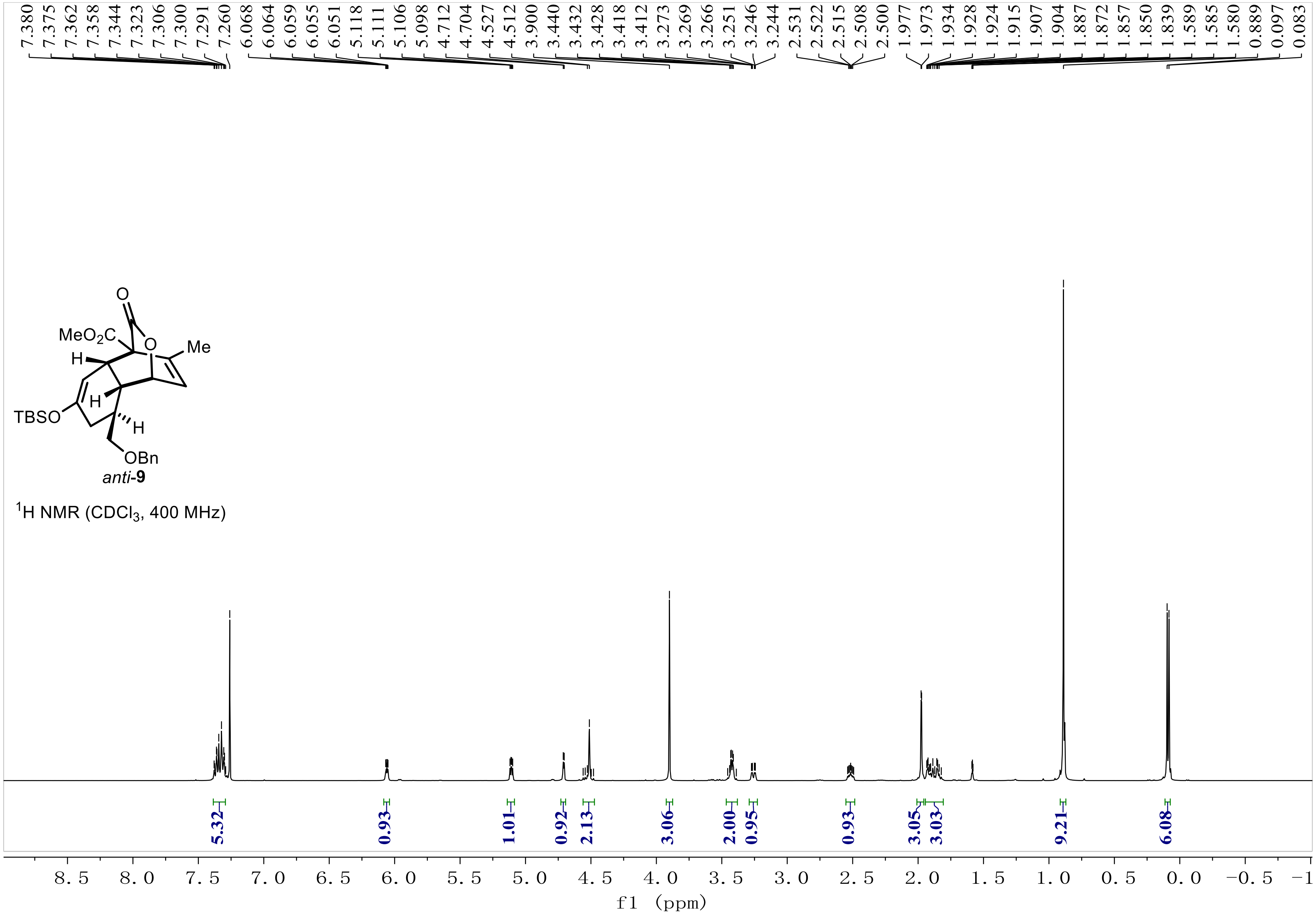
7.380
7.375
7.362
7.358
7.344
7.323
7.306
7.300
7.291
7.260
6.068
6.064
6.059
6.055
6.051
5.118
5.111
5.106
5.098
4.712
4.704
4.527
4.512
3.900
3.440
3.432
3.428
3.418
3.412
3.273
3.269
3.266
3.251
3.246
3.244
2.531
2.522
2.515
2.508
2.500
1.977
1.973
1.934
1.928
1.924
1.915
1.907
1.904
1.887
1.872
1.857
1.850
1.839
1.589
1.585
1.580
0.889
0.097
0.083
MeO2C
O
O
Me
H
H
H
TBSO
OBn
anti-9
1H NMR (CDCl3, 400 MHz)
5.32
0.93
1.01
0.92
2.13
3.06
2.00
0.95
0.93
3.05
3.03
9.21
6.08
8.5
8.0
7.5
7.0
6.5
6.0
5.5
5.0
4.5
4.0
3.5
3.0
2.5
2.0
1.5
1.0
0.5
0.0
-0.5
-1
f1 (ppm)

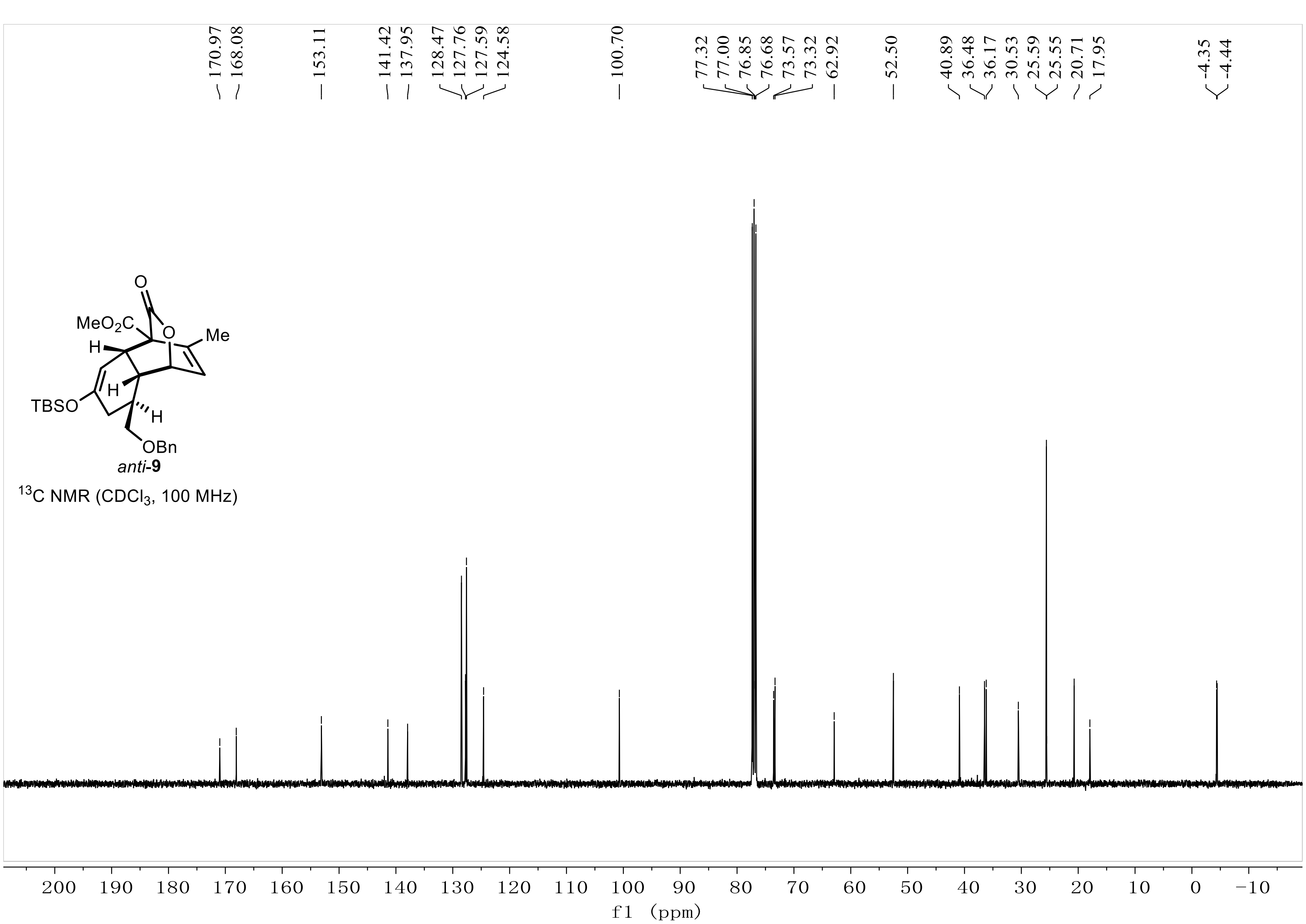
170.97
168.08
153.11
141.42
137.95
128.47
127.76
127.59
124.58
100.70
77.32
77.00
76.85
76.68
73.57
73.32
62.92
52.50
40.89
36.48
36.17
30.53
25.59
25.55
20.71
17.95
-4.35
-4.44
O
MeO2C
O
Me
H
H
H
TBSO
OBn
anti-9
13C NMR (CDCl3, 100 MHz)
200 190 180 170 160 150 140 130 120 110 100 90 80 70 60 50 40 30 20 10 0 −10
f1 (ppm)

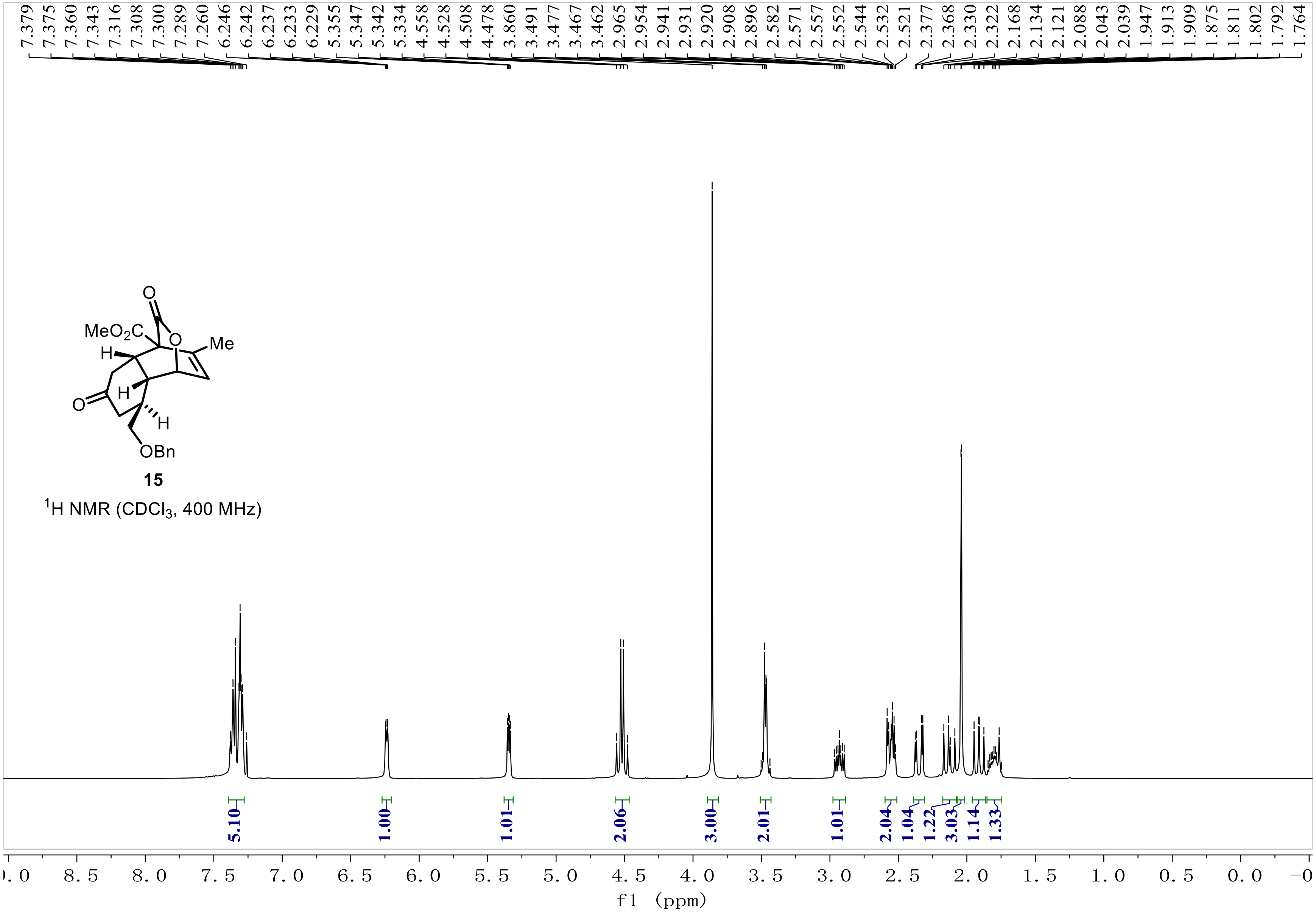

MeO2C
O
O
Me
H
H
H
O
OBn
15
1H NMR (CDCl3, 400 MHz)
7.379 7.375 7.360 7.343 7.316 7.308 7.300 7.289 7.260 6.246 6.242 6.237 6.233 6.229 5.355 5.347 5.342 5.334 4.558 4.528 4.508 4.478 3.860 3.491 3.477 3.467 3.462 2.965 2.954 2.941 2.931 2.920 2.908 2.896 2.582 2.571 2.557 2.552 2.544 2.532 2.521 2.377 2.368 2.330 2.322 2.168 2.134 2.121 2.088 2.043 2.039 1.947 1.913 1.909 1.875 1.811 1.802 1.792 1.764
5.10 1.00 1.01 2.06 3.00 2.01 1.01 2.04 1.04 1.22 3.03 1.14 1.33
f1 (ppm)

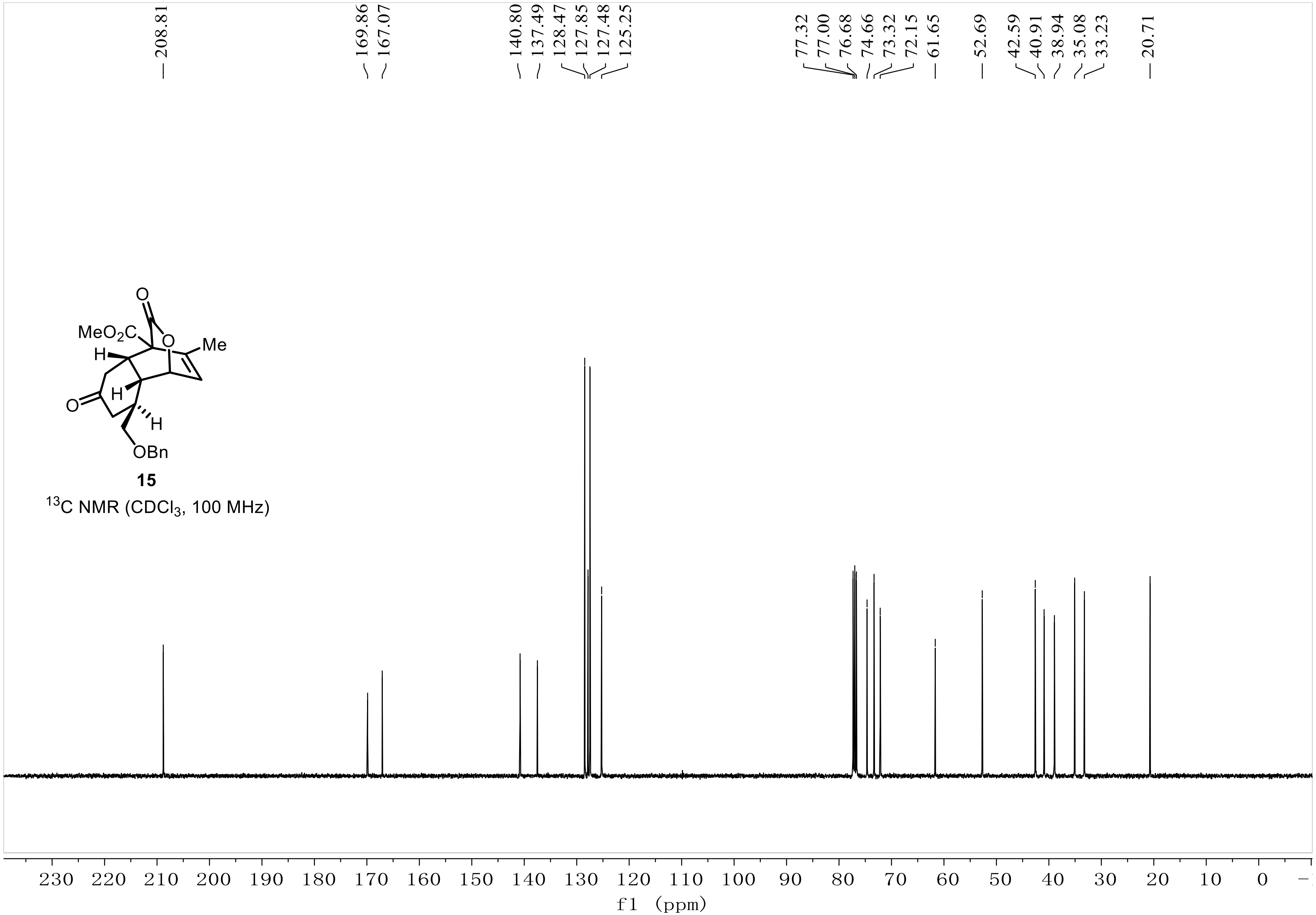

15

^{13}C NMR ($CDCl_3$, 100 MHz)

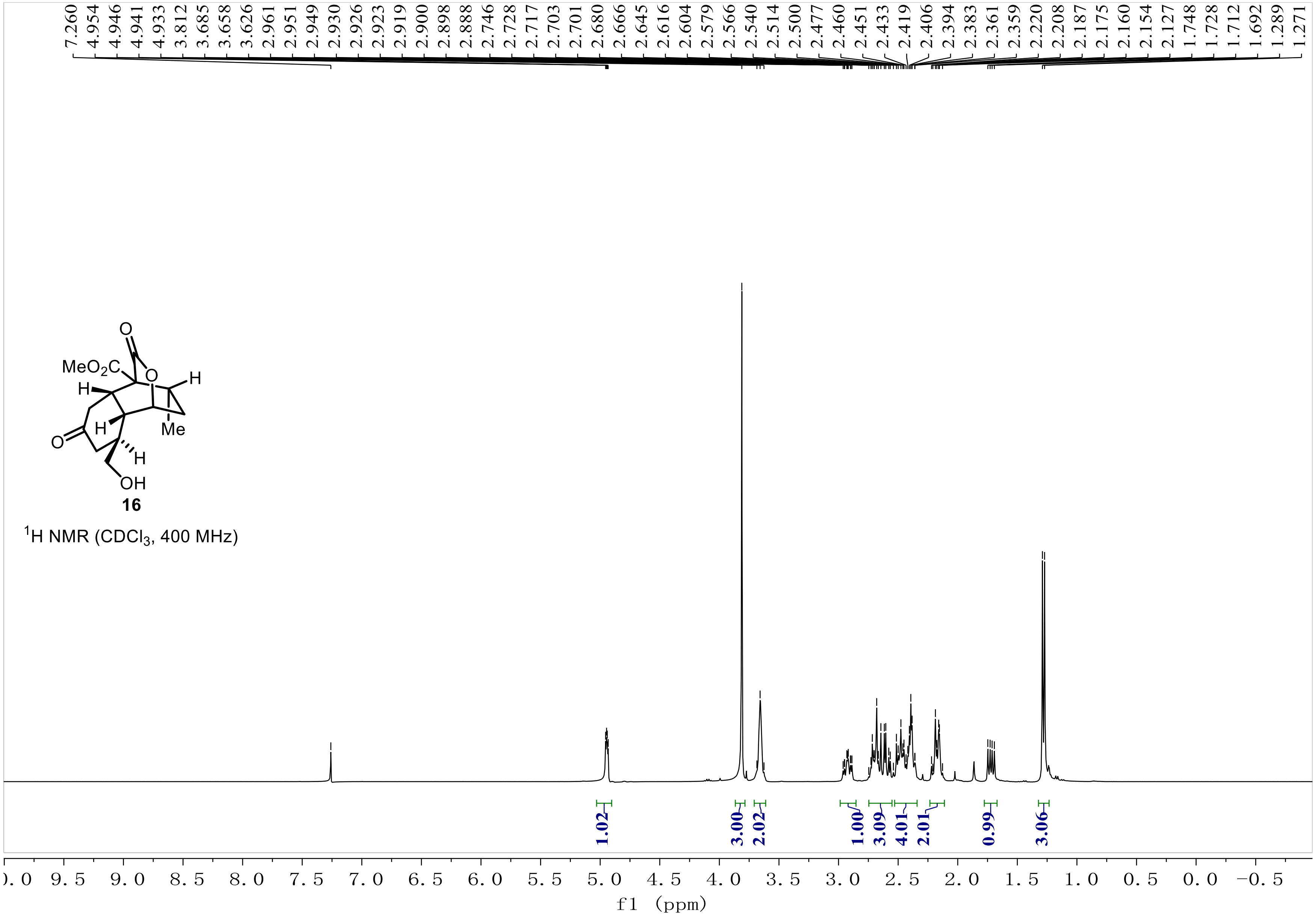

MeO2C
O
H
Me
OH
16
1H NMR (CDCl3, 400 MHz)
7.260
4.954
4.946
4.941
4.933
3.812
3.685
3.658
3.626
2.961
2.951
2.949
2.930
2.926
2.923
2.919
2.900
2.898
2.888
2.746
2.728
2.717
2.703
2.701
2.680
2.666
2.645
2.616
2.604
2.579
2.566
2.540
2.514
2.500
2.477
2.460
2.451
2.433
2.419
2.406
2.394
2.383
2.361
2.359
2.220
2.208
2.187
2.175
2.160
2.154
2.127
1.748
1.728
1.712
1.692
1.289
1.271
1.02
3.00
2.02
1.00
3.09
4.01
2.01
0.99
3.06
f1 (ppm)

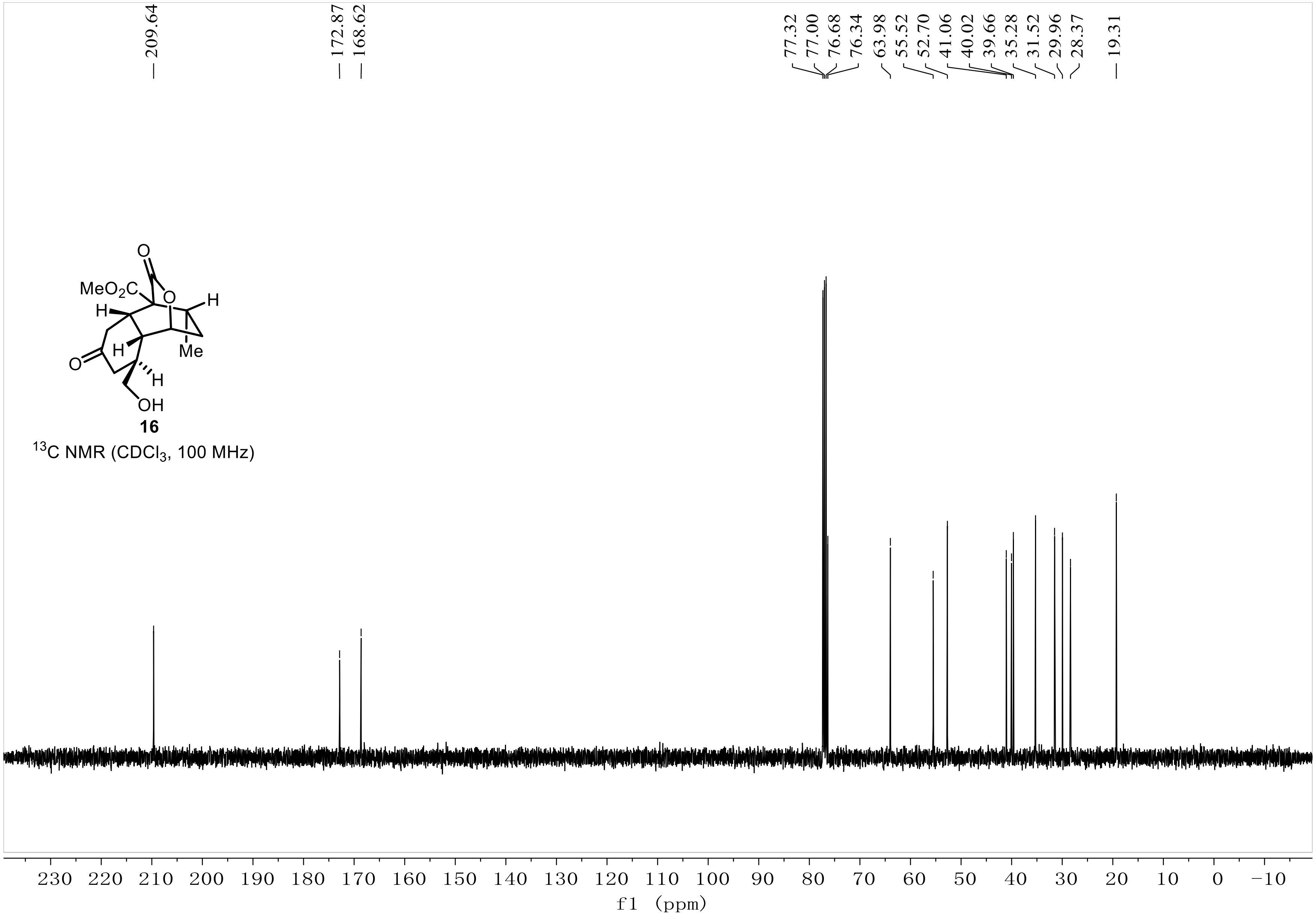
209.64
172.87
168.62
77.32
77.00
76.68
76.34
63.98
55.52
52.70
41.06
40.02
39.66
35.28
31.52
29.96
28.37
19.31
MeO2C
O
H
Me
OH
16
13C NMR (CDCl3, 100 MHz)
230 220 210 200 190 180 170 160 150 140 130 120 110 100 90 80 70 60 50 40 30 20 10 0 −10
f1 (ppm)

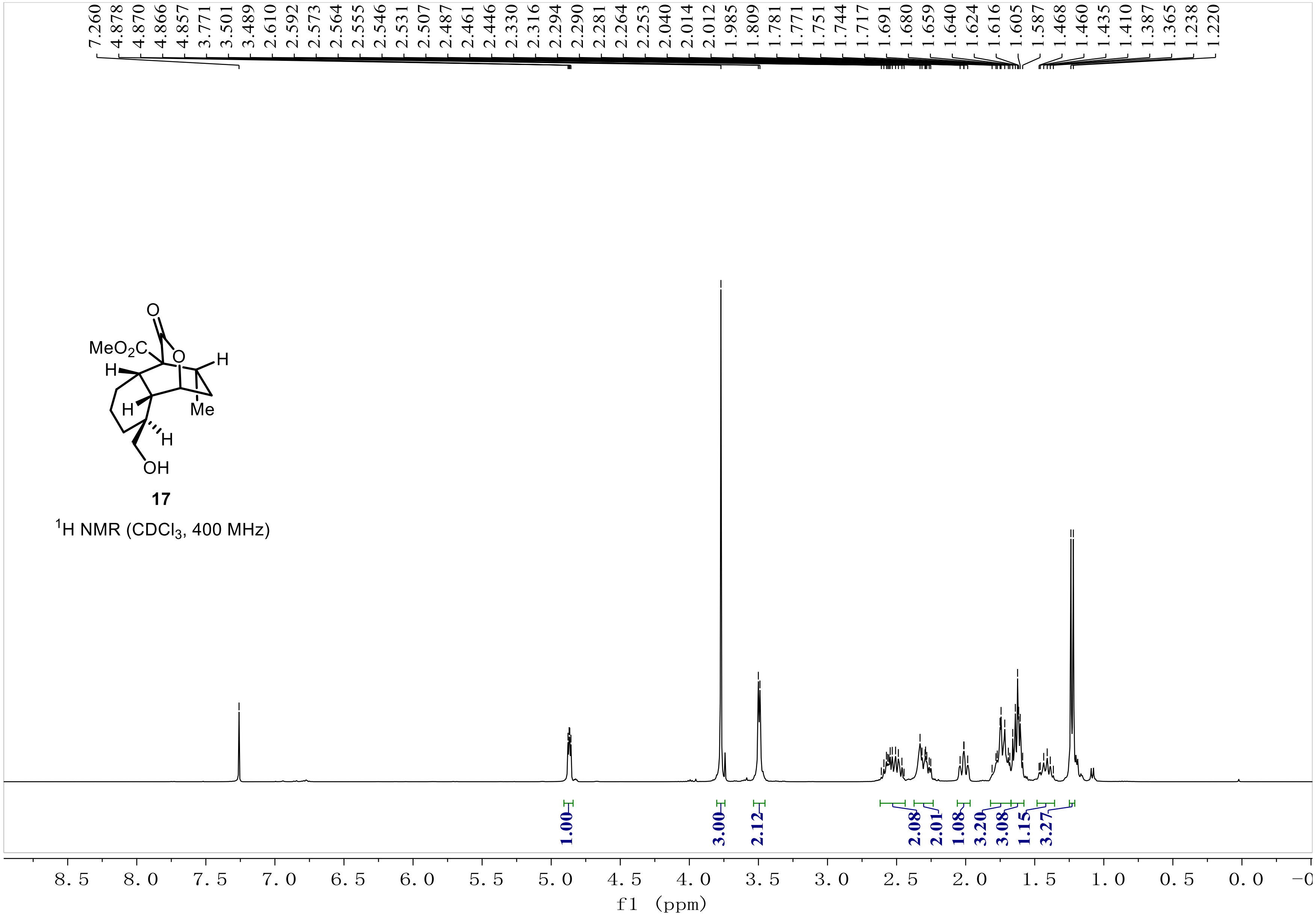

17

^{1}H NMR ($CDCl_3$, 400 MHz)

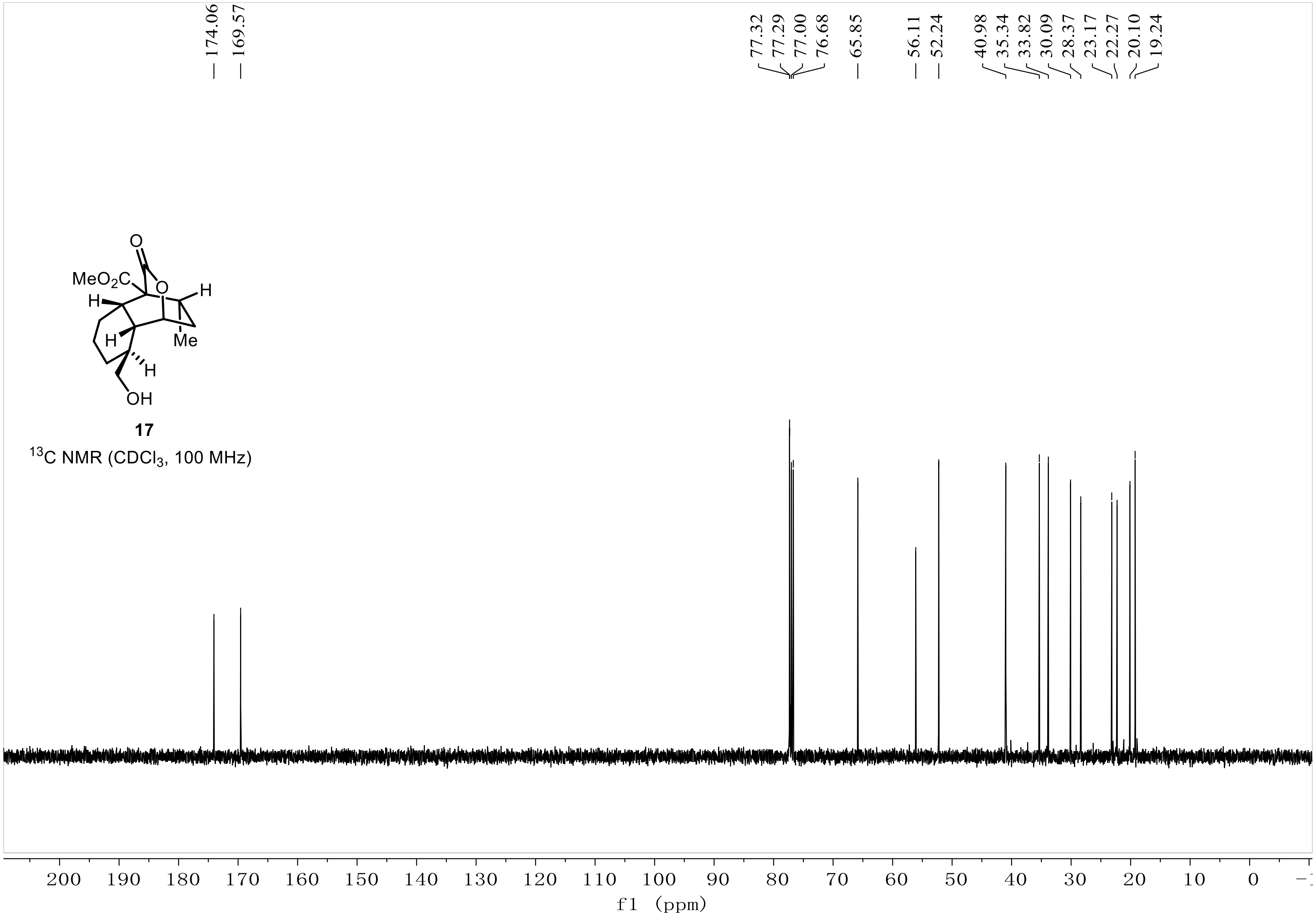
174.06
169.57
77.32
77.29
77.00
76.68
65.85
56.11
52.24
40.98
35.34
33.82
30.09
28.37
23.17
22.27
20.10
19.24
MeO2C
O
H
Me
OH
17
13C NMR (CDCl3, 100 MHz)
200 190 180 170 160 150 140 130 120 110 100 90 80 70 60 50 40 30 20 10 0
f1 (ppm)

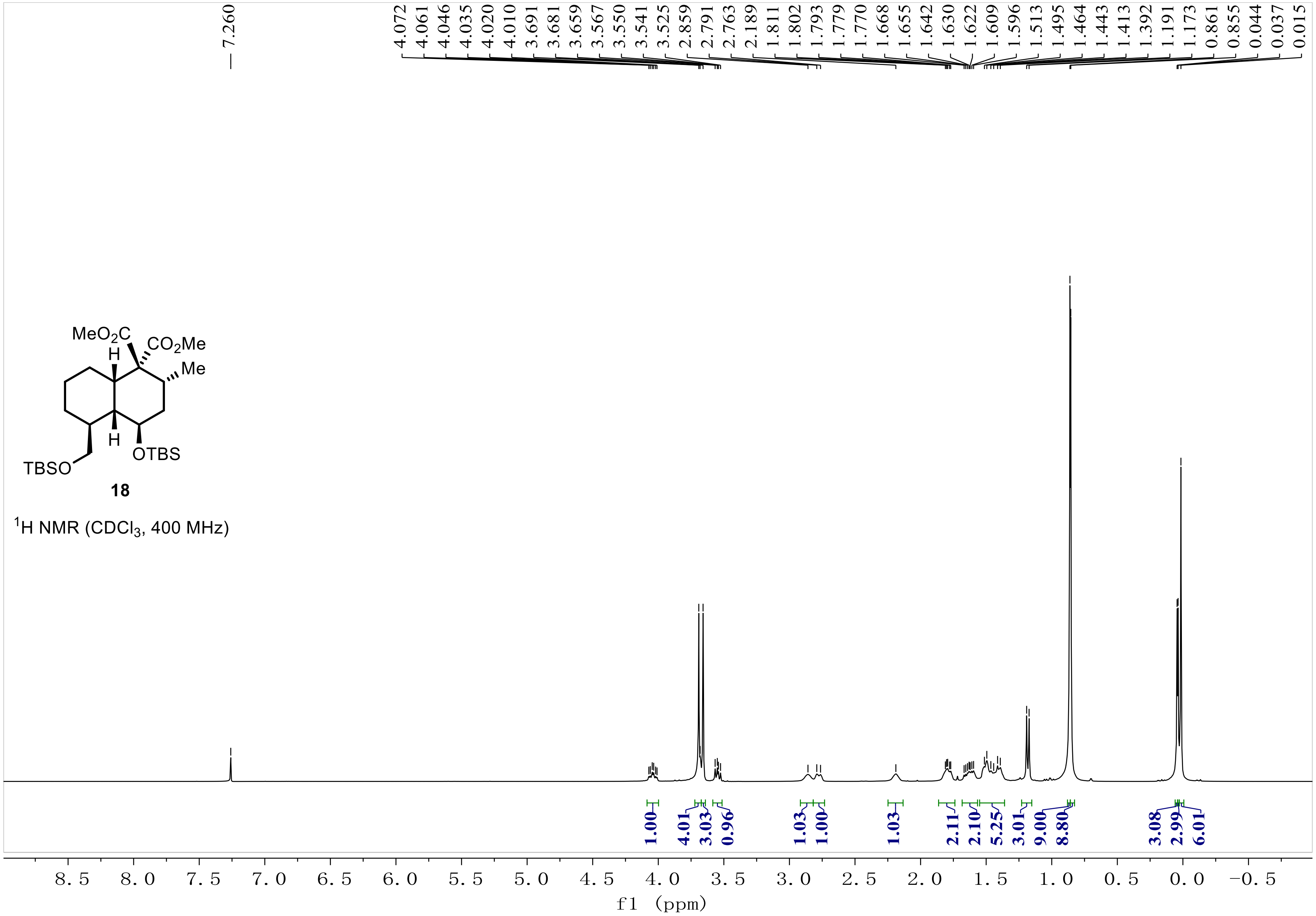

MeO2C
CO2Me
Me
H
H
OTBS
TBSO
18
1H NMR (CDCl3, 400 MHz)
f1 (ppm)

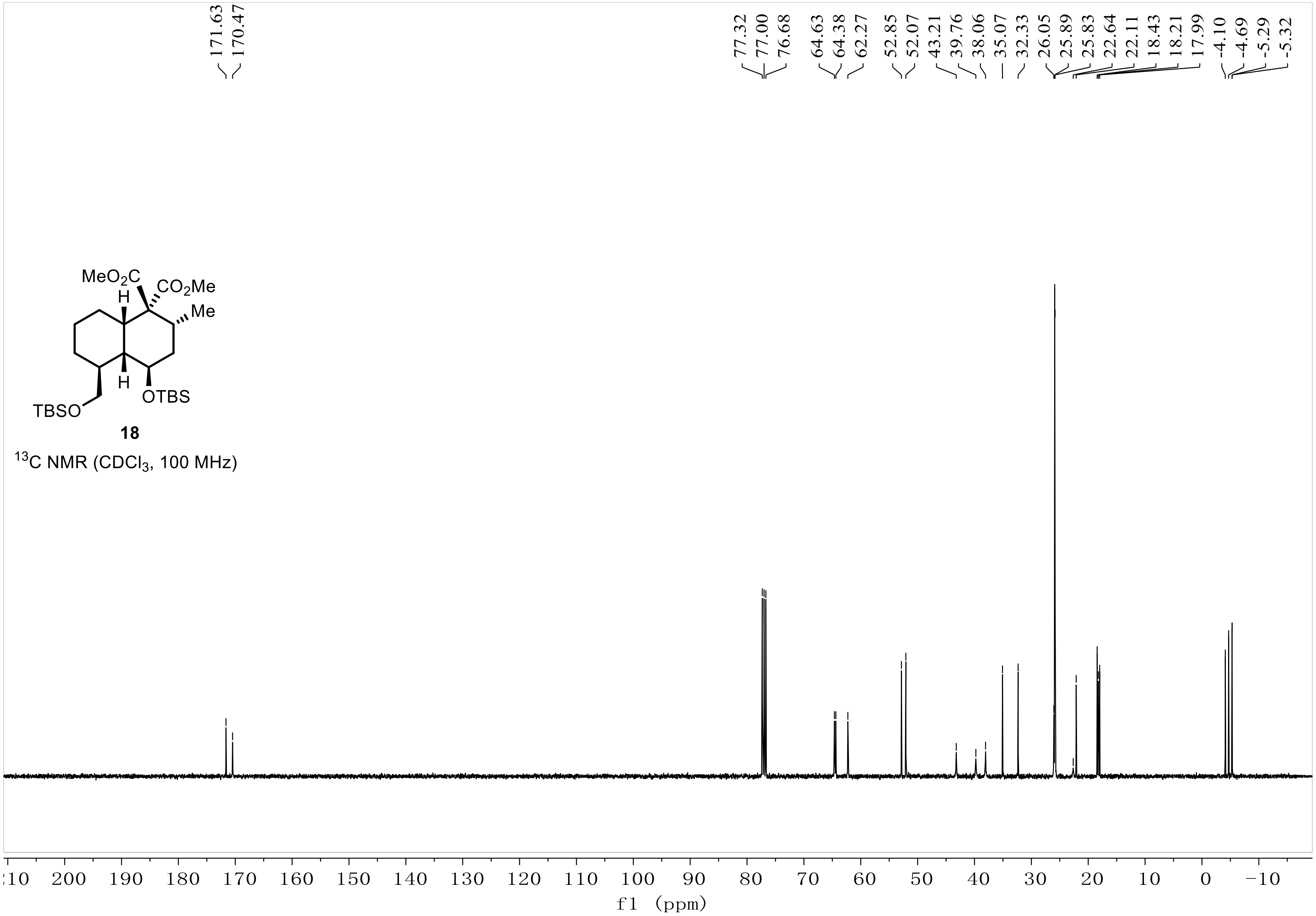
171.63
170.47
77.32
77.00
76.68
64.63
64.38
62.27
52.85
52.07
43.21
39.76
38.06
35.07
32.33
26.05
25.89
25.83
22.64
22.11
18.43
18.21
17.99
-4.10
-4.69
-5.29
-5.32
MeO2C
CO2Me
H
Me
H
OTBS
TBSO
18
13C NMR (CDCl3, 100 MHz)
f1 (ppm)

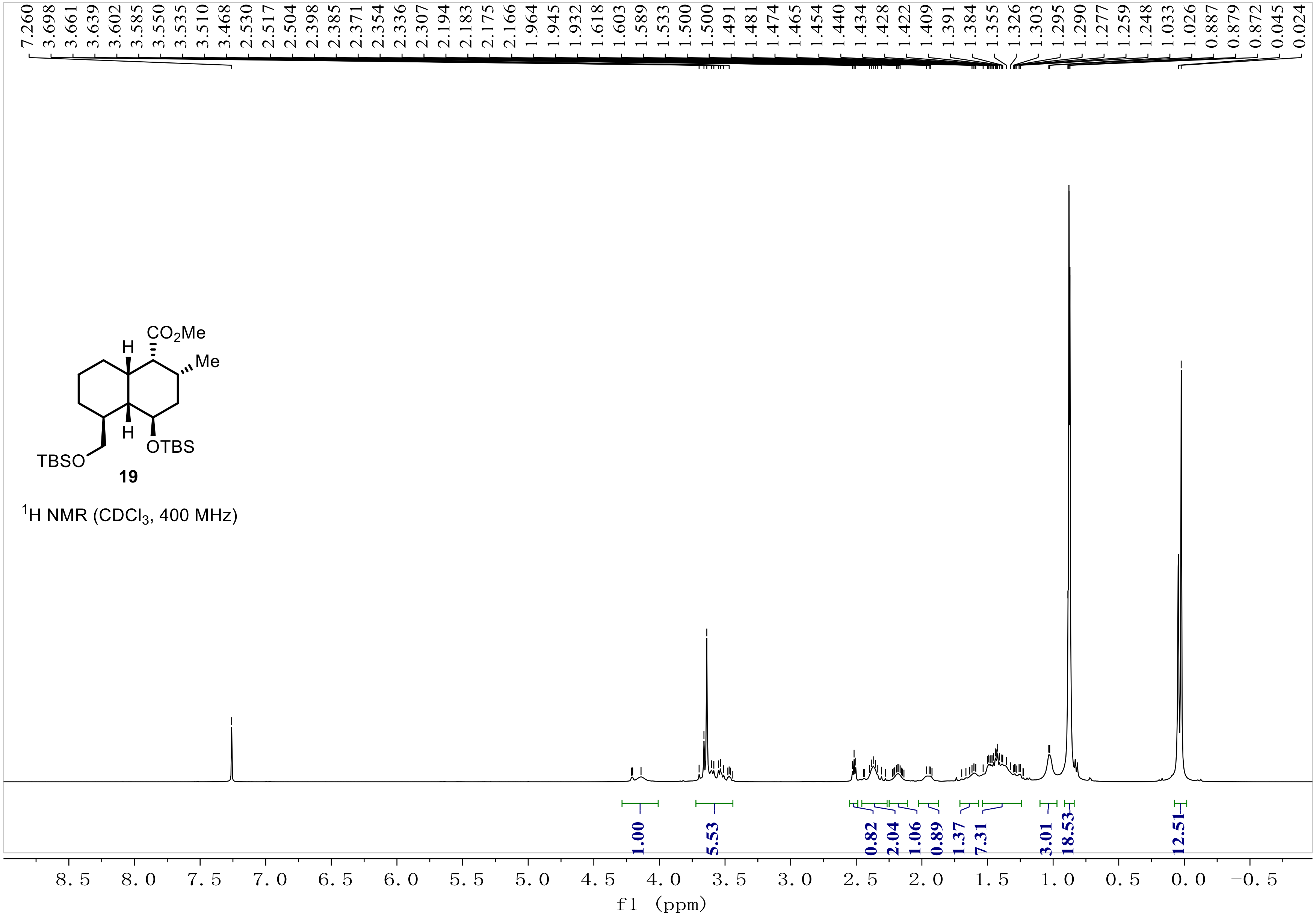

7.260
3.698
3.661
3.639
3.602
3.585
3.550
3.535
3.510
3.468
2.530
2.517
2.504
2.398
2.385
2.371
2.354
2.336
2.307
2.194
2.183
2.175
2.166
1.964
1.945
1.932
1.618
1.603
1.589
1.533
1.500
1.500
1.491
1.481
1.474
1.465
1.454
1.440
1.434
1.428
1.422
1.409
1.391
1.384
1.355
1.326
1.303
1.295
1.290
1.277
1.259
1.248
1.033
1.026
0.887
0.879
0.872
0.045
0.024
CO2Me
H
Me
H
OTBS
TBSO
19
1H NMR (CDCl3, 400 MHz)
1.00
5.53
0.82
2.04
1.06
0.89
1.37
7.31
3.01
18.53
12.51
8.5
8.0
7.5
7.0
6.5
6.0
5.5
5.0
4.5
4.0
3.5
3.0
2.5
2.0
1.5
1.0
0.5
0.0
-0.5
f1 (ppm)

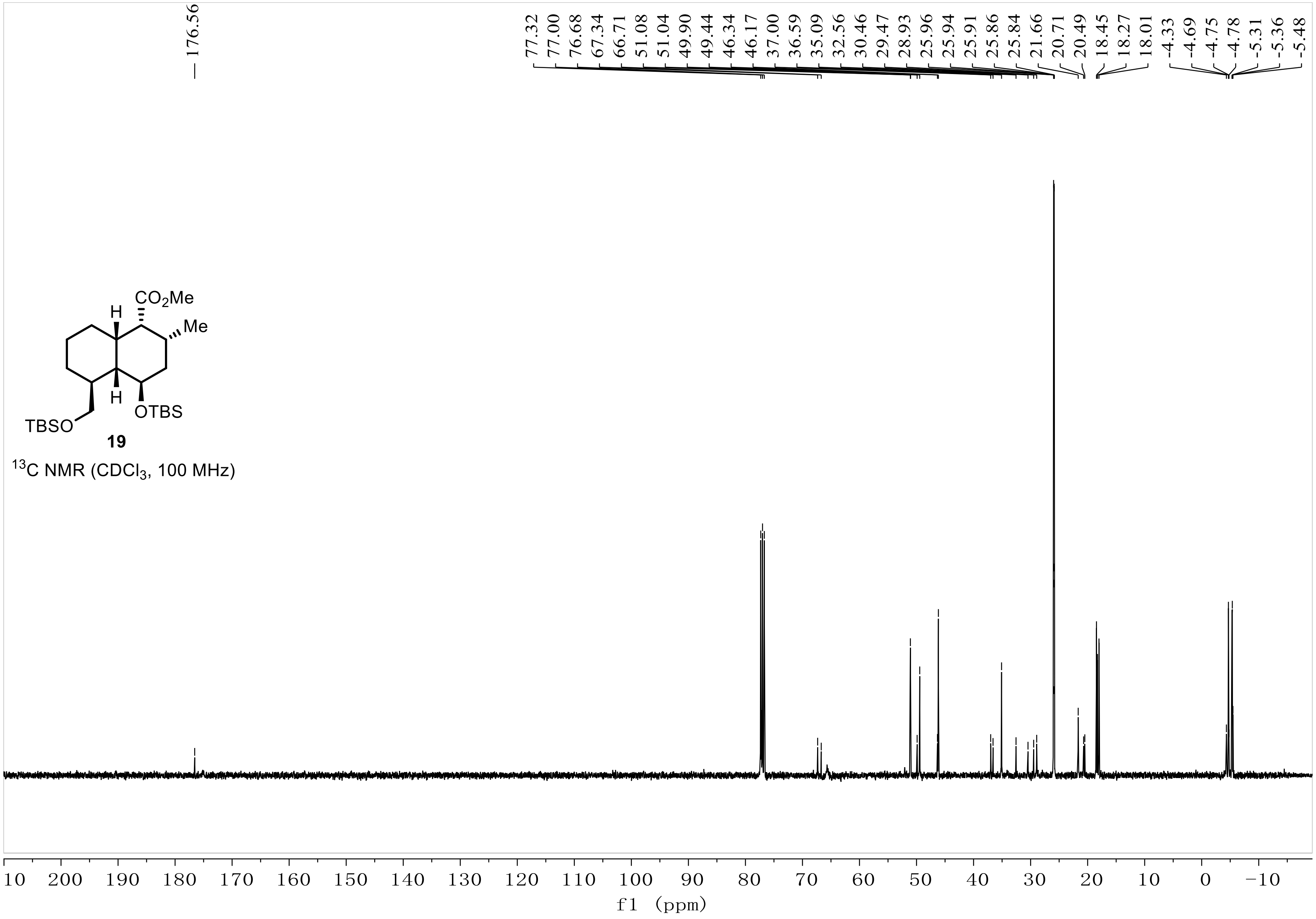

176.56
77.32
77.00
76.68
67.34
66.71
51.08
51.04
49.90
49.44
46.34
46.17
37.00
36.59
35.09
32.56
30.46
29.47
28.93
25.96
25.94
25.91
25.86
25.84
21.66
20.71
20.49
18.45
18.27
18.01
-4.33
-4.69
-4.75
-4.78
-5.31
-5.36
-5.48
CO2Me
H
Me
H
OTBS
TBSO
19
13C NMR (CDCl3, 100 MHz)
f1 (ppm)

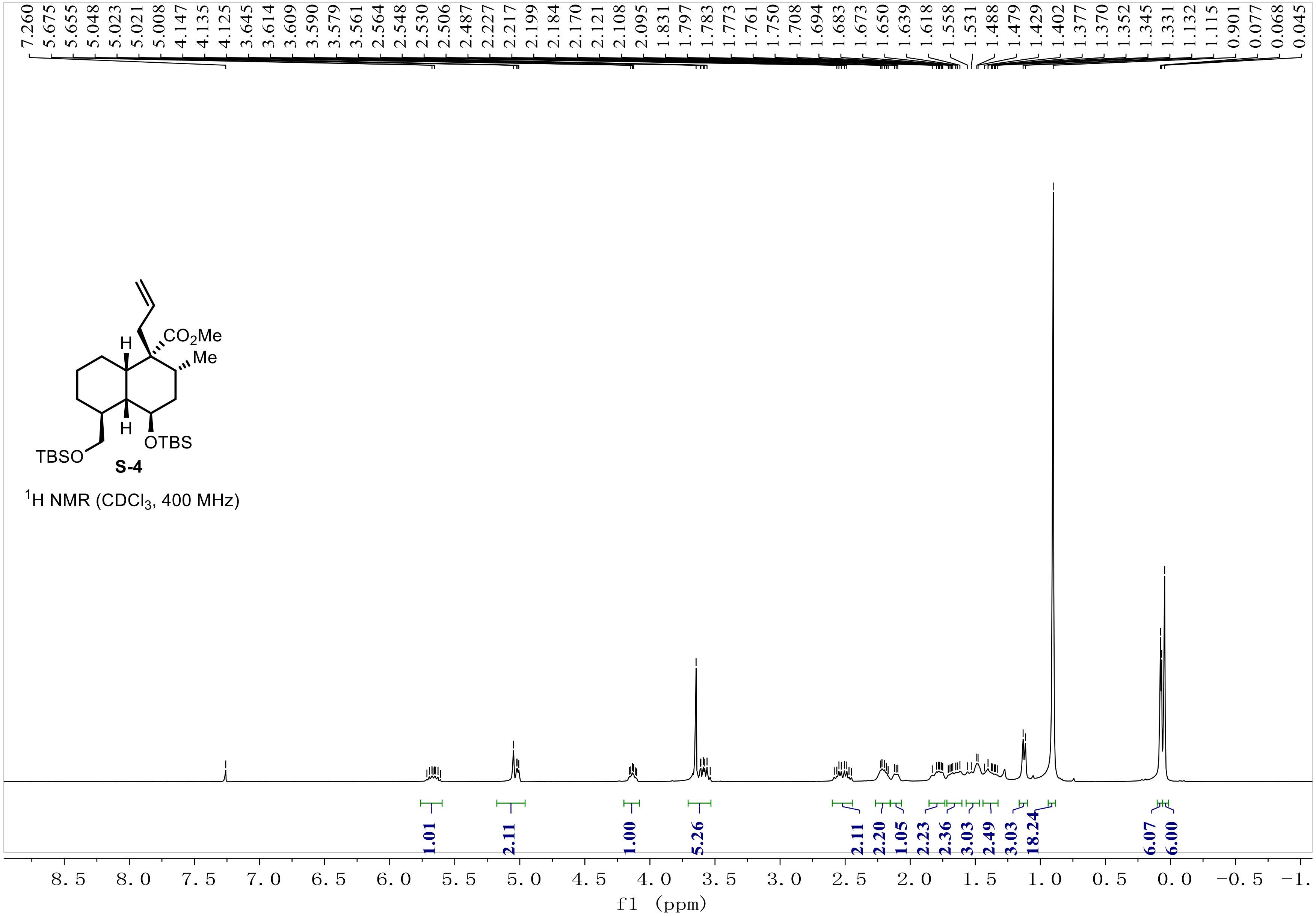
7.260 5.675 5.655 5.048 5.023 5.021 5.008 4.147 4.135 4.125 3.645 3.614 3.609 3.590 3.579 3.561 2.564 2.548 2.530 2.506 2.487 2.227 2.217 2.199 2.184 2.170 2.121 2.108 2.095 1.831 1.797 1.783 1.773 1.761 1.750 1.708 1.694 1.683 1.673 1.650 1.639 1.618 1.558 1.531 1.488 1.479 1.429 1.402 1.377 1.370 1.352 1.345 1.331 1.132 1.115 0.901 0.077 0.068 0.045
CO2Me
Me
H
H
OTBS
TBSO
S-4
1H NMR (CDCl3, 400 MHz)
1.01 2.11 1.00 5.26 2.11 2.20 1.05 2.23 2.36 3.03 2.49 3.03 18.24 6.07 6.00
8.5 8.0 7.5 7.0 6.5 6.0 5.5 5.0 4.5 4.0 3.5 3.0 2.5 2.0 1.5 1.0 0.5 0.0 -0.5 -1.
f1 (ppm)

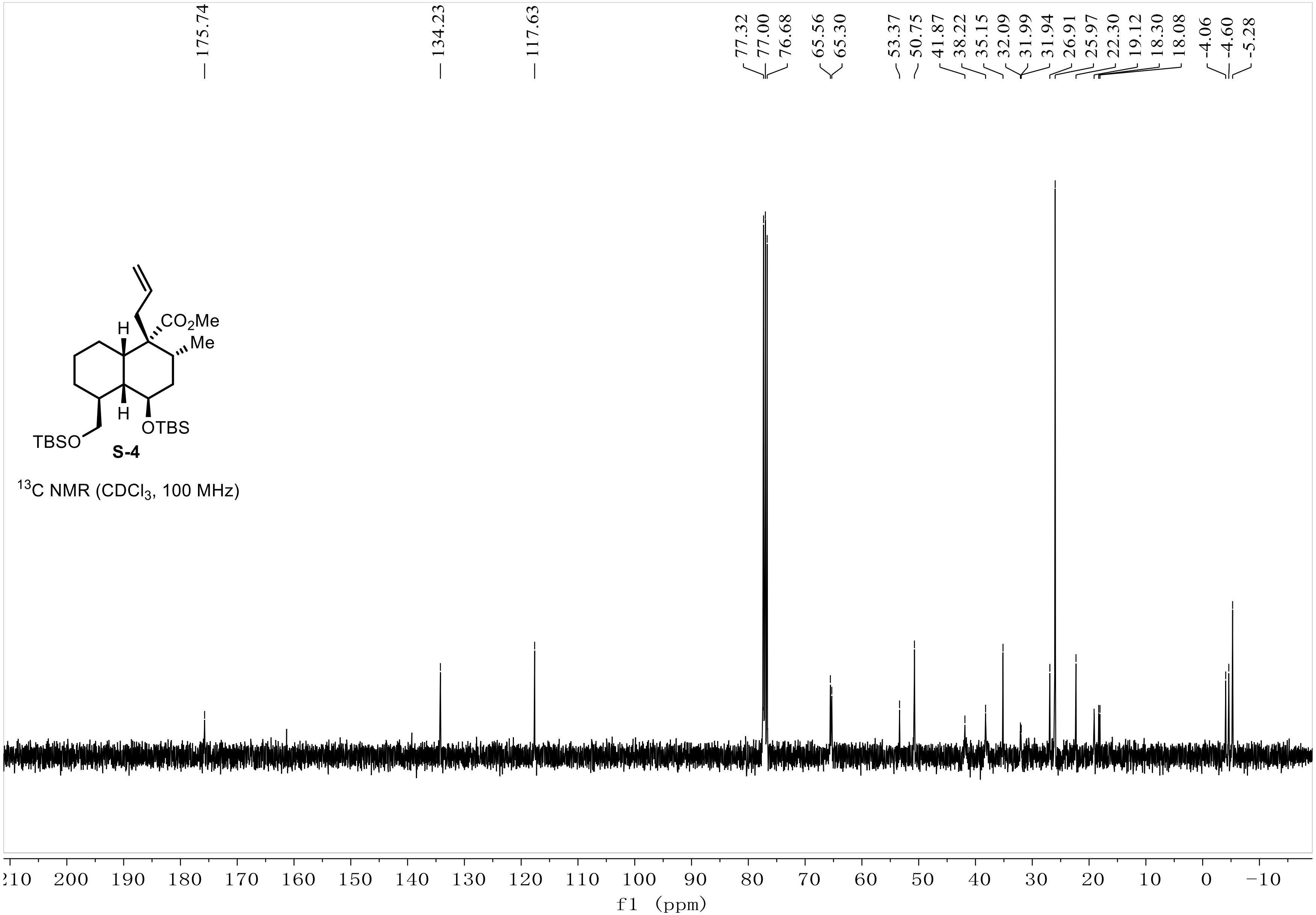
175.74
134.23
117.63
77.32
77.00
76.68
65.56
65.30
53.37
50.75
41.87
38.22
35.15
32.09
31.99
31.94
26.91
25.97
22.30
19.12
18.30
18.08
-4.06
-4.60
-5.28
CO2Me
Me
H
H
OTBS
TBSO
S-4
13C NMR (CDCl3, 100 MHz)
210 200 190 180 170 160 150 140 130 120 110 100 90 80 70 60 50 40 30 20 10 0 -10
f1 (ppm)

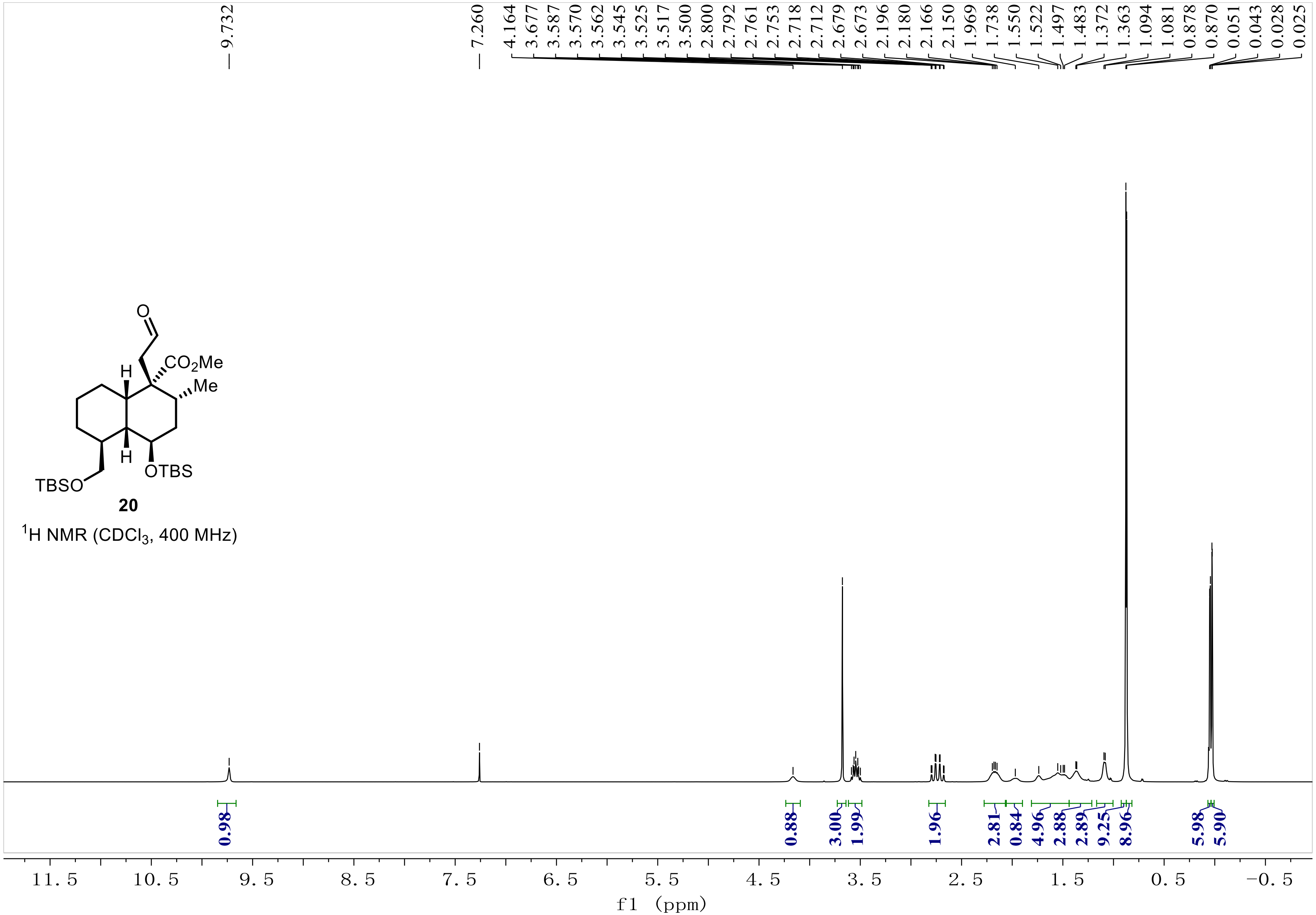
9.732
7.260
4.164 3.677 3.587 3.570 3.562 3.545 3.525 3.517 3.500 2.800 2.792 2.761 2.753 2.718 2.712 2.679 2.673 2.196 2.180 2.166 2.150 1.969 1.738 1.550 1.522 1.497 1.483 1.372 1.363 1.094 1.081 0.878 0.870 0.051 0.043 0.028 0.025
O
CO2Me
H
Me
H
OTBS
TBSO
20
1H NMR (CDCl3, 400 MHz)
0.98 0.88 3.00 1.99 1.96 2.81 0.84 4.96 2.88 2.89 9.25 8.96 5.98 5.90
11.5 10.5 9.5 8.5 7.5 6.5 5.5 4.5 3.5 2.5 1.5 0.5 −0.5
f1 (ppm)

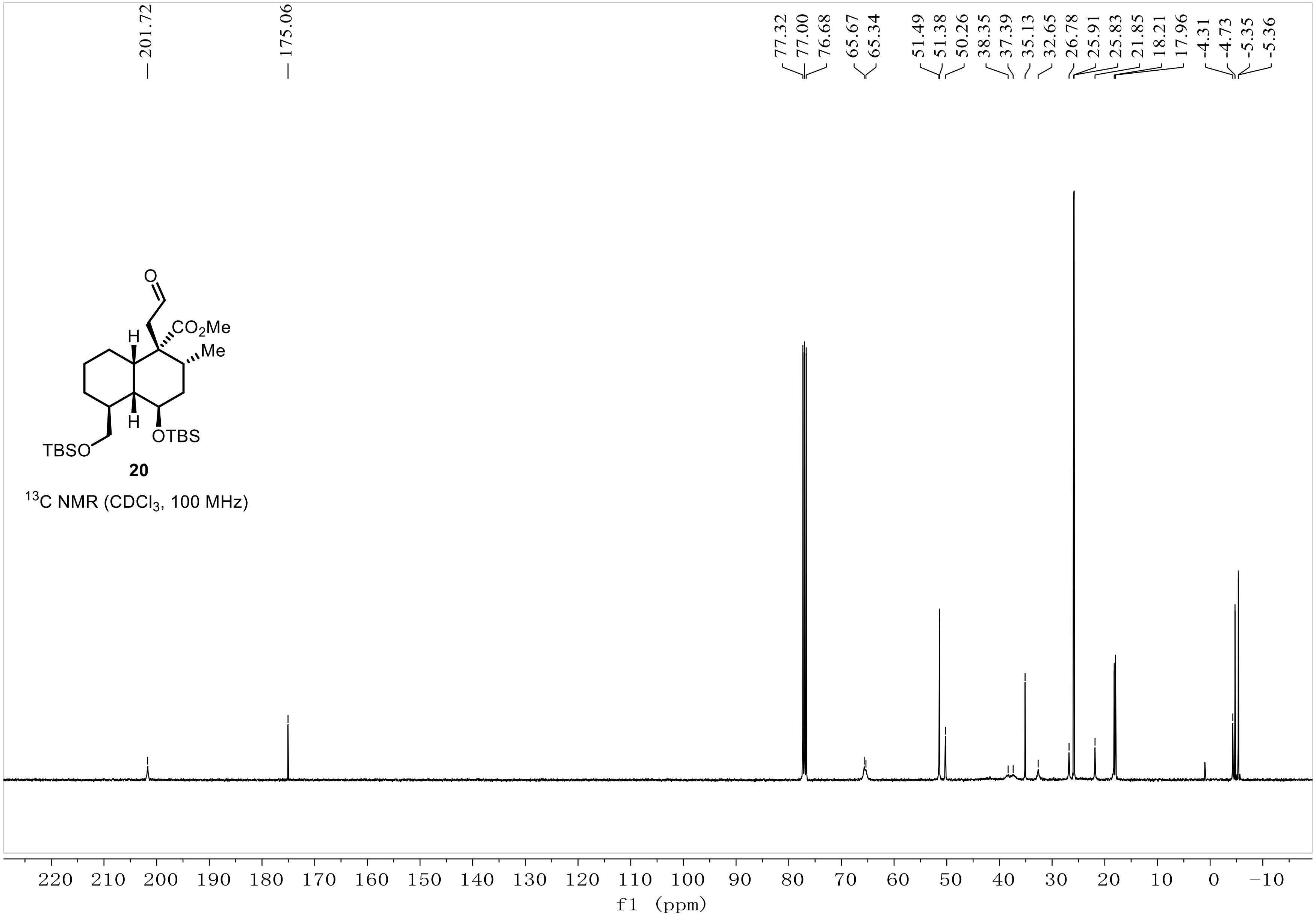
201.72
175.06
77.32
77.00
76.68
65.67
65.34
51.49
51.38
50.26
38.35
37.39
35.13
32.65
26.78
25.91
25.83
21.85
18.21
17.96
-4.31
-4.73
-5.35
-5.36
CO2Me
Me
OTBS
TBSO
20
13C NMR (CDCl3, 100 MHz)
220 210 200 190 180 170 160 150 140 130 120 110 100 90 80 70 60 50 40 30 20 10 0 −10
f1 (ppm)

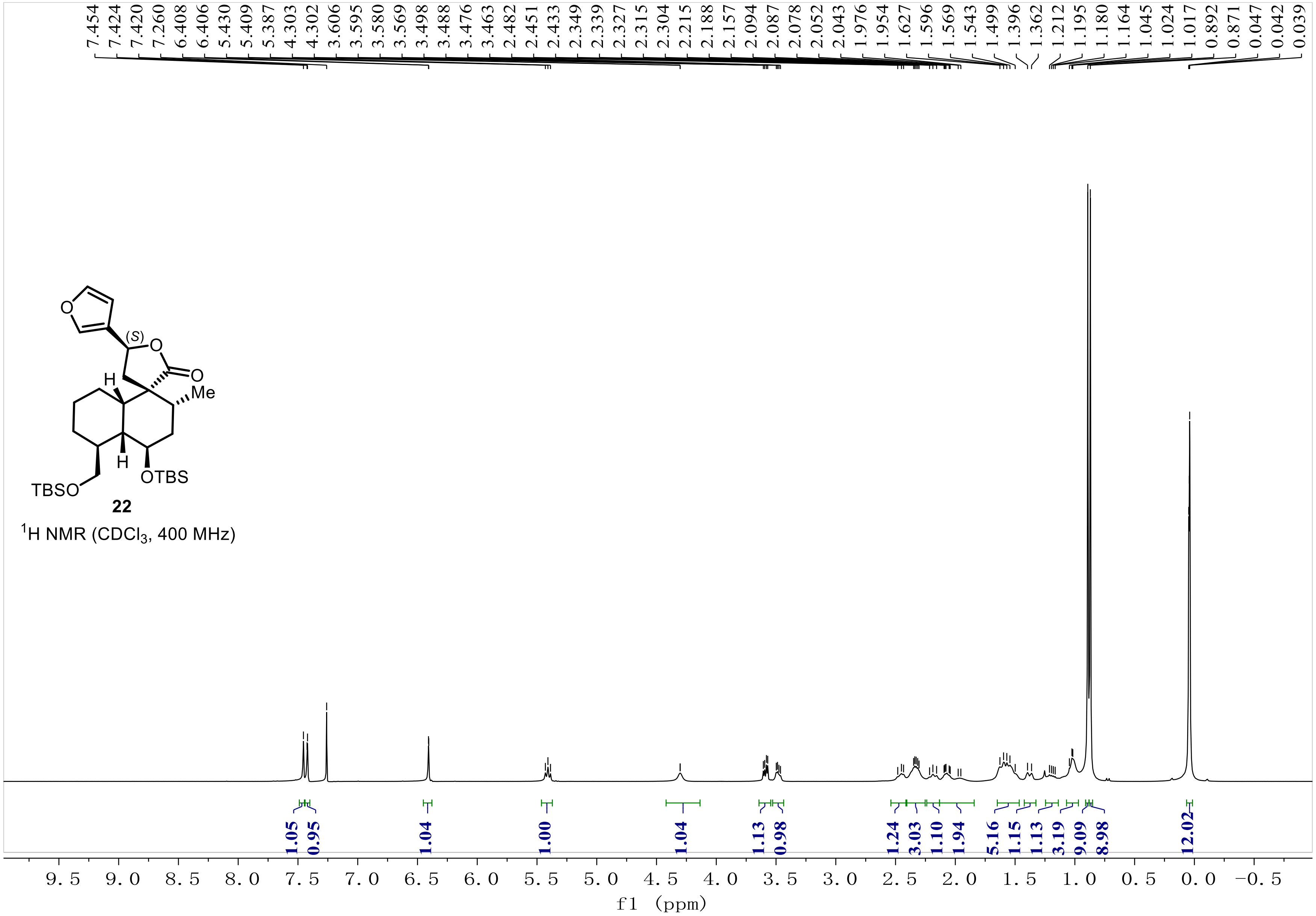
7.454 7.424 7.420 7.260 6.408 6.406 5.430 5.409 5.387 4.303 4.302 3.606 3.595 3.580 3.569 3.498 3.488 3.476 3.463 2.482 2.451 2.433 2.349 2.339 2.327 2.315 2.304 2.215 2.188 2.157 2.094 2.087 2.078 2.052 2.043 1.976 1.954 1.627 1.596 1.569 1.543 1.499 1.396 1.362 1.212 1.195 1.180 1.164 1.045 1.024 1.017 0.892 0.871 0.047 0.042 0.039
O
(S)
O
O
H
Me
H
OTBS
TBSO
22
1H NMR (CDCl3, 400 MHz)
1.05 0.95 1.04 1.00 1.04 1.13 0.98 1.24 3.03 1.10 1.94 5.16 1.15 1.13 3.19 9.09 8.98 12.02
9.5 9.0 8.5 8.0 7.5 7.0 6.5 6.0 5.5 5.0 4.5 4.0 3.5 3.0 2.5 2.0 1.5 1.0 0.5 0.0 -0.5
f1 (ppm)

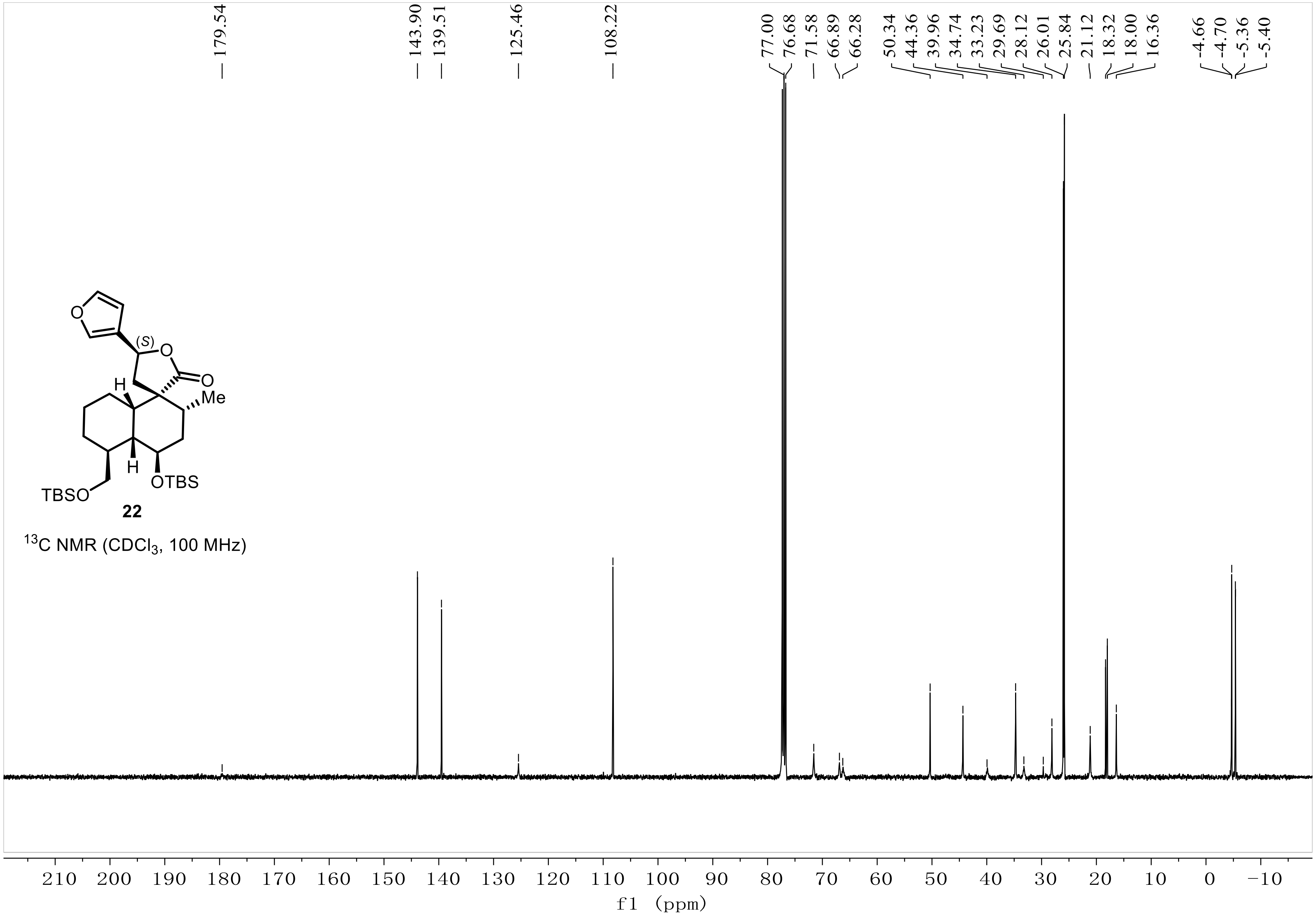
179.54
143.90
139.51
125.46
108.22
77.00
76.68
71.58
66.89
66.28
50.34
44.36
39.96
34.74
33.23
29.69
28.12
26.01
25.84
21.12
18.32
18.00
16.36
-4.66
-4.70
-5.36
-5.40
O
(S)
O
O
H
Me
H
OTBS
TBSO
22
13C NMR (CDCl3, 100 MHz)
210 200 190 180 170 160 150 140 130 120 110 100 90 80 70 60 50 40 30 20 10 0 −10
f1 (ppm)

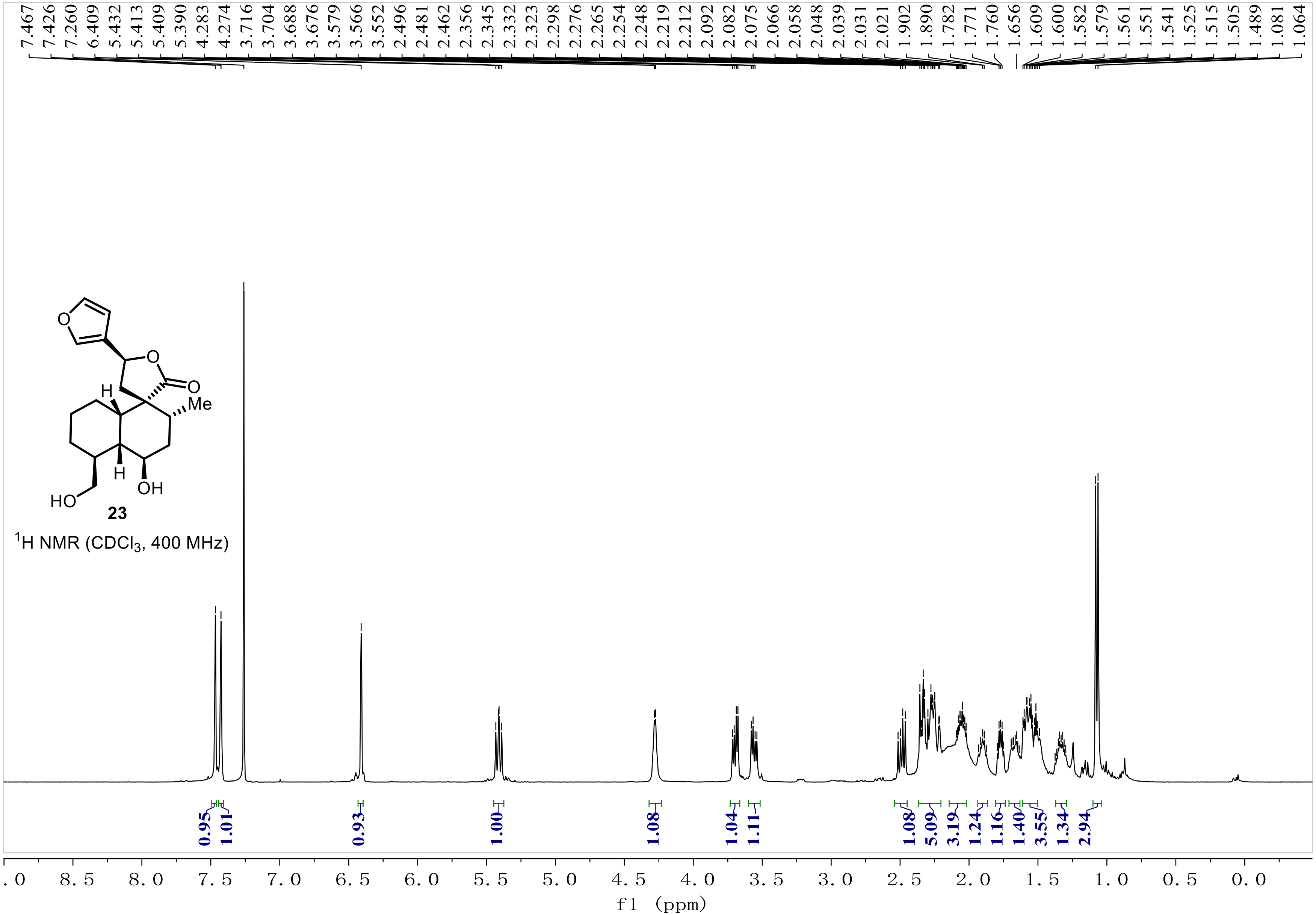

7.467 7.426 7.260 6.409 5.432 5.413 5.409 5.390 4.283 4.274 3.716 3.704 3.688 3.676 3.579 3.566 3.552 2.496 2.481 2.462 2.356 2.345 2.332 2.323 2.298 2.276 2.265 2.254 2.248 2.219 2.212 2.092 2.082 2.075 2.066 2.058 2.048 2.039 2.031 2.021 1.902 1.890 1.782 1.771 1.760 1.656 1.609 1.600 1.582 1.579 1.561 1.551 1.541 1.525 1.515 1.505 1.489 1.081 1.064
O
O
O
H
Me
H
OH
HO
23
1H NMR (CDCl3, 400 MHz)
0.95 1.01 0.93 1.00 1.08 1.04 1.11 1.08 5.09 3.19 1.24 1.16 1.40 3.55 1.34 2.94
.0 8.5 8.0 7.5 7.0 6.5 6.0 5.5 5.0 4.5 4.0 3.5 3.0 2.5 2.0 1.5 1.0 0.5 0.0
f1 (ppm)

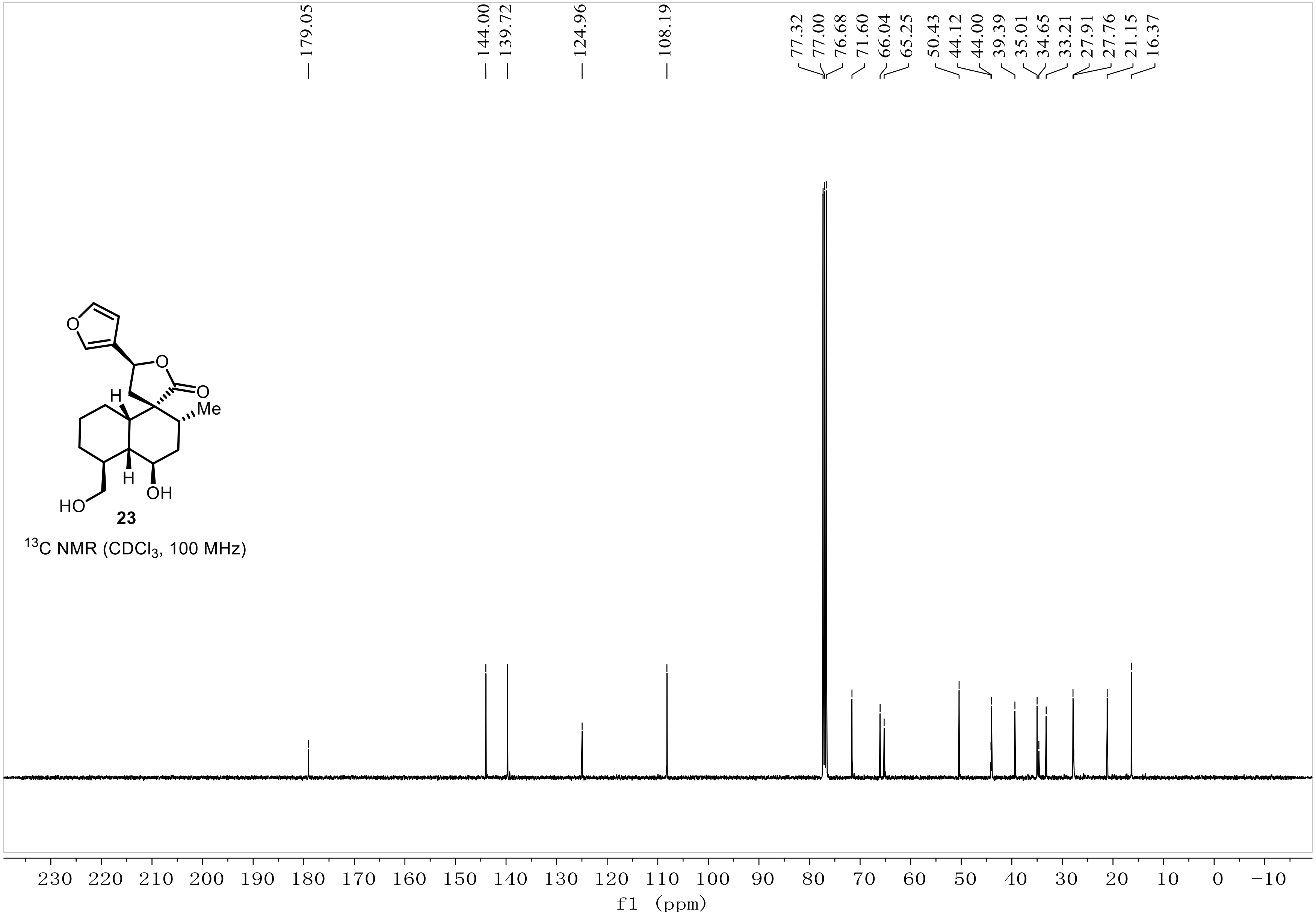
179.05
144.00
139.72
124.96
108.19
77.32
77.00
76.68
71.60
66.04
65.25
50.43
44.12
44.00
39.39
35.01
34.65
33.21
27.91
27.76
21.15
16.37
O
O
O
H
Me
H
OH
HO
23
^{13}C NMR (CDCl$_3$, 100 MHz)
230 220 210 200 190 180 170 160 150 140 130 120 110 100 90 80 70 60 50 40 30 20 10 0 −10
f1 (ppm)

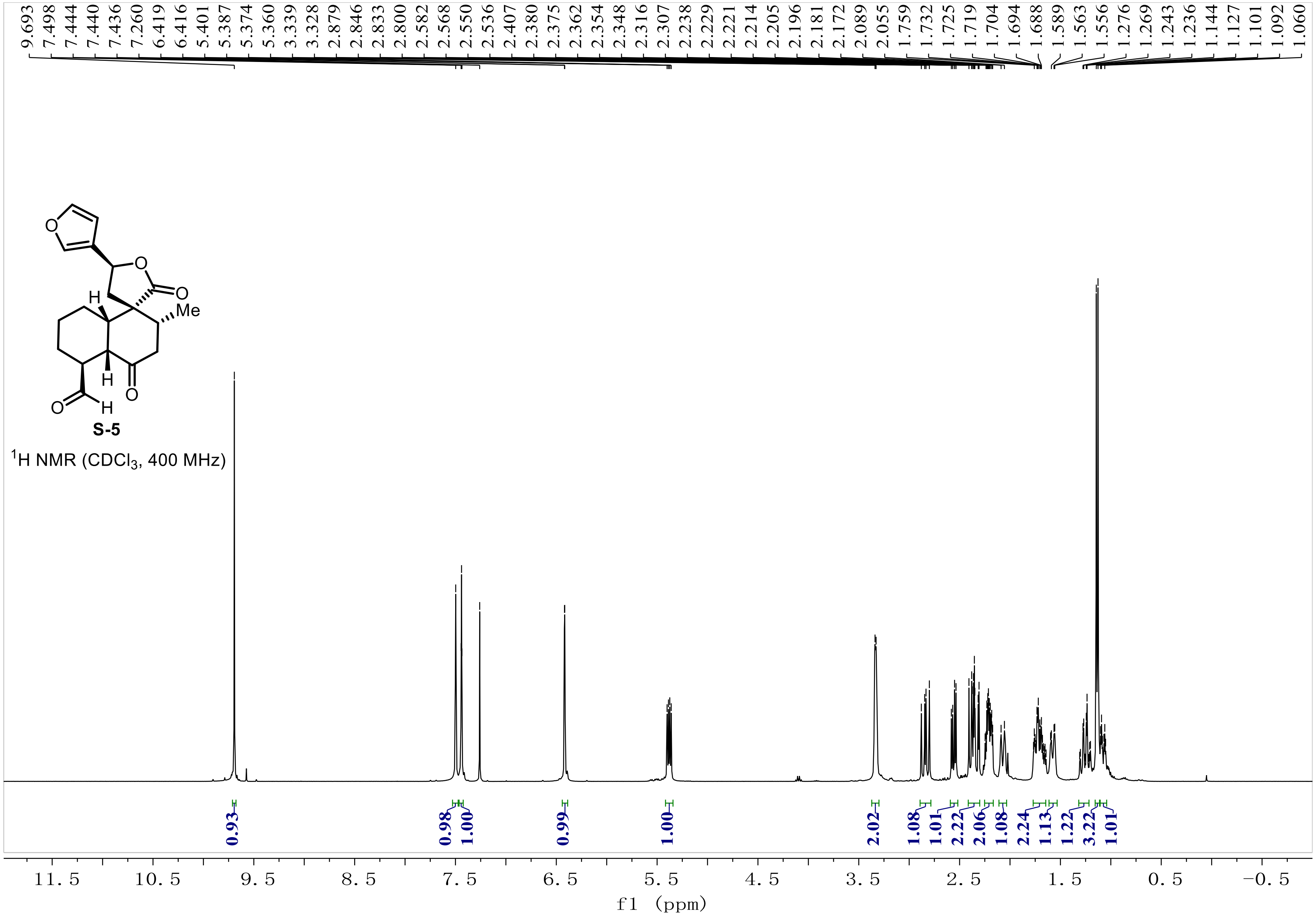
9.693 7.498 7.444 7.440 7.436 7.260 6.419 6.416 5.401 5.387 5.374 5.360 3.339 3.328 2.879 2.846 2.833 2.800 2.582 2.568 2.550 2.536 2.407 2.380 2.375 2.362 2.354 2.348 2.316 2.307 2.238 2.229 2.221 2.214 2.205 2.196 2.181 2.172 2.089 2.055 1.759 1.732 1.725 1.719 1.704 1.694 1.688 1.589 1.563 1.556 1.276 1.269 1.243 1.236 1.144 1.127 1.101 1.092 1.060
O
H
Me
O
H
O
H
S-5
^{1}H NMR ($CDCl_3$, 400 MHz)
0.93 0.98 1.00 0.99 1.00 2.02 1.08 1.01 2.22 2.06 1.08 2.24 1.13 1.22 3.22 1.01
11.5 10.5 9.5 8.5 7.5 6.5 5.5 4.5 3.5 2.5 1.5 0.5 −0.5
f1 (ppm)

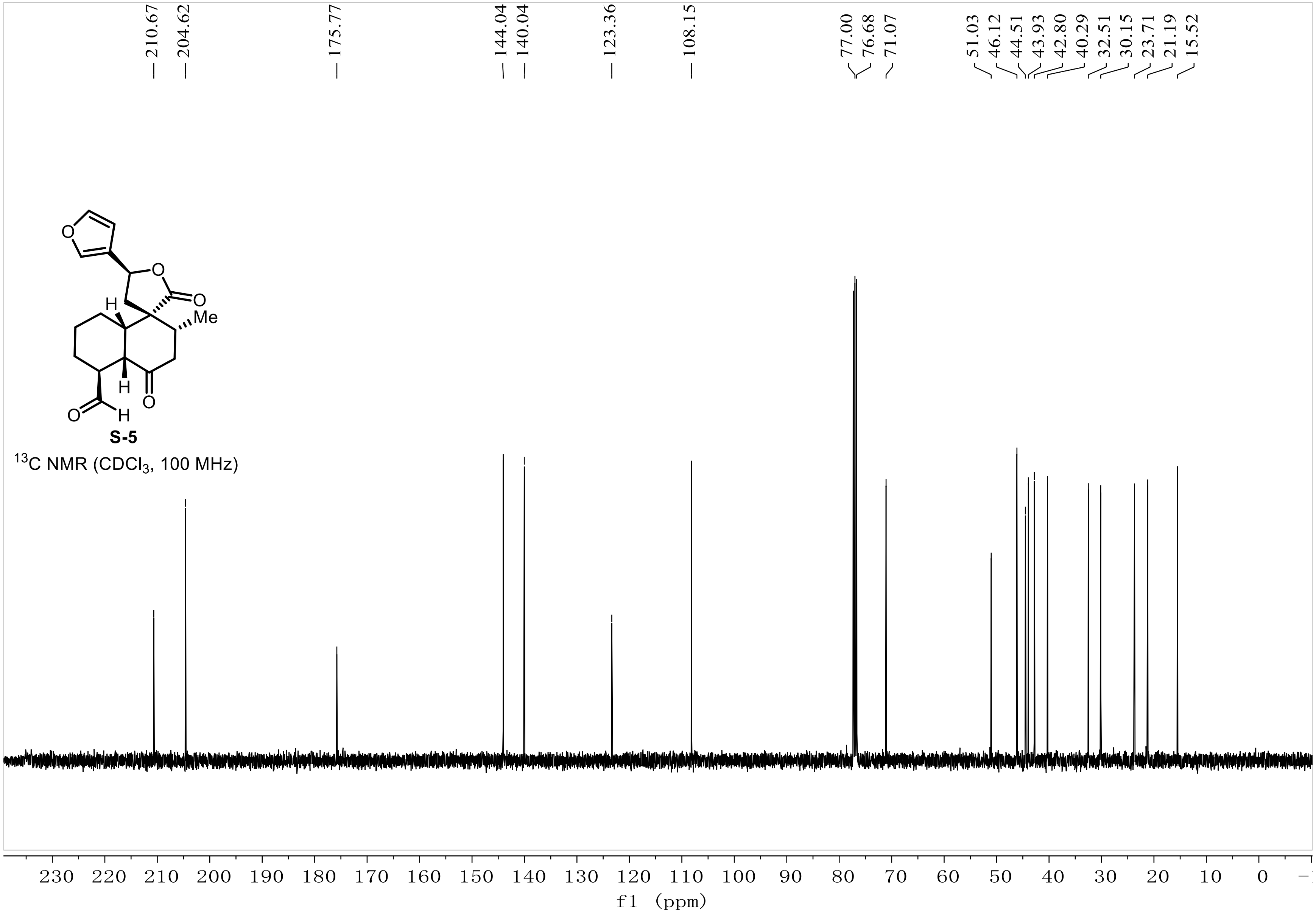
210.67
204.62
175.77
144.04
140.04
123.36
108.15
77.00
76.68
71.07
51.03
46.12
44.51
43.93
42.80
40.29
32.51
30.15
23.71
21.19
15.52
O
O
O
H
Me
H
O
O
H
S-5
13C NMR (CDCl3, 100 MHz)
230 220 210 200 190 180 170 160 150 140 130 120 110 100 90 80 70 60 50 40 30 20 10 0
f1 (ppm)

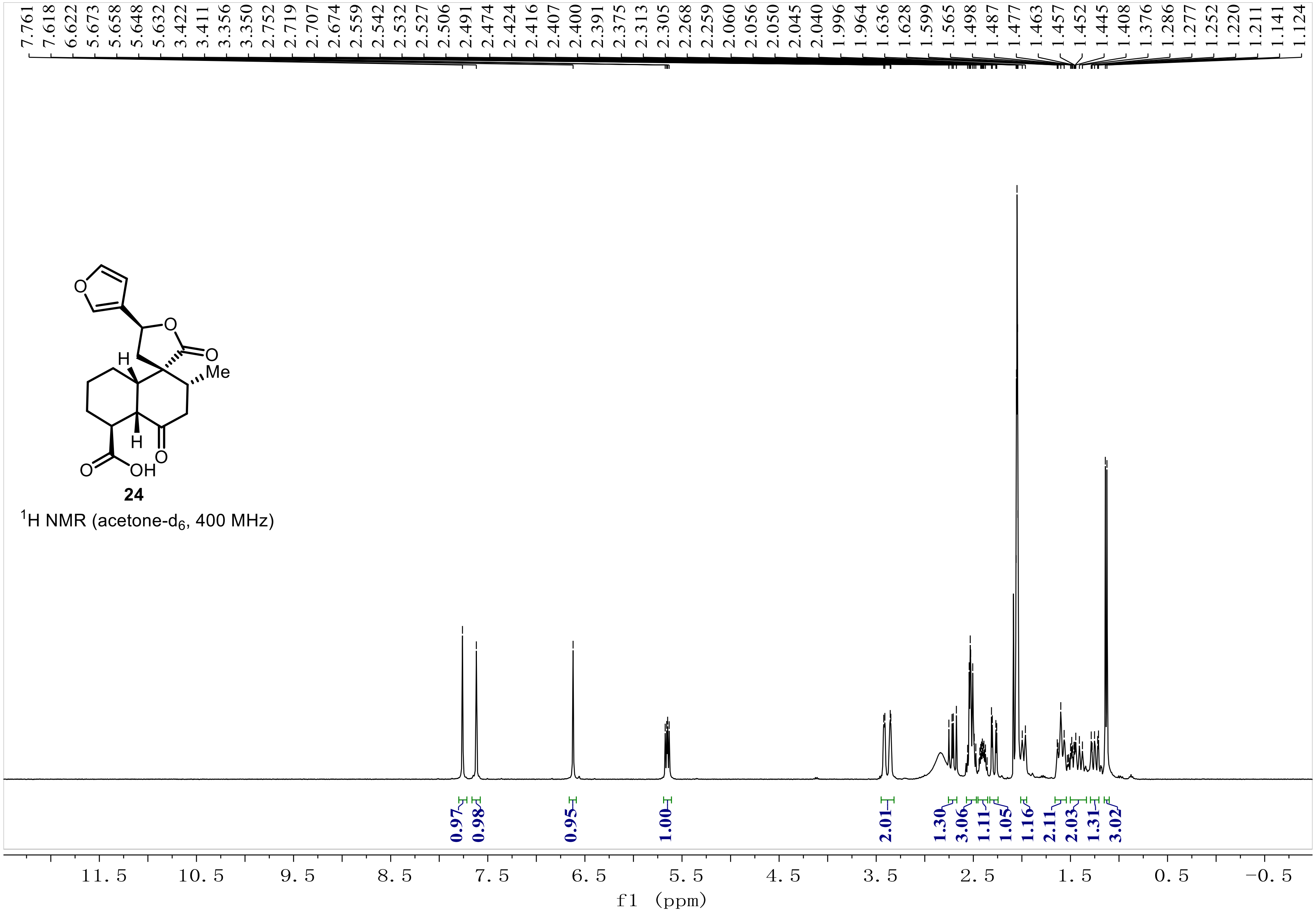
7.761 7.618 6.622 5.673 5.658 5.648 5.632 3.422 3.411 3.356 3.350 2.752 2.719 2.707 2.674 2.559 2.542 2.532 2.527 2.506 2.491 2.474 2.424 2.416 2.407 2.400 2.391 2.375 2.313 2.305 2.268 2.259 2.060 2.056 2.050 2.045 2.040 1.996 1.964 1.636 1.628 1.599 1.565 1.498 1.487 1.477 1.463 1.457 1.452 1.445 1.408 1.376 1.286 1.277 1.252 1.220 1.211 1.141 1.124
O
O
O
H
Me
H
O
O
OH
24
1H NMR (acetone-d6, 400 MHz)
0.97 0.98 0.95 1.00 2.01 1.30 3.06 1.11 1.05 1.16 2.11 2.03 1.31 3.02
11.5 10.5 9.5 8.5 7.5 6.5 5.5 4.5 3.5 2.5 1.5 0.5 −0.5
f1 (ppm)

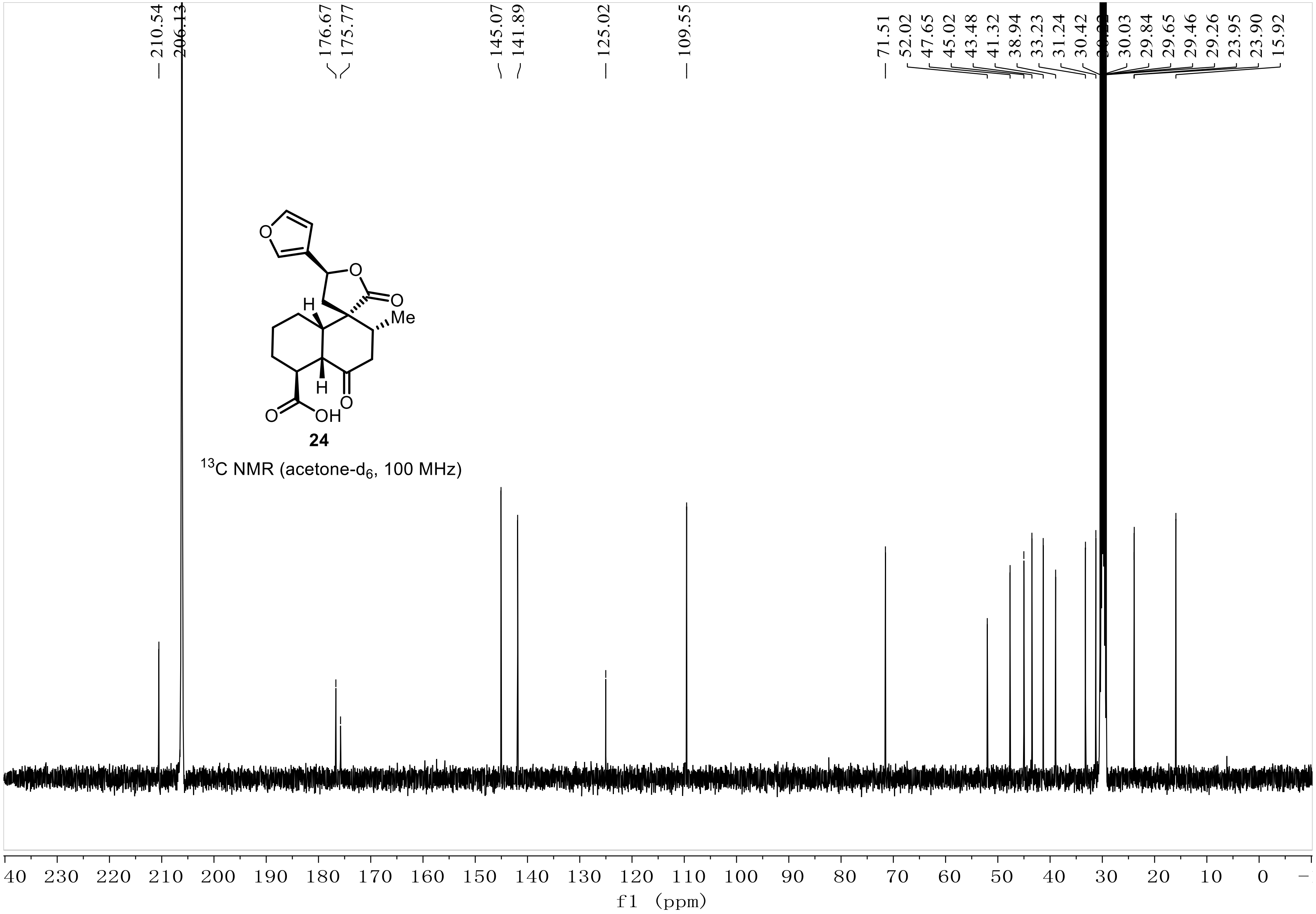
210.54
206.13
176.67
175.77
145.07
141.89
125.02
109.55
71.51
52.02
47.65
45.02
43.48
41.32
38.94
33.23
31.24
30.42
30.03
29.84
29.65
29.46
29.26
23.95
23.90
15.92
O
O
O
H
Me
H
O
O
OH
24
^{13}C NMR (acetone-d$_6$, 100 MHz)
40 230 220 210 200 190 180 170 160 150 140 130 120 110 100 90 80 70 60 50 40 30 20 10 0
f1 (ppm)

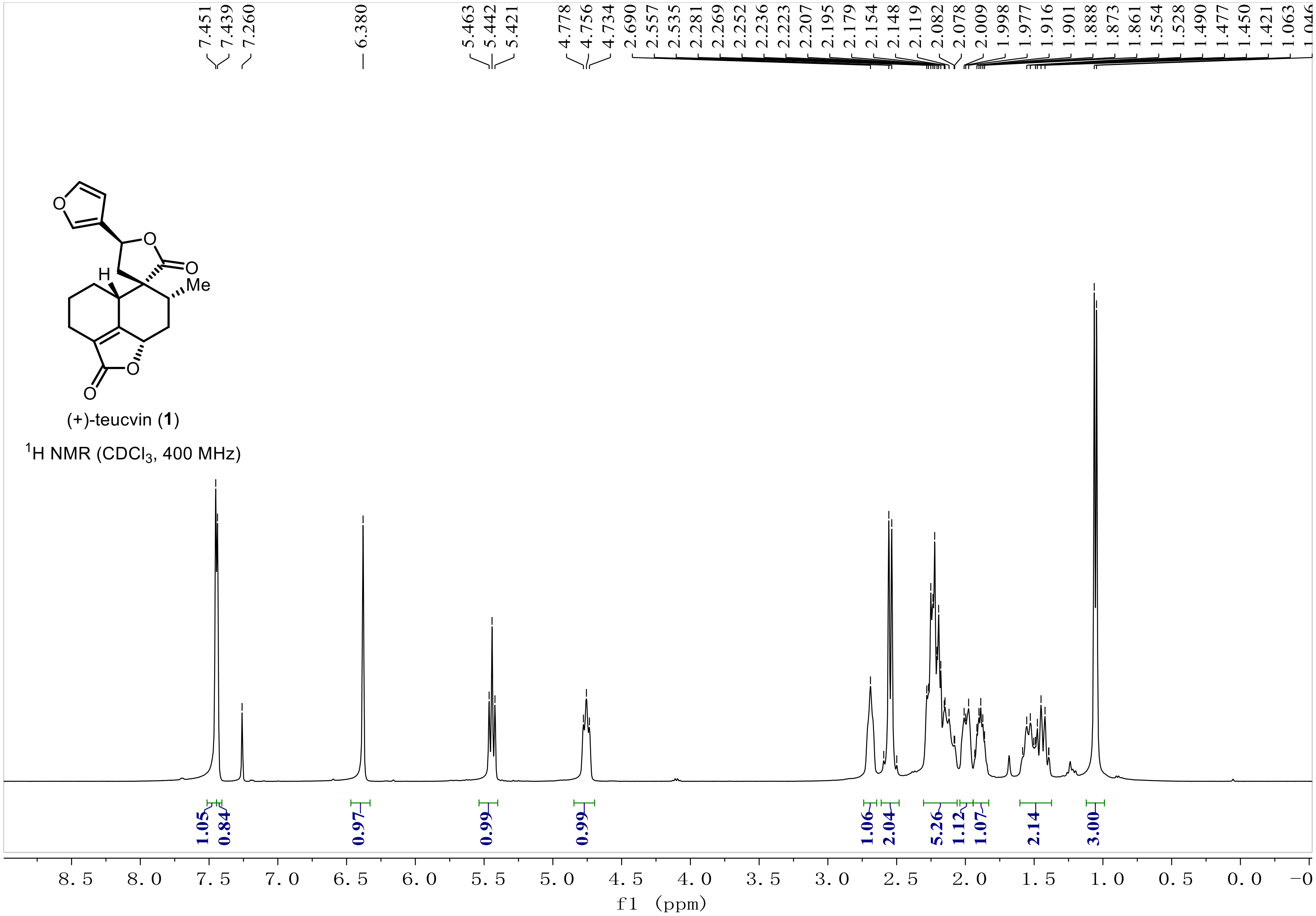
7.451
7.439
7.260
6.380
5.463
5.442
5.421
4.778
4.756
4.734
2.690
2.557
2.535
2.281
2.269
2.252
2.236
2.223
2.207
2.195
2.179
2.154
2.148
2.119
2.082
2.078
2.009
1.998
1.977
1.916
1.901
1.888
1.873
1.861
1.554
1.528
1.490
1.477
1.450
1.421
1.063
1.046
O
O
O
H
Me
O
O
(+)-teucvin (1)
1H NMR (CDCl3, 400 MHz)
1.05
0.84
0.97
0.99
0.99
1.06
2.04
5.26
1.12
1.07
2.14
3.00
8.5
8.0
7.5
7.0
6.5
6.0
5.5
5.0
4.5
4.0
3.5
3.0
2.5
2.0
1.5
1.0
0.5
0.0
f1 (ppm)

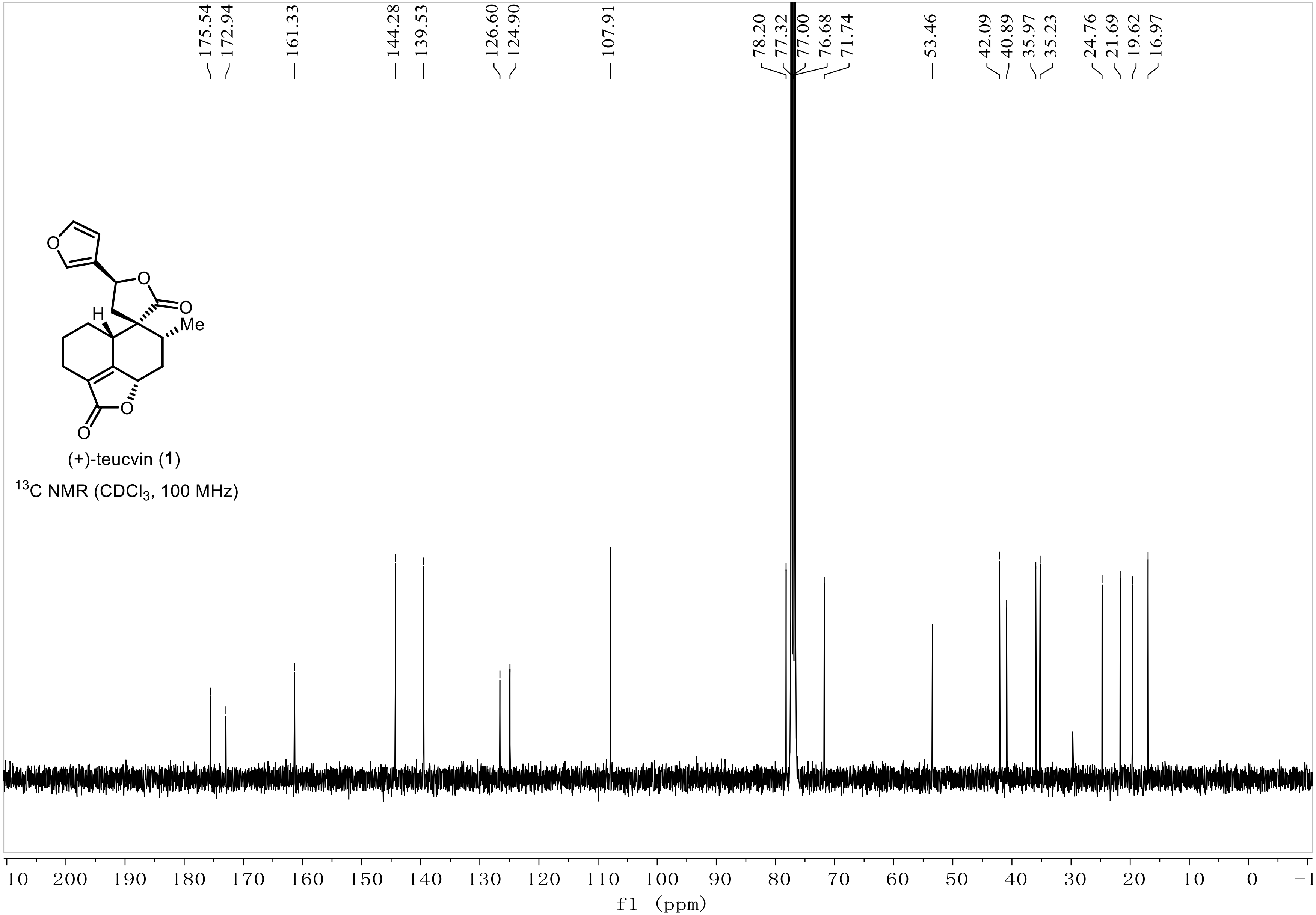
175.54
172.94
161.33
144.28
139.53
126.60
124.90
107.91
78.20
77.32
77.00
76.68
71.74
53.46
42.09
40.89
35.97
35.23
24.76
21.69
19.62
16.97
(+)-teucvin (**1**)
^{13}C NMR ($CDCl_3$, 100 MHz)
f1 (ppm)

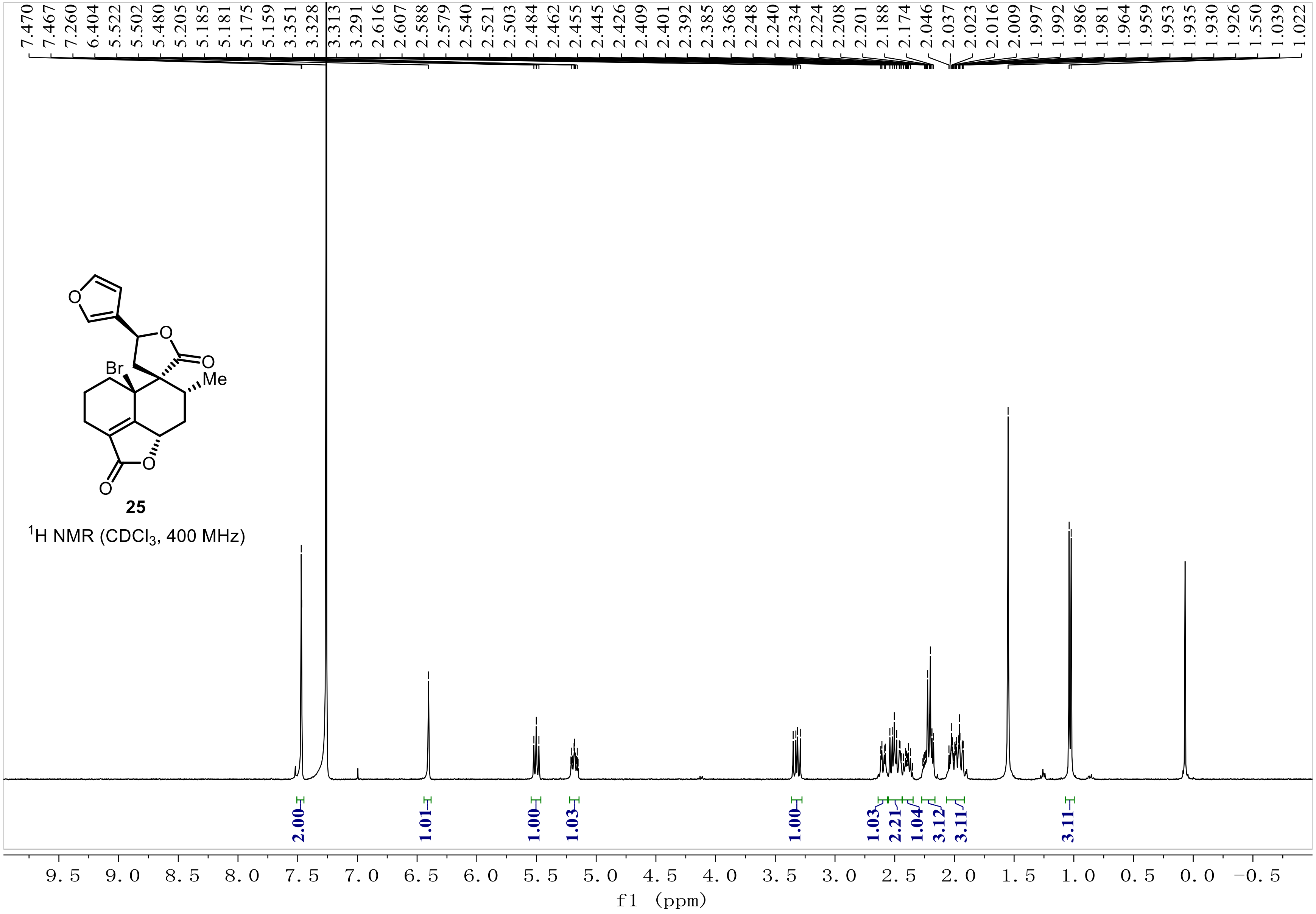
7.470 7.467 7.260 6.404 5.522 5.502 5.480 5.205 5.185 5.181 5.175 5.159 3.351 3.328 3.313 3.291 2.616 2.607 2.588 2.579 2.540 2.521 2.503 2.484 2.462 2.455 2.445 2.426 2.409 2.401 2.392 2.385 2.368 2.248 2.240 2.234 2.224 2.208 2.201 2.188 2.174 2.046 2.037 2.023 2.016 2.009 1.997 1.992 1.986 1.981 1.964 1.959 1.953 1.935 1.930 1.926 1.550 1.039 1.022
O
O
O
Br
Me
O
O
O
25
^{1}H NMR ($CDCl_3$, 400 MHz)
2.00
1.01
1.00
1.03
1.00
1.03
2.21
1.04
3.12
3.11
3.11
9.5 9.0 8.5 8.0 7.5 7.0 6.5 6.0 5.5 5.0 4.5 4.0 3.5 3.0 2.5 2.0 1.5 1.0 0.5 0.0 -0.5
f1 (ppm)

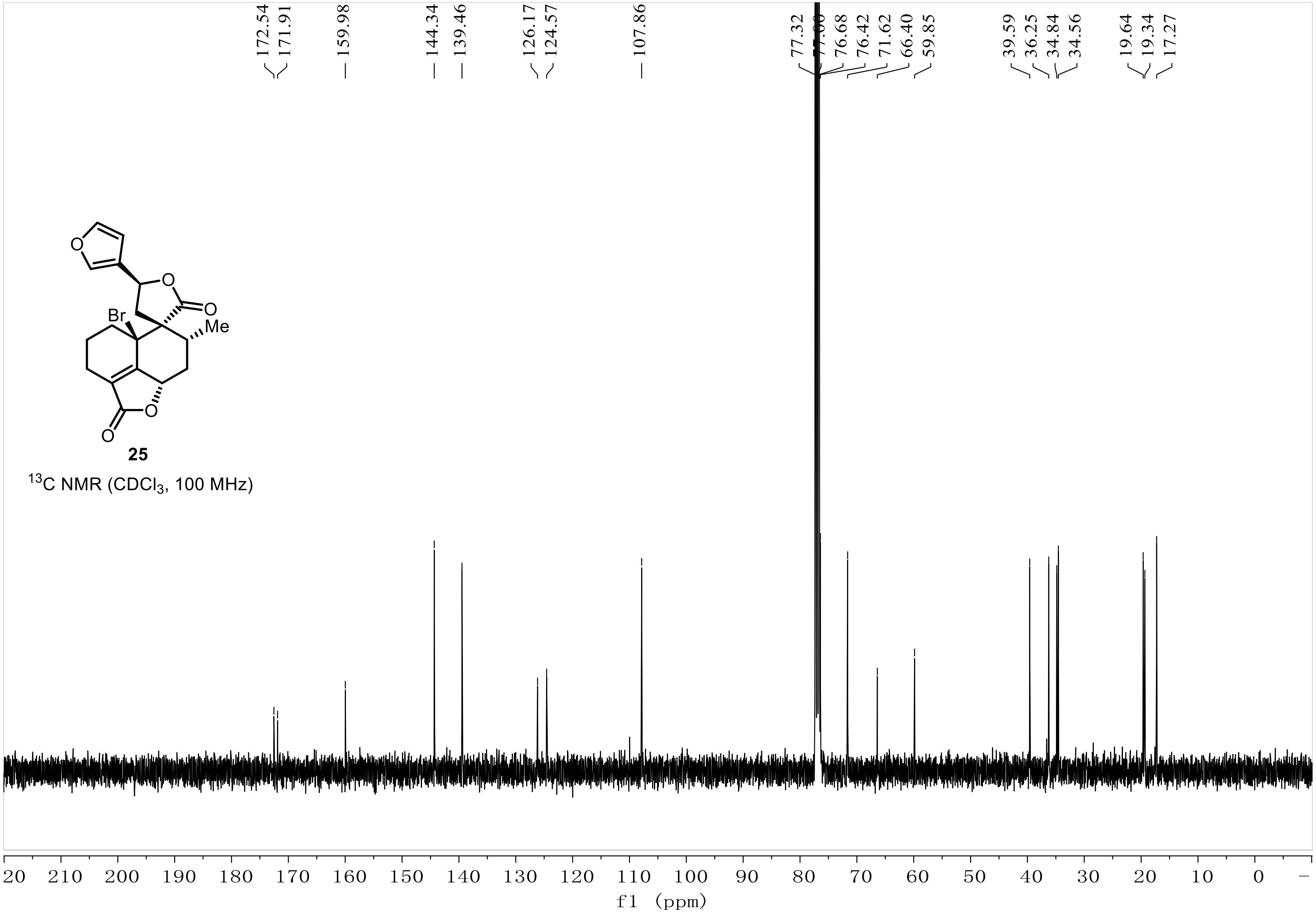
172.54
171.91
159.98
144.34
139.46
126.17
124.57
107.86
77.32
77.00
76.68
76.42
71.62
66.40
59.85
39.59
36.25
34.84
34.56
19.64
19.34
17.27
O
O
O
Br
Me
O
O
O
25
^{13}C NMR (CDCl$_3$, 100 MHz)
20 210 200 190 180 170 160 150 140 130 120 110 100 90 80 70 60 50 40 30 20 10 0
f1 (ppm)

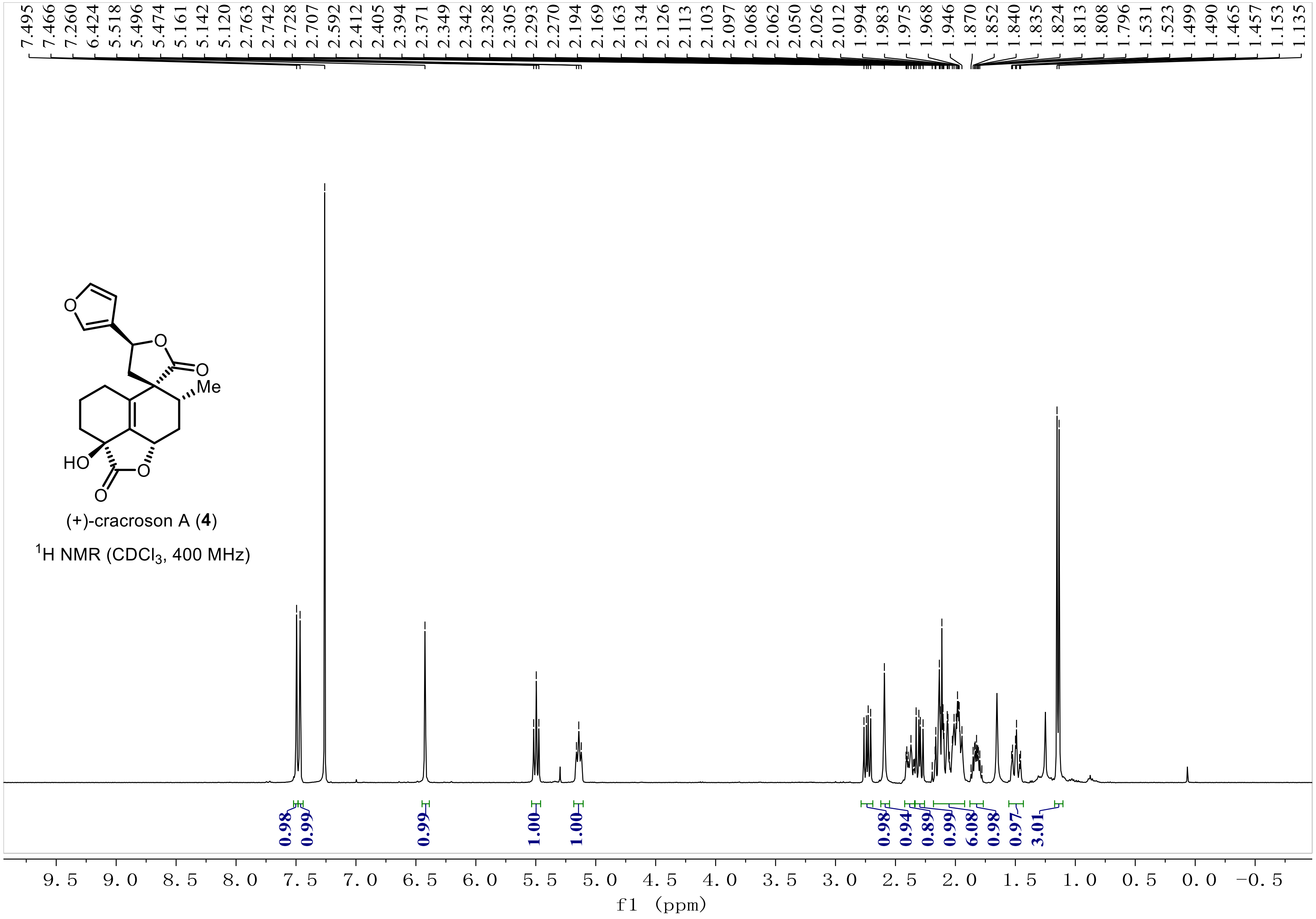
7.495 7.466 7.260 6.424 5.518 5.496 5.474 5.161 5.142 5.120 2.763 2.742 2.728 2.707 2.592 2.412 2.405 2.394 2.371 2.349 2.342 2.328 2.305 2.293 2.270 2.194 2.169 2.163 2.134 2.126 2.113 2.103 2.097 2.068 2.062 2.050 2.026 2.012 1.994 1.983 1.975 1.968 1.946 1.870 1.852 1.840 1.835 1.824 1.813 1.808 1.796 1.531 1.523 1.499 1.490 1.465 1.457 1.153 1.135
O
O
O
Me
HO
O
O
O
(+)-cracroson A (**4**)
^{1}H NMR ($CDCl_3$, 400 MHz)
0.98 0.99 0.99 1.00 1.00 0.98 0.94 0.89 0.99 6.08 0.98 0.97 3.01
9.5 9.0 8.5 8.0 7.5 7.0 6.5 6.0 5.5 5.0 4.5 4.0 3.5 3.0 2.5 2.0 1.5 1.0 0.5 0.0 -0.5
f1 (ppm)

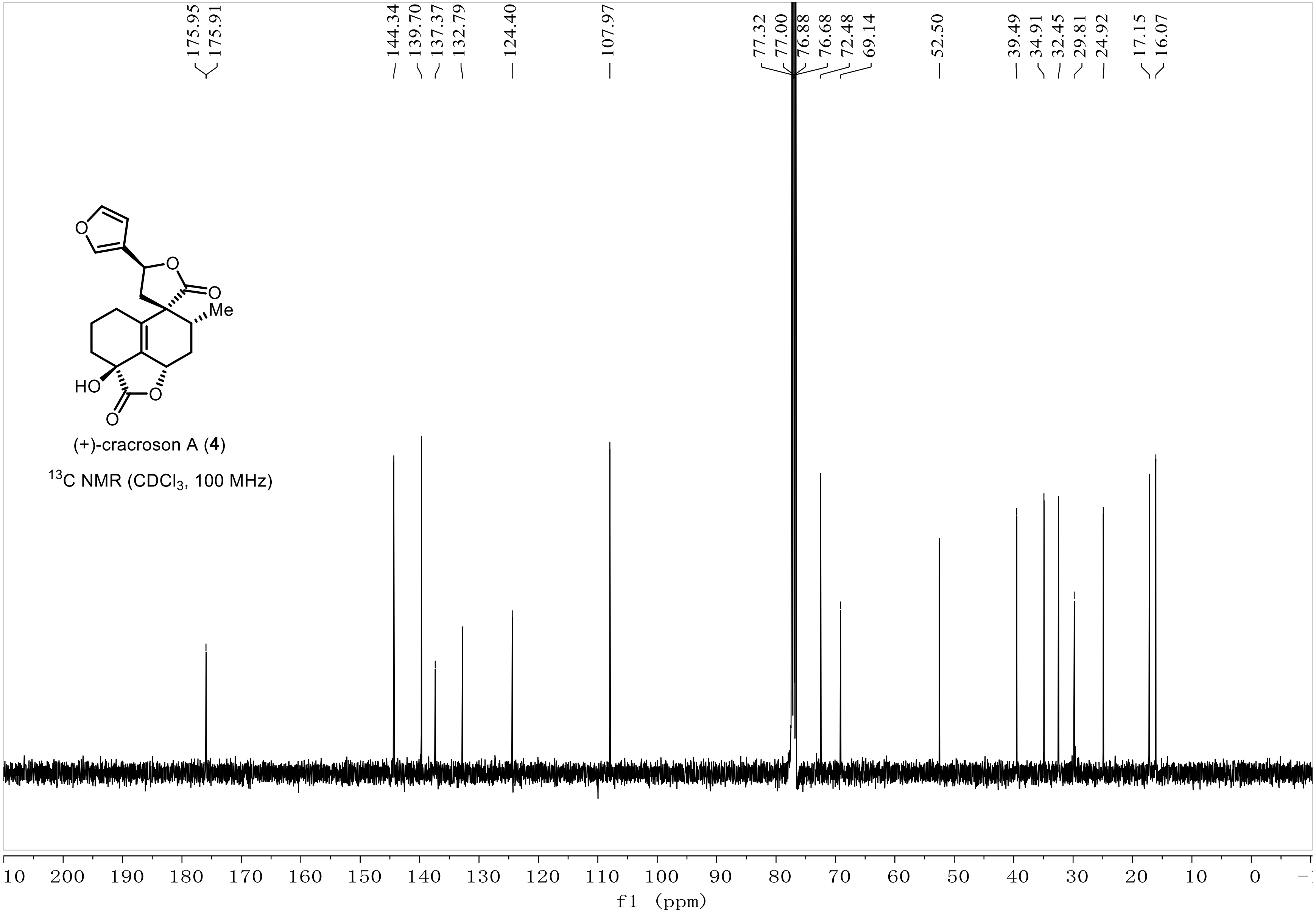
175.95
175.91
144.34
139.70
137.37
132.79
124.40
107.97
77.32
77.00
76.88
76.68
72.48
69.14
52.50
39.49
34.91
32.45
29.81
24.92
17.15
16.07
Me
HO
O
(+)-cracroson A (4)
^{13}C NMR (CDCl$_3$, 100 MHz)
f1 (ppm)

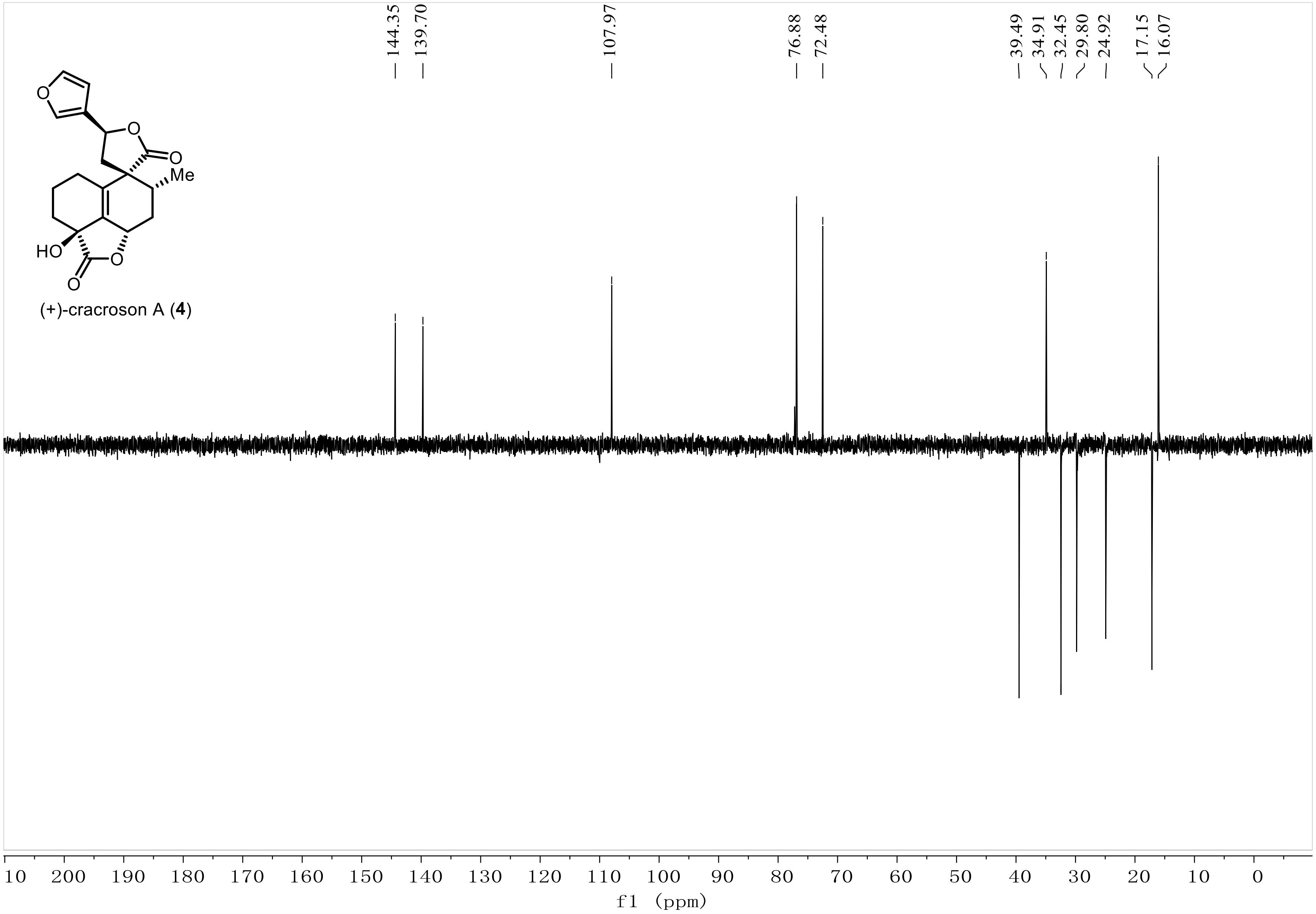

144.35
139.70
107.97
76.88
72.48
39.49
34.91
32.45
29.80
24.92
17.15
16.07
O
O
O
Me
HO
O
O
O
(+)-cracroson A (4)
10 200 190 180 170 160 150 140 130 120 110 100 90 80 70 60 50 40 30 20 10 0
f1 (ppm)

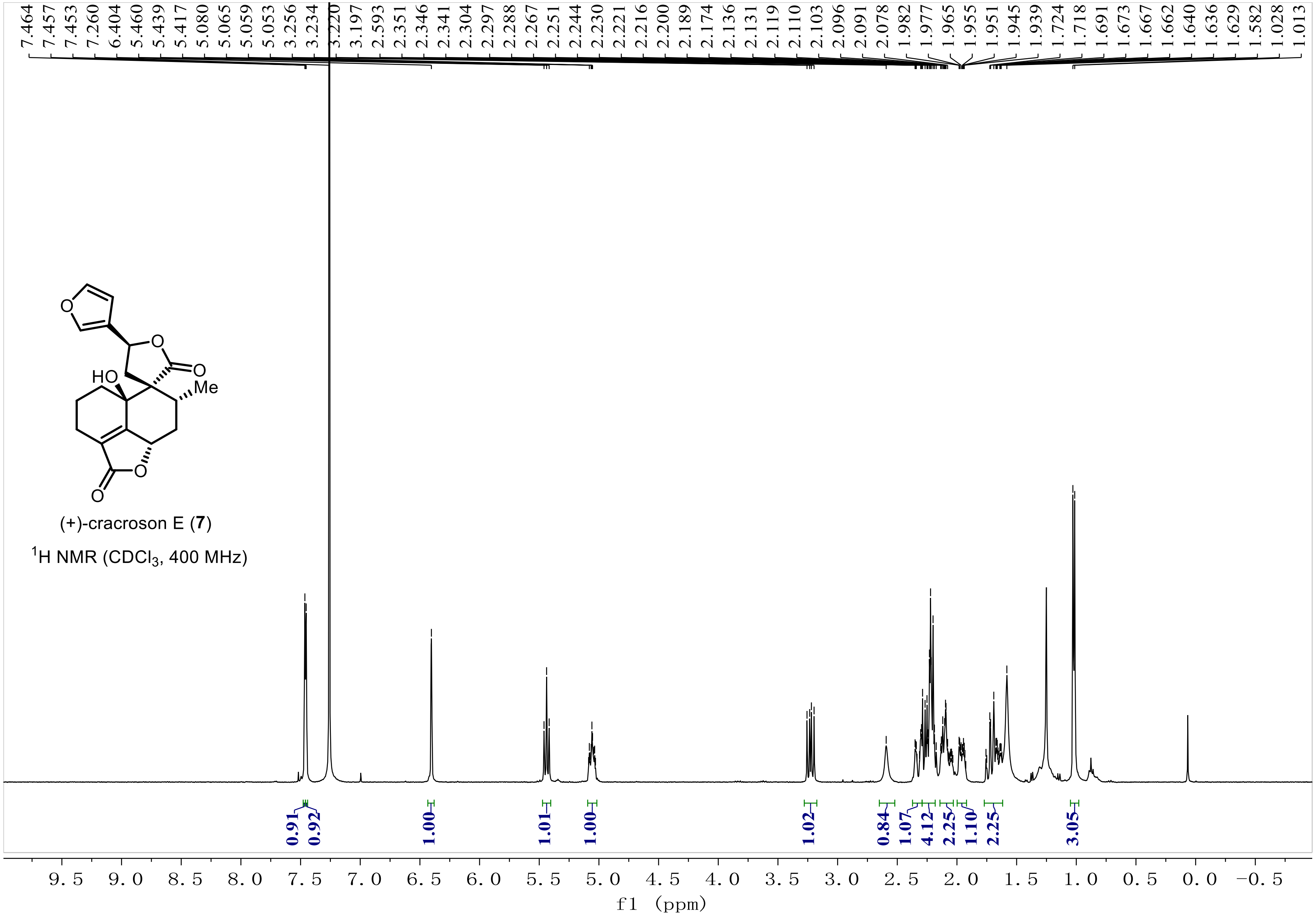

(+)-cracroson E (**7**)

^{1}H NMR ($CDCl_3$, 400 MHz)

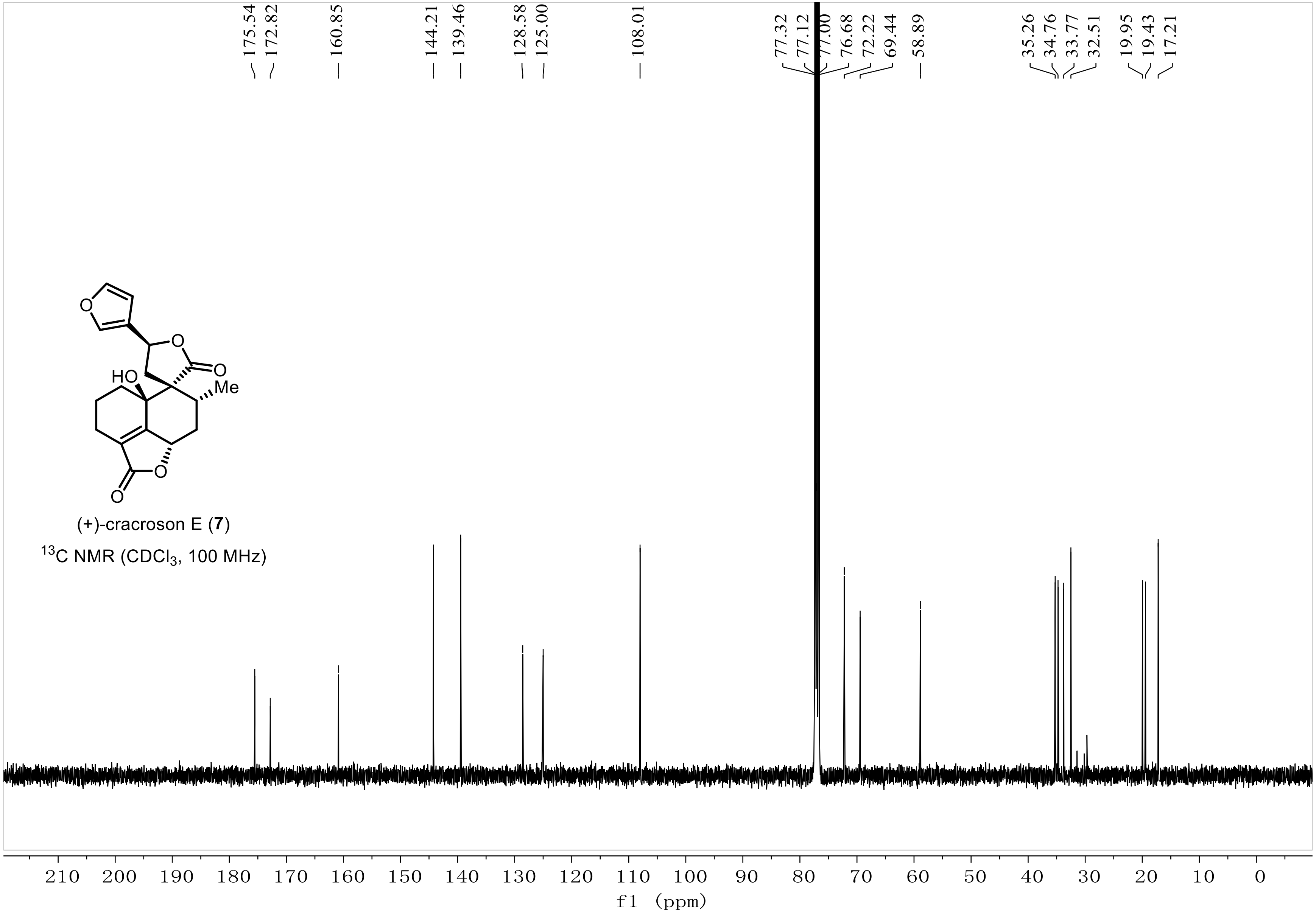
175.54
172.82
160.85
144.21
139.46
128.58
125.00
108.01
77.32
77.12
77.00
76.68
72.22
69.44
58.89
35.26
34.76
33.77
32.51
19.95
19.43
17.21
HO
Me
O
(+)-cracroson E (7)
13C NMR (CDCl3, 100 MHz)
210 200 190 180 170 160 150 140 130 120 110 100 90 80 70 60 50 40 30 20 10 0
f1 (ppm)

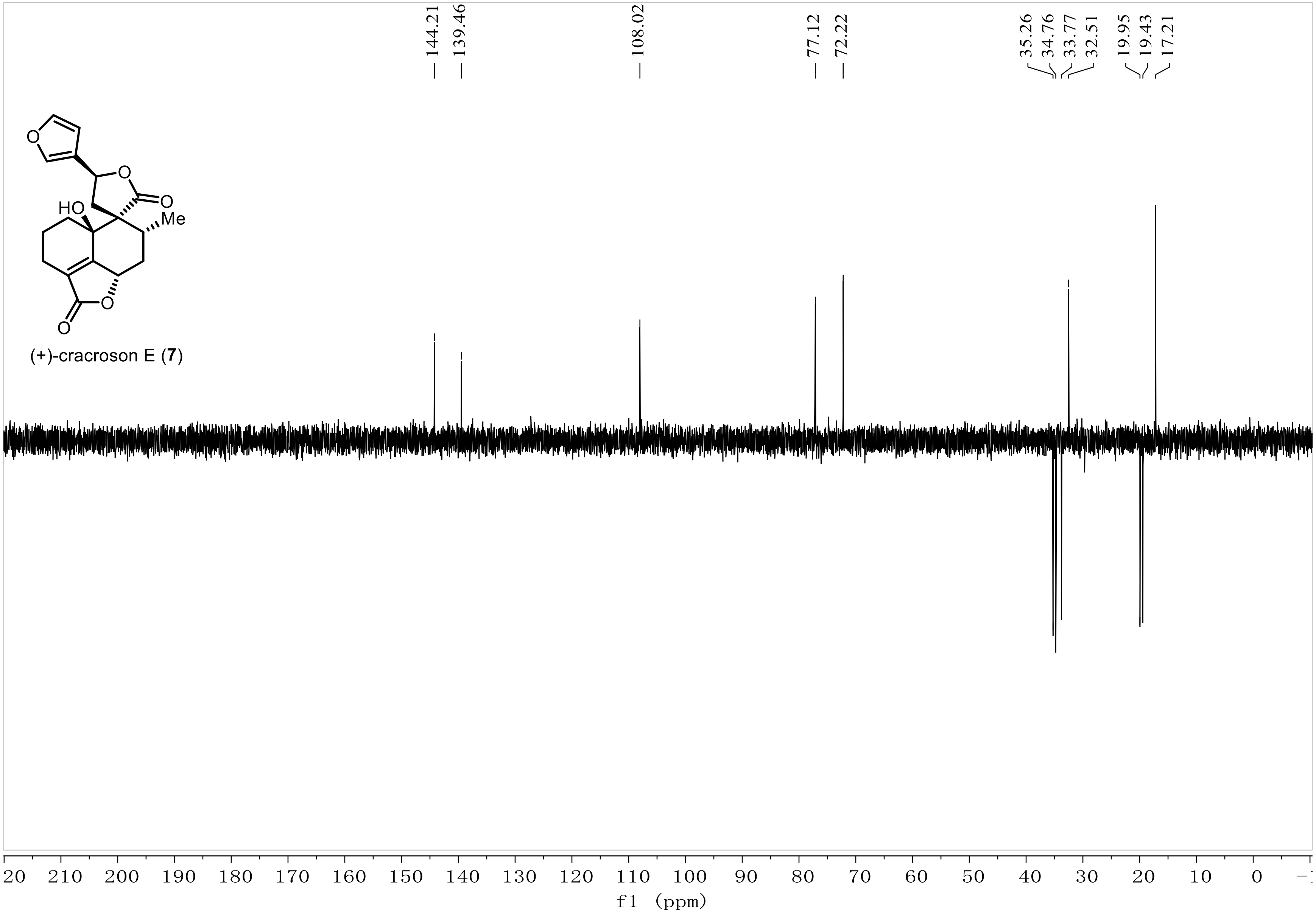
144.21
139.46
108.02
77.12
72.22
35.26
34.76
33.77
32.51
19.95
19.43
17.21
HO
O
O
O
Me
O
O
(+)-cracroson E (7)
20 210 200 190 180 170 160 150 140 130 120 110 100 90 80 70 60 50 40 30 20 10 0
f1 (ppm)

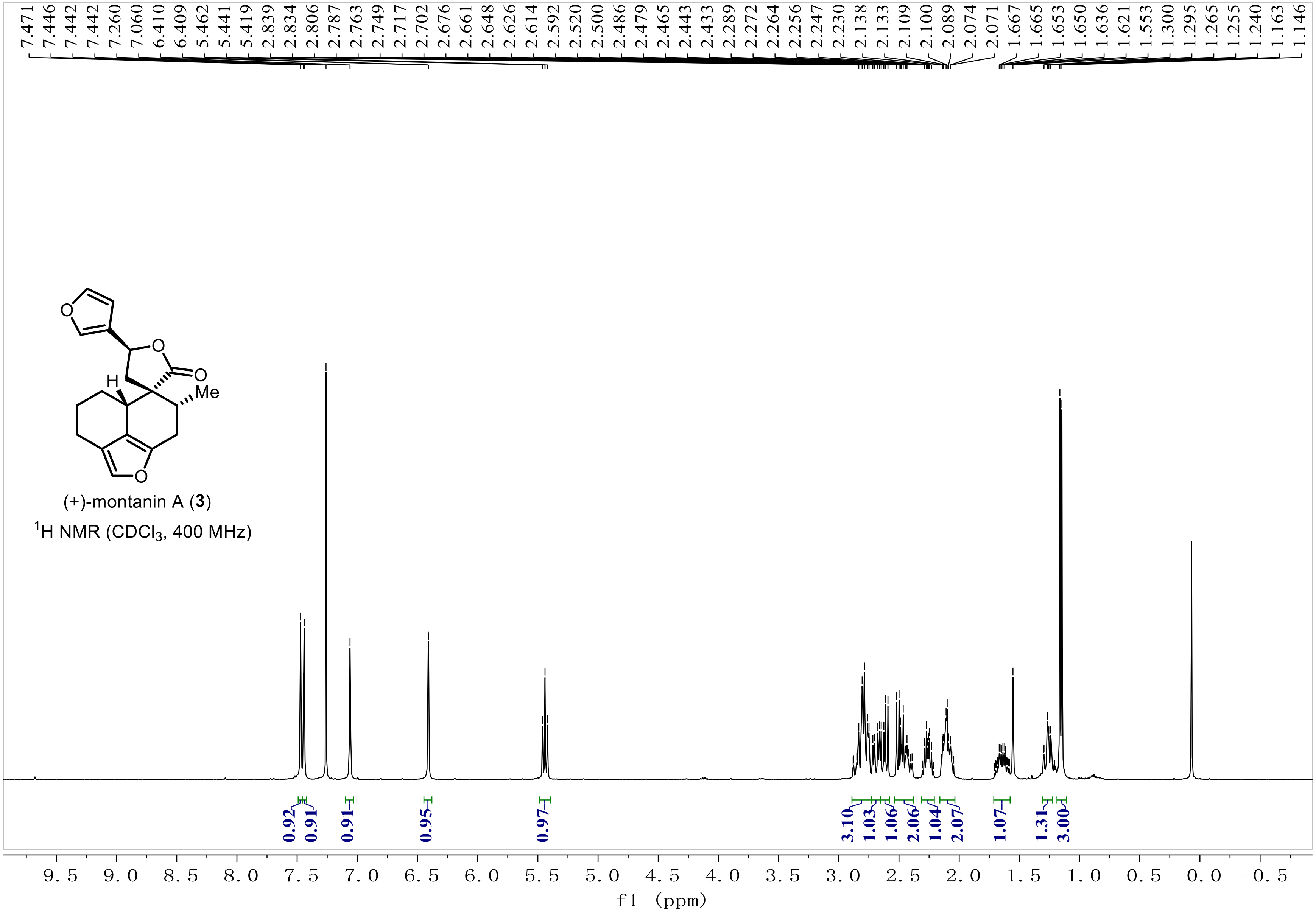
(+)-montanin A (3)
1H NMR (CDCl3, 400 MHz)
f1 (ppm)

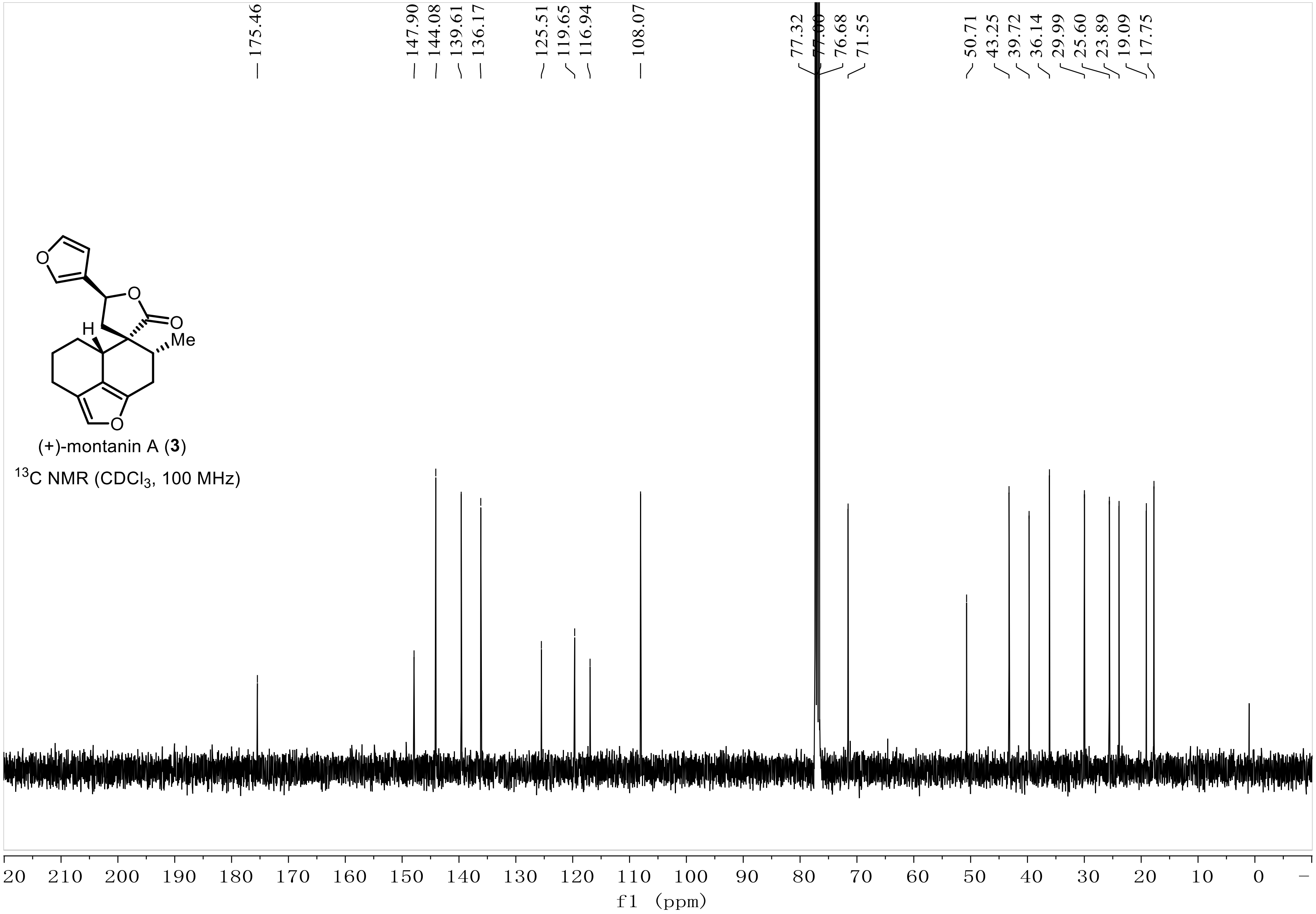
175.46
147.90
144.08
139.61
136.17
125.51
119.65
116.94
108.07
77.32
76.68
71.55
50.71
43.25
39.72
36.14
29.99
25.60
23.89
19.09
17.75
O
O
O
H
Me
O
(+)-montanin A (3)
13C NMR (CDCl3, 100 MHz)
20 210 200 190 180 170 160 150 140 130 120 110 100 90 80 70 60 50 40 30 20 10 0
f1 (ppm)

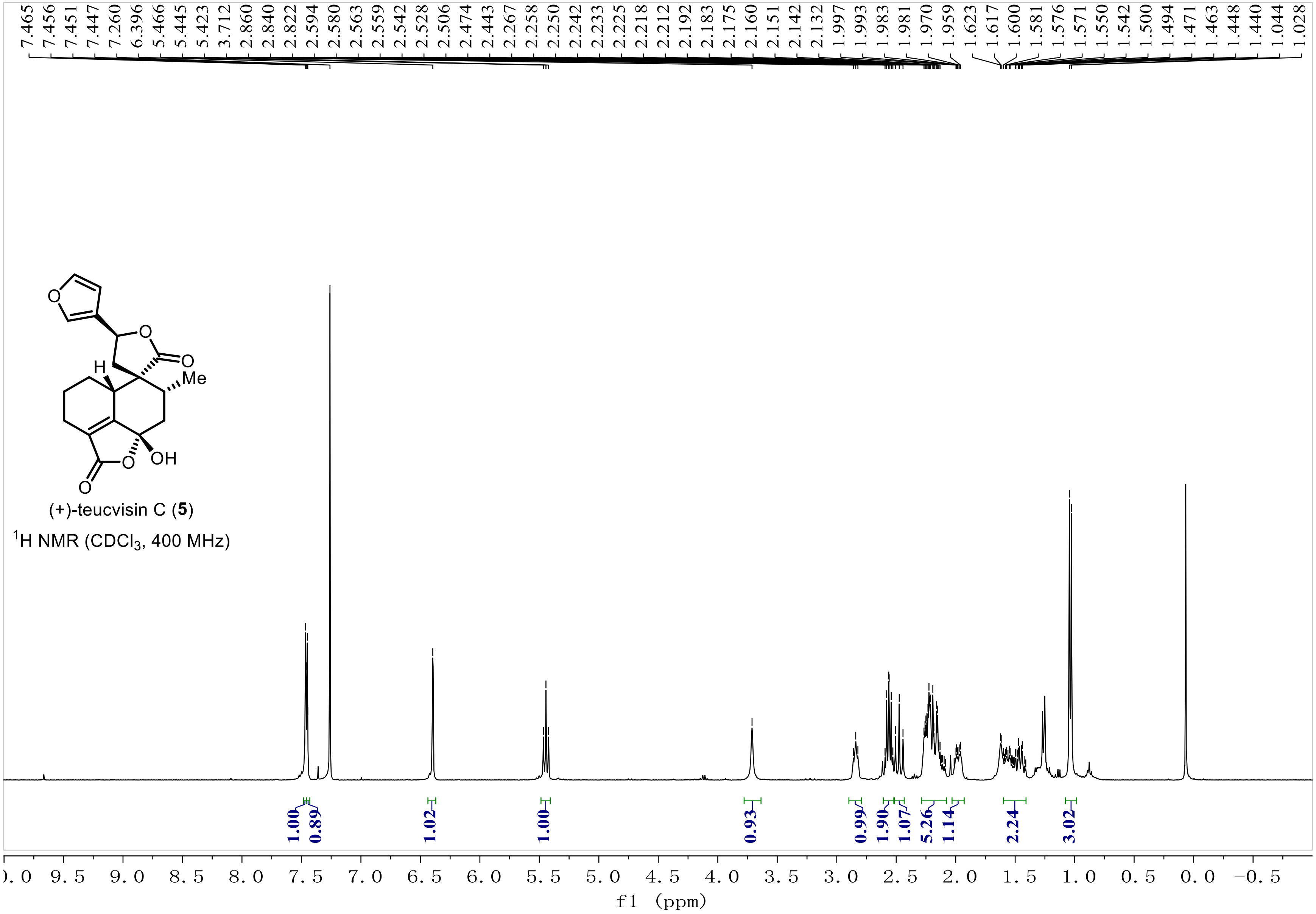
(+)-teucvisin C (5)
^{1}H NMR ($CDCl_3$, 400 MHz)
O
O
O
H
Me
O
OH
O
7.465 7.456 7.451 7.447 7.260 6.396 5.466 5.445 5.423 3.712 2.860 2.840 2.822 2.594 2.580 2.563 2.559 2.542 2.528 2.506 2.474 2.443 2.267 2.258 2.250 2.242 2.233 2.225 2.218 2.212 2.192 2.183 2.175 2.160 2.151 2.142 2.132 1.997 1.993 1.983 1.981 1.970 1.959 1.623 1.617 1.600 1.581 1.576 1.571 1.550 1.542 1.500 1.494 1.471 1.463 1.448 1.440 1.044 1.028
1.00 0.89 1.02 1.00 0.93 0.99 1.90 1.07 5.26 1.14 2.24 3.02
f1 (ppm)

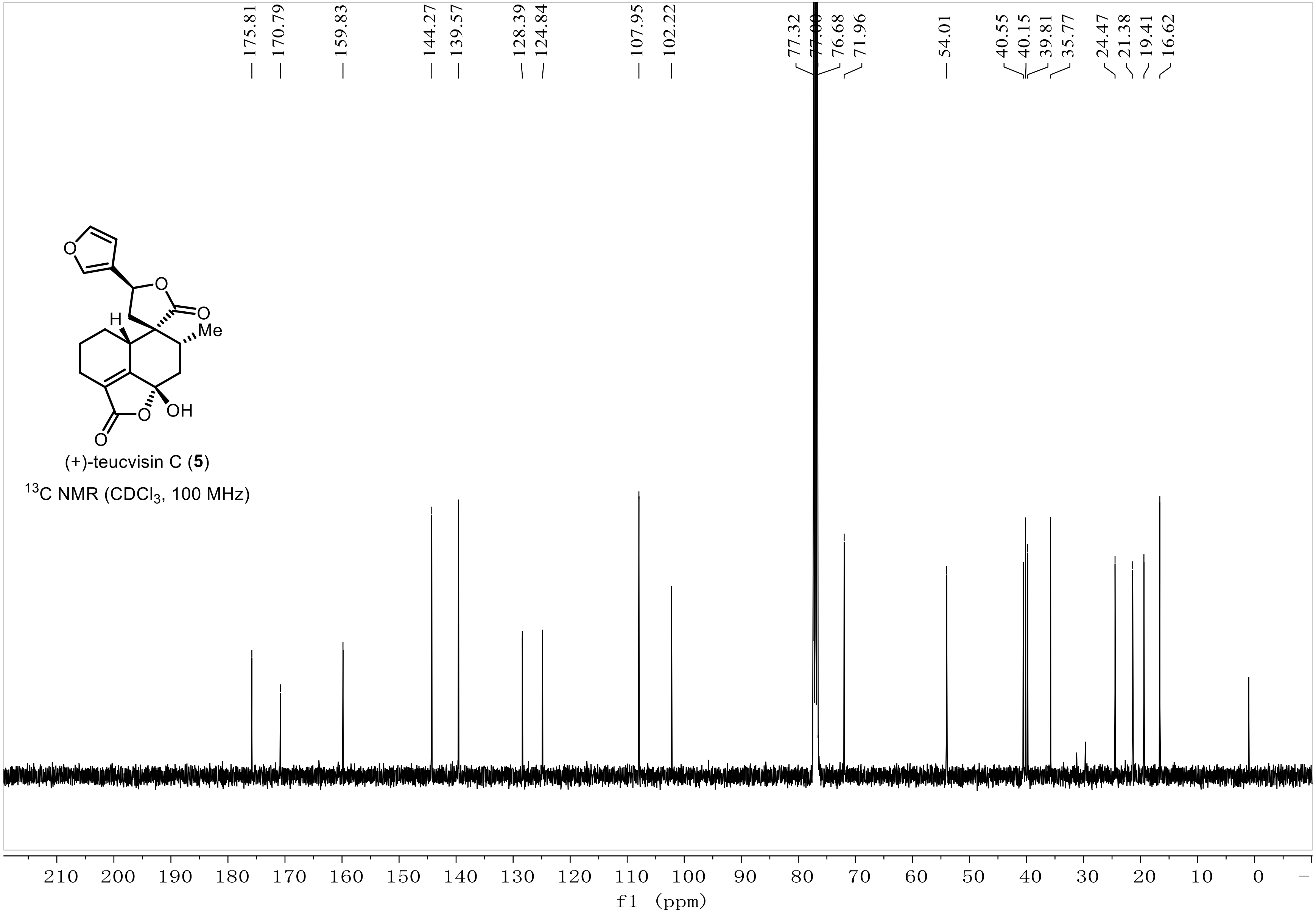
175.81
170.79
159.83
144.27
139.57
128.39
124.84
107.95
102.22
77.32
77.00
76.68
71.96
54.01
40.55
40.15
39.81
35.77
24.47
21.38
19.41
16.62
Me
H
OH
(+)-teucvisin C (**5**)
^{13}C NMR ($CDCl_3$, 100 MHz)
210 200 190 180 170 160 150 140 130 120 110 100 90 80 70 60 50 40 30 20 10 0
f1 (ppm)

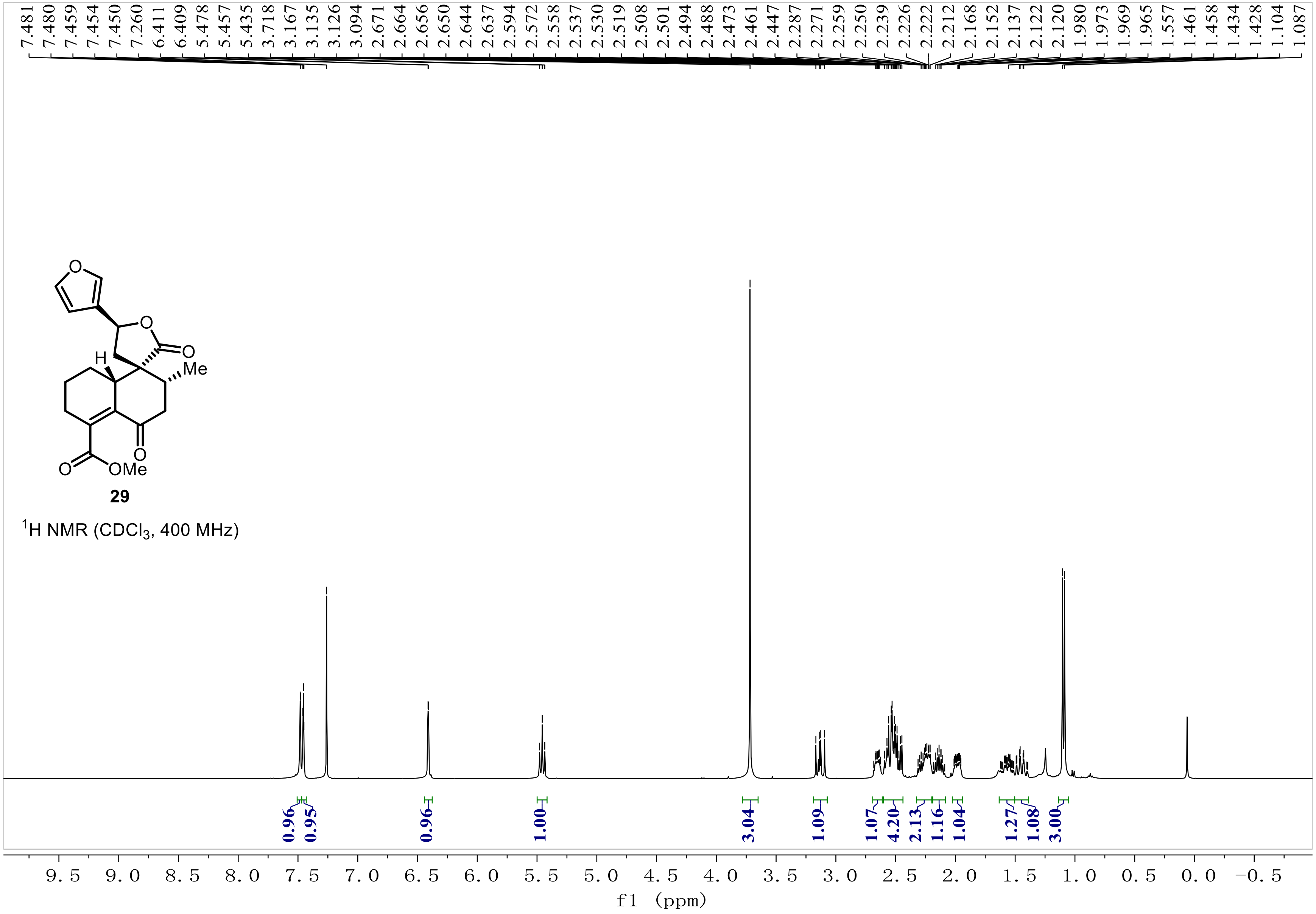
7.481 7.480 7.459 7.454 7.450 7.260 6.411 6.409 5.478 5.457 5.435 3.718 3.167 3.135 3.126 3.094 2.671 2.664 2.656 2.650 2.644 2.637 2.594 2.572 2.558 2.537 2.530 2.519 2.508 2.501 2.494 2.488 2.473 2.461 2.447 2.287 2.271 2.259 2.250 2.239 2.226 2.222 2.212 2.168 2.152 2.137 2.122 2.120 1.980 1.973 1.969 1.965 1.557 1.461 1.458 1.434 1.428 1.104 1.087
H
Me
O
OMe
29
^{1}H NMR ($CDCl_3$, 400 MHz)
0.96 0.95 0.96 1.00 3.04 1.09 1.07 4.20 2.13 1.16 1.04 1.27 1.08 3.00
9.5 9.0 8.5 8.0 7.5 7.0 6.5 6.0 5.5 5.0 4.5 4.0 3.5 3.0 2.5 2.0 1.5 1.0 0.5 0.0 -0.5
f1 (ppm)

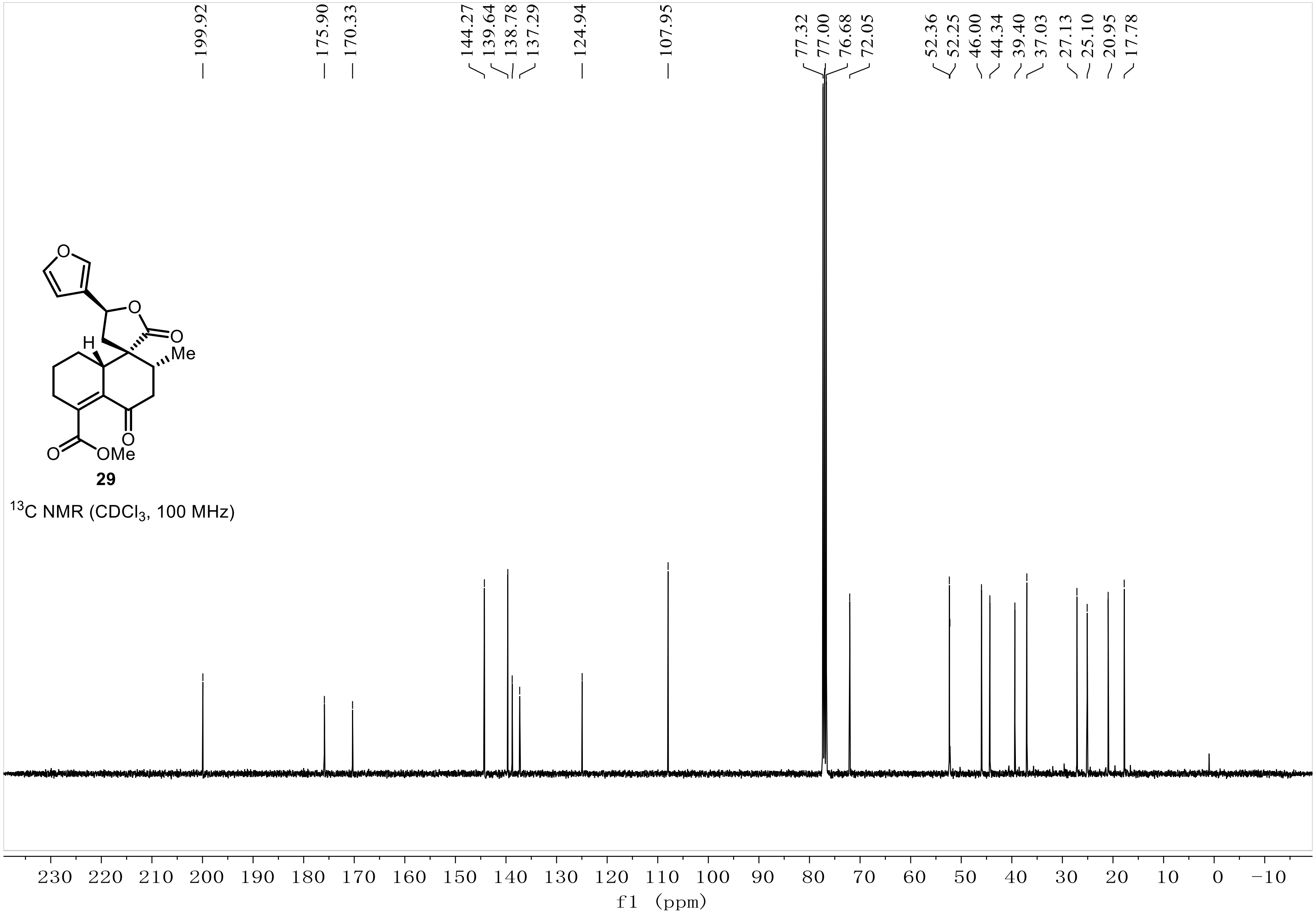
199.92
175.90
170.33
144.27
139.64
138.78
137.29
124.94
107.95
77.32
77.00
76.68
72.05
52.36
52.25
46.00
44.34
39.40
37.03
27.13
25.10
20.95
17.78
O
H
Me
OMe
29
^{13}C NMR ($CDCl_3$, 100 MHz)
230 220 210 200 190 180 170 160 150 140 130 120 110 100 90 80 70 60 50 40 30 20 10 0 −10
f1 (ppm)

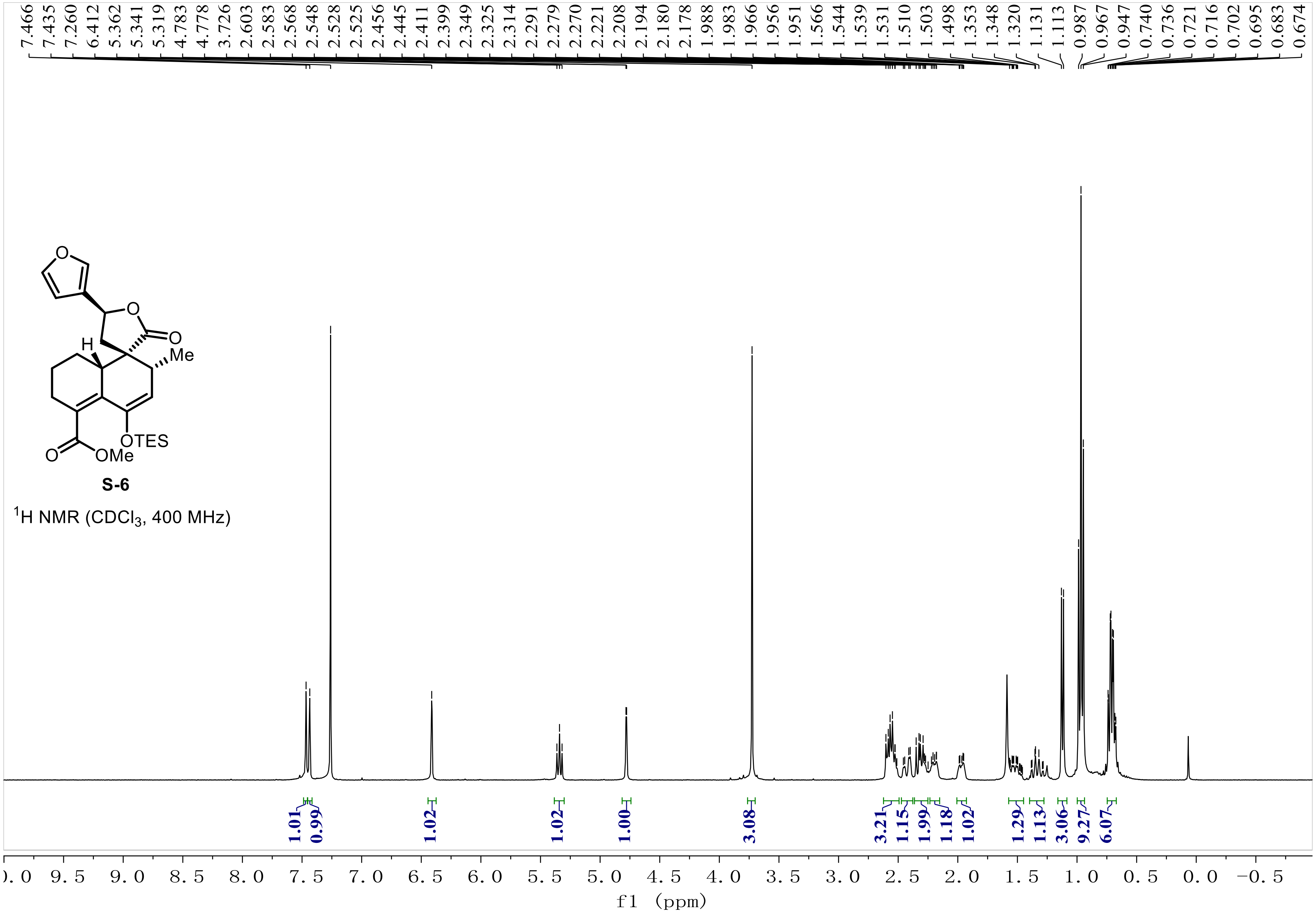
S-6
^{1}H NMR (CDCl$_3$, 400 MHz)
f1 (ppm)

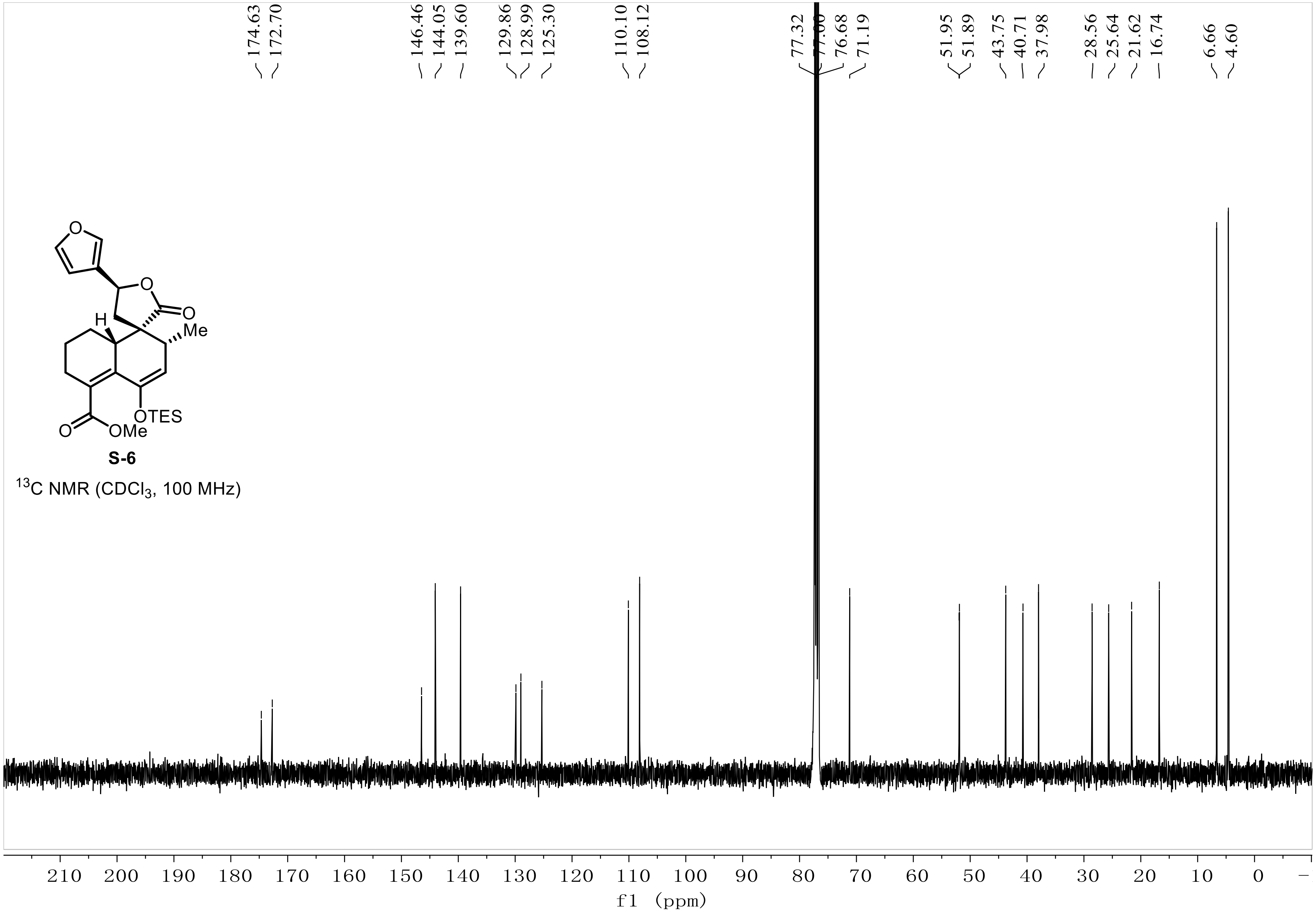
174.63
172.70
146.46
144.05
139.60
129.86
128.99
125.30
110.10
108.12
77.32
76.68
71.19
51.95
51.89
43.75
40.71
37.98
28.56
25.64
21.62
16.74
6.66
4.60
O
O
O
H
Me
OTES
O
OMe
S-6
^{13}C NMR ($CDCl_3$, 100 MHz)
210 200 190 180 170 160 150 140 130 120 110 100 90 80 70 60 50 40 30 20 10 0
f1 (ppm)

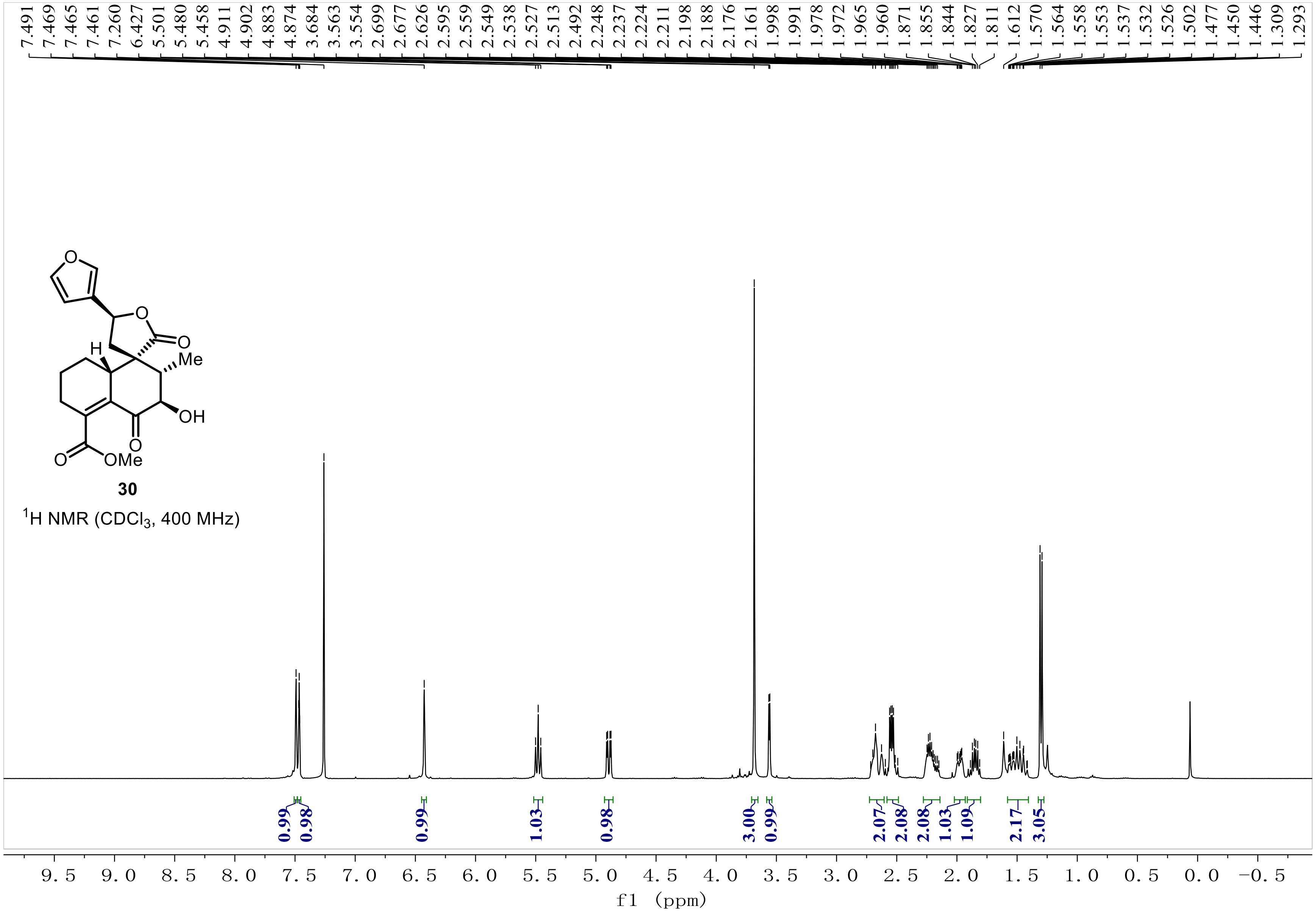
7.491 7.469 7.465 7.461 7.260 6.427 5.501 5.480 5.458 4.911 4.902 4.883 4.874 3.684 3.563 3.554 2.699 2.677 2.626 2.595 2.559 2.549 2.538 2.527 2.513 2.492 2.248 2.237 2.224 2.211 2.198 2.188 2.176 2.161 1.998 1.991 1.978 1.972 1.965 1.960 1.871 1.855 1.844 1.827 1.811 1.612 1.570 1.564 1.558 1.553 1.537 1.532 1.526 1.502 1.477 1.450 1.446 1.309 1.293
O
O
O
H
Me
OH
O
O
OMe
30
^{1}H NMR ($CDCl_3$, 400 MHz)
0.99 0.98 0.99 1.03 0.98 3.00 0.99 2.07 2.08 2.08 1.03 1.09 2.17 3.05
9.5 9.0 8.5 8.0 7.5 7.0 6.5 6.0 5.5 5.0 4.5 4.0 3.5 3.0 2.5 2.0 1.5 1.0 0.5 0.0 -0.5
f1 (ppm)

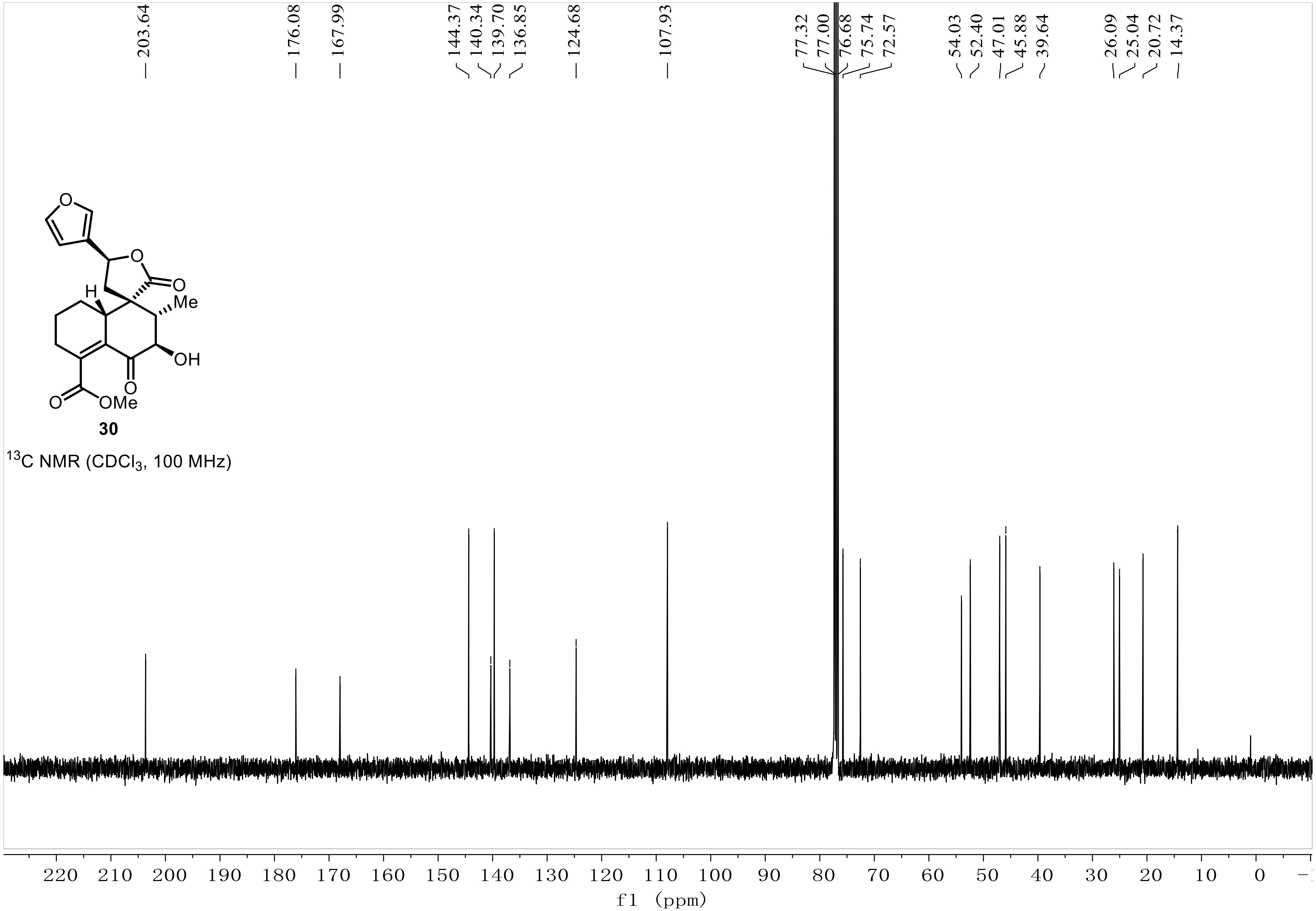
203.64
176.08
167.99
144.37
140.34
139.70
136.85
124.68
107.93
77.32
77.00
76.68
75.74
72.57
54.03
52.40
47.01
45.88
39.64
26.09
25.04
20.72
14.37
O
O
O
H
Me
OH
O
O
OMe
30
^{13}C NMR ($CDCl_3$, 100 MHz)
220 210 200 190 180 170 160 150 140 130 120 110 100 90 80 70 60 50 40 30 20 10 0
f1 (ppm)

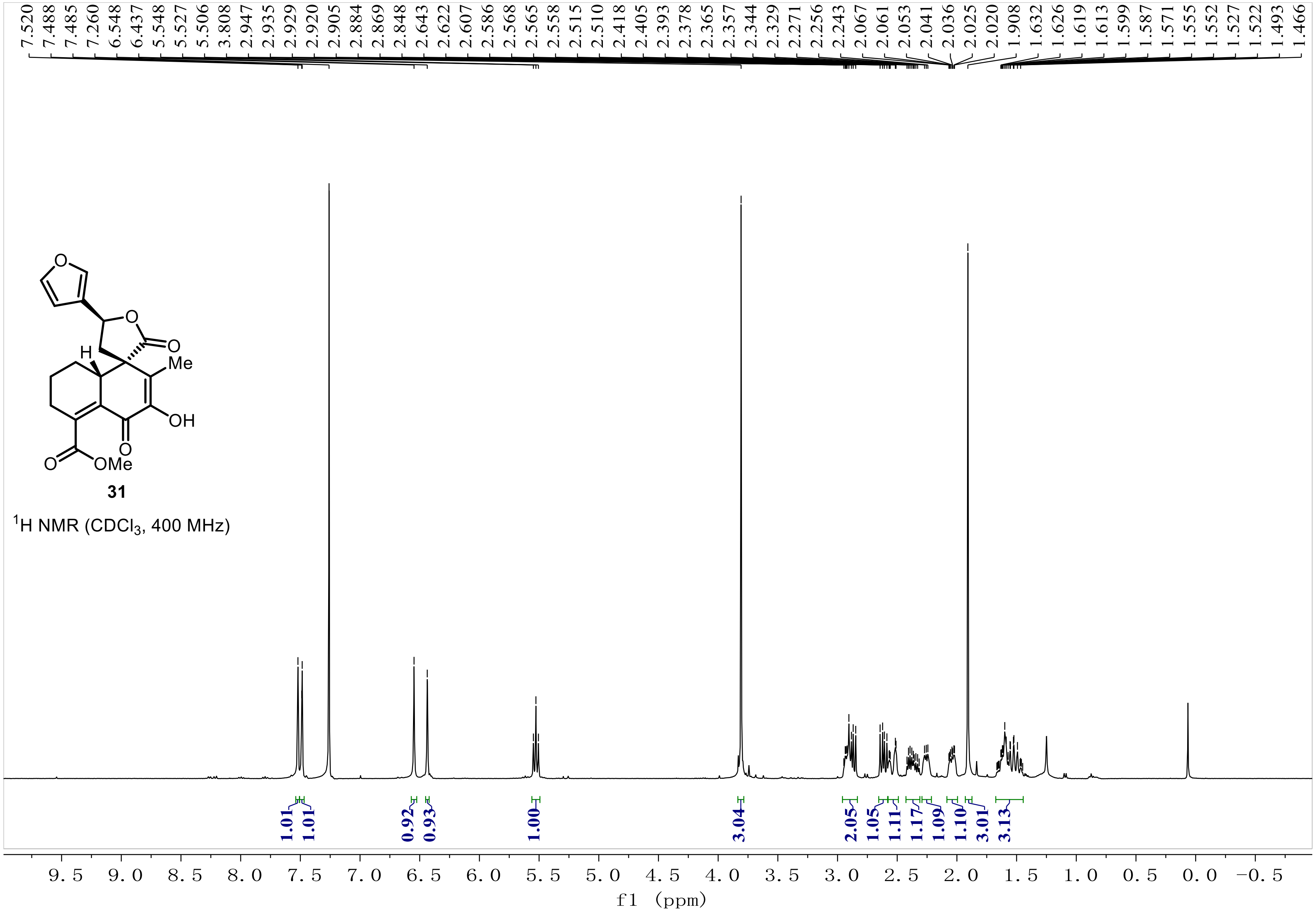
O
O
O
H
Me
OH
O
O
OMe
31
1H NMR (CDCl3, 400 MHz)
7.520
7.488
7.485
7.260
6.548
6.437
5.548
5.527
5.506
3.808
2.947
2.935
2.929
2.920
2.905
2.884
2.869
2.848
2.643
2.622
2.607
2.586
2.568
2.565
2.558
2.515
2.510
2.418
2.405
2.393
2.378
2.365
2.357
2.344
2.329
2.271
2.256
2.243
2.067
2.061
2.053
2.041
2.036
2.025
2.020
1.908
1.632
1.626
1.619
1.613
1.599
1.587
1.571
1.555
1.552
1.527
1.522
1.493
1.466
1.01
1.01
0.92
0.93
1.00
3.04
2.05
1.05
1.11
1.17
1.09
1.10
3.01
3.13
9.5 9.0 8.5 8.0 7.5 7.0 6.5 6.0 5.5 5.0 4.5 4.0 3.5 3.0 2.5 2.0 1.5 1.0 0.5 0.0 -0.5
f1 (ppm)

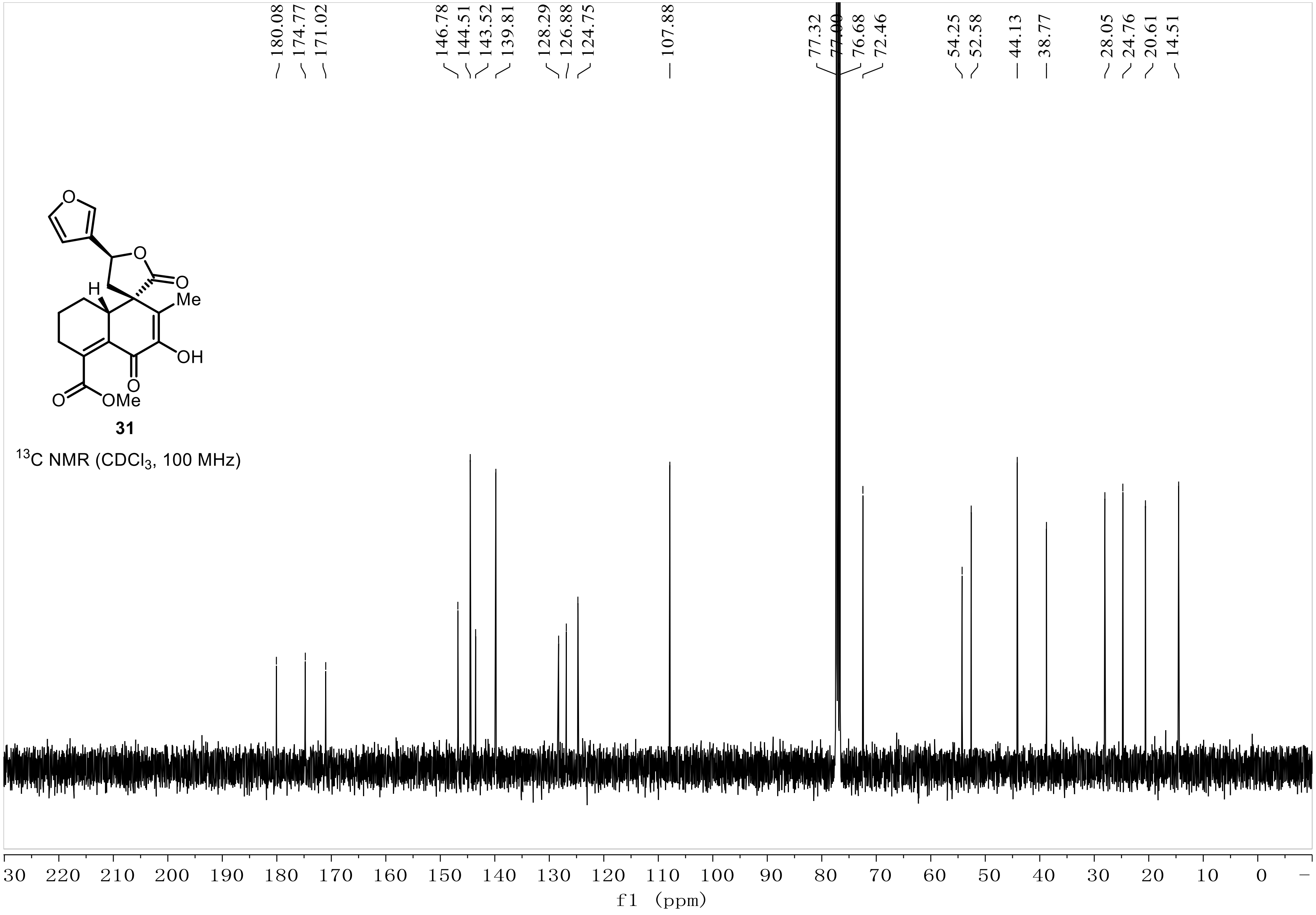

31

^{13}C NMR ($CDCl_3$, 100 MHz)

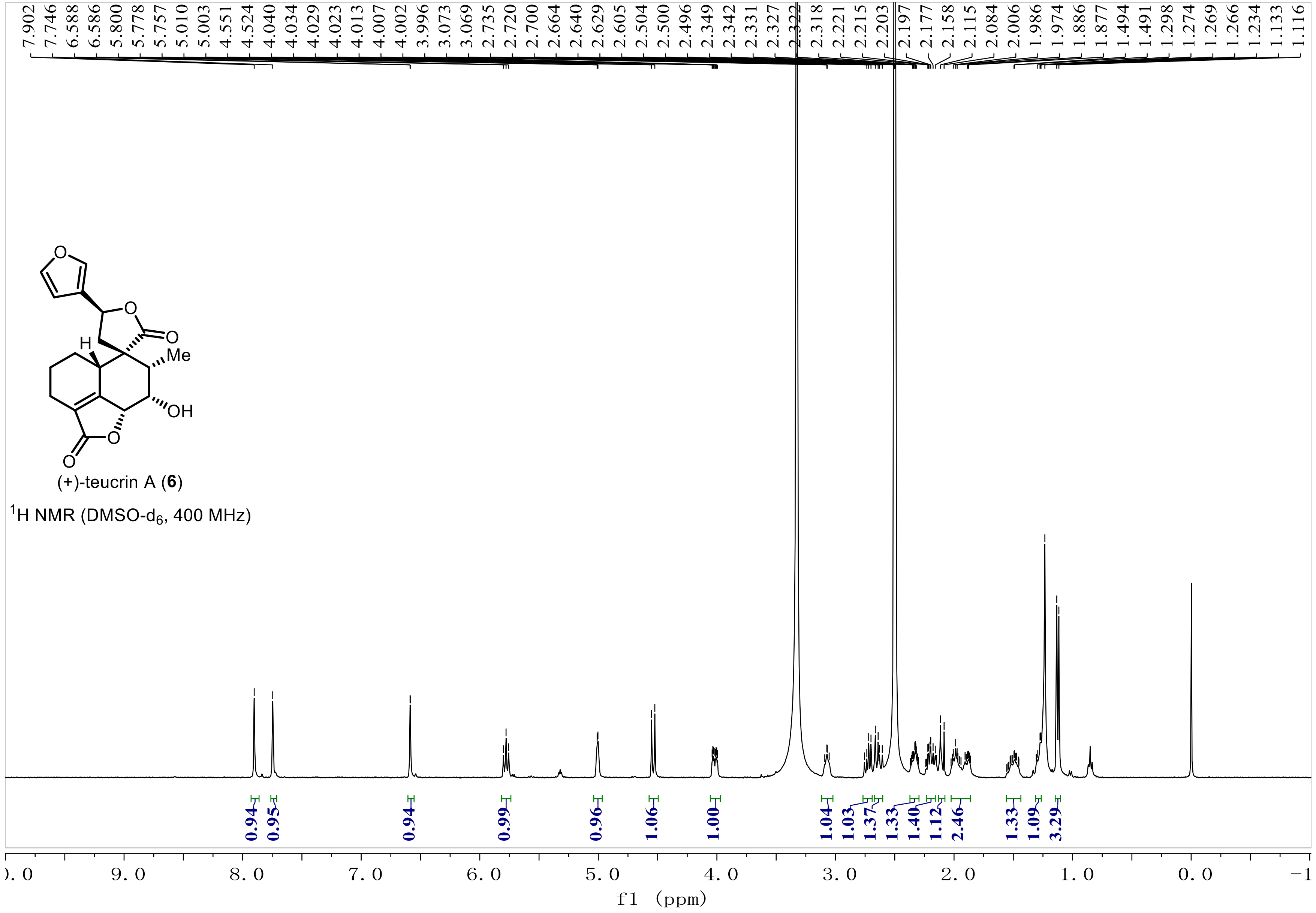

7.902
7.746
6.588
6.586
5.800
5.778
5.757
5.010
5.003
4.551
4.524
4.040
4.034
4.029
4.023
4.013
4.007
4.002
3.996
3.073
3.069
2.735
2.720
2.700
2.664
2.640
2.629
2.605
2.504
2.500
2.496
2.349
2.342
2.331
2.327
2.322
2.318
2.221
2.215
2.203
2.197
2.177
2.158
2.115
2.084
2.006
1.986
1.974
1.886
1.877
1.494
1.491
1.298
1.274
1.269
1.266
1.234
1.133
1.116
(+)-teucrin A (6)
1H NMR (DMSO-d6, 400 MHz)
0.94
0.95
0.94
0.99
0.96
1.06
1.00
1.04
1.03
1.37
1.33
1.40
1.12
2.46
1.33
1.09
3.29
9.0
8.0
7.0
6.0
5.0
4.0
3.0
2.0
1.0
0.0
f1 (ppm)

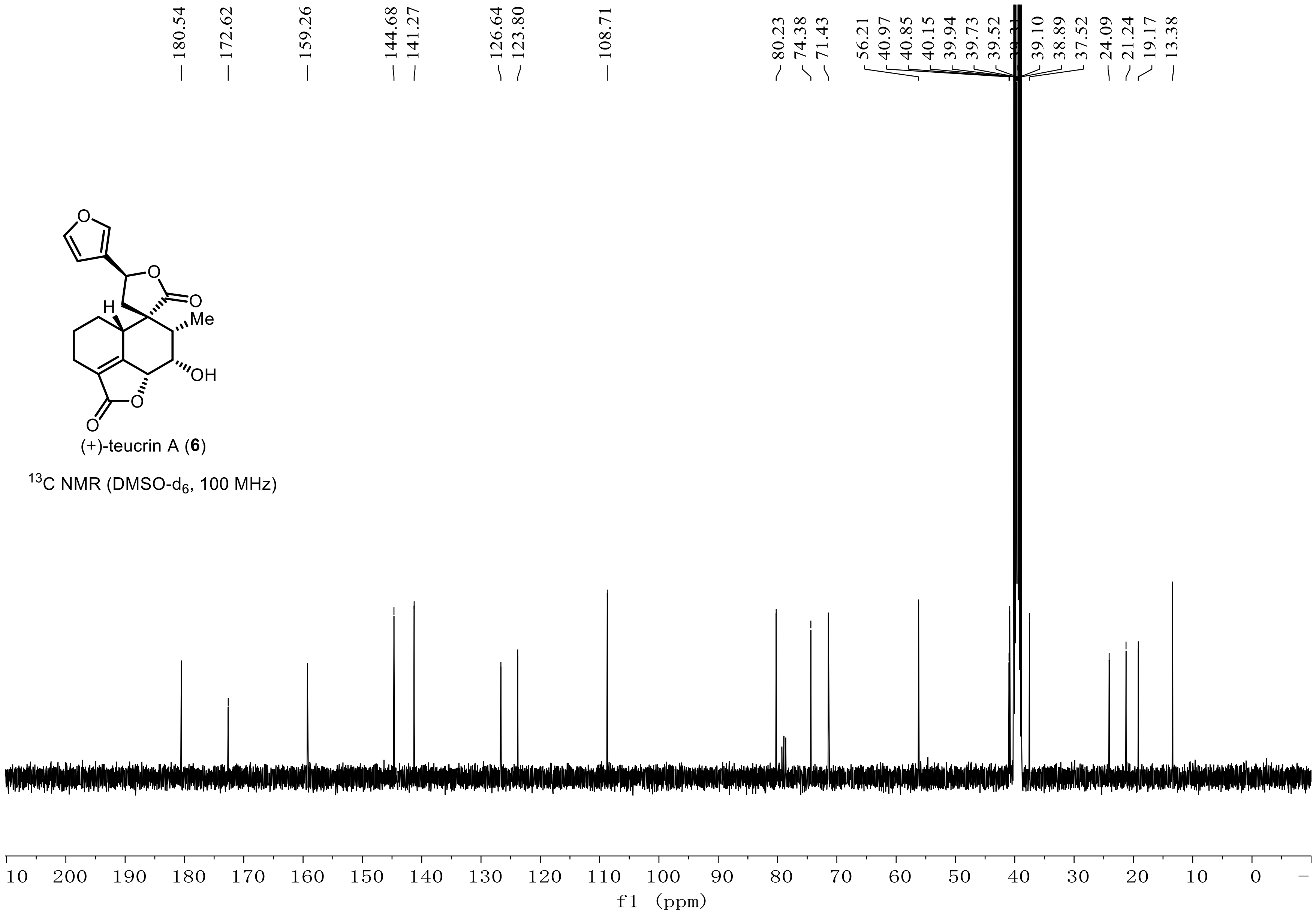

(+)-teucrin A (**6**)

^{13}C NMR (DMSO-d_6, 100 MHz)

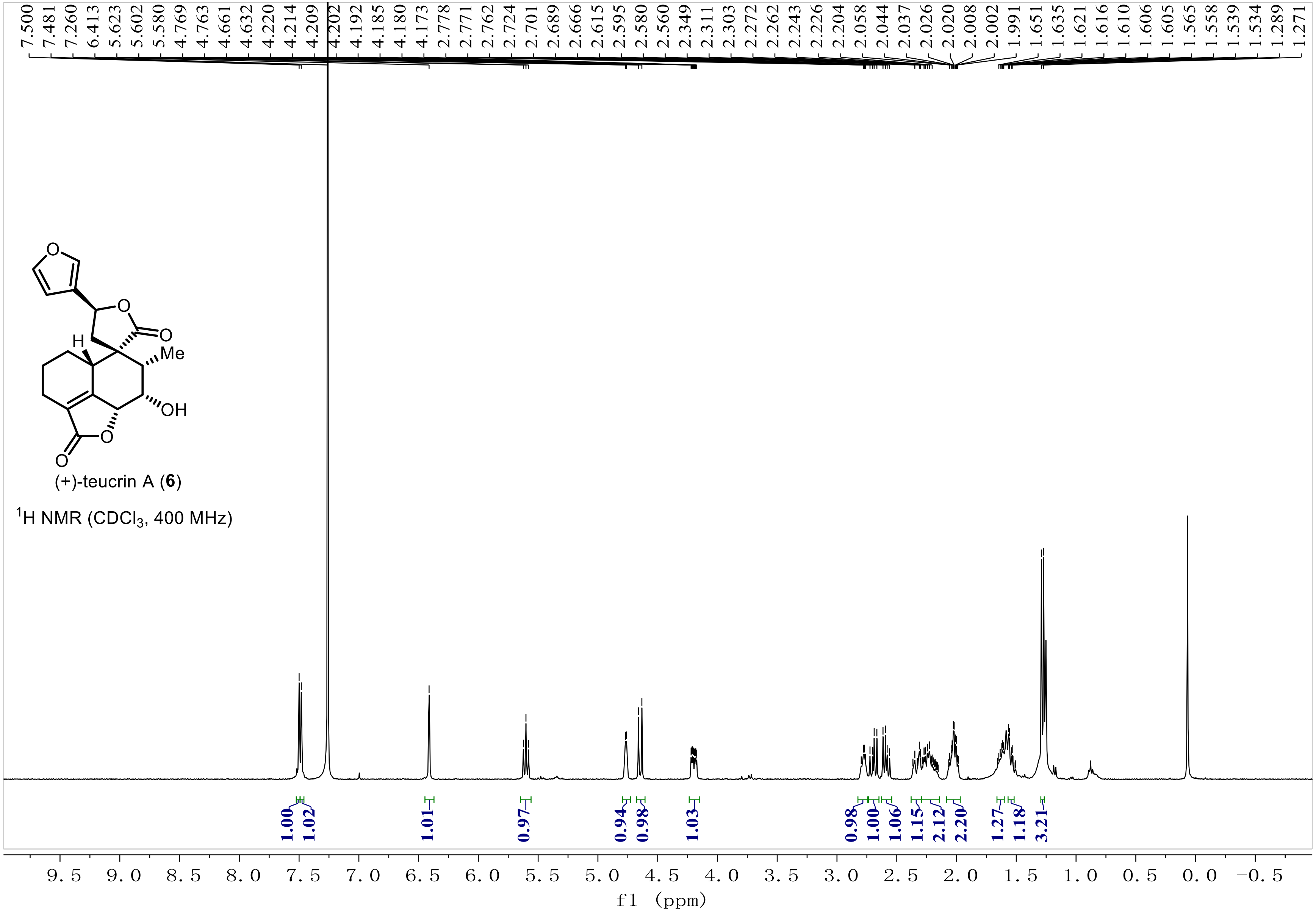
7.500 7.481 7.260 6.413 5.623 5.602 5.580 4.769 4.763 4.661 4.632 4.220 4.214 4.209 4.202 4.192 4.185 4.180 4.173 2.778 2.771 2.762 2.724 2.701 2.689 2.666 2.615 2.595 2.580 2.560 2.349 2.311 2.303 2.272 2.262 2.243 2.226 2.204 2.058 2.044 2.037 2.026 2.020 2.008 2.002 1.991 1.651 1.635 1.621 1.616 1.610 1.606 1.605 1.565 1.558 1.539 1.534 1.289 1.271
O
O
O
H
Me
OH
O
O
(+)-teucrin A (6)
1H NMR (CDCl3, 400 MHz)
1.00 1.02 1.01 0.97 0.94 0.98 1.03 0.98 1.00 1.06 1.15 2.12 2.20 1.27 1.18 3.21
9.5 9.0 8.5 8.0 7.5 7.0 6.5 6.0 5.5 5.0 4.5 4.0 3.5 3.0 2.5 2.0 1.5 1.0 0.5 0.0 -0.5
f1 (ppm)

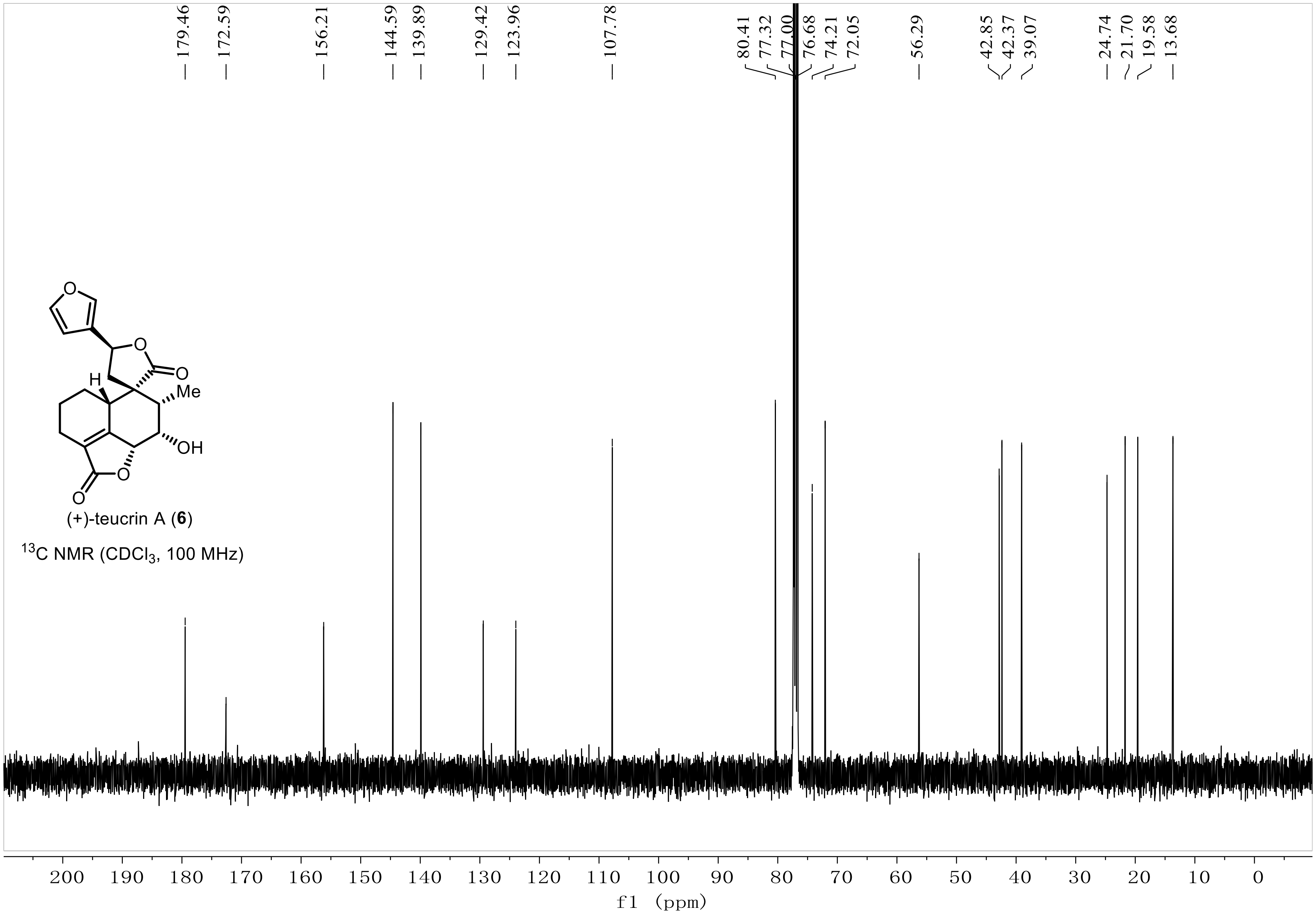
179.46
172.59
156.21
144.59
139.89
129.42
123.96
107.78
80.41
77.32
77.00
76.68
74.21
72.05
56.29
42.85
42.37
39.07
24.74
21.70
19.58
13.68
H
O
O
Me
OH
(+)-teucrin A (6)
13C NMR (CDCl3, 100 MHz)
200 190 180 170 160 150 140 130 120 110 100 90 80 70 60 50 40 30 20 10 0
f1 (ppm)

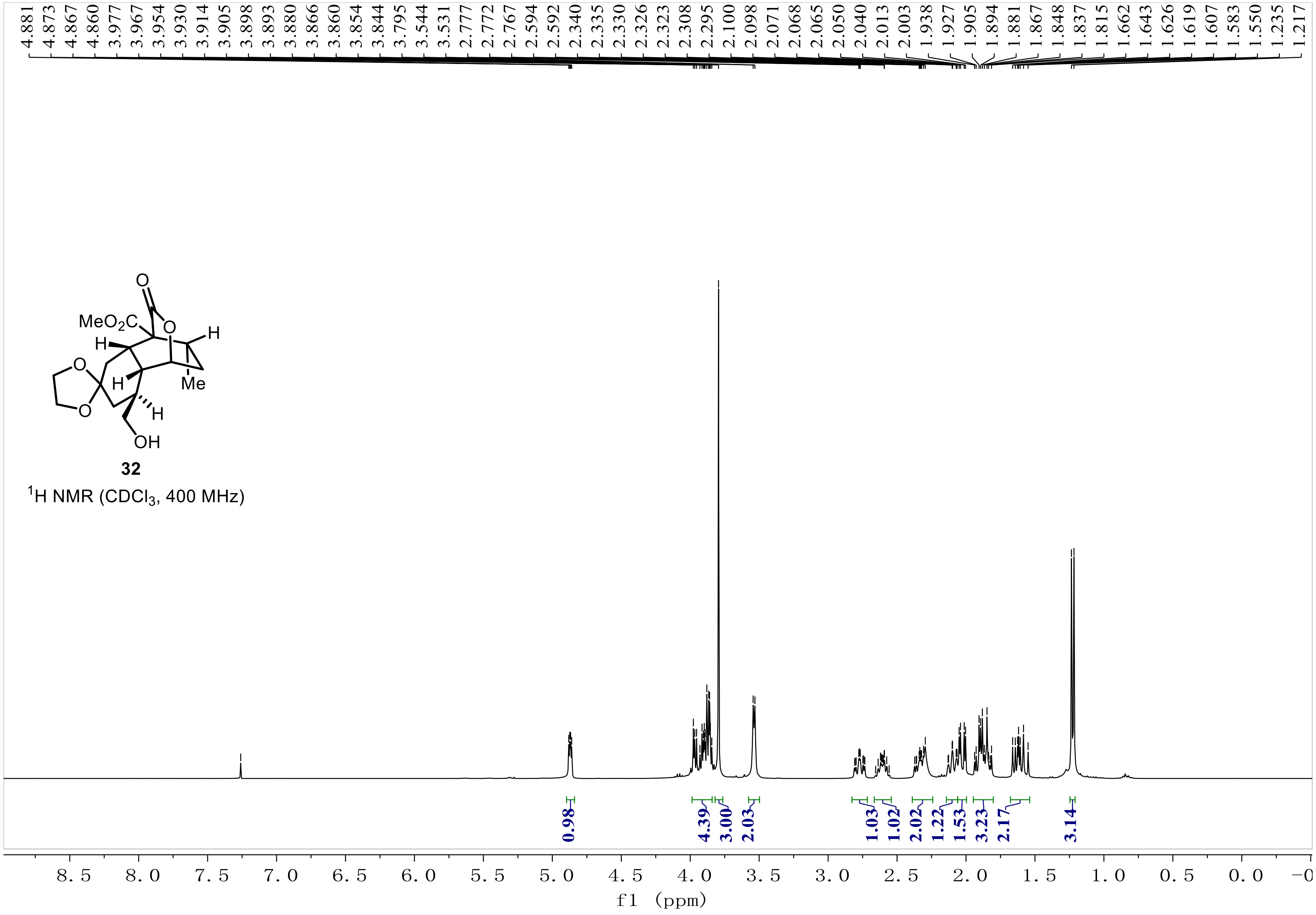
4.881 4.873 4.867 4.860 3.977 3.967 3.954 3.930 3.914 3.905 3.898 3.893 3.880 3.866 3.860 3.854 3.844 3.795 3.544 3.531 2.777 2.772 2.767 2.594 2.592 2.340 2.335 2.330 2.326 2.323 2.308 2.295 2.100 2.098 2.071 2.068 2.065 2.050 2.040 2.013 2.003 1.938 1.927 1.905 1.894 1.881 1.867 1.848 1.837 1.815 1.662 1.643 1.626 1.619 1.607 1.583 1.550 1.235 1.217
MeO2C
O
H
Me
OH
32
1H NMR (CDCl3, 400 MHz)
0.98 4.39 3.00 2.03 1.03 1.02 2.02 1.22 1.53 3.23 2.17 3.14
8.5 8.0 7.5 7.0 6.5 6.0 5.5 5.0 4.5 4.0 3.5 3.0 2.5 2.0 1.5 1.0 0.5 0.0 -0
f1 (ppm)

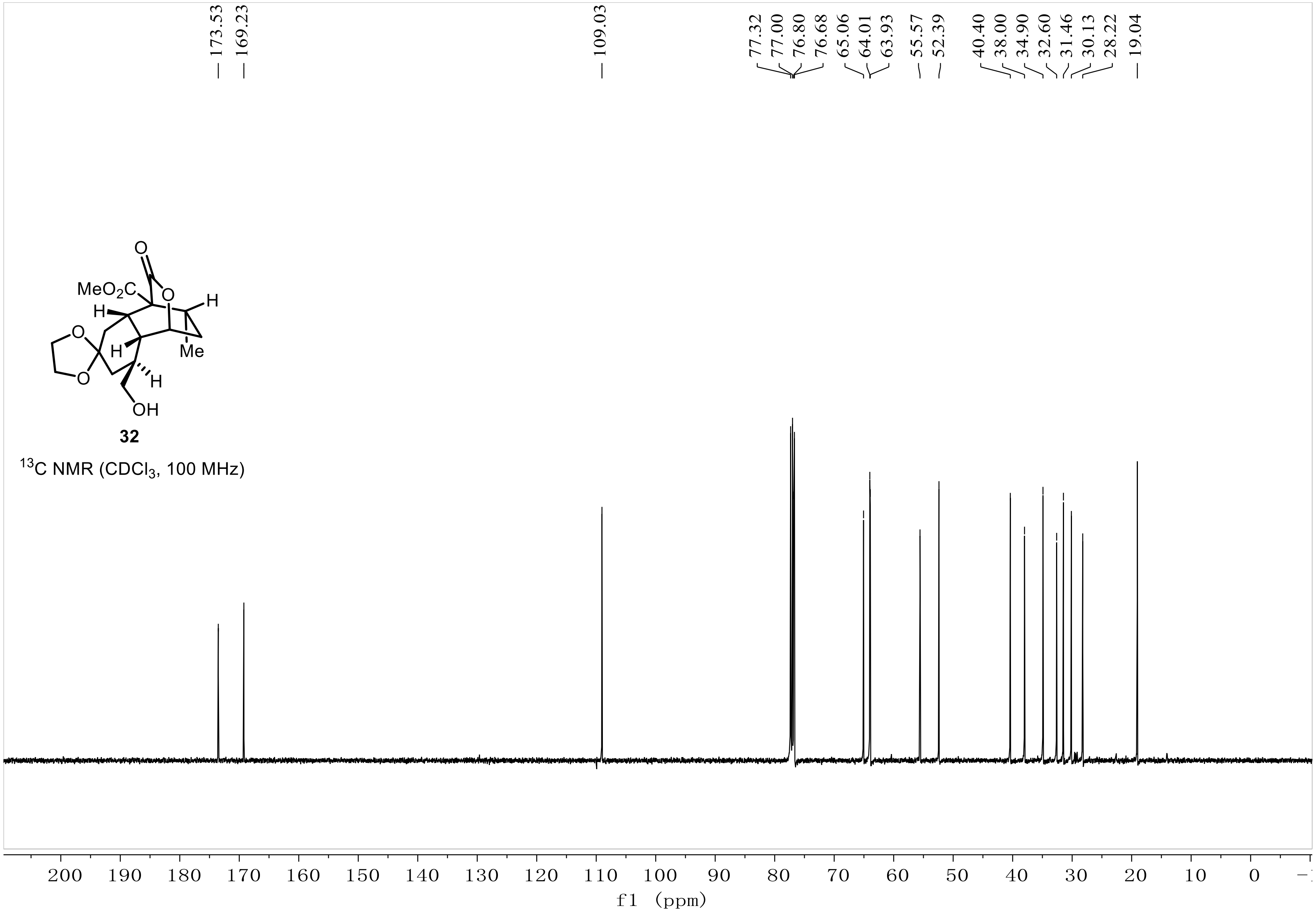
173.53
169.23
109.03
77.32
77.00
76.80
76.68
65.06
64.01
63.93
55.57
52.39
40.40
38.00
34.90
32.60
31.46
30.13
28.22
19.04
MeO2C
O
O
H
H
H
H
Me
O
O
OH
32
13C NMR (CDCl3, 100 MHz)
200 190 180 170 160 150 140 130 120 110 100 90 80 70 60 50 40 30 20 10 0
f1 (ppm)

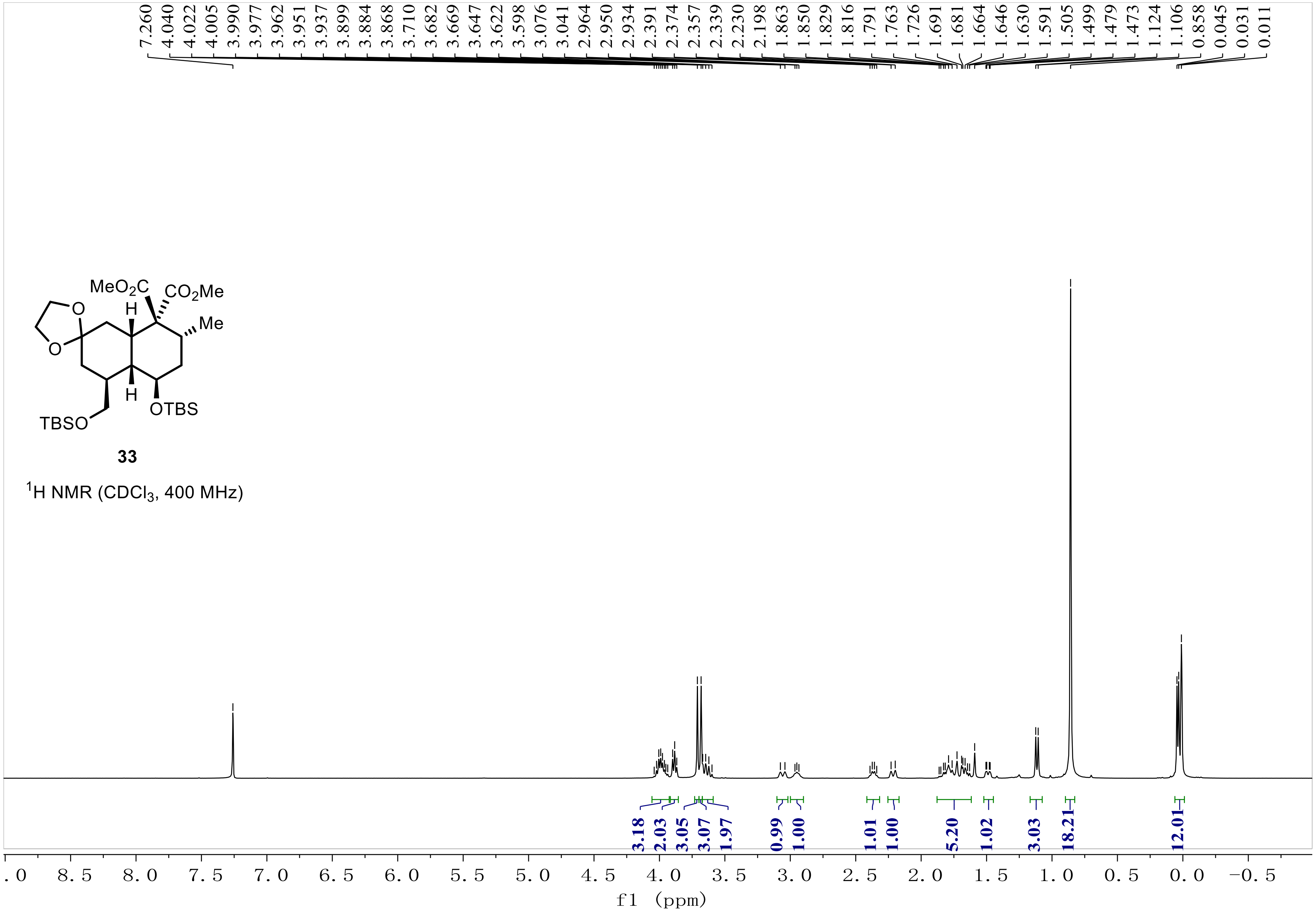
7.260 4.040 4.022 4.005 3.990 3.977 3.962 3.951 3.937 3.899 3.884 3.868 3.710 3.682 3.669 3.647 3.622 3.598 3.076 3.041 2.964 2.950 2.934 2.391 2.374 2.357 2.339 2.230 2.198 1.863 1.850 1.829 1.816 1.791 1.763 1.726 1.691 1.681 1.664 1.646 1.630 1.591 1.505 1.499 1.479 1.473 1.124 1.106 0.858 0.045 0.031 0.011
MeO2C
CO2Me
H
Me
O
O
H
OTBS
TBSO
33
1H NMR (CDCl3, 400 MHz)
3.18 2.03 3.05 3.07 1.97 0.99 1.00 1.01 1.00 5.20 1.02 3.03 18.21 12.01
f1 (ppm)

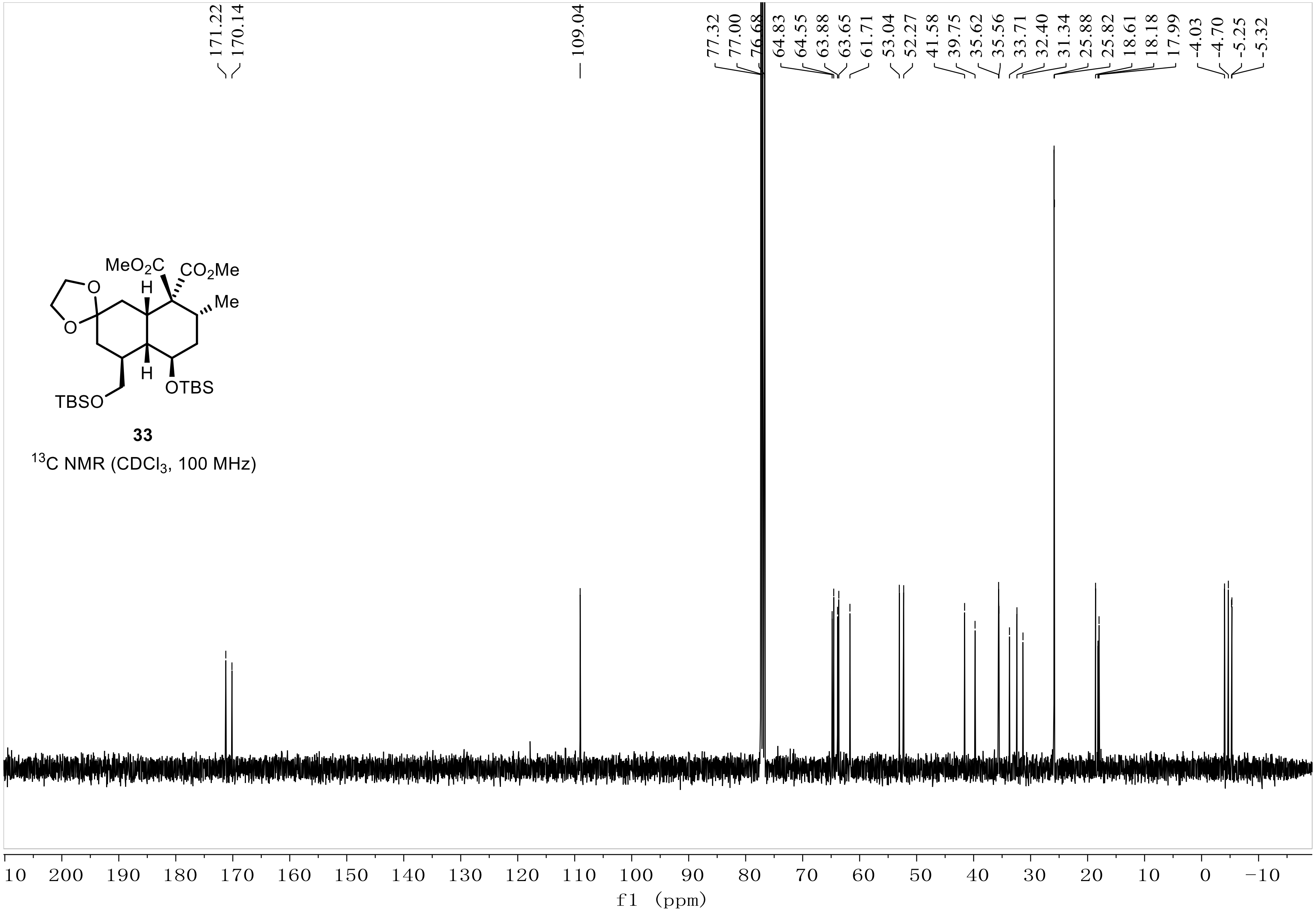
171.22
170.14
109.04
77.32
77.00
76.68
64.83
64.55
63.88
63.65
61.71
53.04
52.27
41.58
39.75
35.62
35.56
33.71
32.40
31.34
25.88
25.82
18.61
18.18
17.99
-4.03
-4.70
-5.25
-5.32
MeO2C
CO2Me
H
Me
O
O
H
OTBS
TBSO
33
13C NMR (CDCl3, 100 MHz)
10 200 190 180 170 160 150 140 130 120 110 100 90 80 70 60 50 40 30 20 10 0 −10
f1 (ppm)

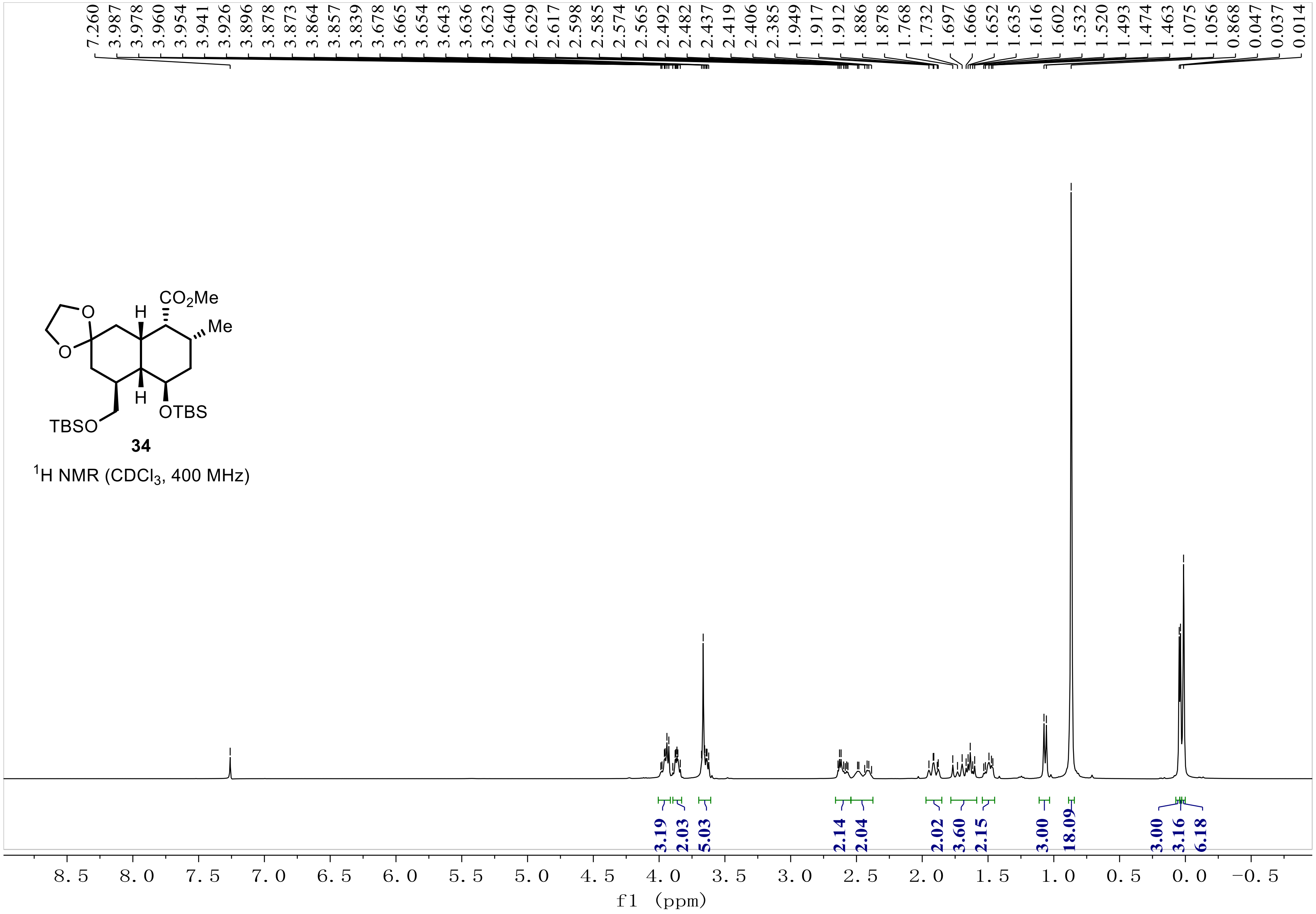
7.260 3.987 3.978 3.960 3.954 3.941 3.926 3.896 3.878 3.873 3.864 3.857 3.839 3.678 3.665 3.654 3.643 3.636 3.623 2.640 2.629 2.617 2.598 2.585 2.574 2.565 2.492 2.482 2.437 2.419 2.406 2.385 1.949 1.917 1.912 1.886 1.878 1.768 1.732 1.697 1.666 1.652 1.635 1.616 1.602 1.532 1.520 1.493 1.474 1.463 1.075 1.056 0.868 0.047 0.037 0.014
CO2Me
Me
H
O
O
H
OTBS
TBSO
34
1H NMR (CDCl3, 400 MHz)
3.19 2.03 5.03 2.14 2.04 2.02 3.60 2.15 3.00 18.09 3.00 3.16 6.18
8.5 8.0 7.5 7.0 6.5 6.0 5.5 5.0 4.5 4.0 3.5 3.0 2.5 2.0 1.5 1.0 0.5 0.0 −0.5
f1 (ppm)

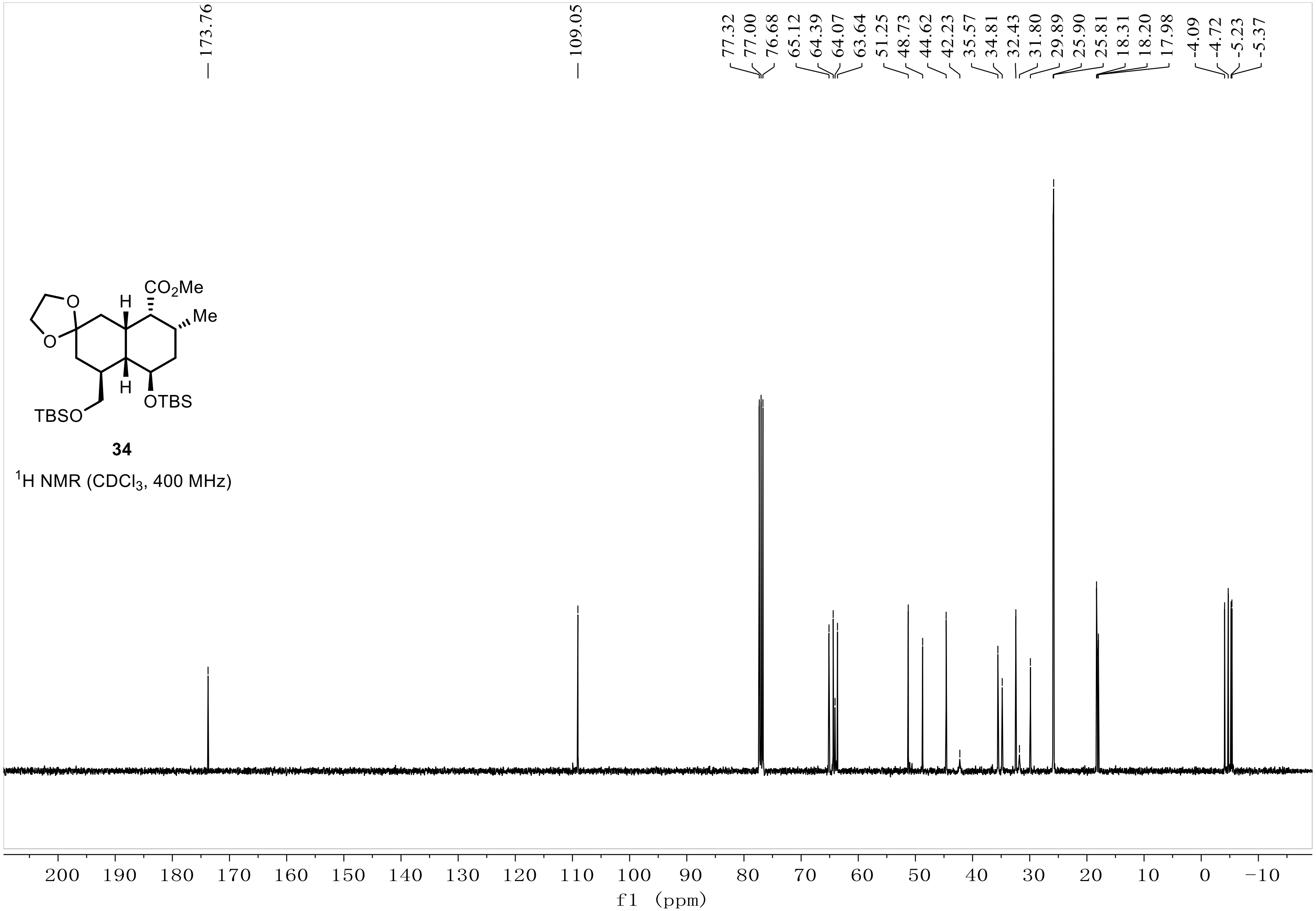
173.76
109.05
77.32
77.00
76.68
65.12
64.39
64.07
63.64
51.25
48.73
44.62
42.23
35.57
34.81
32.43
31.80
29.89
25.90
25.81
18.31
18.20
17.98
-4.09
-4.72
-5.23
-5.37
CO2Me
Me
H
H
O
O
OTBS
TBSO
34
1H NMR (CDCl3, 400 MHz)
200 190 180 170 160 150 140 130 120 110 100 90 80 70 60 50 40 30 20 10 0 −10
f1 (ppm)

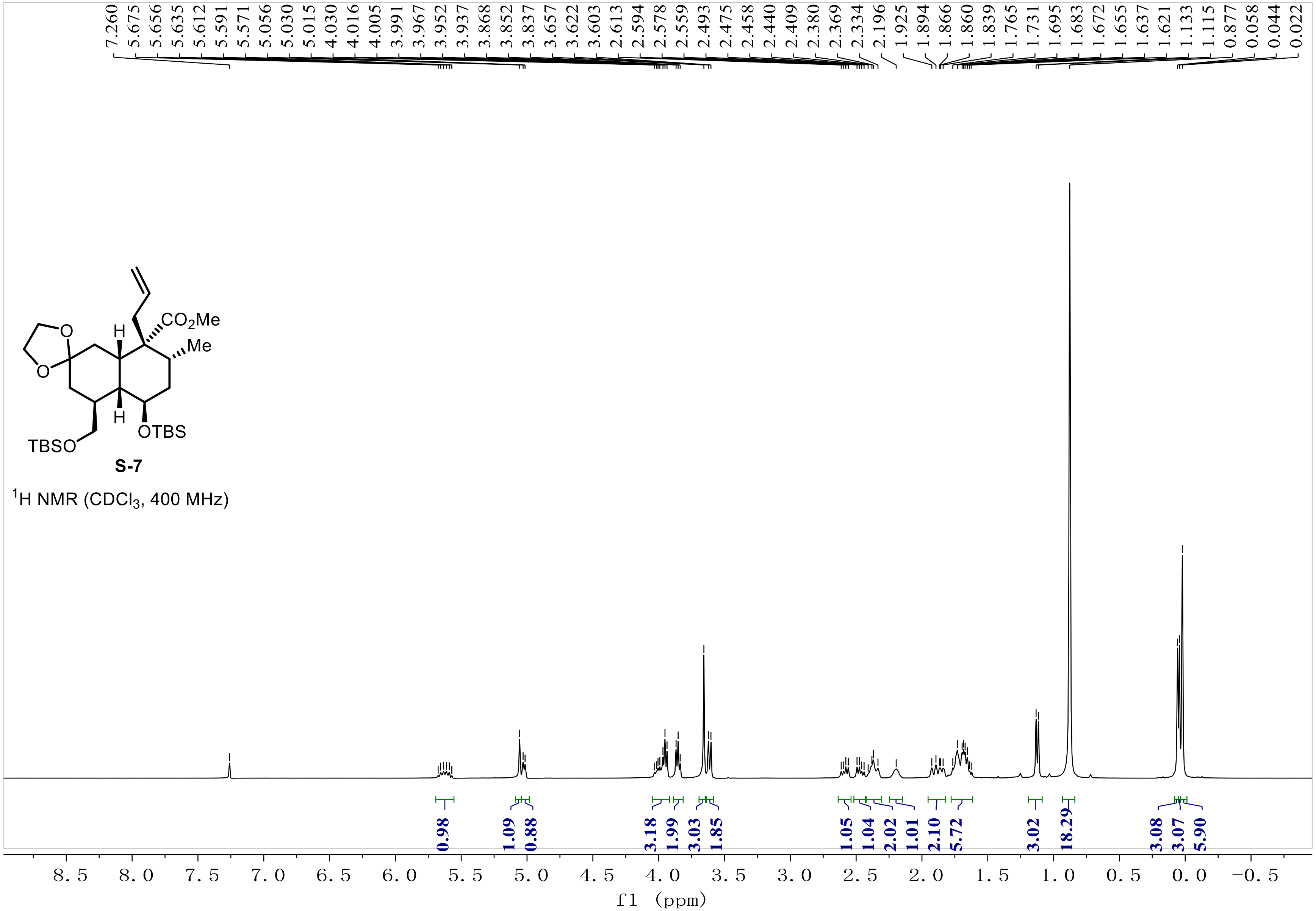
7.260 5.675 5.656 5.635 5.612 5.591 5.571 5.056 5.030 5.015 4.030 4.016 4.005 3.991 3.967 3.952 3.937 3.868 3.852 3.837 3.657 3.622 3.603 2.613 2.594 2.578 2.559 2.493 2.475 2.458 2.440 2.409 2.380 2.369 2.334 2.196 1.925 1.894 1.866 1.860 1.839 1.765 1.731 1.695 1.683 1.672 1.655 1.637 1.621 1.133 1.115 0.877 0.058 0.044 0.022
CO2Me
Me
H
O
O
H
OTBS
TBSO
S-7
1H NMR (CDCl3, 400 MHz)
0.98 1.09 0.88 3.18 1.99 3.03 1.85 1.05 1.04 2.02 1.01 2.10 5.72 3.02 18.29 3.08 3.07 5.90
8.5 8.0 7.5 7.0 6.5 6.0 5.5 5.0 4.5 4.0 3.5 3.0 2.5 2.0 1.5 1.0 0.5 0.0 −0.5
f1 (ppm)

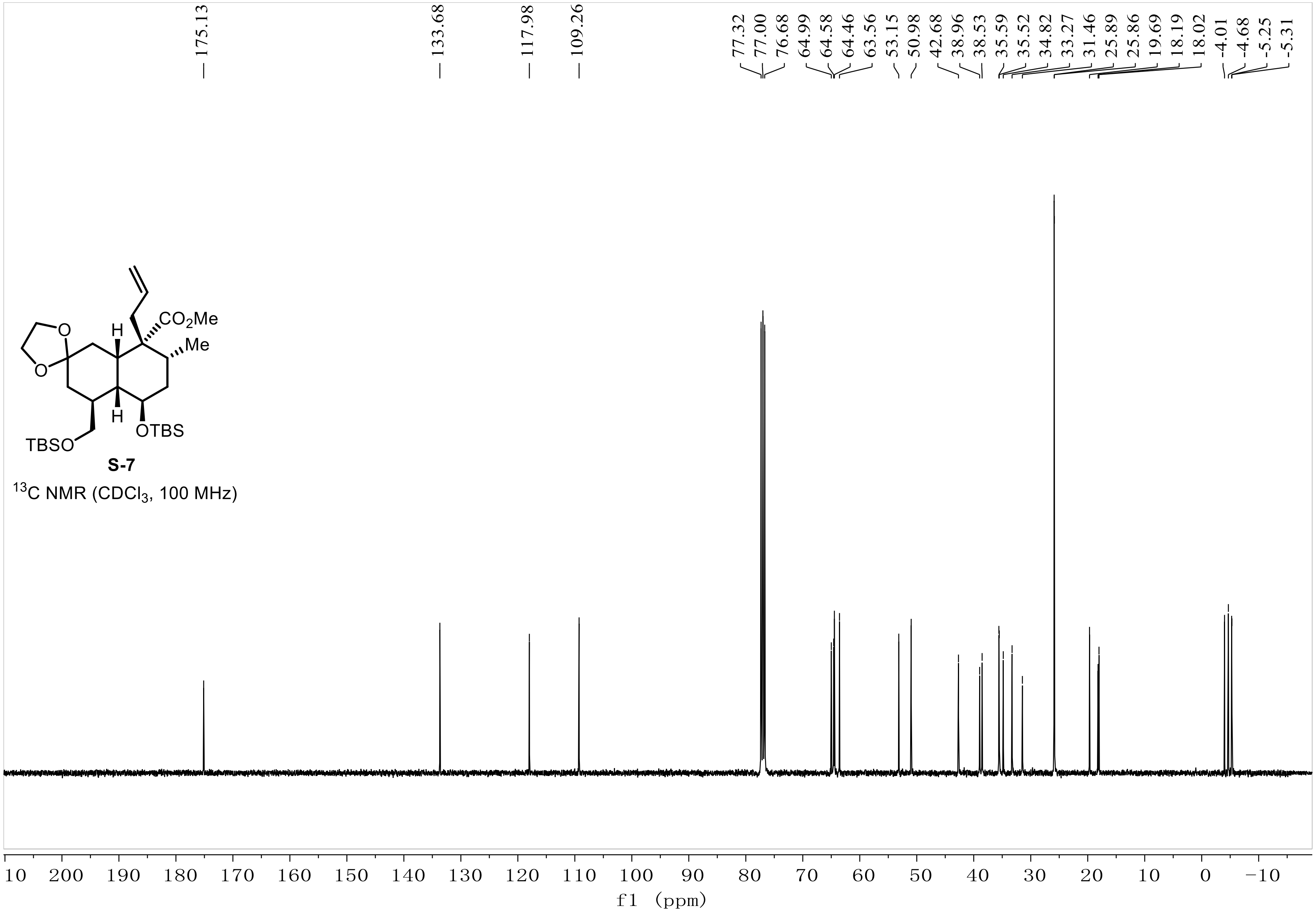
175.13
133.68
117.98
109.26
77.32
77.00
76.68
64.99
64.58
64.46
63.56
53.15
50.98
42.68
38.96
38.53
35.59
35.52
34.82
33.27
31.46
25.89
25.86
19.69
18.19
18.02
-4.01
-4.68
-5.25
-5.31
CO2Me
Me
O
O
H
H
OTBS
TBSO
S-7
13C NMR (CDCl3, 100 MHz)
10 200 190 180 170 160 150 140 130 120 110 100 90 80 70 60 50 40 30 20 10 0 −10
f1 (ppm)

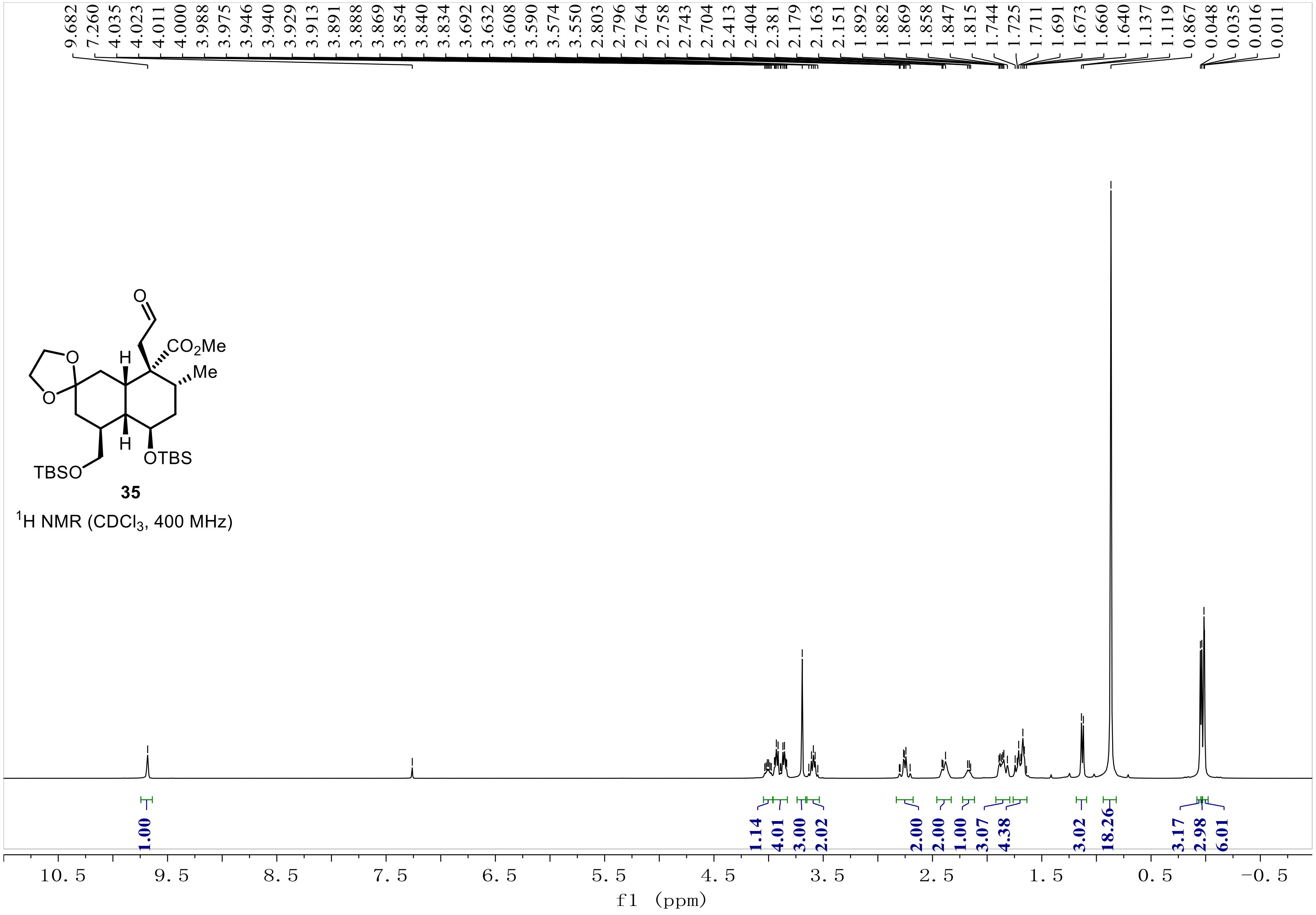

35

^{1}H NMR ($CDCl_3$, 400 MHz)

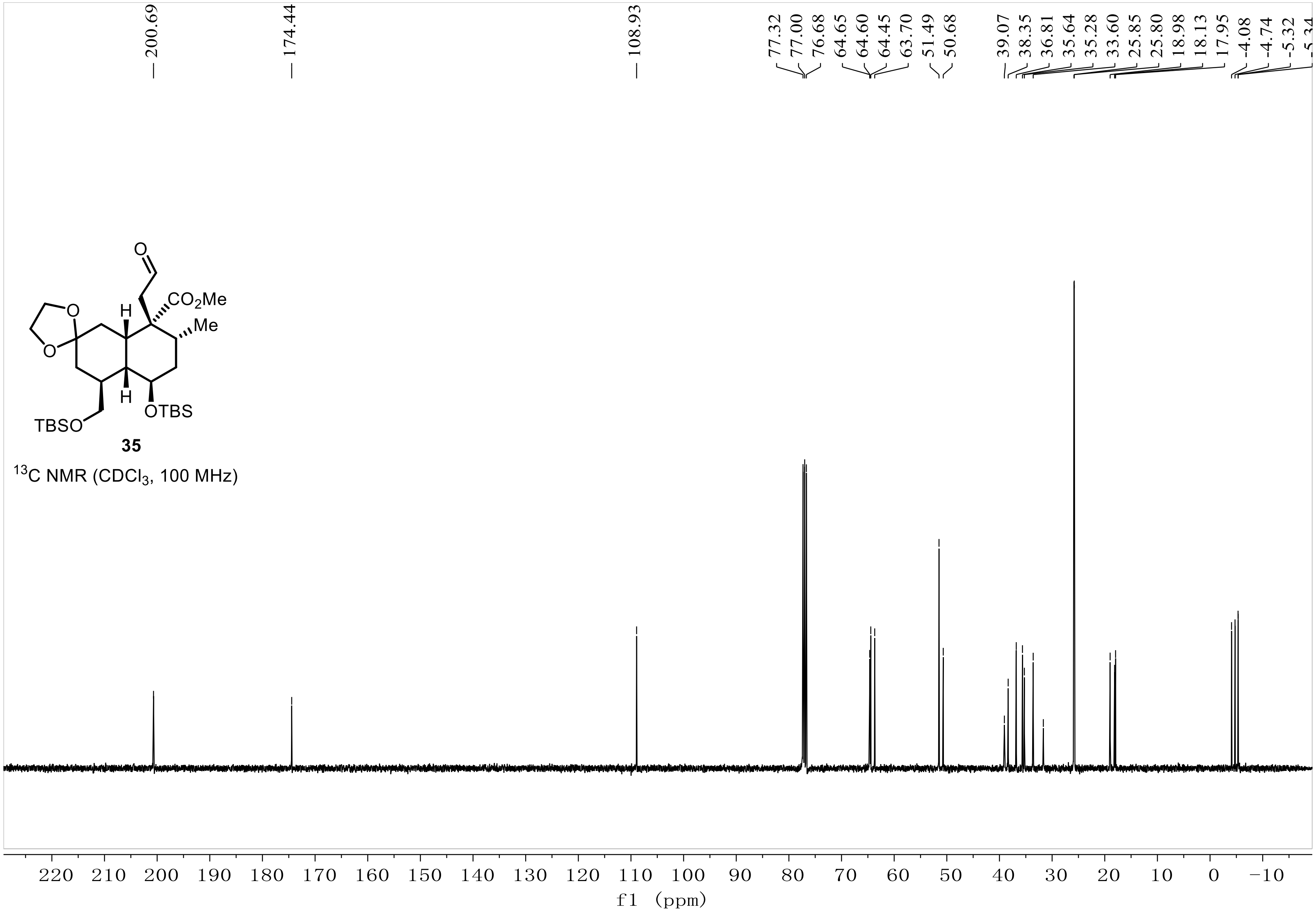
200.69
174.44
108.93
77.32
77.00
76.68
64.65
64.60
64.45
63.70
51.49
50.68
39.07
38.35
36.81
35.64
35.28
33.60
25.85
25.80
18.98
18.13
17.95
-4.08
-4.74
-5.32
O
CO_2Me
Me
O
O
H
H
OTBS
TBSO
35
^{13}C NMR ($CDCl_3$, 100 MHz)
220 210 200 190 180 170 160 150 140 130 120 110 100 90 80 70 60 50 40 30 20 10 0 −10
f1 (ppm)

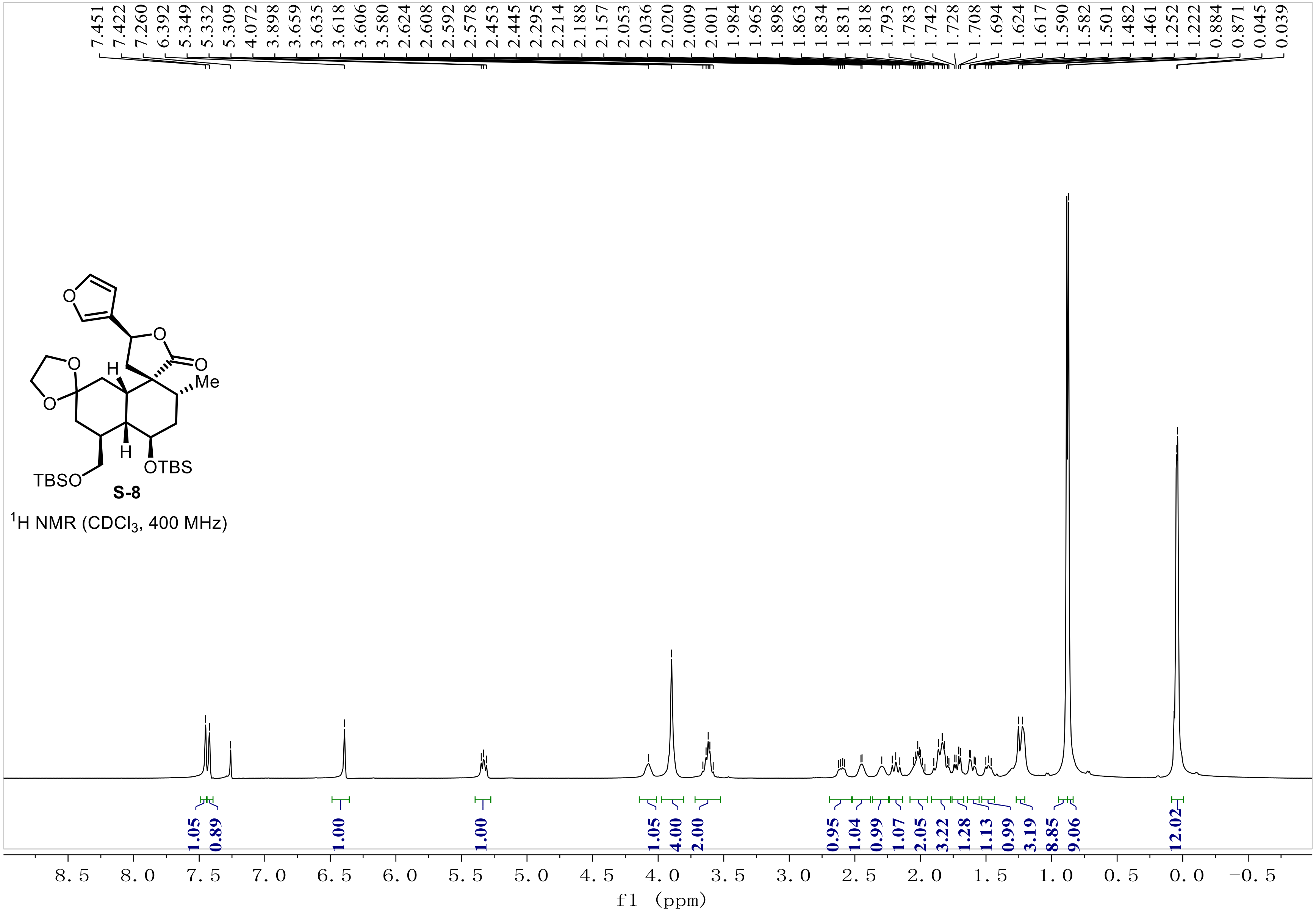

S-8

^{1}H NMR ($CDCl_3$, 400 MHz)

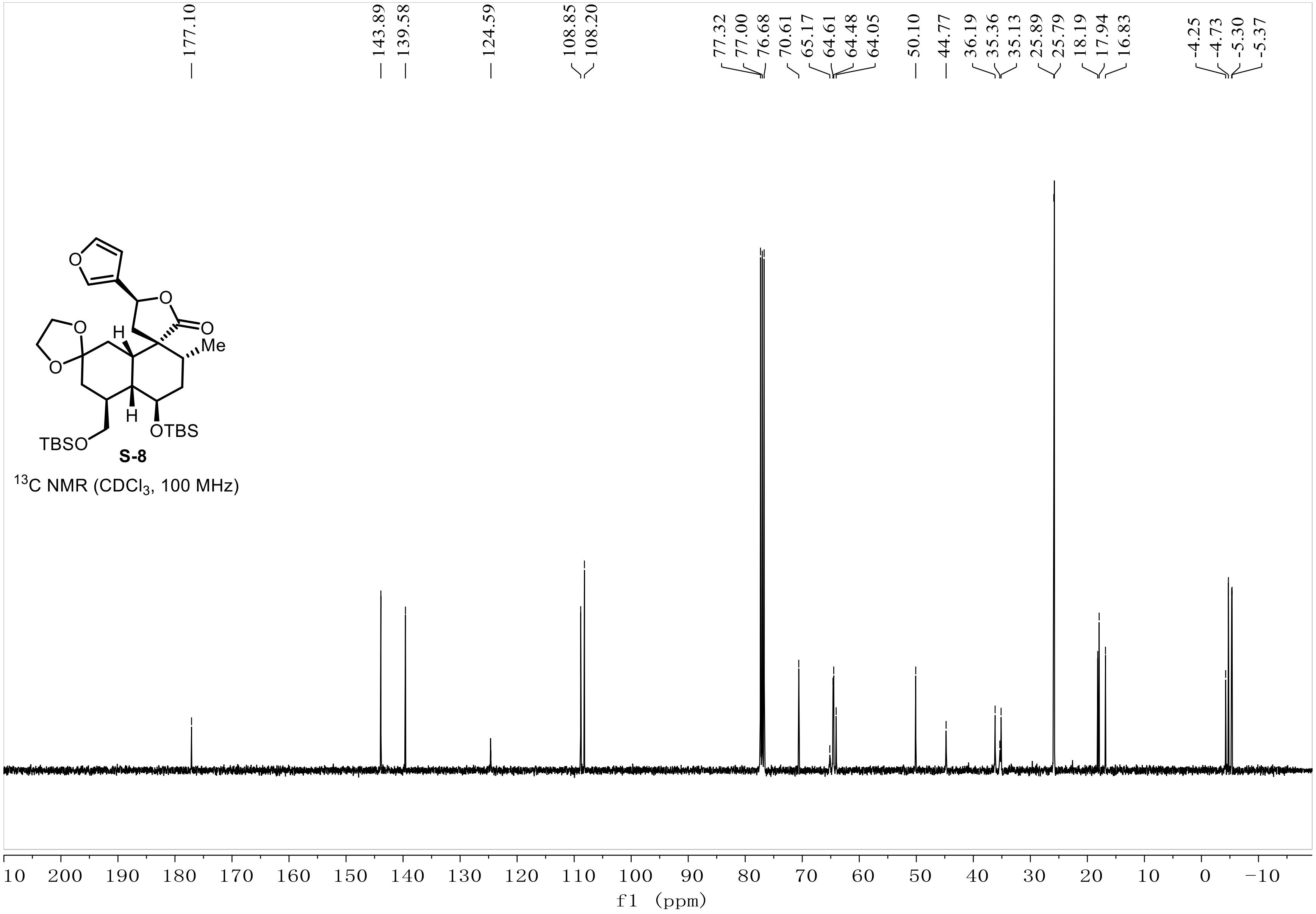

177.10
143.89
139.58
124.59
108.85
108.20
77.32
77.00
76.68
70.61
65.17
64.61
64.48
64.05
50.10
44.77
36.19
35.36
35.13
25.89
25.79
18.19
17.94
16.83
-4.25
-4.73
-5.30
-5.37
O
O
O
O
O
H
Me
H
OTBS
TBSO
S-8
^{13}C NMR (CDCl$_3$, 100 MHz)
10 200 190 180 170 160 150 140 130 120 110 100 90 80 70 60 50 40 30 20 10 0 −10
f1 (ppm)

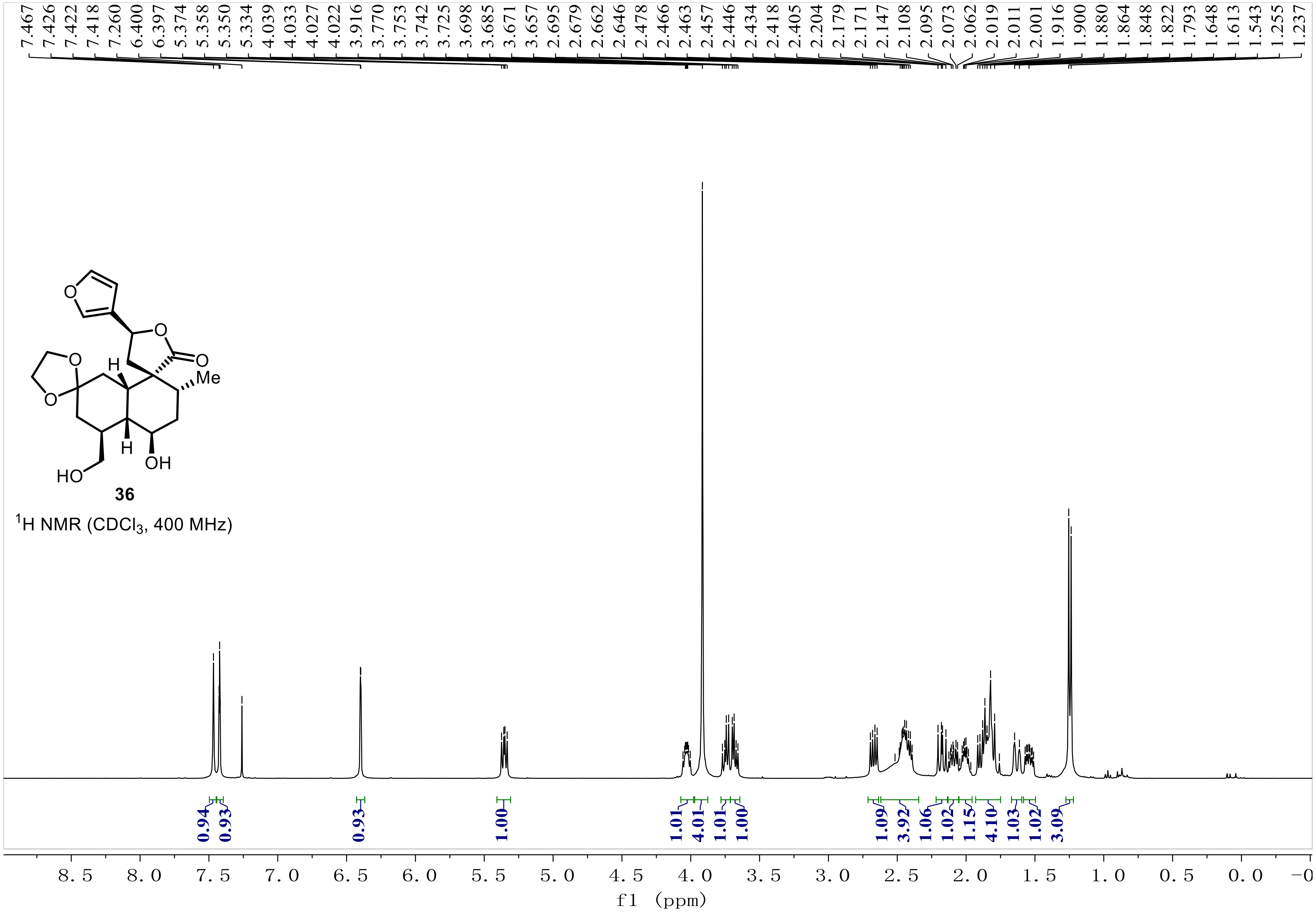
7.467 7.426 7.422 7.418 7.260 6.400 6.397 5.374 5.358 5.350 5.334 4.039 4.033 4.027 4.022 3.916 3.770 3.753 3.742 3.725 3.698 3.685 3.671 3.657 2.695 2.679 2.662 2.646 2.478 2.466 2.463 2.457 2.446 2.434 2.418 2.405 2.204 2.179 2.171 2.147 2.108 2.095 2.073 2.062 2.019 2.011 2.001 1.916 1.900 1.880 1.864 1.848 1.822 1.793 1.648 1.613 1.543 1.255 1.237
Me
H
O
OH
HO
36
1H NMR (CDCl3, 400 MHz)
0.94 0.93 0.93 1.00 1.01 4.01 1.01 1.00 1.09 3.92 1.06 1.02 1.15 4.10 1.03 1.02 3.09
8.5 8.0 7.5 7.0 6.5 6.0 5.5 5.0 4.5 4.0 3.5 3.0 2.5 2.0 1.5 1.0 0.5 0.0
f1 (ppm)

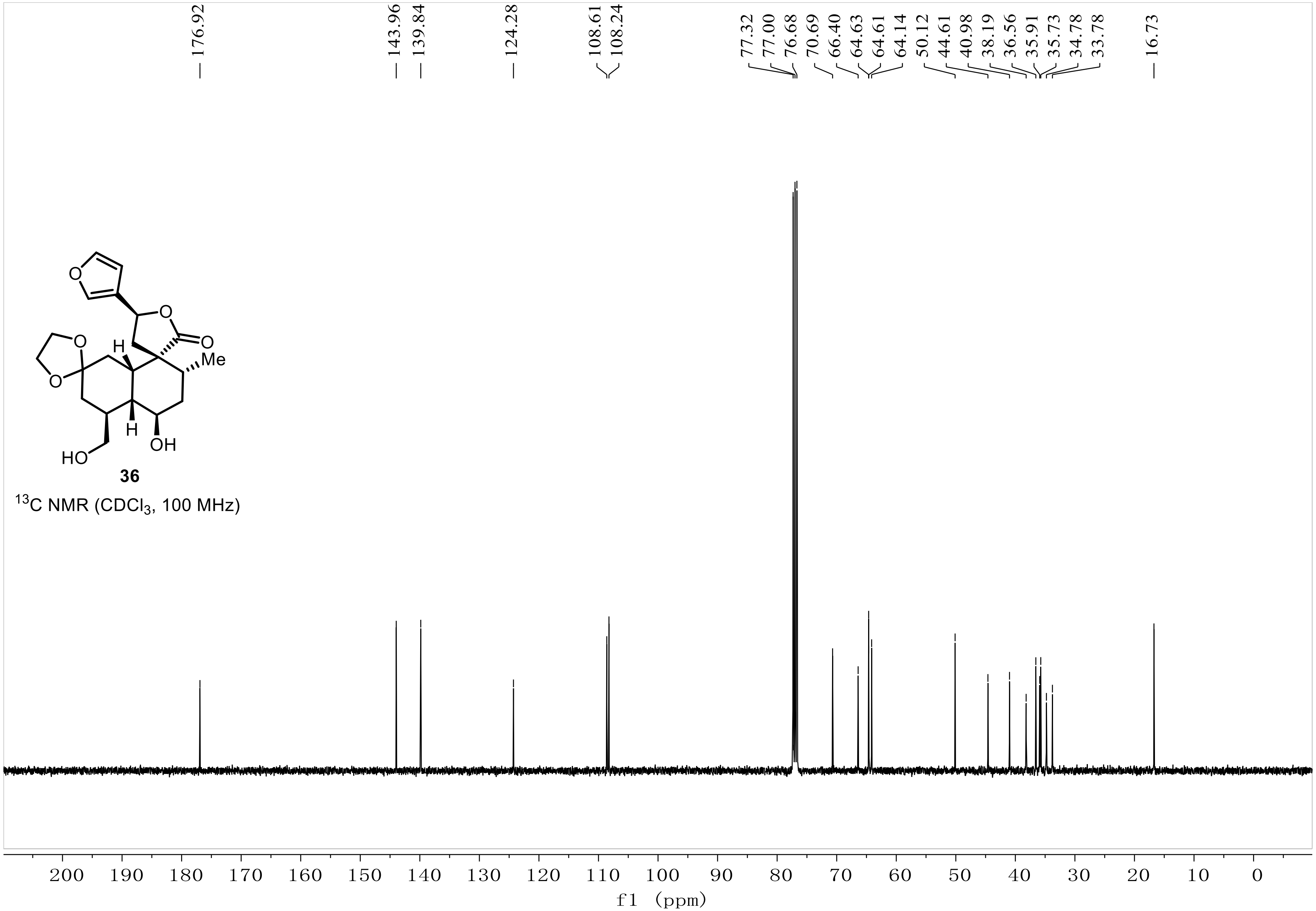
176.92
143.96
139.84
124.28
108.61
108.24
77.32
77.00
76.68
70.69
66.40
64.63
64.61
64.14
50.12
44.61
40.98
38.19
36.56
35.91
35.73
34.78
33.78
16.73
O
O
O
O
O
H
H
Me
OH
HO
36
^{13}C NMR ($CDCl_3$, 100 MHz)
200 190 180 170 160 150 140 130 120 110 100 90 80 70 60 50 40 30 20 10 0
f1 (ppm)

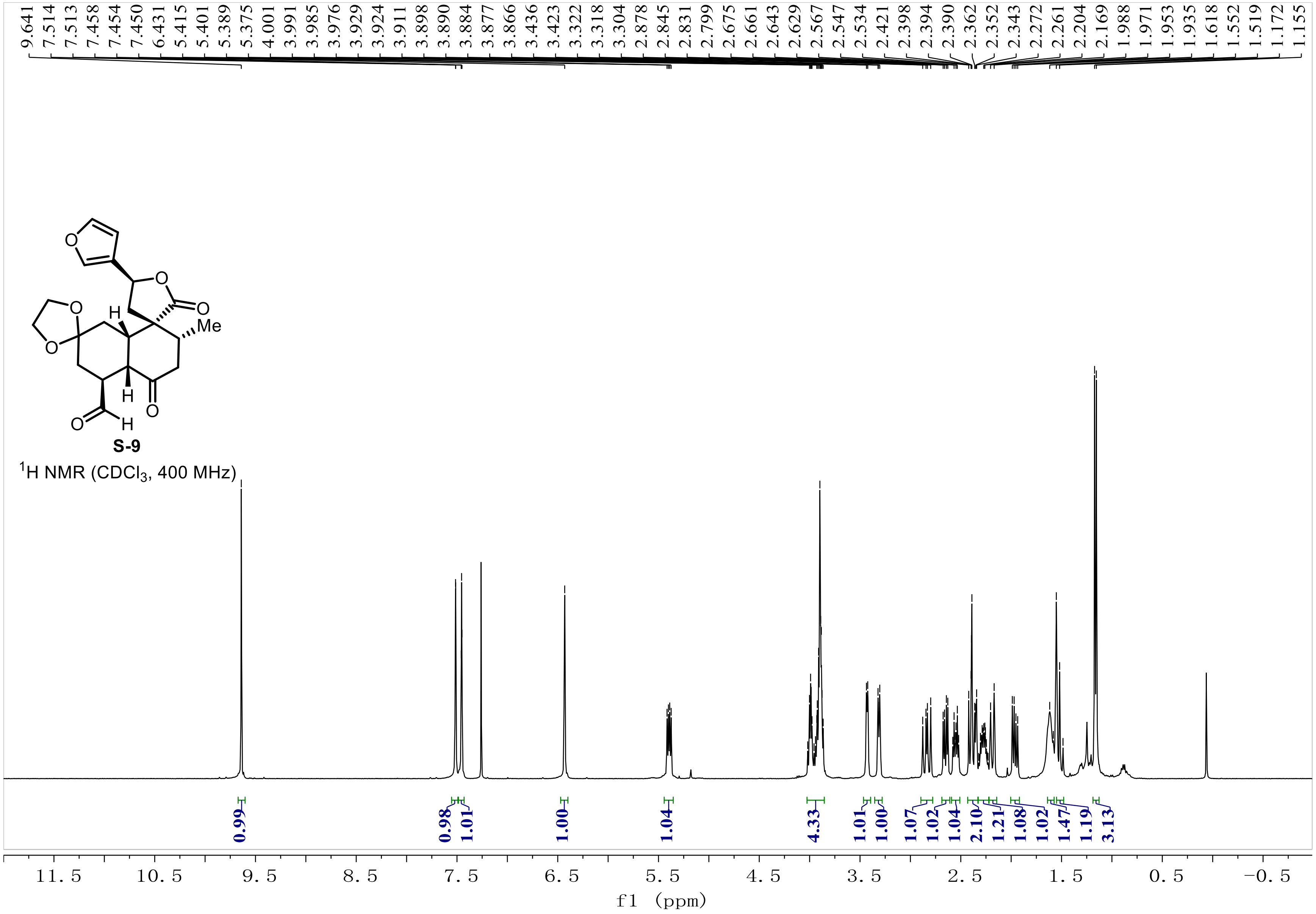
9.641 7.514 7.513 7.458 7.454 7.450 6.431 5.415 5.401 5.389 5.375 4.001 3.991 3.985 3.976 3.929 3.924 3.911 3.898 3.890 3.884 3.877 3.866 3.436 3.423 3.322 3.318 3.304 2.878 2.845 2.831 2.799 2.675 2.661 2.643 2.629 2.567 2.547 2.534 2.421 2.398 2.394 2.390 2.362 2.352 2.343 2.272 2.261 2.204 2.169 1.988 1.971 1.953 1.935 1.618 1.552 1.519 1.172 1.155
O
O
O
H
Me
O
O
H
O
O
H
S-9
1H NMR (CDCl3, 400 MHz)
0.99
0.98
1.01
1.00
1.04
4.33
1.01
1.00
1.07
1.02
1.04
2.10
1.21
1.08
1.02
1.47
1.19
3.13
11.5
10.5
9.5
8.5
7.5
6.5
5.5
4.5
3.5
2.5
1.5
0.5
−0.5
f1 (ppm)

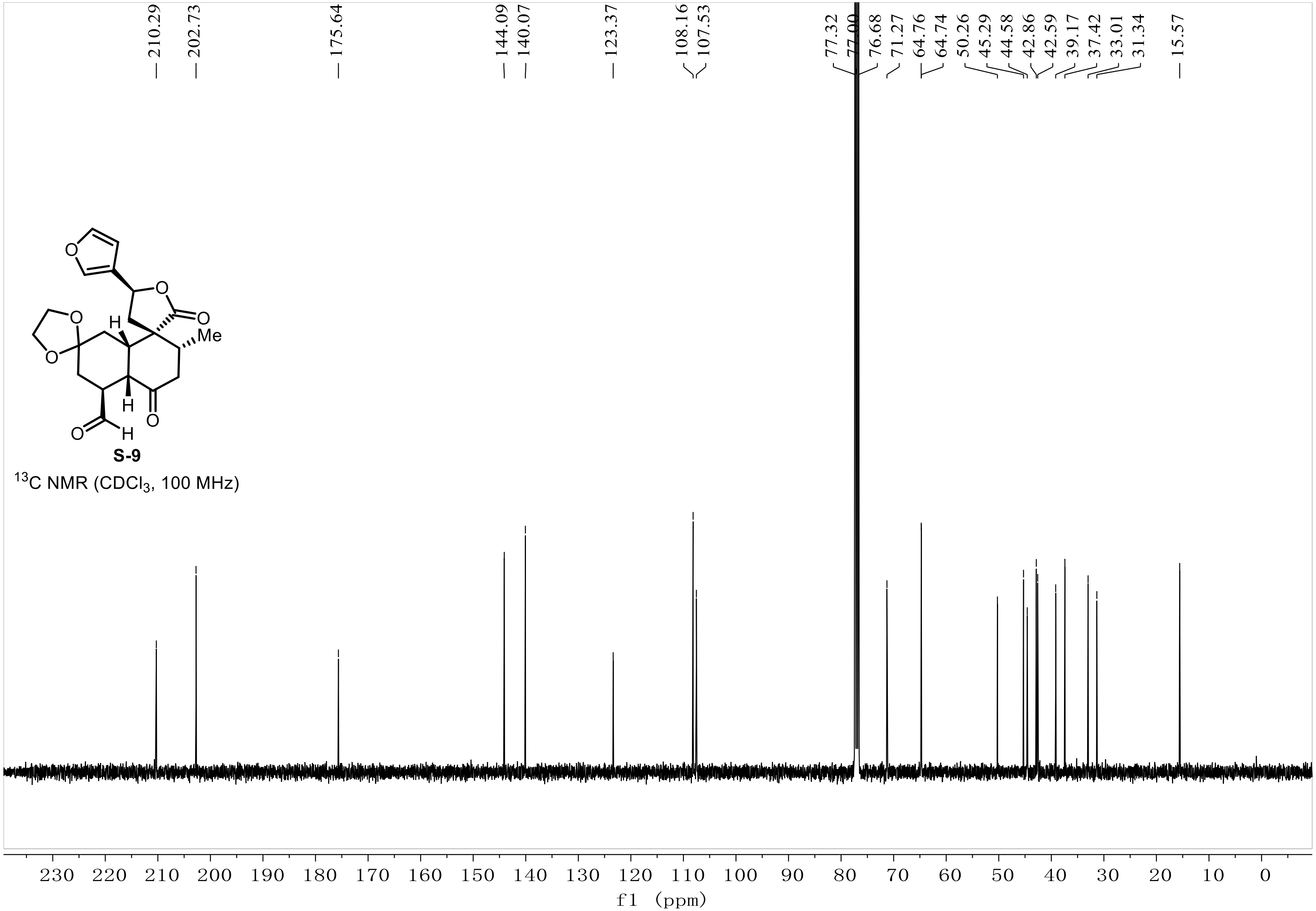
210.29
202.73
175.64
144.09
140.07
123.37
108.16
107.53
77.32
77.00
76.68
71.27
64.76
64.74
50.26
45.29
44.58
42.86
42.59
39.17
37.42
33.01
31.34
15.57
O
O
O
O
O
Me
H
H
O
O
H
S-9
13C NMR (CDCl3, 100 MHz)
230 220 210 200 190 180 170 160 150 140 130 120 110 100 90 80 70 60 50 40 30 20 10 0
f1 (ppm)

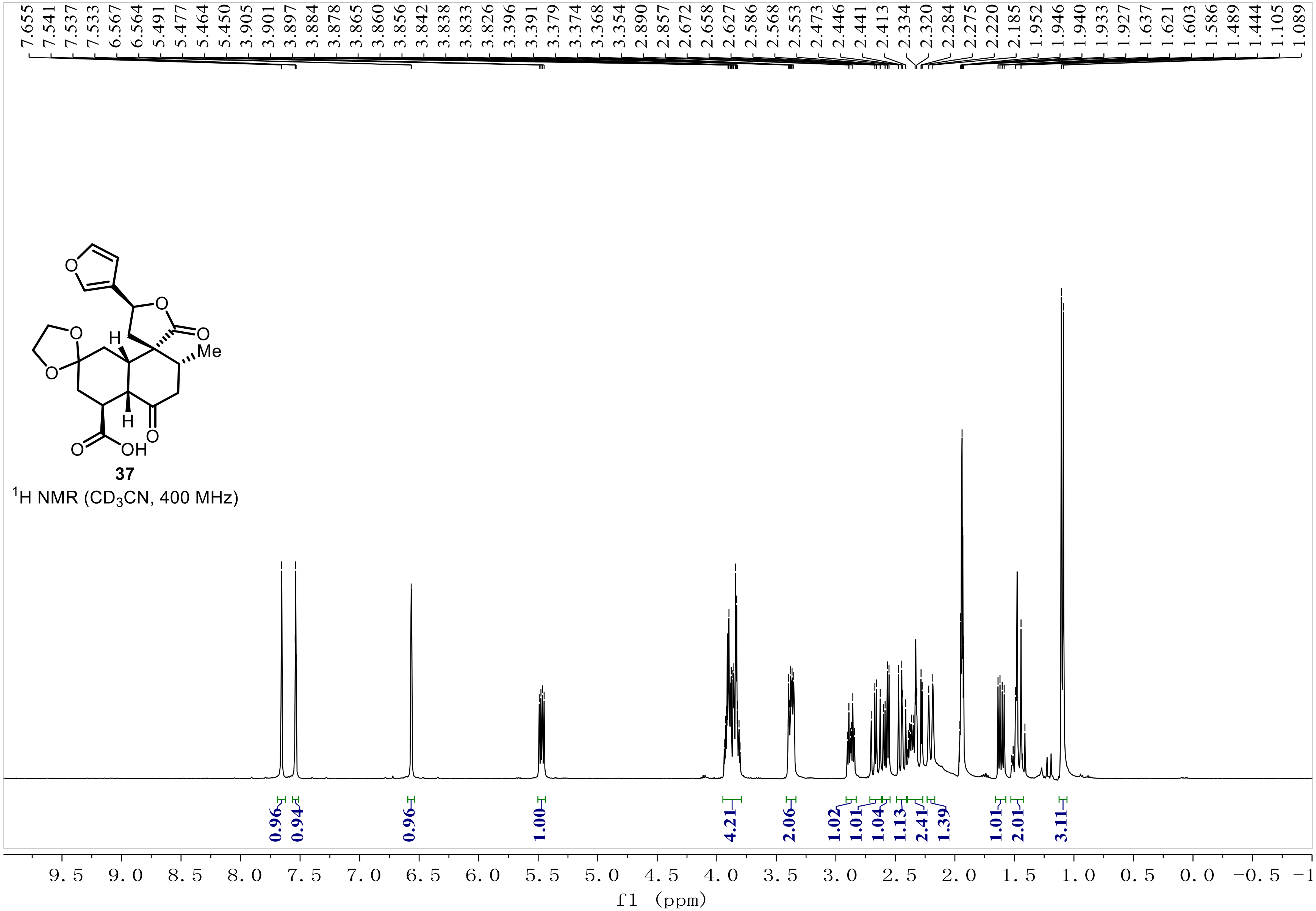

7.655
7.541
7.537
7.533
6.567
6.564
5.491
5.477
5.464
5.450
3.905
3.901
3.897
3.884
3.878
3.865
3.860
3.856
3.842
3.838
3.833
3.826
3.396
3.391
3.379
3.374
3.368
3.354
2.890
2.857
2.672
2.658
2.627
2.586
2.568
2.553
2.473
2.446
2.441
2.413
2.334
2.320
2.284
2.275
2.220
2.185
1.952
1.946
1.940
1.933
1.927
1.637
1.621
1.603
1.586
1.489
1.444
1.105
1.089
Me
37
^{1}H NMR (CD_3CN, 400 MHz)
0.96
0.94
0.96
1.00
4.21
2.06
1.02
1.01
1.04
1.13
2.41
1.39
1.01
2.01
3.11
f1 (ppm)

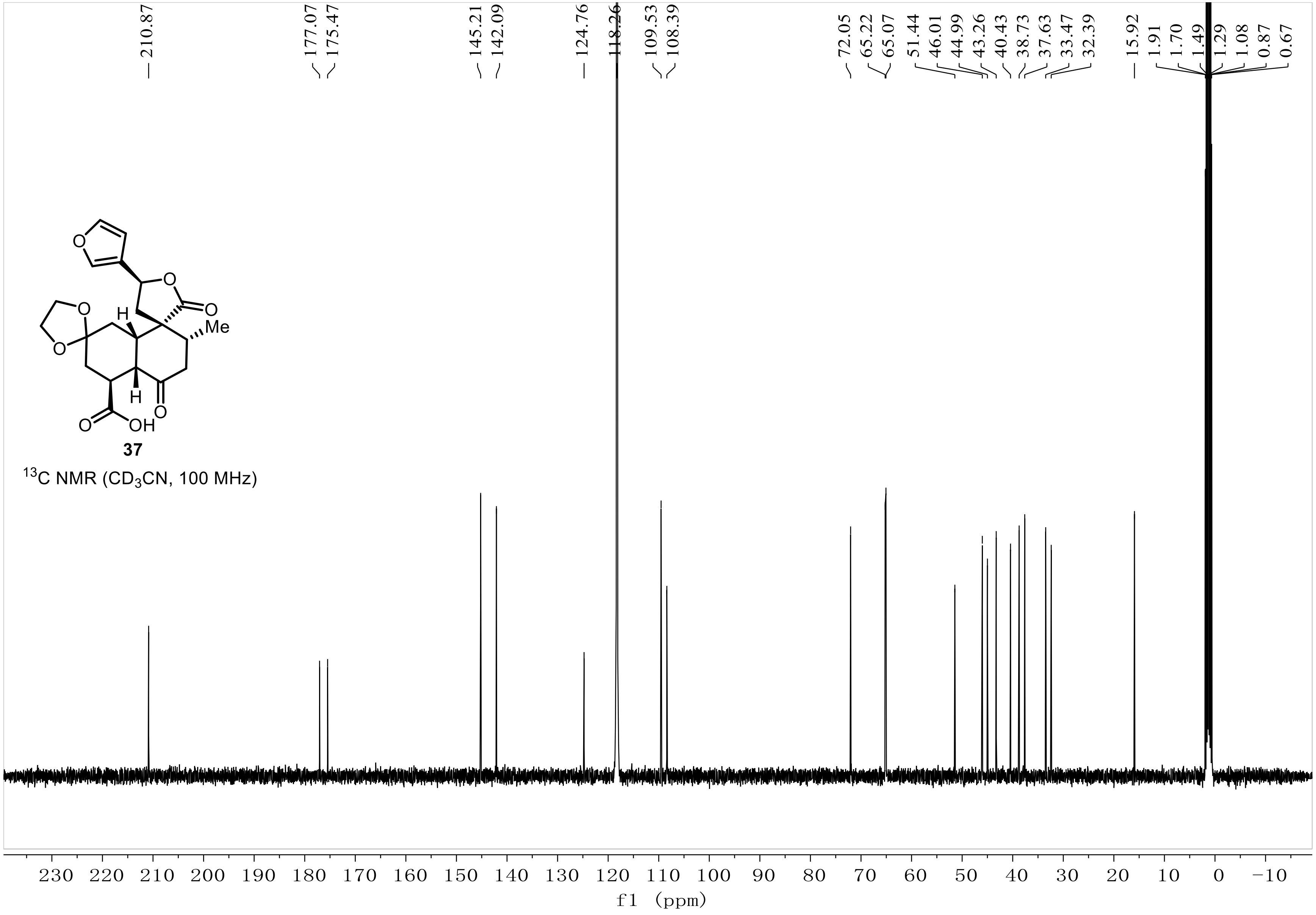
210.87
177.07
175.47
145.21
142.09
124.76
118.26
109.53
108.39
72.05
65.22
65.07
51.44
46.01
44.99
43.26
40.43
38.73
37.63
33.47
32.39
15.92
1.91
1.70
1.49
1.29
1.08
0.87
0.67
37
13C NMR (CD3CN, 100 MHz)
230 220 210 200 190 180 170 160 150 140 130 120 110 100 90 80 70 60 50 40 30 20 10 0 −10
f1 (ppm)

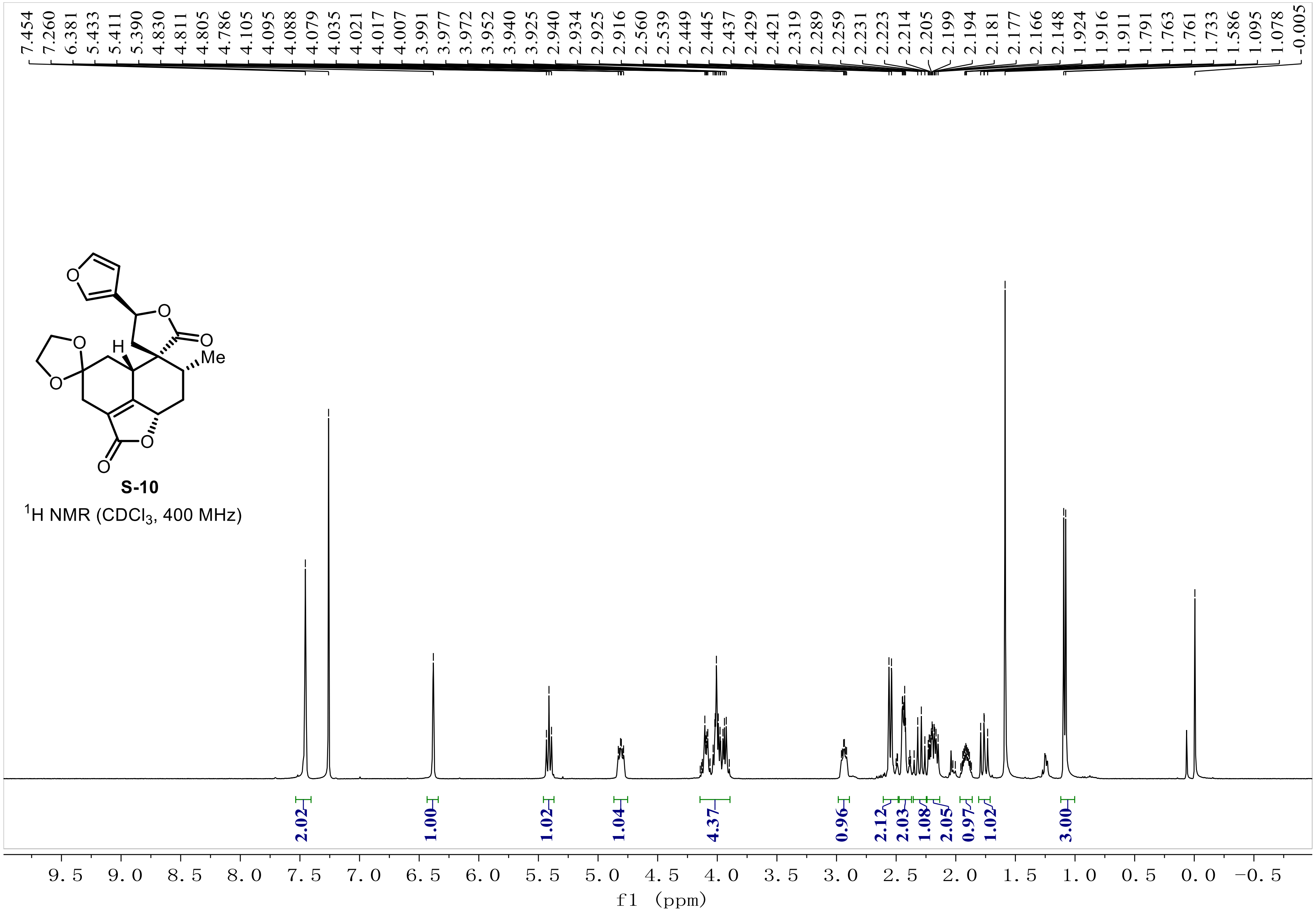
7.454 7.260 6.381 5.433 5.411 5.390 4.830 4.811 4.805 4.786 4.105 4.095 4.088 4.079 4.035 4.021 4.017 4.007 3.991 3.977 3.972 3.952 3.940 3.925 2.940 2.934 2.925 2.916 2.560 2.539 2.449 2.445 2.437 2.429 2.421 2.319 2.289 2.259 2.231 2.223 2.214 2.205 2.199 2.194 2.181 2.177 2.166 2.148 1.924 1.916 1.911 1.791 1.763 1.761 1.733 1.586 1.095 1.078 -0.005
H
Me
S-10
1H NMR (CDCl3, 400 MHz)
2.02 1.00 1.02 1.04 4.37 0.96 2.12 2.03 1.08 2.05 0.97 1.02 3.00
f1 (ppm)

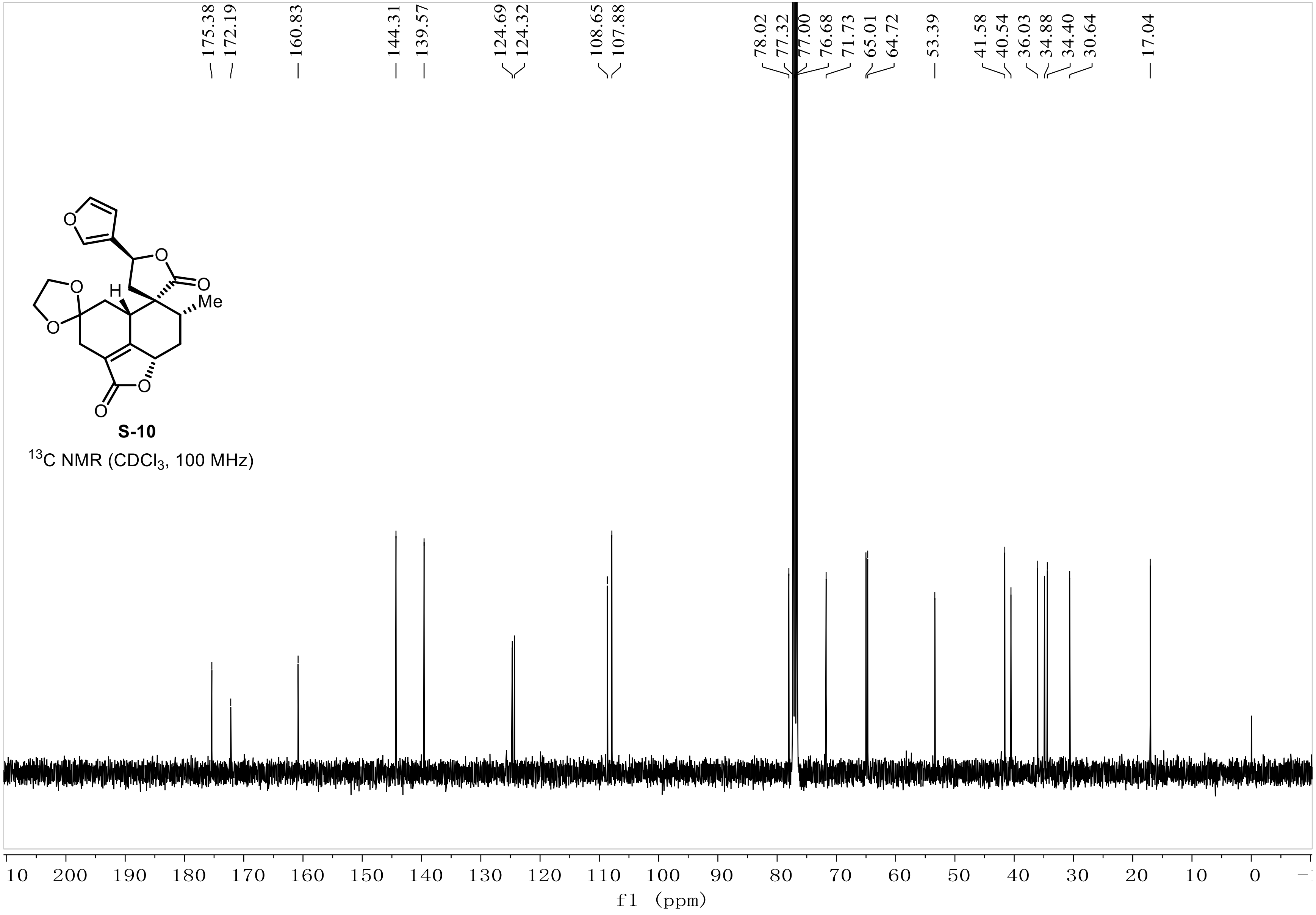

S-10

^{13}C NMR (CDCl$_3$, 100 MHz)

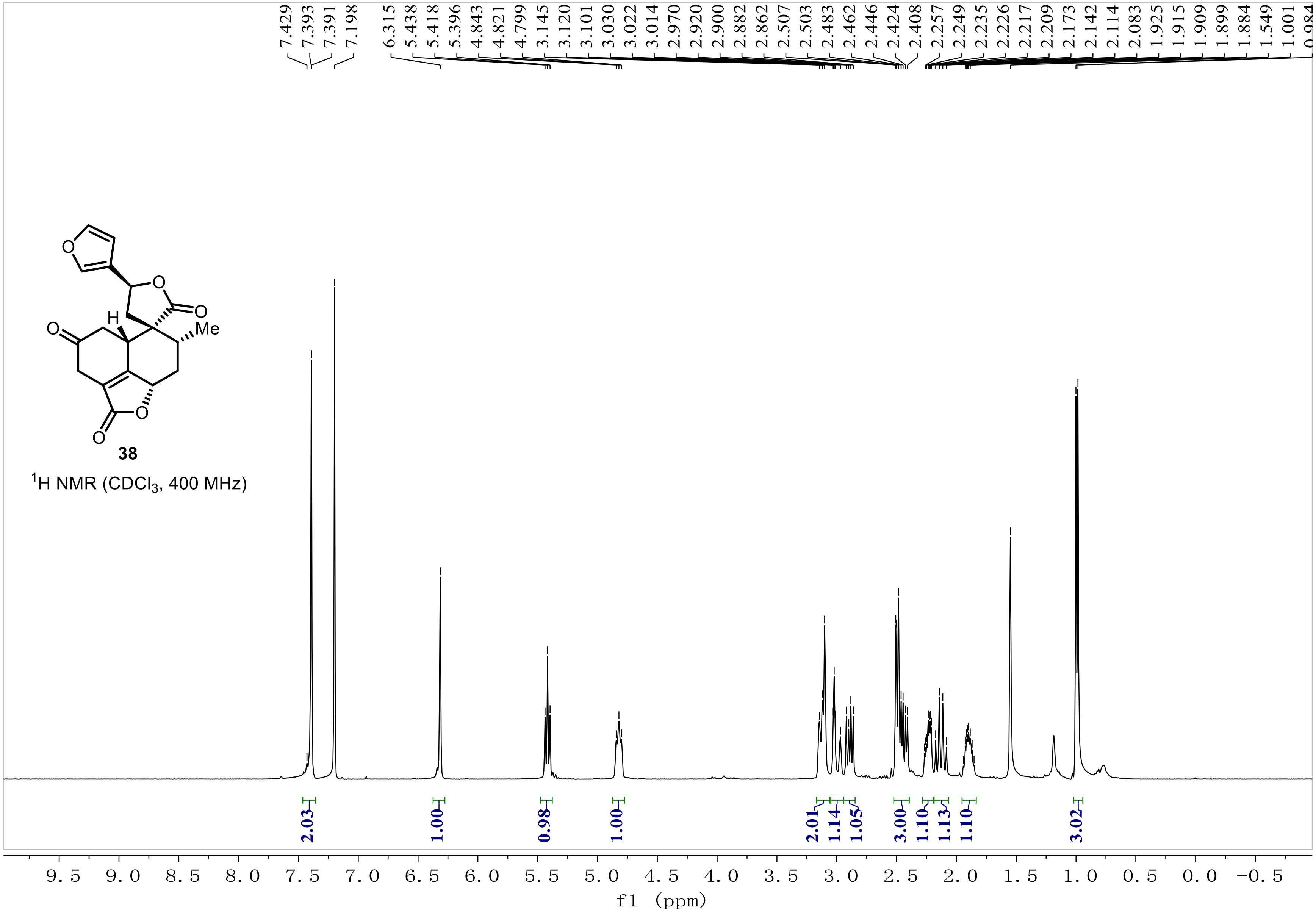
7.429
7.393
7.391
7.198
6.315
5.438
5.418
5.396
4.843
4.821
4.799
3.145
3.120
3.101
3.030
3.022
3.014
2.970
2.920
2.900
2.882
2.862
2.507
2.503
2.483
2.462
2.446
2.424
2.408
2.257
2.249
2.235
2.226
2.217
2.209
2.173
2.142
2.114
2.083
1.925
1.915
1.909
1.899
1.884
1.549
1.001
0.984
Me
H
O
38
1H NMR (CDCl3, 400 MHz)
2.03
1.00
0.98
1.00
2.01
1.14
1.05
3.00
1.10
1.13
1.10
3.02
9.5 9.0 8.5 8.0 7.5 7.0 6.5 6.0 5.5 5.0 4.5 4.0 3.5 3.0 2.5 2.0 1.5 1.0 0.5 0.0 -0.5
f1 (ppm)

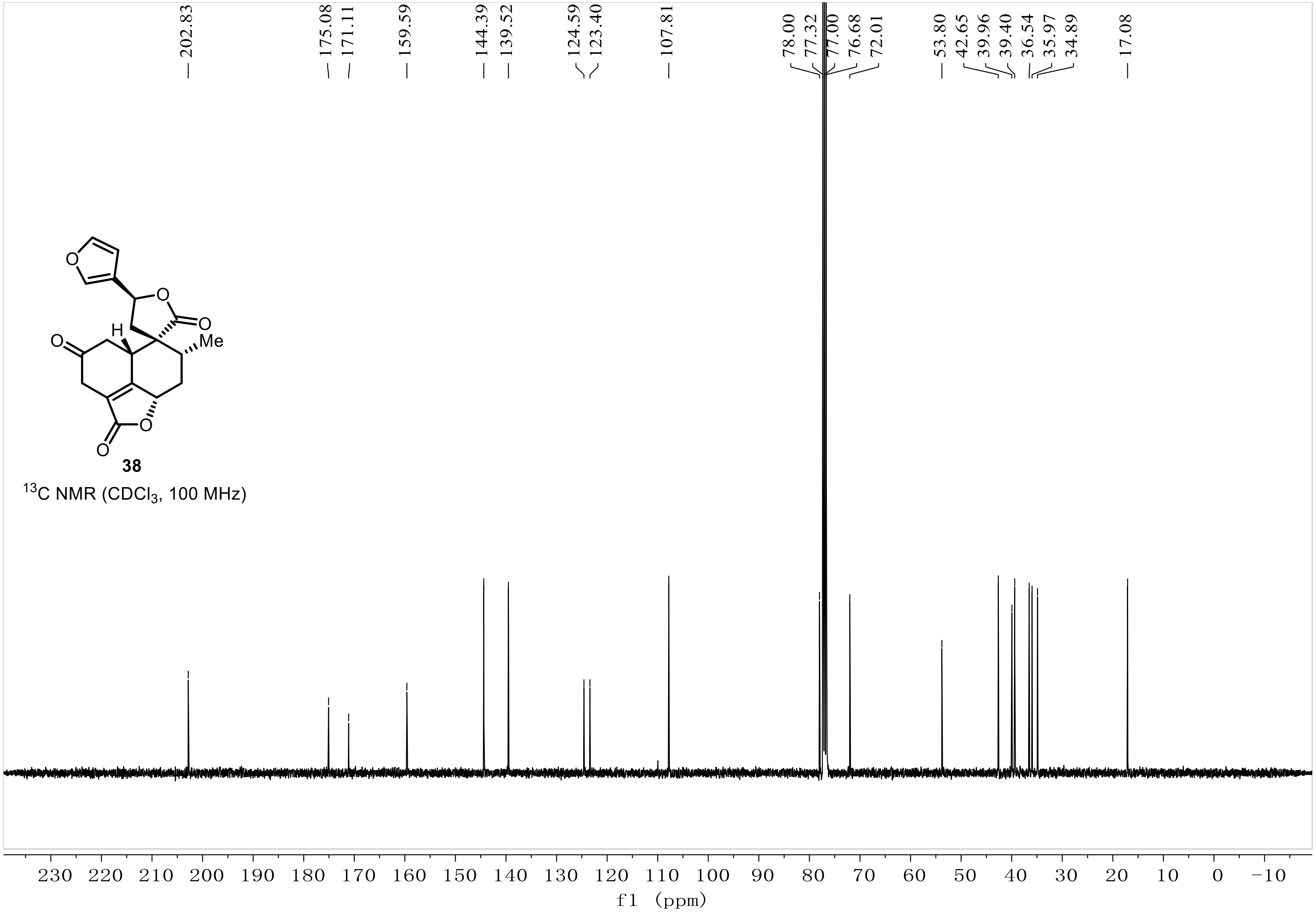
202.83
175.08
171.11
159.59
144.39
139.52
124.59
123.40
107.81
78.00
77.32
77.00
76.68
72.01
53.80
42.65
39.96
39.40
36.54
35.97
34.89
17.08
O
O
O
O
H
Me
O
O
38
¹³C NMR (CDCl₃, 100 MHz)
230 220 210 200 190 180 170 160 150 140 130 120 110 100 90 80 70 60 50 40 30 20 10 0 −10
f1 (ppm)

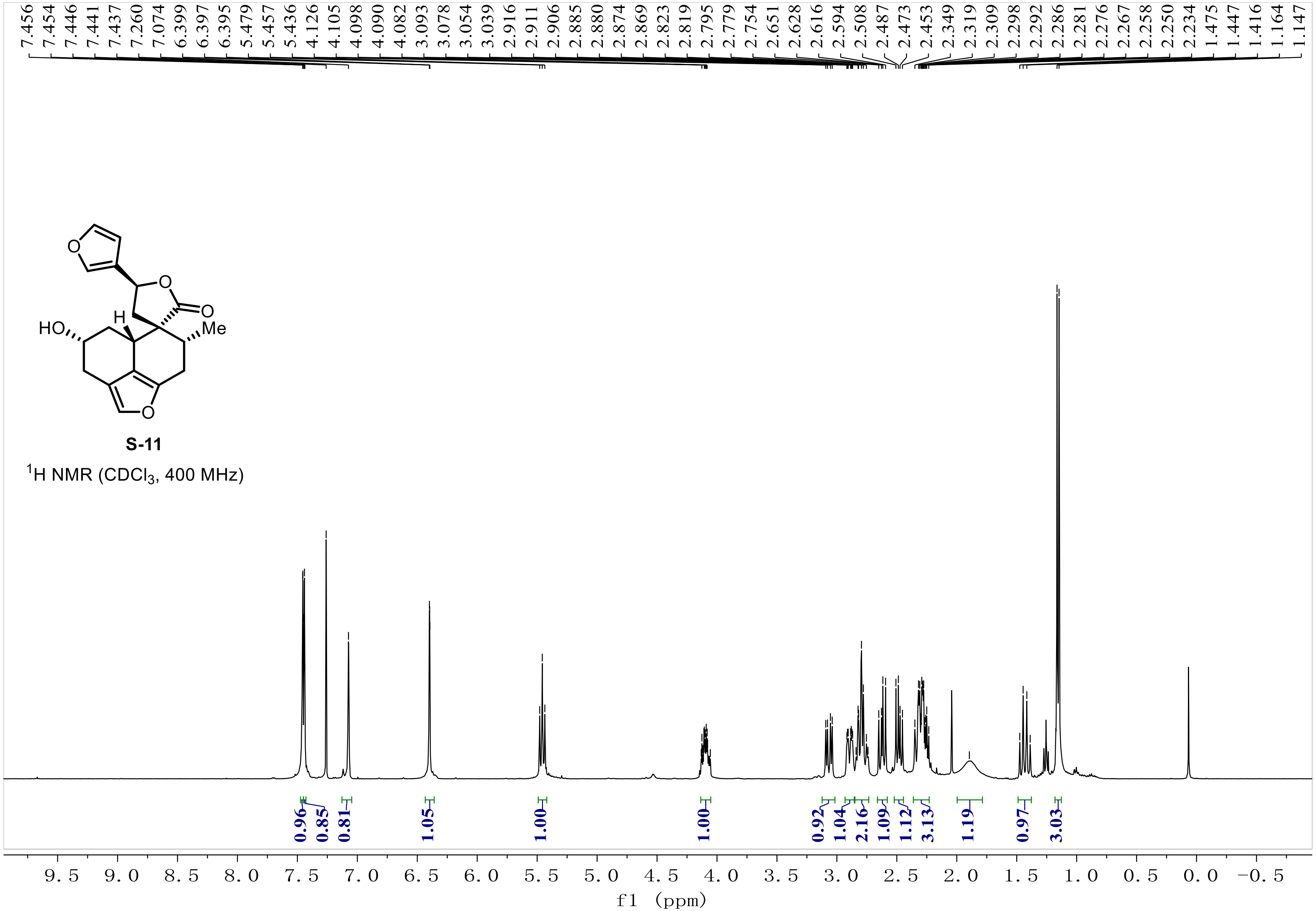
7.456 7.454 7.446 7.441 7.437 7.260 7.074 6.399 6.397 6.395 5.479 5.457 5.436 4.126 4.105 4.098 4.090 4.082 3.093 3.078 3.054 3.039 2.916 2.911 2.906 2.885 2.880 2.874 2.869 2.823 2.819 2.795 2.779 2.754 2.651 2.628 2.616 2.594 2.508 2.487 2.473 2.453 2.349 2.319 2.309 2.298 2.292 2.286 2.281 2.276 2.267 2.258 2.250 2.234 1.475 1.447 1.416 1.164 1.147
O
O
O
HO
H
Me
O
S-11
^{1}H NMR ($CDCl_3$, 400 MHz)
0.96 0.85 0.81 1.05 1.00 1.00 0.92 1.04 2.16 1.09 1.12 3.13 1.19 0.97 3.03
9.5 9.0 8.5 8.0 7.5 7.0 6.5 6.0 5.5 5.0 4.5 4.0 3.5 3.0 2.5 2.0 1.5 1.0 0.5 0.0 -0.5
f1 (ppm)

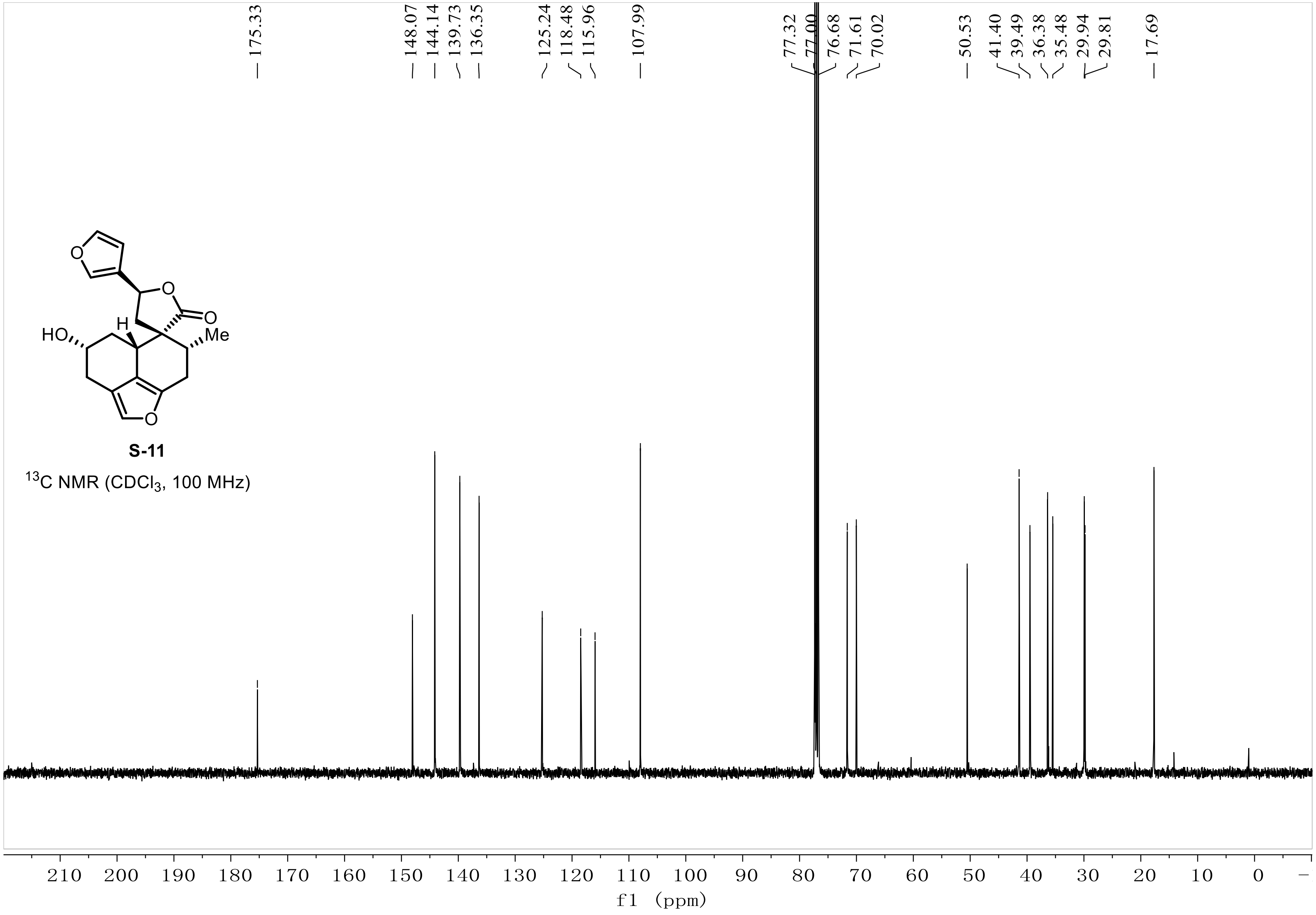
175.33
148.07
144.14
139.73
136.35
125.24
118.48
115.96
107.99
77.32
77.00
76.68
71.61
70.02
50.53
41.40
39.49
36.38
35.48
29.94
29.81
17.69
HO
H
O
Me
S-11
^{13}C NMR ($CDCl_3$, 100 MHz)
210 200 190 180 170 160 150 140 130 120 110 100 90 80 70 60 50 40 30 20 10 0
f1 (ppm)

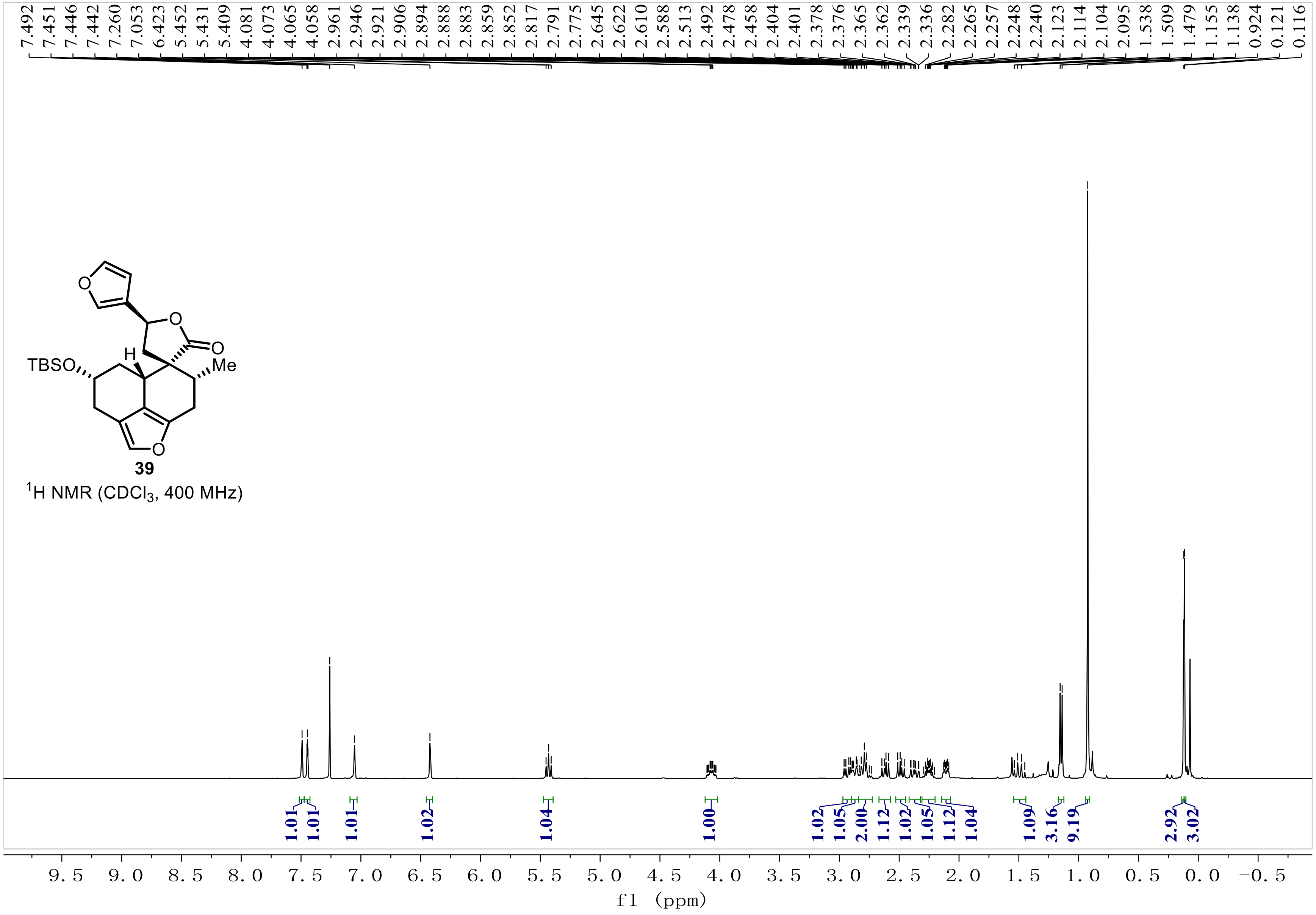
7.492 7.451 7.446 7.442 7.260 7.053 6.423 5.452 5.431 5.409 4.081 4.073 4.065 4.058 2.961 2.946 2.921 2.906 2.894 2.888 2.883 2.859 2.852 2.817 2.791 2.775 2.645 2.622 2.610 2.588 2.513 2.492 2.478 2.458 2.404 2.401 2.378 2.376 2.365 2.362 2.339 2.336 2.282 2.265 2.257 2.248 2.240 2.123 2.114 2.104 2.095 1.538 1.509 1.479 1.155 1.138 0.924 0.121 0.116
TBSO
H
Me
O
39
1H NMR (CDCl3, 400 MHz)
1.01 1.01 1.01 1.02 1.04 1.00 1.02 1.05 2.00 1.12 1.02 1.05 1.12 1.04 1.09 3.16 9.19 2.92 3.02
9.5 9.0 8.5 8.0 7.5 7.0 6.5 6.0 5.5 5.0 4.5 4.0 3.5 3.0 2.5 2.0 1.5 1.0 0.5 0.0 -0.5
f1 (ppm)

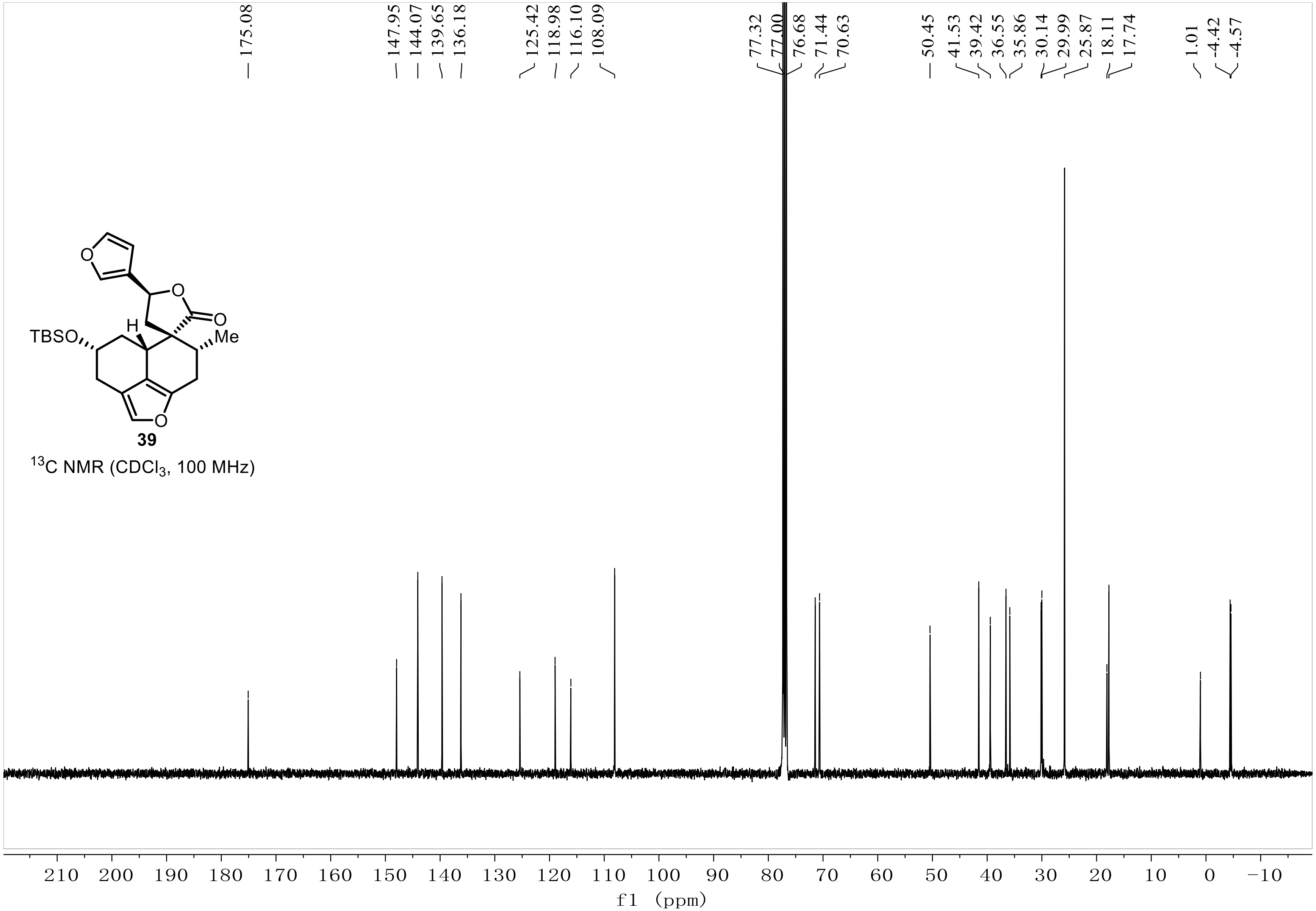
175.08
147.95
144.07
139.65
136.18
125.42
118.98
116.10
108.09
77.32
77.00
76.68
71.44
70.63
50.45
41.53
39.42
36.55
35.86
30.14
29.99
25.87
18.11
17.74
1.01
-4.42
-4.57
TBSO
H
Me
39
^{13}C NMR ($CDCl_3$, 100 MHz)
210 200 190 180 170 160 150 140 130 120 110 100 90 80 70 60 50 40 30 20 10 0 −10
f1 (ppm)

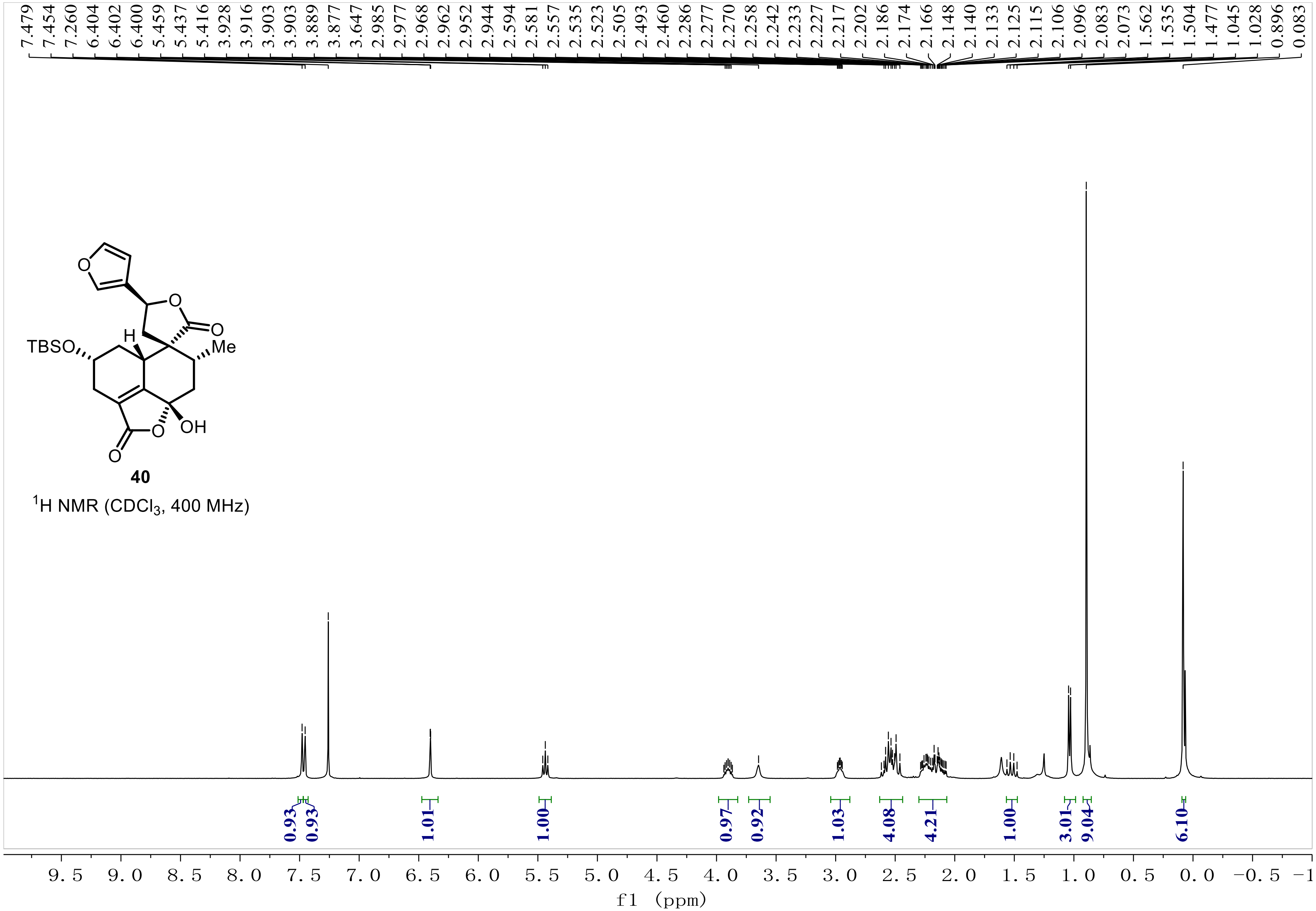
7.479 7.454 7.260 6.404 6.402 6.400 5.459 5.437 5.416 3.928 3.916 3.903 3.903 3.889 3.877 3.647 2.985 2.977 2.968 2.962 2.952 2.944 2.594 2.581 2.557 2.535 2.523 2.505 2.493 2.460 2.286 2.277 2.270 2.258 2.242 2.233 2.227 2.217 2.202 2.186 2.174 2.166 2.148 2.140 2.133 2.125 2.115 2.106 2.096 2.083 2.073 1.562 1.535 1.504 1.477 1.045 1.028 0.896 0.083
O
O
O
TBSO
H
Me
OH
O
O
40
1H NMR (CDCl3, 400 MHz)
0.93 0.93 1.01 1.00 0.97 0.92 1.03 4.08 4.21 1.00 3.01 9.04 6.10
9.5 9.0 8.5 8.0 7.5 7.0 6.5 6.0 5.5 5.0 4.5 4.0 3.5 3.0 2.5 2.0 1.5 1.0 0.5 0.0 -0.5 -1
f1 (ppm)

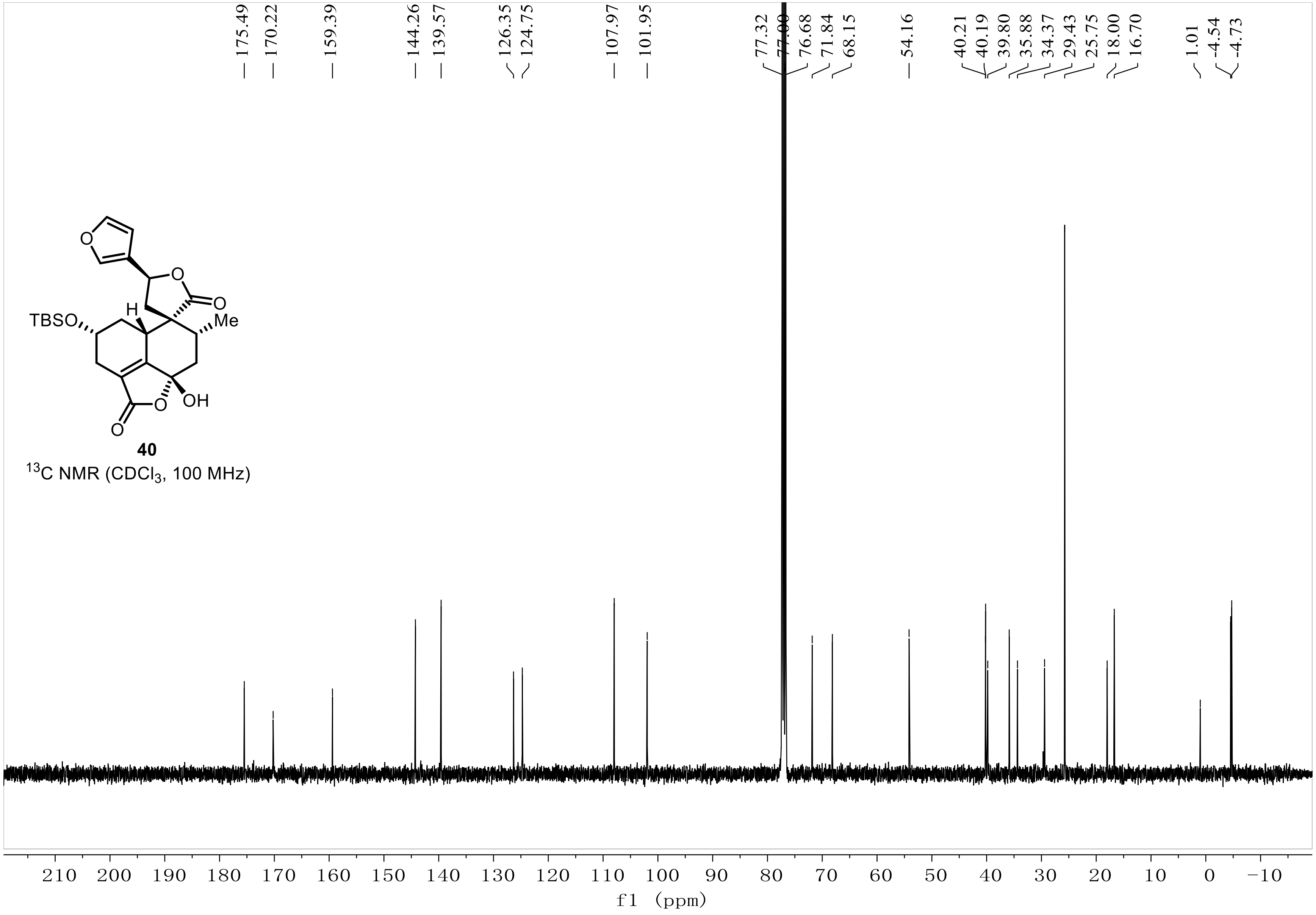
175.49
170.22
159.39
144.26
139.57
126.35
124.75
107.97
101.95
77.32
77.00
76.68
71.84
68.15
54.16
40.21
40.19
39.80
35.88
34.37
29.43
25.75
18.00
16.70
1.01
-4.54
-4.73
TBSO
H
O
O
Me
OH
O
O
O
40
^{13}C NMR ($CDCl_3$, 100 MHz)
210 200 190 180 170 160 150 140 130 120 110 100 90 80 70 60 50 40 30 20 10 0 −10
f1 (ppm)

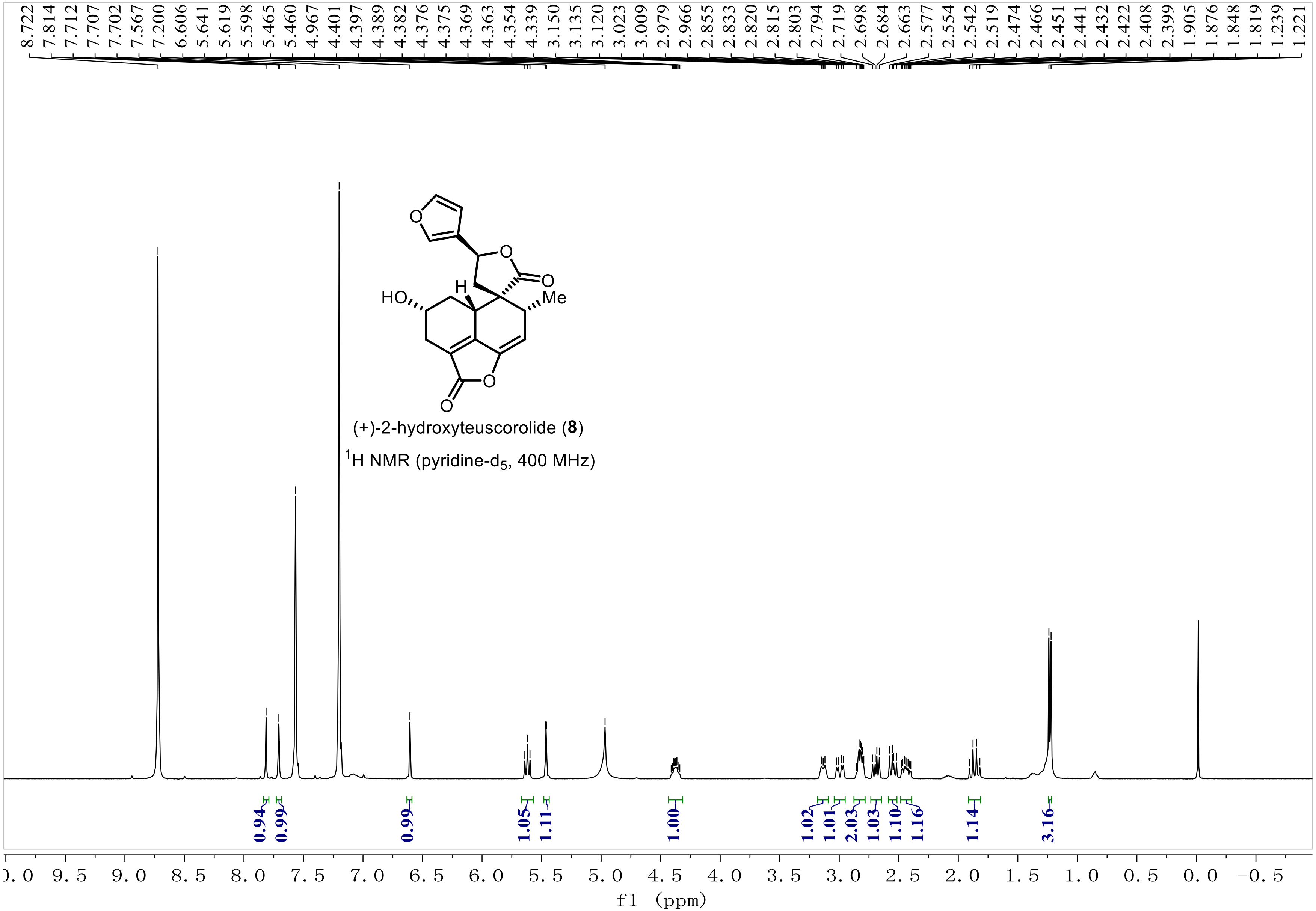
8.722 7.814 7.712 7.707 7.702 7.567 7.200 6.606 5.641 5.619 5.598 5.465 5.460 4.967 4.401 4.397 4.389 4.382 4.376 4.375 4.369 4.363 4.354 4.339 3.150 3.135 3.120 3.023 3.009 2.979 2.966 2.855 2.833 2.820 2.815 2.803 2.794 2.719 2.698 2.684 2.663 2.577 2.554 2.542 2.519 2.474 2.466 2.451 2.441 2.432 2.422 2.408 2.399 1.905 1.876 1.848 1.819 1.239 1.221
HO
H
O
Me
(+)-2-hydroxyteuscorolide (8)
1H NMR (pyridine-d5, 400 MHz)
0.94 0.99 0.99 1.05 1.11 1.00 1.02 1.01 2.03 1.03 1.10 1.16 1.14 3.16
f1 (ppm)

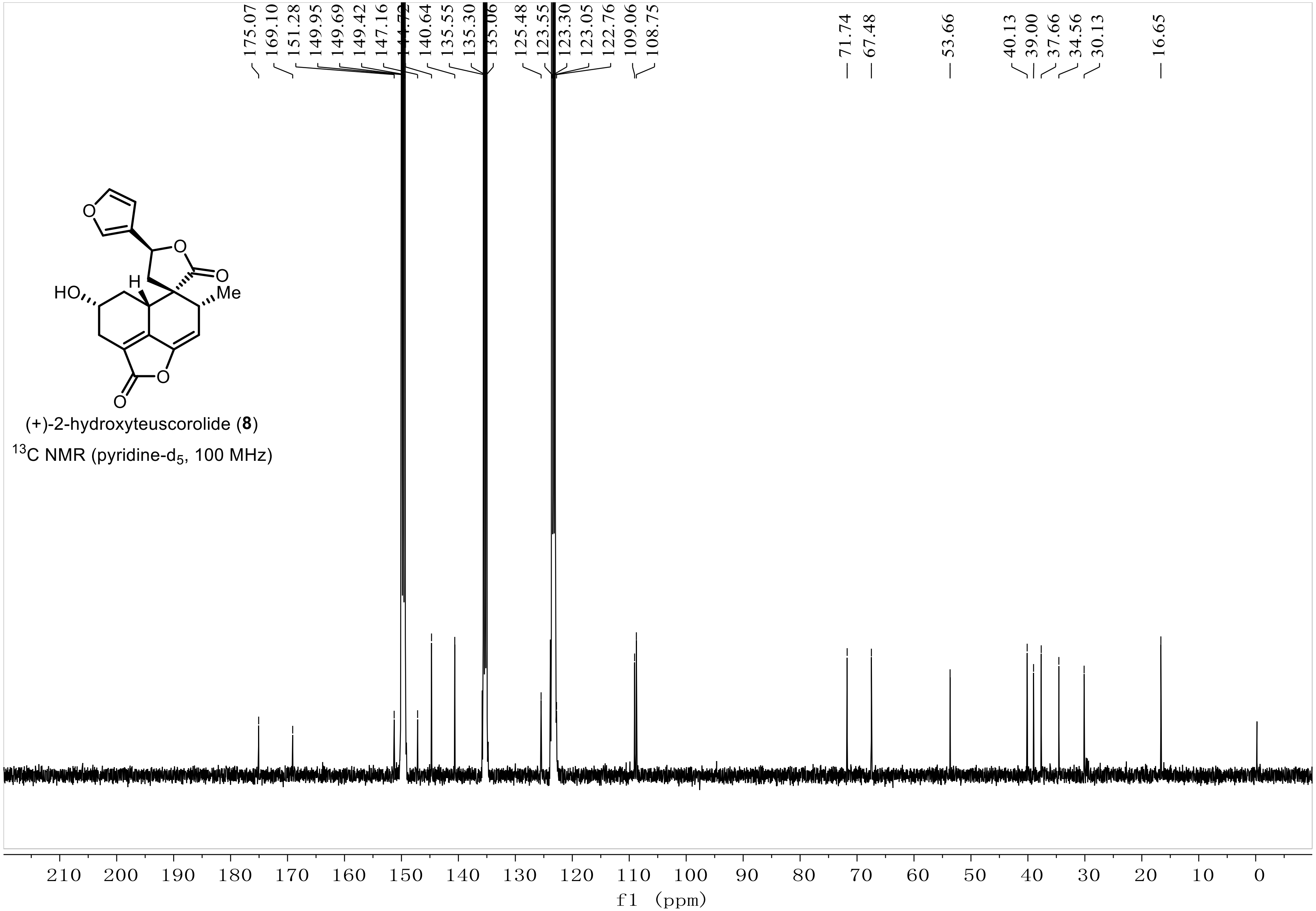
175.07
169.10
151.28
149.95
149.69
149.42
147.16
140.64
135.55
135.30
135.06
125.48
123.55
123.30
123.05
122.76
109.06
108.75
71.74
67.48
53.66
40.13
39.00
37.66
34.56
30.13
16.65
HO
H
O
O
O
Me
O
O
(+)-2-hydroxyteuscorolide (8)
13C NMR (pyridine-d5, 100 MHz)
210 200 190 180 170 160 150 140 130 120 110 100 90 80 70 60 50 40 30 20 10 0
f1 (ppm)
